# Major histocompatibility complex (MHC) fragment numbers alone – in Atlantic cod and in general - do not represent functional variability

**DOI:** 10.12688/f1000research.15386.2

**Published:** 2018-09-06

**Authors:** Johannes M. Dijkstra, Unni Grimholt

**Affiliations:** 1 Institute for Comprehensive Medical Science, Fujita Health University, Toyoake, Aichi, 470-1192, Japan; 2 Fish Research Group, Norwegian Veterinary Institute, Oslo, Oslo N-0106, Norway

**Keywords:** fish, MHC, Atlantic cod, evolution, speciation rate

## Abstract

This correspondence concerns a publication by Malmstrøm *et al.* in Nature Genetics in October 2016. Malmstrøm *et al.* made an important contribution to fish phylogeny research by using low-coverage genome sequencing for comparison of 66 teleost (modern bony) fish species, with 64 of those 66 belonging to the species-rich clade Neoteleostei, and with 27 of those 64 belonging to the order Gadiformes. For these 66 species, Malmstrøm *et al.* estimated numbers of genes belonging to the major histocompatibility complex (MHC) class I lineages U and Z and concluded that in teleost fish these combined numbers are positively associated with, and a driving factor of, the rates of establishment of new fish species (speciation rates). They also claimed that functional genes for the MHC class II system molecules MHC IIA, MHC IIB, CD4 and CD74 were lost in early Gadiformes. Our main criticisms are (1) that the authors did not provide sufficient evidence for presence or absence of intact functional MHC class I or MHC class II system genes, (2) that they did not discuss that an MHC subpopulation gene number alone is a very incomplete measure of MHC variance, and (3) that the MHC system is more likely to reduce speciation rates than to enhance them. Furthermore, their use of the Ornstein-Uhlenbeck model is a typical example of overly naïve use of that model system. In short, we conclude that their new model of MHC class I evolution, reflected in their title “Evolution of the immune system influences speciation rates in teleost fish”, is unsubstantiated, and that their “pinpointing” of the functional loss of the MHC class II system and all the important MHC class II system genes to the onset of Gadiformes is preliminary, because they did not sufficiently investigate the species at the clade border.

## Correspondence

In the below, we explain our criticisms of the Malmstrøm *et al.* ^1^ article as they are summarized in our abstract.

*When was the MHC class II system lost in Gadiformes?* The data as presented by Malmstrøm *et al.* ^1^ suggest a simultaneous loss of major histocompatibility complex (MHC) IIA, MHC IIB, CD4 and CD74 functions at the evolutionary onset of Gadiformes (see their Figure 2). However, within their datasets for gadiform fishes, sequence reads that represent these genes can readily be detected ( Table S1 and Supplementary File 1). These sequence read numbers are much lower than found for the non-gadiform fish, and they may be contaminations, but that should be appropriately tested. Meanwhile, for several non-gadiform fishes, including for *S. chordatus* which among the investigated fishes is the species closest related to Gadiformes, there are no full-length *MHC IIA*, *MHC IIB*, *CD4* or *CD74* gene sequences in the unitig and scaffold datasets presented by Malmstrøm *et al.* ^1^ ( Supplementary File 2 and Table S2). We agree with the conclusion by Malmstrøm *et al.* ^1^ that their data suggest that throughout Gadiformes there is no canonical MHC class II system. However, as for the evolutionary timings of the loss of an intact MHC class II system and of the losses of the individual MHC class II system genes, we find their study technically wanting and preliminary. The combination of (i) not finding intact full-length sequences for all important MHC class II system genes in species closely related to Gadiformes, while (ii) finding reads of these genes in gadiform fishes, prohibits what the authors call “pinpointing the loss of MHC II pathway genes to the common ancestor of Gadiformes”. At least for a few species at either side of the Gadiformes clade border, Malmstrøm *et al.* ^1^ should have substantiated their claims by addition of specific PCR plus sequencing analyses, which should confirm presence of full-length intact MHC class II genes in the non-gadiform fishes, and their absence in the gadiform fishes.

*Discussion of the MHC class I counting strategy by Malmstrøm et al. ^1^* Whereas our criticisms of the MHC class II system analysis by Malmstrøm *et al.* ^1^ are about technical issues and the preliminary character of their conclusions, we more fundamentally disagree with their analyses and discussions of MHC class I. The authors assumed ^1^, as postulated by other researchers before them, that there can be a “copy number optimum” of MHC genes affected by a tradeoff between a higher number allowing the presentation of more pathogen antigens while also having a depletion effect on the T cell population. Regardless of the extent to which this mostly theoretical concept is true ^2^, the MHC counting strategy by Malmstrøm *et al.* ^1^ should be deemed incomplete and far too simplistic. For their number determination Malmstrøm *et al.* ^1^ solely relied on estimation of U plus Z lineage genomic α3 exon fragment numbers, despite that the typical “birth and death” mode of MHC evolution can produce many pseudogenes ^3^. The decision of the authors to only count U plus Z lineage gene fragments was based on their unsubstantiated perception that (neo-)teleost U and Z molecules “predominantly” bind peptide ligands ^1^. However, not all teleost U and Z molecules are expected to present peptides ^4, 5^, for example this is not expected for the majority of U lineage molecules in the neoteleost fish medaka ^6^ and the non-neoteleost fish rainbow trout ^7, 8^; how this is in the majority of the species investigated by Malmstrøm *et al.* ^1^ remains to be determined. Furthermore, it should be realized that MHC class II and non-peptide-binding MHC class I molecules (like maybe teleost molecules of the MHC class I lineages L, P and S ^4^) also can contribute to T cell depletion ^e.g. 9^. Peculiarly, while from their referencing it follows that Malmstrøm *et al.* ^1^ were aware of an MHC class II impact on T cell depletion, the authors did not look at MHC class II when investigating their optimum MHC number model. A more general shortcoming of the article ^1^ is the lack of awareness that the direct determiner of the levels of “antigen coverage” and T cell depletion is the variation between the relevant MHC molecules ^2^, rather than merely the MHC gene copy number. Table 1 (with detailed explanations in Supplementary File 3) summarizes that different teleost fish species can have very different levels of variation between MHC molecules ^4^, and that despite its many U lineage gene copies the extent of MHC variation in Atlantic cod can be considered as relatively limited. Previously, we showed that salmon, zebrafish and eel share variation in U lineage sequences, dating from probably more than 300 million years ago (MYA), whereas all U lineage variation found within the neoteleost fishes stickleback and Atlantic cod probably was established only after these two species separated around 150 MYA ^4^. Without experimental evidence, it cannot simply be assumed that “antigen coverage” and/or T cell depletion are highest in fishes with the highest counts of U plus Z α3 fragments, while neglecting levels of variance among the intact U and Z molecules and possible presences of other categories of MHC molecules. As a last critical comment we point out that, in stark contrast to the evolution of any other known MHC lineage, most deduced Z lineage molecules are characterized by a putative peptide binding groove which was almost perfectly conserved since >400 MYA ^4^; this questions the model by Malmstrøm *et al.* ^1^ that Z lineage evolution was driven by pathogen antigen variation, and is a further argument against the use of combined U+Z numbers for analysis of MHC evolution.

**Table 1.** Intra-species major histocompatibility complex (MHC) variation differs among teleost fishes. This table shows the lowest percentages of amino acid sequence identities between membrane-distal domains (α1+α2 for MHC I, α1 for MHC IIA, β1 for MHC IIB) of same category MHC molecules found between reported sequences of the same species. In some species no genes for particular categories were found (black boxes), while in other instances only one seemingly intact gene sequence was found (1 sequence) or only pseudogenes were found (pseudogene). A more detailed explanation of this table is provided in Supplementary File 3.

		**Species**		Neoteleostei				
		Zebrafish	Salmon	Medaka	Fugu	Stickleback	Tilapia	Cod
MHC category		*(Danio* *rerio)*	*(Salmo salar)*	*(Oryzias* *latipes)*	*(Takifugu* *rubripes)*	*(Gasterosteus* *aculeatus)*	*(Oreochromis* *niloticus)*	*(Gadus* *morhua)*
**MHC class I**	U classical	40%	47%	52%	75%	76%	52%	58%
	U all	27%	38%	32%	29%	62%	27%	58%
	Z	70%	84%	84%	1 sequence	1 sequence	78%	89%
	L	27%	51%				1 sequence	
	P		pseudogene		85%			1 sequence
	S		99%					
**MHC class II**	DA IIA	34%	84%	48%	72%	64%	39%	
	DA IIB	34%	76%	56%	76%	57%	46%	
	DB IIA	23%	20%	20%		1 sequence	21%	
	DB IIB	31%	25%	26%		1 sequence	22%	
	DE IIA		99%					
	DE IIB		99%					

*Overall discussion of the model by Malmstrøm et al. ^1^ saying that U+Z numbers in teleost fish affect speciation rates and that the half-life for reaching the U+Z optimum number is 23 million years.* Malmstrøm *et al.* ^1^ postulated their multiple-regime Ornstein-Uhlenbeck model with very slow progress towards optimum MHC numbers because it was the best fitting model among the few models that they tested. However, an even better fitting model would be that in each species the respective optimal U and Z gene organizations were achieved. Further criticism is that their calculation methods for optimum U+Z numbers and half-life periods incorporated calculations of U+Z gene multiplication speeds, which suffered from the fact that (like for their other considerations) Malmstrøm *et al.* considered all U and Z genes as identical mathematical units ^1^. For such speed calculations U and Z genes should have been studied separately, and it also should have been realized that whereas from some U or Z genes multiple new copies were generated, others were lost in accordance with the “MHC gene birth and death” model ^3^. Lastly, even if, regardless of the discussable calculations for speeds and optimum numbers, there is a positive association in neoteleost fish between speciation rates and U+Z α3 fragment numbers (see their Figure 3), then still their model which considers MHC genes as “speciation genes that promote rapid diversification” ^1^ would be implausible in regard to cause and effect. Namely, in most species, there is a strong evolutionary pressure to maintain old allelic variation within MHC genes (trans-species polymorphism ^3, 4, 10^), which, if anything, is likely to slow down speciation rates because it increases the required size of the founder population ^10^. If old allelic or haplotype variation can’t be maintained because of rapid speciation through small founder populations, it can be speculated that a species might benefit from an enhanced capacity for the creation of new MHC allelic and/or haplotype variation by duplications/deletions and recombination ^11^ between a high number of linked MHC gene copies. However, in that scenario it wouldn’t be the MHC organization which drives the speciation rate, as suggested by Malmstrøm *et al.* ^1^, but the other way around.

*Detailed discussion of the use of the Ornstein-Uhlenbeck model by by Malmstrøm et al.* ^1^. The manner in which Malmstrøm *et al.* ^1^ used the multiple-regime Ornstein-Uhlenbeck (OU) model concerns model fitting rather than model testing. The few restrictions within this system, because multiple regimes are allowed, and the lack of testing of the conclusions with independent datasets, makes the OU-based conclusions by Malmstrøm *et al.* ^1^ statistically wanting. Cooper *et al.* (2016) ^12^ listed limitations of, and recommendations for, meaningful use of the OU modeling system. The Malmstrøm *et al.* ^1^ study did not comply with those recommendations, namely:

1. Cooper *et al.* ^12^ found that OU model error rates are unacceptably high when tree size is small (< 200 species tips). Malmstrøm *et al.* ^1^ only used 66 species tips for their OU calculations of U+Z α3 fragment optima.

2. Cooper *et al.* ^12^ concluded that the system can be strongly affected by measurement errors. Comparison between different studies questions the reliability of the estimation of 80 U lineage α3 fragment per haploid cod genome by Malmstrøm *et al.* ^1^. Namely, with several of the same authors, an earlier study concluded approximately 100 U lineage α3 fragments per haploid genome based on qPCR analysis (Star *et al.*, 2011 ^13^), and in a later study ^14^, upon improved analysis of Atlantic cod genomic scaffold sequences by Tørresen *et al.* (2018 ^14^), a similar set of authors could only detect 13 different sequences with α1+α2+α3 fragment combinations, 13 with only α1, 7 with only α2, 16 with only α3, and 4 with α2+α3 fragments. Based on those various findings, Tørresen *et al.* ^14^ stated that they found 53 copies of U lineage genes (sum of all hits) and that those represented 76% of the number previously estimated by Malmstrøm *et al.* ^1^, which according to Tørresen *et al.* ^14^ was 70. This appears to be an incorrect statement, because, according to their Supplementary Table 3 ^1^, Malmstrøm *et al.* ^1^ concluded a copy number of approximately 80.1 (and not 70) α3 fragments per haploid genome, while Tørresen *et al.* ^1^ only found 20 different sequences with α3 fragments per diploid genome which theoretically could be derived from only 10 loci. Incorporation of measurement errors into the OU modeling by Malmstrøm *et al.* ^1^ are not only suggested by comparison of various studies on cod MHC class I numbers, but also by their ^1^ *Hox* gene copy analysis. Namely, they ^1^ tried to validate the copy number estimation procedure by estimating the “relatively conserved number” of *Hox* gene copies for all investigated species. However, whereas their ^1^ estimation of 50.3 *Hox* copies average among the investigated fish species may approximate the biological reality, without a reasonable biological explanation the finding of variation in *Hox* copy numbers between 30 and 99 in different species (see their Supplementary Figure 3 and Supplementary Table 3 ^1^) suggests measurement errors inherent in their analytical system. The fact that Malmstrøm *et al.* ^1^ included a *Hox* control revealed their awareness that their Illumina read libraries might not equally represent all genomic regions, and their *Hox* analysis results should not have relieved them of those worries.

3. Cooper *et al.* ^12^ concluded that OU calculations are sensitive to intraspecific variation. MHC class I gene copy numbers can differ widely between individuals (haplotype variation), for example, it was estimated that the U lineage gene copy number in the neoteleost fish tilapia can differ between 11 and 17 per haplotype ^15^. Also, concerning Atlantic cod haplotype variation, the presence/absence of U lineage genes was found ^16^. The availability of copy number variation within species makes it highly unlikely that the half-life for reaching optimum numbers of U+Z α3 fragments is 23 million years in teleost fish, since there is already an existing copy number variation from which can be selected. A period of 23 million years typically includes multiple speciation events (see Table S2).

4. Cooper *et al.* ^12^ recommended that OU-based conclusions should be tested by comparison with other analyses. Table S2 shows that the Malmstrøm *et al.* ^1^ conclusion that species richness is associated with elevated U+Z α3 numbers may agree with their division into clades (“regimes”) used to fit their model, but that such a conclusion does not hold if other branchings in the neoteleost phylogenetic tree are compared ( Table S2). For example, Percomorphaceae excluding Ophidiiformes have more species and a higher average number of U+Z α3 numbers than Ophidiiformes, which agrees with the model, but at the same time Carangiformes appear to have many more species but considerably lower U+Z α3 numbers than Abantiformes ( Table S2), which does not agree with the model. Table S2 shows that there is not a general tendency for the branches with more species to have higher U+Z α3 numbers. The calculated half-life of 23 million years for reaching optimum copy number also should be considered as a reason to abandon the model; namely, the long period would imply that hardly any of the investigated species have the optimum MHC number, whereas the previously discussed haplotype variations between individuals of the same species reveal that the system is quite plastic. After all, the type of gene copy number variation investigated here is generally not about gene duplicates that acquired entirely new functions, but more commonly about duplicated genes that acquired only slightly modified functions (for example presenting a slightly different set of peptides), and genes and pseudogenes which may predominantly serve as a reservoir for increased genomic recombination, or which may have no function at all. For example, the turnover of closely related MHC class I genes during primate evolution has produced a human MHC genomic region with only 3 classical class I genes *HLA-A*, *-B*, and *-C*, 3 nonclassical genes *HLA-E*, *-F* and *-G* that also encode molecules with peptide binding ability, and numerous pseudogenes including *HLA-H*, *-J*, *-K*, *-L*, and *-V*, and additional orphan sequence fragments that together account for 6 MHC class I coding genes and 13 pseudogenes ^17, 18^. In evolution, by recombination events, increase or decrease in numbers of tandemly organized similar genes can go very rapidly (e.g. 19). A half-life of 23 million years also would have trouble explaining how the Atlantic cod has 80 U lineage α3 fragments, whereas the fish *Theragra chalcogramma*, which separated from Atlantic cod only ~3 million years ago, only has 31; this observed difference implies very rapid large changes in copy numbers.

*Additional criticisms in regard to the modelling by Malmstrøm et al.* ^1^.

There are several more important criticisms that can be made about the modelling by Malmstrøm *et al.* ^1^.

(A) Malmstrøm *et al.* ^1^ claimed that their study is about teleosts, but basically (with 64 of 66 investigated species) they only investigated within one clade of teleosts, namely the neoteleosts, and neglected information obtained for fish from other large clades of teleosts.

(B) By focusing solely on the α3 exons, Malmstrøm *et al.* ^1^ investigated the part of MHC class I genes which is the least informative for function.

(C) In teleosts, although probably the least pronounced in neoteleosts, selection on U lineage variation happens at two levels ^4, 20^. In fish like cyprinids and salmonids, extreme evolutionary pressure appears to maintain ancient allelic sequence variation from hundreds of millions of years ago, which extends far beyond the peptide binding groove. Simultaneously, there is also the “normal” balancing/diversifying selection on peptide binding groove variation that has been well described for mammals. It is hard to take any model on teleost MHC class I evolution seriously that does not recognize these two different levels of selection.

(D) Questions also can be raised concerning the reliability of the BAMM method used by Malmstrøm *et al.* ^1^ for analysis of speciation rates ^21^. By using this method, Malmstrøm *et al.* ^1^ found higher speciation rates in species-rich clades than in species-poor clades, which seems to be a logical conclusion. However, when Rabosky, the author of the BAMM method ^22^, applied the method to fish, a “negative relationship between species richness and mean speciation rate” was found, which is counterintuitive and curiously not explained in the respective article ^23^.

(E) Malmstrøm *et al.* ^1^ used a bivalent BiSSE model for determining statistical significance of a positive association between elevated speciation rates and high U+Z α3 numbers. It seems that they speculated that the concluded significance of the finding derived from accelerated speciation in fish lineages where U+Z α3 numbers were higher than a threshold of about 20–25 copies, although that would not explain the overall distribution seen in their Figure 3b. As a potential biological exlanation for their proposed model, they ^1^ suggested that the effect of T cell depletion on hybrid fitness becomes more pronounced in the 20–25 copy range and that this might affect mate choice in species with copy numbers above this threshold, promoting inbreeding and reinforcement. However, their speculation can not explain how some species can have many more than 20–25 copies, for example the >100 copies observed in *Muraenolepis marmoratus*, and why such high copy numbers are not associated with even higher speciation rates (see their Figure 3b).

(F) Malmstrøm *et al.* ^1^ concluded “an optimum of 6.8 MHC I copies for basal branches of the phylogeny, which is in concordance with the hypothesized MHC I repertoire of early gnathostomes”. However, previously estimated U+Z numbers in zebrafish, Mexican tetra (cavefish) and Atlantic salmon were 14, 31 and 14, respectively ^4^, and U+Z α3 copies that Malmstrøm *et al.* ^1^ estimated for *Borostomias antarcticus* were 20, suggesting that the U+Z number was considerably higher than 6.8 at the start of the neoteleost lineage. This should have been acknowledged by the authors.

In regard to the modelling methods used by Malmstrøm *et al.* ^1^, we also recommend readers to read the extensive critical comments by reviewer Dr. Jerzy Kulski, which he placed under the first version of our manuscript. In those comments he discusses the models used by Malmstrøm *et al.* ^1^.

## Conclusion

Malmstrøm *et al.* ^1^ used low-coverage genome sequencing for comparison of 66 mostly neoteleost fish, and so helped with elucidating their phylogeny. They found that intact MHC class II system genes may be completely absent in Gadiformes, and believed that related non-gadiform fishes have intact MHC class II system genes. However, their genomic databases were incomplete and in the case of many Gadiformes spiked with reads from MHC class II system genes that may or may not be contaminations, so that final conclusions require some additional analysis of at least a few species at the gadiform/non-gadiform clades border. We suggest that they need to perform a number of PCR and sequencing experiments to clarify this matter. When comparing class I and class II situations in their investigated neoteleosts, Malmstrøm *et al.* ^1^ also found that their earlier theory, which was that the absence of an MHC class II system might explain the high number of MHC class I genes in Atlantic cod ^13^, could not be corroborated. Instead, solely based on estimations of U+Z α3 fragment numbers, they ^1^ proposed a new theory on MHC class I evolution which they referred to in their manuscript title. We hope to have shown sufficiently that their conclusions on MHC class I evolution were unsubstantiated, that estimation of U+Z α3 fragment numbers is not a proper way to analyze MHC functions or MHC evolution, and that, apart from not investigating logical units that are better suited for their methods of modeling, also the number estimations and modelling systems used by Malmstrøm *et al.* ^1^ were flawed and/or non-trustworthy. Before any meaningful discussion can be started about the evolution of MHC class I genes in neoteleosts, a much higher level of information about sequences and genomic positions is necessary.

## Data availability

The datasets analyzed in this study originate from Malmstrøm et al. (2016)1 and are publicly available in the NCBI bioproject https://www.ncbi.nlm.nih.gov/bioproject/PRJEB12469 and in the DRYAD repository https://datadryad.org/resource/doi:10.5061/dryad.326r8. Details are explained in Supplementary File 1, Supplementary File 2 and Supplementary File 3.

## Supplementary material

Supplementary Table S1: Examples of sequence reads of major histocompatibility complex (MHC) class II system genes found in single read archive (SRA) datasets published by Malmstrøm *et al*. for Gadiformes and closely related fishes.

Supplementary Table 2: Species numbers in teleost clades, and estimated U and Z a3 copy numbers in teleost species, investigated by Malmstrøm and co-workers.

Supplementary File 1: List of sequence reads in SRA datasets of Gadiformes published by Malmstrøm *et al.* that match with major histocompatibility complex (MHC) class II system genes.

Supplementary File 2: Investigation of unitigs with (partial) major histocompatibility complex (MHC) class II system genes which are listed by Malmstrøm *et al.* in their Table S7 for the non-gadiform fishes *S. chordatus*, *C. roseus*, *Z. faber*, T *. subterraneus*, *P. transmontana*, and *P. japonica*.

Supplementary File 3: Detailed explanation of Table 1.
