# Supplementary material for: Major histocompatibility complex (MHC) fragment numbers alone – in Atlantic cod and in general - do not represent functional variability
Source: F1000Res. 2018 Sep 6;7:963. Originally published 2018 Jun 28. [Version 2] doi: 10.12688/f1000research.15386.2 (PMC6081975; doi:10.12688/f1000research.15386.2)
Supplement: Supplementary file 1 [file f1000research-7-17714-s0000.tgz › c20cf7a7-ab13-4385-89b1-efc7d1997573.docx]

Table S1. Examples of sequence reads of MHC class II system genes found in single read archive (SRA) datasets published by Malmstrøm *et al.* [1] for Gadiformes and closely related fishes

MHC II alpha

Clade	Order / Family	Species	MHC II alpha (DA lineage) DA-alpha1	MHC II alpha (DA lineage) DA-alpha2 (or DB?)*	MHC II alpha (DB category) DB-alpha1	MHC II alpha (DB category) DB-alpha2 (or DA?)*	MHC II alpha (DE lineage) DE-alpha1	MHC II alpha (DE lineage) DE-alpha2
Paracanthopterygii	Polymixiiformes	Polymixia japonica	ERR1473839.44358697.2	ERR1473839.20025846.2	ERR1473839.33259465.2	ERR1473839.18935083.1	ERR1473839.27534957.1	ERR1473839.23930146.2
	Percopsiformes	Percopsis transmontana	ERR1473840.50880588.1	ERR1473840.45133268.1	ERR1473840.69904655.2	ERR1473840.12901815.2	ERR1473840.42818125.2	ERR1473840.65158873.1
		Typhlichthys subterraneus	ERR1473841.18484405.2	ERR1473841.63862866.2	ERR1473841.36365326.1	ERR1473841.55915884.2	ERR1473841.8926952.2	ERR1473841.41889616.1
	Zeiformes	Zeus faber	ERR1473842.17254250.1	ERR1473842.71948405.1	ERR1473842.29847247.1	ERR1473842.34174392.1	not found	not found
		Cyttopsis roseus	ERR1473843.4968817.2	ERR1473843.3678503.2	ERR1473843.36126730.1	ERR1473843.20707828.2	not found	ERR1473843.64784299.1
	Stylephoriformes	Stylephorus chordatus	ERR1473844.58669768.1	ERR1473844.60703990.1	not found	not found	not found	ERR1473844.21942590.2
Gadiformes	Bregmacerotidae	Bregmaceros cantori	not found	not found	not found	not found	not found	not found
	Merlucciidae	Merluccius polli	not found	not found	not found	not found	not found	not found
		Merluccius merluccius	not found	not found	ERR1473849.16460326.2	ERR1473848.13341992.2	not found	not found
		Merluccius capensis	ERR1473850.24337980.1	ERR1473850.4305135.2	ERR1473850.29286756.2	not found	not found	not found
	Melanonidae	Melanonus zugmayeri	ERR1473851.14541320.1	ERR1473851.1276178.1	ERR1473852.5535820.1	ERR1473851.34596639.2	ERR1473851.28557328.1	ERR1473851.15069226.1
	Muraenolepididae	Muraenolepsis marmoratus	not found	not found	ERR1473853.13532583.1	ERR1473853.35942289.2	not found	not found
	Trachyrincinae	Trachyrincus scabrus	not found	ERR1473855.24170403.1	not found	not found	not found	not found
		Trachyrincus murrayi	not found	not found	not found	not found	not found	not found
	Moridae	Mora moro	not found	not found	not found	not found	not found	not found
		Laemonema laureysi	ERR1473862.42883890.1	ERR1473862.20754527.2	not found	ERR1473862.38712638.2	not found	not found
	Bathyganidae	Bathygadus melanobranchus	not found	ERR1473863.13853066.1	not found	not found	ERR1473863.33589778.2	not found
	Macrourinae	Macrourus berglax	not found	not found	not found	not found	not found	not found
		Malacocephalus occidentalis	not found	not found	not found	not found	not found	not found
Gadidae	Phycinae	Phycis blennoides	ERR1473867.1216359.2	ERR1473869.6893481.1	not found	not found	not found	not found
		Phycis phycis	ERR1473871.51739494.1	not found	not found	not found	not found	not found
	Lotinae	Lota lota	not found	not found	not found	not found	not found	not found
		Molva molva	not found	not found	not found	not found	not found	not found
		Brosme brosme	not found	not found	not found	not found	not found	not found
	Gadinae	Trisopterus minutus	not found	not found	not found	not found	not found	not found
		Gadiculus argenteus	not found	not found	not found	not found	not found	not found
		Pollachius virens	not found	not found	not found	not found	not found	not found
		Melanogrammus aeglefinus	not found	not found	not found	not found	not found	not found
		Merlangius merlangus	not found	not found	not found	not found	not found	not found
		Arctogadus glacialis	not found	not found	not found	not found	not found	not found
		Boreogadus saida	not found	not found	not found	not found	not found	not found
		Theragra chalcogramma	not found	not found	not found	not found	not found	not found
		Gadus morhua	not found	not found	not found	not found	not found	not found

MHC II beta

Species	MHC II beta (DA lineage) DA-beta1	MHC II beta (DA lineage) DA-beta2 (or DB?)*	MHC II beta (DB category) DB-beta1	MHC II beta (DB category) DB-beta2 (or DA?)*	MHC II beta (DE lineage) DE-beta1	MHC II beta (DE lineage) DE-beta2
Polymixia japonica	ERR1473839.815172.1	ERR1473839.22986580.2	ERR1473839.42799920.1	ERR1473839.4576291.2	ERR1473839.34239709.1	not found
Percopsis transmontana	ERR1473840.67666957.1	ERR1473840.51056990.2	ERR1473840.2412803.1	ERR1473840.8212410.2	ERR1473840.63097915.1	ERR1473840.19495824.1
Typhlichthys subterraneus	ERR1473841.29304581.1	ERR1473841.44184621.2	ERR1473841.30644660.1	ERR1473841.37608682.1	ERR1473841.53256578.2	ERR1473841.54337889.2
Zeus faber	ERR1473842.9039535.1	ERR1473842.19180854.2	ERR1473842.64009737.1	not found	not found	not found
Cyttopsis roseus	ERR1473843.74163567.1	ERR1473843.54084847.1	ERR1473843.67102374.1	ERR1473843.22495065.1	not found	not found
Stylephorus chordatus	ERR1473844.9747412.1	ERR1473844.79517300.1	not found	not found	not found	not found
Bregmaceros cantori	not found	not found	not found	not found	not found	not found
Merluccius polli	not found	not found	not found	not found	not found	not found
Merluccius merluccius	ERR1473848.25746747.1	ERR1473849.20183195.2	ERR1473848.48940243.2	ERR1473848.4129854.2	not found	not found
Merluccius capensis	ERR1473850.7430792.2	not found	ERR1473850.3437343.2	ERR1473850.36098825.1	not found	not found
Melanonus zugmayeri	ERR1473851.54447277.1	ERR1473851.48106054.2	ERR1473851.50709829.1	ERR1473851.36199669.2	not found	not found
Muraenolepsis marmoratus	not found	not found	not found	not found	not found	not found
Trachyrincus scabrus	not found	not found	not found	not found	not found	not found
Trachyrincus murrayi	ERR1473859.14956511.1	ERR1473859.5695319.1	not found	not found	not found	not found
Mora moro	not found	not found	not found	not found	not found	not found
Laemonema laureysi	not found	not found	not found	not found	not found	not found
Bathygadus melanobranchus	not found	ERR1473863.7278419.2	not found	not found	not found	not found
Macrourus berglax	not found	not found	not found	not found	not found	not found
Malacocephalus occidentalis	ERR1473866.32278067.2	ERR1473866.37837891.1	not found	not found	not found	not found
Phycis blennoides	not found	not found	not found	ERR1473870.508665.2	not found	not found
Phycis phycis	ERR1473871.42393311.2	ERR1473871.3128027.1	not found	not found	not found	not found
Lota lota	ERR1473872.28413271.1	not found	not found	not found	not found	not found
Molva molva	not found	not found	not found	not found	not found	not found
Brosme brosme	not found	not found	not found	not found	not found	not found
Trisopterus minutus	not found	not found	not found	not found	not found	not found
Gadiculus argenteus	not found	not found	not found	not found	not found	not found
Pollachius virens	not found	not found	not found	not found	not found	not found
Melanogrammus aeglefinus	not found	not found	not found	not found	not found	not found
Merlangius merlangus	not found	not found	not found	not found	not found	not found
Arctogadus glacialis	not found	not found	not found	not found	not found	not found
Boreogadus saida	not found	not found	not found	not found	not found	not found
Theragra chalcogramma	not found	ERR1473886.15640894.2	not found	not found	not found	not found
Gadus morhua	not found	not found	not found	not found	not found	not found

CD4 and CD74

Species	CD4 CD4-1	CD4 CD4-2	CD74 CD74a	CD74 CD74b
Polymixia japonica	ERR1473839.35036371.2	ERR1473839.15269991.2	ERR1473839.25975760.2	ERR1473839.44335506.1
Percopsis transmontana	ERR1473840.44787874.2	ERR1473840.15995881.1	ERR1473840.73517397.1	ERR1473840.42654085.1
Typhlichthys subterraneus	ERR1473841.6035905.2	ERR1473841.16238802.1	ERR1473841.13918841.2	ERR1473841.1078273.1
Zeus faber	ERR1473842.70813063.1	ERR1473842.21842095.1	ERR1473842.51168298.1	not found
Cyttopsis roseus	ERR1473843.16746230.2	ERR1473843.4133665.1	ERR1473843.39754671.1	ERR1473843.22599869.2
Stylephorus chordatus	ERR1473844.50446872.2	ERR1473844.34353759.1	ERR1473844.53854689.2	ERR1473844.80864479.2
Bregmaceros cantori	not found	not found	not found	not found
Merluccius polli	ERR1473847.52770223.2	not found	ERR1473847.19205782.1	not found
Merluccius merluccius	ERR1473849.19576829.1	not found	not found	not found
Merluccius capensis	ERR1473850.33239539.2	ERR1473850.24696544.1	not found	not found
Melanonus zugmayeri	ERR1473851.55141915.1	ERR1473851.41782205.1	ERR1473851.1976448.2	ERR1473851.36808274.1
Muraenolepsis marmoratus	ERR1473853.29378090.1	not found	not found	not found
Trachyrincus scabrus	ERR1473855.9410251.1	not found	not found	ERR1473854.58716562.1
Trachyrincus murrayi	ERR1473858.2717135.1	ERR1473858.1849697.2	ERR1473857.5327458.1	not found
Mora moro	ERR1473861.24477611.2	not found	not found	not found
Laemonema laureysi	ERR1473862.46680409.2	not found	not found	ERR1473862.22060450.1
Bathygadus melanobranchus	ERR1473863.34468851.2	ERR1473863.58116857.2	ERR1473863.46876077.1	not found
Macrourus berglax	ERR1473865.5128973.2	not found	not found	not found
Malacocephalus occidentalis	ERR1473866.4779853.1	not found	ERR1473866.6838980.2	not found
Phycis blennoides	ERR1473867.8606077.2	not found	not found	not found
Phycis phycis	ERR1473871.40208761.1	not found	not found	not found
Lota lota	ERR1473872.30301307.2	not found	not found	not found
Molva molva	ERR1473874.27302905.2	not found	not found	not found
Brosme brosme	ERR1473875.34246991.1	not found	not found	not found
Trisopterus minutus	ERR1473876.48813342	not found	not found	not found
Gadiculus argenteus	ERR1473877.54990389.1	not found	not found	not found
Pollachius virens	ERR1473878.12887793.1	not found	not found	not found
Melanogrammus aeglefinus	ERR1473879.17160361.2	not found	not found	not found
Merlangius merlangus	ERR1473880.24550207.1	not found	not found	not found
Arctogadus glacialis	ERR1473882.53485727.1	not found	not found	not found
Boreogadus saida	ERR1473884.54645318.2	not found	not found	not found
Theragra chalcogramma	ERR1473886.18309701.1	not found	not found	not found
Gadus morhua	ERR1473889.3816183.1	not found	not found	not found

This table shows examples of sequence reads that appear to contain parts of genomic sequences of MHC class II system genes indicated in the upper row and were deposited in the SRA database of NCBI (https://www.nih.gov/) by Malmstrøm *et al.* [1] for the species (-clades) indicated in the left columns. The sequence clones were found by "tblastn" analysis using appropriate search sequences, after which gene identity was confirmed by additional sequence comparisons. MHC class II searches were done separately for each of the domains alpha-1, alpha-2, beta-1 and beta-2, of the classical plus classical-related class II lineage DA, the nonclassical class II category DB, and the nonclassical class II lineage DE (for explanation of DAversus DB and DE see supplementary text S1 and reference[2]). Only one matching sequence read per search is shown, but in most cases sequence reads of more than one matching clone were found. The amino acid sequences encoded by the listed SRAs are shown in Figure S1, where they are aligned with representive sequences of the respective category. *Because the sequence reads are only short (~150 nt) and the Ig-like domain regions are relatively well conserved between the DA and DB categories of MHC class II, for SRAs matching to MHC class II alpha-2 or beta-2 domains we probably can't reliably distinguish between DA or DB identity; DE lineage Ig-like domain sequences have more pronounced distinguishing features (see Fig. S1). In the on-line correspondence section of a previous article by this research group [3] we already discussed that it might need more evidence before concluding that the non-canonical CD4-1 sequence in Atlantic cod is a pseudogene; regardless of whether the Atlantic cod *CD4-1* sequence produces a functional protein, with their current data Malmstrøm *et al.* [1] should have deduced the evolution from consensus CD4-1 in a non-gadiform ancestor to the gene forms found in Gadiformes. Examples of matching sequence reads are described in yellow fields, while white fields with "not found" represent negative findings.

References:

1. Malmstrøm, M. *et al.* Evolution of the immune system influences speciation rates in teleost fishes. *Nat. Genet.* **48**, 1204-1210 (2016).
2. Dijkstra, J.M. *et al.* Comprehensive analysis of MHC class II genes in teleost fish genomes reveals dispensability of the peptide-loading DM system in a large part of vertebrates. *BMC Evol. Biol.* **13**, 260 (2013).
3. Star, B. *et al.* The genome sequence of Atlantic cod reveals a unique immune system. *Nature* **477**, 207-210 (2011).
