# Supplementary material for: Major histocompatibility complex (MHC) fragment numbers alone – in Atlantic cod and in general - do not represent functional variability
Source: F1000Res. 2018 Sep 6;7:963. Originally published 2018 Jun 28. [Version 2] doi: 10.12688/f1000research.15386.2 (PMC6081975; doi:10.12688/f1000research.15386.2)
Supplement: Supplementary file 2 [file f1000research-7-17714-s0004.tgz › ce2dbd68-fe94-4459-869c-d2d2b0713485.pdf]

Table S2. Species numbers in teleost clades, and estimated U and Z α3 copy numbers in teleost species, investigated by Malmstrøm and co-workers.

(for the table legend also see next page)

Clades for which Malmstrøm et al.1 calculated elevated copy number optima	Malmstrøm et al.1 Fig. 1 clades		Species investigated by Malmstrøm et al.1	*estimated[1] Z- α3 copies*	estimated[1] U-α3 copies	Gadiform species numbers according to Nelson 2006[2]	Species numbers according to Davis *et al.* 2016[3] (or Nelson 2006[2], or Wikipedia)
Percomorphaceae excluding Ophidiiformes 25.2		Ovalentariae incertae sedis	*Pseudochromis fuscus*	*3*	6		Ovalentariae incertae sedis
			Chromis chromis	*2*	33		
		Blenniformes	*Parablennius parvicornis*	*1*	8		Blenniformes 1930
		Anabantiformes	*Anabas testudineus*	*4*	51		Wiki: Anabantiformes 252
			Helostoma temminckii	*6*	56		
		Carangiformes	*Selene dorsalis*	*2*	13		Carangiformes 1074
		Lophiiformes	*Antennarius striatus*	*2*	18		Nelson: Lophiiformes 313
		Spariformes	*Spondyliosoma cantharus*	*8*	80		Wiki: Sparidae 155
		Labriformes	*Symphodus melops*	*3*	20		Labriformes 687
		Perciformes	*Chaenocephalus aceratus*	*2*	19		Scorpaeniformes, Centarchiformes, Acropomatiformes and Acanthuriformes 5780
			Sebastes norvegicus	*4*	21		
			Myoxocephalus scorpius	*1*	9		
			Perca fluviatilis	*4*	29		
		Gobiformes	*Lesueurigobius cf. sanzi*	*2*	14		Gobiformes 2379
		Scombriformes	*Thunnus albacares*	*6*	47		Scombriformes and Syngnathiformes 944
		Batrachoidiformes	*Chatrabus melanurus*	*1*	21		Batrachoidiformes 83
		Ophidiiformes	*Carapus acus*	*1*	7		Ophidiiformes 531
			Lamprogrammus exutus	*1*	5		
			Brotula barbata	*1*	15		
		Beryciformes	*Acanthochaenus luetkenii*	*1*	11		*Beryciformes 264*
			Rondeletia loricata	*1*	2		
Berycodidei 41.5			*Beryx splendens*	*1*	39		
		Holocentriformes	*Neoniphon sammara*	*1*	8		
			Holocentrus rufus	*2*	11		
			Myripristis jacobus	*1*	5		
		Beryciformes	*Monocentris japonica*	*1*	6		
		Lampridiformes	*Lampris guttauts*	*1*	3		Lampriformes 24
			Regalecus glesne	*1*	4		
Gadiformes excluding Bregmacerotidae 19.8	Gadiformes	Gadinae	*Gadus morhua*	*7*	80	Gadinae 25	Gadiformes 610
			Theragra chalcogramma	*10*	31		
			Boreogadus saida	*5*	41		
			Arctogadus glacialis	*4*	39		
			Merlangius merlangus	*4*	36		
			Melanogrammus aeglefinus	*3*	32		
			Pollachius virens	*4*	18		
			Gadiculus argenteus	*0.3*	16		
			Trisopterus minutus	*1*	5		
		Lotinae	*Brosme brosme*	*1*	27	*Lotinae 5*	
			Molva molva	*2*	12		
			Lota lota	*2*	24		
		Phycinae	*Phycis phycis*	*1*	8	Phycidae 25 (Phycinae 13)	
			Phycis blennoides	*4*	10		
Gadiformes excluding Bregmacerotidae and Gadidae 57.5		Macrourinae	*Malacocephalus occidentalis*	*1*	14	Macrourinae 320	
			Macrourus berglax	*3*	42		
		Bathygadinae	*Bathygadus melanobranchus*	*1*	79	Bathygadinae 22	
		Moridae	*Laemonema laureysi*	*2*	17	Moridae 105	
			Mora moro	*1*	11		
		Trachyrincinae	*Trachyrincus murrayi*	*1*	70	Trachyrincinae 6	
			Trachyrincus scabrus	*1*	89		
		Muraenolepididae	*Muraenolepis marmoratus*	*1*	104	Muraenolepididae 4	
		Melanonidae	*Melanonus zugmayeri*	*1*	55	Melanonidae 2	
		Merlucciidae	*Merluccius capensis*	*1*	73	Merluccidae 22	
			Merluccius merluccius	*2*	98		
			Merluccius polli	*2*	94		
		Bregmacerotidae	*Bregmaceros cantori*	*3*	0.2	Bregmacerotidae 15	
		Stylephoriformes	*Stylephorus chordatus*	*2*	10		Stylephoriformes 1
		Zeiformes	*Cyttopsis roseus*	*1*	5		Zeiformes 33
			Zeus faber	*2*	3		
Percopsiformes and Polymixiformes 22.1		Percopsiformes	*Typhlichthys subterraneus*	*1*	34		Percopsiformes 9
			Percopsis transmontana	*1*	13		
		Polymixiformes	*Polymixia japonica*	*1*	20		Polymixiformes 10
		Myctophiformes	*Benthosema glaciale*	*3*	0.1		Myctophiformes 258
		Ateleopodiformes	*Guentherus altivela*	*2*	7		Ateleopodiformes 13
		Aulopiformes	*Parasudis fraserbrunneri*	*2*	3		Aulopiformes 267
		Stomiatiformes	*Borostomias antarcticus*	*2*	17		Stomiiformes 426
		Osmeriformes	*Osmerus eperlanus*	*4*	3		Osmeriformes 41

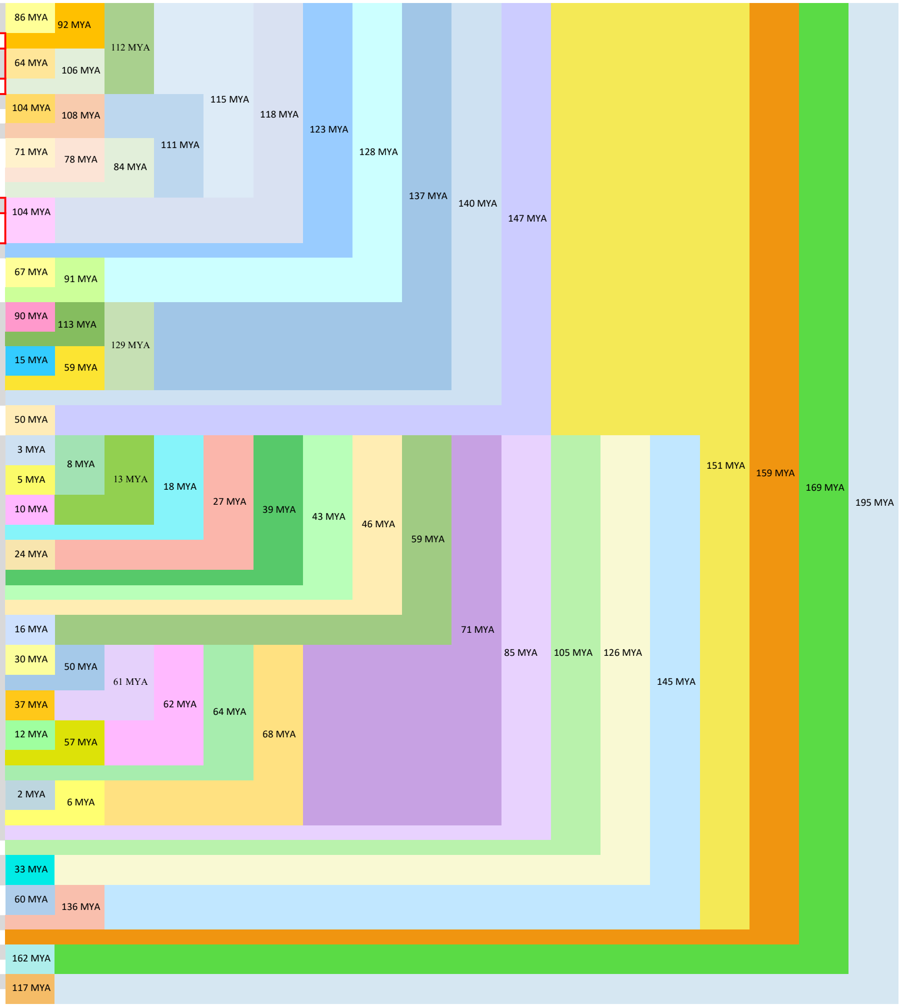

How this Table is organized:

In the columns at the far left, the dark gray shading with white font highlights the clades for which Malmstrøm et al.1 concluded elevated copy number optima, with the numbers indicating the optimum U+Z a3 values as listed in Malmstrøm et al.1 Fig. 3.

In the second set of columns, with the title "Malmstrøm et al.1 Fig. 1 clades", it shows the lowest level of clades, and also the Gadiformes clade, distinguished by Malmstrøm et al.1 in their Fig. 1. White or gray coloring of blocks only serves easier visibility. Beryciformes and Lotinae may not form proper phylogenetic clades.

The colum "Species investigated by Malmstrøm et al.1" lists the 66 species that Malmstrøm et al.1 investigated.

At the far right, the colored fields, which are not in proper ratios, represent the phylogeny and time scale (MYA=million years ago) which Malmstrøm et al.1 assigned to the separations of the investigated clades and species in their Fig. 1 and Supplementary Table 5.

The columns "estimated1 Z-a3 copies" and "estimated1 U-a3 copies" indicates the number of Z and U lineage a3 copies per species as listed in Malmstrøm et al.1 Supplementary Table 3.

The column "Gadiform species numbers according to Nelson 2006[2]" indicates the number of species per gadiform clade as indicated in the book by Nelson 2006[2].

The column "Species numbers according to Davis *et al.* 2016[3] (or Nelson 2006[2], or Wikipedia)" indicates the number of species per clade following Davis et al. 2016[3], or, if Davis *et al.* 2016[3] did not provide the information, by Nelson 2006[2] or Wikipedia (Wiki).

Red boxes highlight examples of situations which do not agree with the concept that abundant species are associated with high estimated numbers of U-α3 copies; the Z copy numbers are not included, since their limited number probably does not make a real difference, and conceptually the combination of U and Z is wrong (see main text).

What this table intends to show:
The choice of clades selected in the OU model by Malmstrøm et al.1 as regimes with elevated copy number optima seems quite arbitraily, and many other branchings do not agree with the concept that abundant species are associated with high estimated numbers of U+Z a3 copies.
The red boxes highlight that:
Blenniformes have many species, but *Parablennius parvicorni* s has only an estimated 8 U-α3 copies.
There are many more species in Carangiformes than in its sisterclade Anabantiformes, but in Anabantiformes the estimated U-α3 copy numbers are much higher than in Canabantiformes.
There are many more species in Gobiformes than in its sisterclade Scombriformes plus Syngnathiformes, but in the latter the estimated U-α3 copy numbers are much higher.
The very high U-α3 number in several Gadiformes, like for example in *Gadus morhua* , does not agree with the species-richness of Gadiformes, which with only 610 species is only modest for a >86 million year old teleost lineage.
Brosme brosme belongs to a very species-poor lineage, but nevertheless has an estimated 27 U-α3 copies.
There are many more species in Macrourinae than in its sisterclade Bathygadinae, but in the latter the estimated U-α3 copy numbers are much higher.
There are many more species in Macrourinae plus Bathygadinae plus Moridae than in the sister clade Trachyrincinae plus Muraenolepididae, but, on average, in the latter the estimated U-α3 copy numbers are much higher.
For representing the offspring of a 55 million year old lineage, Melanonidae has very few species, but nevertheless has a very high estimated U-α3 copy number.
For representing the offspring of a 138 million year old lineage, Percopsiformes has very few species, but nevertheless *Typhlichthys subterraneus* has a very high estimated U-α3 copy number.

References used in this supplementary table
1) Malmstrøm M, Matschiner M, Tørresen OK, Star B, Snipen LG, Hansen TF, Baalsrud HT, Nederbragt AJ, Hanel R, Salzburger W, Stenseth NC, Jakobsen KS, Jentoft S: Evolution of the immune system influences speciation rates in teleost fishes. *Nat Genet.* 2016; 48(10):1204-10.
2) Nelson JS: Fishes of the World. Fourth Edition. John Wiley & Sons, Inc., Hoboken, New Jersey.
3) Davis MP, Sparks JS, Smith WL: Repeated and Widespread Evolution of Bioluminescence in Marine Fishes. *PLoS One.* 2016; 11(6):e0155154.
