# Supplementary material for: Major histocompatibility complex (MHC) fragment numbers alone – in Atlantic cod and in general - do not represent functional variability
Source: F1000Res. 2018 Sep 6;7:963. Originally published 2018 Jun 28. [Version 2] doi: 10.12688/f1000research.15386.2 (PMC6081975; doi:10.12688/f1000research.15386.2)
Supplement: Supplementary file 3 [file f1000research-7-17714-s0001.tgz › 81c8742c-20c9-453f-b6fa-c469a5a67477.docx]

**Supplementary File 1:**

List of sequence reads in SRA datasets of Gadiformes published by Malmstrøm *et al.* that match with MHC class II system genes.

**Table of Contents Page**

1. Summary/Discussion 2

2. References used in this supplementary file 3

3. Read sequences 4

3.1 *MHC IIA* 4

3.2 *MHC IIB* 9

3.3 *CD4-2* 17

3.4 *CD74a* 18

3.5 *CD74b* 19

**1. Summary/Discussion**

This is a list of the MHC class II system gene matching nucleotide reads in FASTA format, except for those of *CD4-1*, which we found for gadiform fishes in the SRA datasets published by Malmstrøm *et al.*^1^. The list includes the SRA reads referred to in Table S1, plus all the additional ones that we found. Also the reads from the opposite ends of the same clones (paired reads) are included. Presented sequence orientations match the gene directions. In this list the MHC IIA and MHC IIB matching reads are not further separated into classical or nonclassical subcategories. Given that the reads may be contaminations, we feel that a detailed discussion of the reads in this article would probably be an overdiscussion. However, interested readers can contact us for such evidence, which includes facts like both read ends having the claimed gene features, the encoding of characteristic residues, and/or reciprocal top-matches.

*The coverage with reported reads of MHC class II system genes is much lower for the gadiform fishes than for the non-gadiform fishes.*

The coverage with the here listed reads per MHC class II system gene in gadiform fishes is generally low, with sometimes only one match per gene found, and did not result in assemblies into unitigs or scaffolds by Malmstrøm *et al.*^1^. In contrast, as shown in Supplementary File 2, for the investigated non-gadiform fishes Malmstrøm *et al.*^1^ found so many reads for the MHC class II system genes that they fulfilled their criteria for being assembled into unitigs and scaffolds. That is why we chose not to list all the individual matching SRAs for the non-gadiform fishes.

*The gadiform fish Melanonus zugmayeri should probably be investigated more intensively.*

With 60 reads (30 fragments with paired reads) matching to MHC class II system genes, the gadiform species *Melanonus zugmayeri* is most frequently represented in the here presented list. Although these reads are substantially less abundant than found for the non-gadiform fishes, we believe that they are nevertheless too many for reliably assuming absence of all (intact) MHC class II system genes. We recommend that Malmstrøm and co-workers do additional experiments for investigating whether all the MHC class II system matching reads in their *Melanonus zugmayeri* dataset do either belong to pseudogenes or are due to accidental contaminations. Such would very much help to reassure their claim that the MHC class II system genes were lost in all Gadiformes. Whether such analysis should also be done for *Merluccius merluccius* and *Merluccius capensis* is discussable.

*We do not analyze CD4-1 evolution.*

Malmstrøm and co-workers agree with us that in Gadiform fishes at least remnants of *CD4-1* gene can be found (see Star *et al.* 2013^2^ and our on-line discussion at the article site of the respective journal; see also Fig. S1). We feel it is their task and not ours to analyze the development from intact *CD4-1* in non-gadiform fish into the form(s) found in Gadiformes, because it is they who claim the functional absence of CD4-1 in Gadiformes. Furthermore, a proper discussion would need full-length information of the *CD4-1* gene region (which is not available for all investigated fishes, for examples see Supplementary File 2) and analysis at the transcription level. Therefore, this supplementary file does not contain information on the *CD4-1* sequences found for Gadiformes.

*Organization of the list.*

The reads are listed per matching gene, and within these gene sections organized based on species phylogeny in the same order as is shown in Table S1 (for the species phylogeny see Malmstrøm *et al.*^1^). The species name is followed by the read name given in the SRA database (<https://www.ncbi.nlm.nih.gov/sra>) and, if applicable, the mentioning that in comparison to the read report the complementary strand is shown (com) and/or mentioning of the region of the molecule that is encoded. The encoded regions are distinguished as being part of MHC IIA α1 domain (alpha-1), MHC IIA α2 domain (alpha-2), MHC IIB β1 domain (beta-1), MHC IIB β2 domain (beta-2), MHC IIB transmembrane domain (TM), CD4-2 Ig-like domain 1 (Ig1), CD4-2 Ig-like domain 2 (Ig2), CD4-2 cytoplasmic tail (CY), CD74a or CD74b single-exon encoded region with transmembrane and amino-terminal part of CLIP regions (TM Nterm-CLIP), and CD74a or CD74b thyroglobulin type-1 domain (Thy).

NCBI database accessions of the datasets that include the here listed reads are:

*Merluccius polli*: ERX1544994; *Merluccius merluccius*: ERX1544995, ERX1544996; *Merluccius capensis*: ERX1544997; *Melanonus zugmayeri*: ERX1544998, ERX1544999; *Muraenolepsis marmoratus*: ERX1545000; *Trachyrincus scabrus*: ERX1545001, ERX1545002; Trachyrincus murrayi: ERX1545003, ERX1545004, ERX1545005, ERX1545006; *Laemonema laureysi*: ERX1545009; *Bathygadus melanobranchus*: ERX1545010; *Malacocephalus occidentalis*: ERX1545013; *Phycis blennoides*: ERX1545014, ERX1545015, ERX1545016, ERX1545017; *Phycis phycis*: ERX1545018; *Lota lota*: ERX1545019, ERX1545020; *Theragra chalcogramma*: ERX1545033.

**2. References used in this supplementary file**

1) Malmstrøm, M., Matschiner, M., Tørresen, O.K., Star, B., Snipen, L.G., Hansen, T.F., Baalsrud, H.T., Nederbragt, A.J., Hanel, R., Salzburger, W., Stenseth, N.C., Jakobsen, K.S. & Jentoft, S. Evolution of the immune system influences speciation rates in teleost fishes. *Nat. Genet.* **48**, 1204-1210 (2016).

2) Star, B., Nederbragt, A.J., Jentoft, S., Grimholt, U., Malmstrøm, M., Gregers, T.F., Rounge, T.B., Paulsen, J., Solbakken, M.H., Sharma, A., Wetten, O.F., Lanzén, A., Winer, R., Knight, J.,Vogel, J.H., Aken, B., Andersen, O., Lagesen, K., Tooming-Klunderud, A., Edvardsen, R.B., Tina, K.G., Espelund, M., Nepal, C., Previti, C., Karlsen, B.O., Moum, T., Skage, M., Berg, P.R., Gjøen, T., Kuhl, H., Thorsen, J., Malde, K., Reinhardt, R., Du, L., Johansen, S.D., Searle, S., Lien, S., Nilsen, F., Jonassen, I., Omholt, S.W., Stenseth, N.C., Jakobsen, K.S. The genome sequence of Atlantic cod reveals a unique immune system. *Nature* **477**, 207-210 (2011).

**3. Read sequences**

**3.1 MHC IIA**

>Merluccius_merluccius_ERR1473848.13341992.1; alpha-2

GTAAAACACATTTATTTTAATTAGGCCTATTTTTAACATATTTCAATGTAACACAACTGA

TATTTTTAATGCTCAAGACCCCCCTGAGAGCATCTTGTATCCAACTGATGAAGTCGAGAC

GGGGGTAGAAAACCATCTCATCTGTTTTGT

>Merluccius_merluccius_ERR1473848.13341992.2 com; alpha-2

AAATTACCCGGCACCAAAAACAATCGTCCAGTTTCTGATGCTGCATCTACCAGTCCATAT

ATGCCCAATAATGACCAGACCTTCCATTGCTTCTCCACTCTCACGATCACGCCGGAGCAA

GGCGATGTGTACAGCTGCACAGTGGAGCAC

>Merluccius_merluccius_ERR1473848.13570169.1; alpha-2

CTTTCCTCAGAGCGACGGCACCTTCACCCAGTTCTCCACCATCGAGGTACTCCAGCCTGA

GGAGGGCCAGGGGTACAGCTGCACCCTGGAGCACCCGGCCCTGAAGGAGCCGGCCACCCG

CCTCTGGAGTGAGTACCTACAACTATAACA

>Merluccius_merluccius_ERR1473848.13570169.2 com

CGACGTGCGCTGACTACCTCGCCGCGGTGGGGAGAGACACTCAACACCCACCACGAACAC

CCCCGCCTGGGGATGGATACACTATAACTATAGCGAACATTAACTACCCCGCCTCTGGAG

TGAGTACCTATAACTATAACTGACTAACCC

>Merluccius_merluccius_ERR1473848.23411049.1 com; alpha-2

AACCGTTGAAATAAAATGGACCAAAAACAGAGAAGTGGTCGCCTCAGACAATCCTTTTGA

AAAGATGATCCTTAACTCCGACGGGACGTTTCACGTTTTCTCCATAATGAGTTTTGTTCC

TAAAGGAGGAGATGTTTACAGTTGCATTGT

>Merluccius_merluccius_ERR1473848.23411049.2; alpha-2

CTTCCAACTTTGGGAATGGCTGTCATTTGAAGTAAATCCCTCTCATTGAGTTTATTGTAA

AATCCTTGTGAACTTGCAGAGCCCCCTGAAGTCCTCATCTACACAAGAGATGATGTTATT

GAGCAGATTGACAACAGCCTCATCTGCTTA

>Merluccius_merluccius_ERR1473848.39302659.1 com; alpha-1

TGTTTTGACACAGGTGACACGCAAATCACTGCTACATTTGATGATGATGAAATTTTGTAT

ATAGACTTCAAAAATGAAAGTGTAGTTTACGACAGCAAAATACCTGTAGTCTACAGAATT

TTCCCATATGACTTAGCAATTTATGCNAAA

>Merluccius_merluccius_ERR1473848.39302659.2

TCATAACACGTAGTCGCTTCCTGGTGTGCAAATATGAGGAAGCCTGTACTAATACTGAAA

TGATATTTGTTTTTGTCCCTATTGAACACGATGACTGCATTCAGCCTGATTACATTTGTT

TGCTTCTCTATTTTCACCTCTGCACATGGT

>Merluccius_merluccius_ERR1473849.5835076.1; alpha-1

GCACATGGTATGGATACATATTATTTCTCTATCTGAAACAGTTAAAACTTGTTTCTTTTC

ATTGCAGGTTTTCATCAACTTTGTCACAGTGTTGCTTGTTTTGACACAGGTGACACGCAA

ATCACTGCTACATTTGATGATGATGAAATT

>Merluccius_merluccius_ERR1473849.5835076.2 com; alpha-1

CAACTTTGTCACAGTGTTGCTTGTTTTGACACAGGTGACACGCAAATCACTGCTACATTT

GATGATGATGAAATTTTGTATATAGACTTCAAAAATGAAAGTGTAGTTTACGACAGCAAA

ATGCCTGTAGTCTACAGAATTTTCCCATAT

>Merluccius_merluccius_ERR1473849.12616696.1 com; alpha-2

AGAATGAAGTGAAAATAAATGTTAAATTGCAGAGCCCCCGGTAAGTGCCATGTATGTCCA

TGACGAGGTGCGTTTGGGTTCTGAGAACACCCTCATCTGCTACGTCACTGGGTTCTACCC

TCCTCGACTCACCGTGAAGTGGACCAGGAA

>Merluccius_merluccius_ERR1473849.12616696.2

AAGTGCAGCTTTTTAAAAATACTTTTTTACTTTGAATTGAAAAGTTTGTACACTGCAGTG

AGTATCATTAACTTTTGACATGTTTCTTATGCCGATTGATATTGTATTTTTAATAATTTT

TTAACAAAGCCAAAGTGAATGCTAAATTGT

>Merluccius_merluccius_ERR1473849.16460326.1 com

TCTTGAGAAAACTATAGGTATGTATCAATGCTTTACTCTTTGTTTGTTTAAGGTTGTGGG

GAATGTTTGTGTTGTTTTTATGTCGGCCTATTACAATAGTTTTCTTAAGGCTTTTGACCA

AATATAACAAGGAACATCTCAATCCAACAA

>Merluccius_merluccius_ERR1473849.16460326.2; alpha-1

TCATTGCAGGTTTTCATCAACTTTGTCACAGTGTTGCTTGTTTTGACACAGGTGACACGC

AAATCACTGCTACATTTGATGATGATGAAATTTTGTATATAGACTTCAAAAATGAAAGTG

TAGTTTACGACAGCAAAATGCCTGTAGTCT

>Merluccius_capensis_ERR1473850.4305135.1 com; alpha-2

CTTCAACCAGTTCTCCAGACTGGAGTTCACCCCACAGCTGGGAGACGTCTACAGCTGTAC

AGTGAGCCATCTGGCCCTGAAGCAACCGCTGACCAGGATCTGGGGTGAGACACCTTATAA

ACCTCTACAAACCCACAACCAGCTCTTTAC

>Merluccius_capensis_ERR1473850.4305135.2; alpha-2

GTCATGTGACTGGATTCTATCCTGCTCCTGTAGAGTTCTACTGGACAAAGAACGAAGAGA

ACGTGACCGAAGGAACCAGCTTCAATGTTCCCTACGTCAACAACGACGGGCCCTTCAACC

AGTTCTCCAGACTGGAGTTCACCCCACAGC

>Merluccius_capensis_ERR1473850.5011156.1 com; alpha-1

GCTACCCGGAGGTATTATATGATAATGCTGAGGCCGATCAACAGACCTGCAGAACAAACC

TGAAGATGGATGATCATGAAGGACCTCCCACTGAACAAAGGTAACGTCTGATCTGATCCT

CATTTCAGTTAATGAACTTGTTAATATGTA

>Merluccius_capensis_ERR1473850.5011156.2; alpha-1

GTTAAATAACTACTGGCACAACTACAGACGATCAGTGAACCAGGTCTTGTCTGTCCAGGT

GTCCATGTGGACATGCAAGTCGTTGGATGTTCAGACTCTGATGGAGAGCAGGCGATCGCT

CTGGATGGAGAAGAGAAGTGGGTTGCAGAC

>Merluccius_capensis_ERR1473850.5095507.1 com; alpha-2

GTATATAATAATAATCTGAGCTGTGTTTGTGTTCAGATGCTCCCTCCAGTCCGATGATCT

ACCCCAGAGACAACGTGGAGCTGGAAGTGAAGAACATCCTGATCTGTCATGTGACTGGTT

TCTTTCCTGCTCCTGTAAAGTTCTCCTGGA

>Merluccius_capensis_ERR1473850.5095507.2

GGGGGGCCCCACCCAAACCTATTTTCCCCCTTTTTTTTTGATTTTTTTTTTTTTTGGGGG

GGGGGAGGGGGTTTTATACTCTCTTGTCGAGGAAAGACAACAGGAACGAGAAAAAAGAAC

AAACAAACACAGGACACGATTATATTTTAG

>Merluccius_capensis_ERR1473850.24337980.1; alpha-1

GGCTGTTCAGAGACTGATGGAGAGGAGTTTTATGGACTAGAAGGGGAAGTGAAGTGGTAC

GCAGACTTCAAGAACAAGAGAGGAGTCGAGCCTCAGCCCAGTTTCATAGATCATATGACC

TATCAGGATGGAGTTTATGAAAGTGCTGAG

>Merluccius_capensis_ERR1473850.24337980.2 com

CTAAAGCCTTCAAGGACTTCCCAGTGGAGCGTGGTAAGTCTTCCCAGTGTTTCACTCAGC

GTTCTGTTCTCCTGTTTACTTTGAACTCAACAGAAGGTTCTATTTGGACAAGAAGACGGG

TTCCTCTCAAAACAACAACAACAACAACAA

>Merluccius_capensis_ERR1473850.29078779.1 com; alpha-2

AGACGTCTACAGCTGTACAGTGAGCCATCTGGCCCTGAAGCAACCGCTGACCAGGATCTG

GGGTGAGACACCTTATAAACCTCTACAGACCCACAACCAGCTCTTTACCTGCTGTCTGTC

TCCAGATGTGGACCTGACTCTCAACTCTGT

>Merluccius_capensis_ERR1473850.29078779.2; alpha-2

ATGTGACTGGATTCTTTCCTGCTCCTGTAGAGTTCTACTGGACAAAGAACGAAGAGAACG

TGACCGAAGGAACCAGCATCAATGTTCACTTCGTCAACAAAGACGGGAACTTCAACCAGT

TCTCCAGACTGGACTTCACNCCACAGCTGG

>Merluccius_capensis_ERR1473850.29286756.1 com

GCTTTTCGTCCCGAGCCTCCCTTACGACAAGGTAGCGAGGCTAACCGCACGTTTGTATTA

CTATAATGGAATTATGTTATTTATTATGTCGGGAATCGATCACACCCTCAGTAAACTCCC

CATTCTGATGTATTTACTCGTTGGCCAGAC

>Merluccius_capensis_ERR1473850.29286756.2; alpha-1

TACGTCTACGGGTGCTATGAGTCCGGCGGGGTGCGGGTGGACATCGTCGTTGACGGGGAG

GTGGCGGTCTACGCTGACTTCAGCAAGGAGGAGGGGGTGATTCTGATGCCCCACATACCA

CAGGCCTTAAAAGACCTGANGGACAAAGGT

>Merluccius_capensis_ERR1473850.35893807.1 com; alpha-2

TATATGAAATATTAATAAGAGCTGTGTTTCTGTTCAGATCCTCCTTCTCGTCTAATCATC

TACGCCCGAGACGCCCTGGACCTGGGGGAGAAGAACACCCTCATCTGTCATGTGACTGAA

TTCTTTCCTGCTCCTGTAACGTTGTACTGG

>Merluccius_capensis_ERR1473850.35893807.2; alpha-2

GTTGCATTAAATTACTCGATGAATAGTTGGATAAATGAGTTTCACTGATAATAAGATATT

ATATGAAATATTAATAAGAGCTGTGTTTCTGTTCAGATCCTCCTTCTCGTCTAATCATCT

ACGCCCGAGACGCCCTCGCNCTGGGGGAGA

>Melanonus_zugmayeri_ERR1473851.1276178.1 com; alpha-2

CCGAAGGAACCAGCATCAACGTTCCCTACCCCAACAAAGACGGCACCTTCACCCAGATCT

CCAGACTGGAGTTCACCCCACAGCAGGGAGACATGTACAGCTGTAGAGTGACACATGTGT

CCCTGGAGGAACCTCTGACCAGATTCTGGG

>Melanonus_zugmayeri_ERR1473851.1276178.2; alpha-2

CATTGATGACACTAATCATAACTTATTGATATGTCCTGTGTTTGTGCACAGATCGTCCCT

CCAATCCGATGATCTACCCCAGAGACAACGTGGAGCTGGGACAGCAGAACCACCTCCTCT

GTCATGTGACTGGTTTCTATCATGCTCCTG

>Melanonus_zugmayeri_ERR1473851.14541320.1; alpha-1

AAGTGGTACGCAGACTTCATCAACAAGAGAGGAGTCGAGCCTCAGCCCAGCTTTGTAGAT

CATGTGAGCTACCAGGAAGGAACTTATGAACAAGCTGTGGCTAATCAACAGATCTGCAAA

GAAAACCTGAAAAAATCACTTAAAGCCGTC

>Melanonus_zugmayeri_ERR1473851.14541320.2 com

ATAATGTAAAATACAAGAATAGAACAGCAGTCCTGTGAACGTCTTTTGTGAATGATTTGG

ACTTTTTTATATTTAGTGTTCATTAAAGGACAGACAGACAATTTCCCCATAAACAAACAA

ACAAACAAAGAAAAGTCCTCATTGTCCACC

>Melanonus_zugmayeri_ERR1473851.15069226.1 com; alpha-2

CACAGCGACTTCACCGTCCGCTTCACGTCCTACCTGGACTTCACTCCTCGGGAGGGCGAC

ATCTACTCCTGCAGCGTGGGACACATCGGTCTGAAGGTGCCTCTCGCCAAGTTCTGGGGT

AGGTCGGGTGTAAGAGTAACAGAGGGGATC

>Melanonus_zugmayeri_ERR1473851.15069226.2; alpha-2

TCCCCAACACCCTCCTCTGCCTCGTTAACGACGTCCATCCGCCAACTCTGGACTTCGCCT

GAACCAGACACGGGCAGCTGCTGTACCGCACGTAGGACAGCCAGACTCAGCACTACTCAA

CACGCGTCTTCACCTTCCTGCACACCTCCC

>Melanonus_zugmayeri_ERR1473851.32053367.1 com; alpha-1

GCTTCGAGAATGGGACGACTGAAATACAGTATCAATTTGACTCTGAGGAGGTTCTACACG

TGGATTTTAAAAGTCAAACTGTTGTATTCAGTGTGCCCACAATTCTTACAGACCAGACAG

AAAAGCTTCTTGCTGATATGAATGTTTATA

>Melanonus_zugmayeri_ERR1473851.32053367.2; alpha-1

TTAAGTTTCTTTAGAGAATACAAACAGAAGAATGGATCAAAAAGTAGCCAGACTTTCTGT

TACTCATCTTTACAGTACATTCATAATTTTACTACATTGTTTTTCAGTTCCCCATGACAT

AACCTACATGGTTGGCTGCTTCGAGAATGG

>Melanonus_zugmayeri_ERR1473851.34596639.1; alpha-2

CAATTTCACATGGACAAAGAATGGGATGGAGGTCACAGAGGGGGTGTCGAACCTGCGTTA

CCGCCACAACAGTGACGGCACGTTCCACAGAATCTCAACTCTGAGTTTCTTTCCTCGGGA

GGGAGACGTTTACTCCAGATCGGAAGAGCA

>Melanonus_zugmayeri_ERR1473851.34596639.2 com; alpha-2

CGCTCTTCCGTGCTCAATTTCACATGGACAAAGAATGGGATGGAGGTCACAGAGGGGGTG

TCGAACCTGCGTTACCGCCACAACAGTGACGGCACGTTCCACAGAATCTCAACTCTGAGT

TTCTTTCCTCGGGAGGGAGACGTTTACTCC

>Melanonus_zugmayeri_ERR1473852.5535820.1 com; alpha-1

CTTGTCGATGACGACGTGGCCGGCTACGCTGATTTCAGTAAAAAAGAAGTAGTGTGGGTG

TTACCTCACCTGCCACCAAGTATAGCAGCCATCTCCAAAGCACAGGCTTATGAAATTGCG

AAAGCAAGCATGATTCACTGTCAGAGCGTT

>Melanonus_zugmayeri_ERR1473852.5535820.2

AAATACCTTCAGTTCAGCCTTTTGCTCCACCAACAGTGGCCCTGACCATCAGACTGTACA

ACAGCTCTCAGTCATGGCAGGAGGCACAATAGACAATAGACGATGGACAAATCTGCCCCT

CTTTGTCTTGCAGACTAGTTTGAGTCAATG

>Melanonus_zugmayeri_ERR1473852.8921481.1; alpha-2

AACATCCTCACCTGCTTTGTGAATCATTTCTACCCACCTGACATCGAAGTCAGCTGGACC

AAAAATGGTCGTCCAGTGTCAGAGGGGGTGTCACTCAGCCGATATGATCCCAATAATGAT

CAAACCTTCCACCAGTTCTCGACCCTGACA

>Melanonus_zugmayeri_ERR1473852.8921481.2 com; alpha-2

ATTCACACCAACGGAGGACGACATTTACAGCTGCACTGTGGAGCACTCAACCCTGGACAG

GCCTACAACAAGGATCTGGGGTAACTATTTTATCTTATGAAGGCACTTAAAAAAATGCAA

TGATTTGTTGTAAAGTTCTTTTTTTTTGCG

>Melanonus_zugmayeri_ERR1473852.15221495.1

TGATTCACTGTCAGAGCGTTCTGGGTAAATCAGAACGAGCGGATCCTGGTGCTGCCTTGC

GACAAGGTATGAGAAGCTGAACACAGAGGAGCATTTAGCAGCTAAAGATCTGGTAGAGAC

TATAAGATGTGCTCACCCACTTTCTTTCTA

>Melanonus_zugmayeri_ERR1473852.15221495.2 com; alpha-2

GGTGCAGAGGTCACAGACGGAGTGTCGTACCTGCGCTCCCGCCAAAACAGTGACGGGATG

TTTCACAGAATTTCAACCCTGAGTTTCACTCCACGGGTGGGGGATGTTTACTCCTGTTCA

GTGGAGCCTCAAGCCTTACAAGAGCCTCTT

>Melanonus_zugmayeri_ERR1473852.18748270.1; alpha-1

ACAGACCAGACAGAAAAGCTTCTTGCTGATATGAATGTTTATAAAAATGCTTTAAAAGCC

CAGAGGGTGTGTGCAGCATTTGGCGCGTTCTTGAAAGTAGAGAAGCAACATCCAGCAGAA

GAAAAGGGTAAGTCAACATTTAGCTCTGCA

>Melanonus_zugmayeri_ERR1473852.18748270.2 com; alpha-2

AGTTAAAAACAGACTCATCTGCTTCGTGAATGACTTCTACCCACCGGACATCAATGTCAG

CTGGACCAGAAATGATCGTCCGGTGTCGGAGGGGGTGTCACTCAGTCGGTATTATCCCAA

TGATGATCAAACCTTCCGCCAGTTTGCCTC

>Melanonus_zugmayeri_ERR1473851.28557328.1; alpha-1

AAAACTCACAGGTCAACACCTGCTTCGCTTCTTGACCTTCTGCCAAAGGGACGTGAATTT

GACAGCGATCAGCTACGTTGACCCTTCCACCTATCGGGTAGTCCAGCGTCTGCCAGAGGT

TGCAGAGGAGTGGGCTCCTCCTGATCCTCG

>Melanonus_zugmayeri_ERR1473851.28557328.2 com

GTTTGCAGAGGAGTGGCTCCCTCCTGCTCCTCGACTGGCCCAATAACACATACATACCCC

GGGGTCCCTGCAAGTACAACCTGCCATAAAAGGAGAAAACTATCCTCCAGAGGTCATAGG

TGAGGATGAGGGCCTAAGCTTCTTTAAAAA

>Muraenolepis_marmoratus_ERR1473853.13532583.1 com; alpha-1

TGTCACAATGTTTTGAGTAAAGCTAGAAGAGCAGATCCTGGTGCTACCTTACGGCAAGGT

AGAGTTTACCCAAACATTTACAGGGTTGGGAGGGTTACTTTAAAAATGTATTCCTTTACA

ATACTAGTTCTGCTTTGGACCAAAGCTTGT

>Muraenolepis_marmoratus_ERR1473853.13532583.2; alpha-1

ACTCCCTCTTCTGTGTTCCTCTCCTGCTTTTCAGCGCCCTCCCATGAATTCCACTTCATC

TACAGCTGCTTTGAGTCCCGCGATGTGCGAGTCGATGCCCTTTTTGTCGACAACGAGGCG

ATGTACGCAGATTTTAACAAGGAGGAGTCA

>Muraenolepis_marmoratus_ERR1473853.35942289.1; alpha-2

TTGAGTTTGGACGTGAAAACAGCCTCATCTGCTTTGTGAATAATTTCTACCCACCTGAGG

TCAGAGTCATGTGGACCAGGAATGGTGTTCAGGTGTCAGAGGGGGTGTCACTCAGCCGAT

ATTTCCCCAATAAAGATCAAACCTTCCACC

>Muraenolepis_marmoratus_ERR1473853.35942289.2 com; alpha-2

GGTGTTGAGGTGTCAGAGGGGGTGTCACTCAGCCGATATTTCCCCAATAAAGATCAAACC

TTCCACCAGTTTTCTACCCTGACGTTCACACCGAGGGAGGGGGACATTTACAGCTGCACT

GTGGAGCACTCAGCCCTGGACATGCCTCAA

>Phycis_blennoides_ERR1473867.1216359.1 com

AACAGAGTCCAGGTGAGGCTGATGAACGTCTTCAGTTCTGCAGGTTTCTGGTCATAAACC

AAACTATTAACAGGTTACCATGGTGACCTGATGGTGGCGCTACAGAGGAGAAGGCAGAGG

GTCATTAGAGTTCTGATGGTTCATCCTGAAG

>Trachyrincus_scabrus_ERR1473854.42984801.1; alpha-2

CGTGAACAACGACGGAACCTTCTACCAGTTCTCCAGTGTGAAGTTCACAACAGAGCAGGG

AGACATCTACAGCTGTACAGTCAACCACCAGTCACTGCTCTGTTGTGAACTTCAAGATCG

GAAGAGCACACGTCTGAACCTACAGTCACA

>Trachyrincus scabrus_ERR1473854.42984801.2 com; alpha-2

TCAACCCTCTGTCCCTACGCGGCTCTCTTCCGATCTCGTGAACAACGACGGAACCTTCTA

CCAGTTCTCCAGTGTGAAGTTCACAACAGAGCAGGGAGACATCTACAGCTGTACAGTCAA

CCACCAGTCACTGCTCTGTTGTGAACTTCA

>Trachyrincus_scabrus_ERR1473855.24170403.1; alpha-2

TTCTATCCTGCTCCAGTGAGCATCACATGGACCAGGAACAACCTCAACCTGACTGATGAC

ATCACAGCCACCGTCCCGTTCCCCAAAGGTGATGGAACCTTCTACCAGTTCTCCAGGCTG

AAGATCATCCCAGAGCAGGGAGACACCTAC

>Trachyrincus_scabrus_ERR1473855.24170403.2 com

CACAAAATGTTCGTCGACTACCTTTTTTTTACTGACGAAAACGAAATGGAAACTAAATAA

AGACTAAACTATGATGACTAAAACTAAGACTAAATATATTGACAATTTGTCAACAAATAA

AAACTAGACCAAAATGTTAGGGAGGGATTG

>Laemonema_laureysi_ERR1473862.17076666.1 com

CTAGCTAACAGTGACGGTAACGGTACGTTAGCTAGCTTAGCAGGTTTAGCAACACCCACC

TTGGGATCAGGCTCCAGGATAGCAGCTAGCGGCTGTCGTCTCCTGCTCTCAGCTGATTCT

CTCTGTCTGTCCCTCTCTTTCTCTCGTACT

>Laemonema_laureysi_ERR1473862.17076666.2; alpha-2

AGGACGGCACCTTCAACCAGTTCTCCAGGCTGACCTTCACTCCAGGAGATGGAGACATCT

ACAGCTGCACCGTGGAGCACCGAGCGCTCAGCGAACCTCTGACCAAAGTCTGGGGGGAGG

ATTCTATGTGTGTGTGTGTGTCTGTAATAT

>Laemonema_laureysi_ERR1473862.20677118.1; alpha-1

GTCCATGTGGAAGTTCATGTCATGGGATGTTCAGACTCTGATGGAGAGAAGATGGAGTCT

CTGGATGGAGAAGAGATATGGGTTGGAGACTTCGTCAAACAGAAGGCAGTGGAGGCTCAG

CTCAGTTTCATGGATCATATCAACTTCGCA

>Laemonema_laureysi_ERR1473862.20677118.2 com

TACCCATGAGCTTATGGTGCTGCTGAGGCCAATCAACAGATCTGCAGAGACAGCCTGAAG

AAGGTCCGTGTCATCATTAAGGACCCCCCTCTGAACAATGGTAAAGTCTGATCTGGTCCT

CACTTCAGTTAAGGATCTTGTTAAGATGTA

>Laemonema_laureysi_ERR1473862.20754527.1 com; alpha-2

GACCGCTGACCAAAGTCTGGGGTGAGGAGTCTATGTGTGTGTGTGTAATATCTATGTCAT

TATCTGTGTGTGTGTGTGTGTATGTGTGTCATTATCTGTGTGTGTGTATGTGTGTGTGTG

TTCAAATTCAAATTAGCTTTATTGGTAGGA

>Laemonema_laureysi_ERR1473862.20754527.2; alpha-2

CTTCTACCCAGCTCCAGTCACAGTCAGGTGGACCAAGAACAACGTCAATGTGACAGATGG

TGCAGCTACCAGCGTCCCCTATCCCAACAAGGACGGCACCTTCAGACAGTTCTCCAGGCT

GACCTTCACTCCAGGAGATGGAGACATCTA

>Laemonema_laureysi_ERR1473862.28257032.1

CAATACCCAGAATACACTCTGAAGTATTGTGTTTAAAAGCCCTGTCAGGTGTTTTATATT

ACTTCAGAATAAAACATACTAGGTGCTAATAGGAATAAAAGCCCTTGGTTAGATAAAACG

CTGTATCAGTATGAAGTTTGTTGTTCTGCA

>Laemonema_laureysi_ERR1473862.28257032.2 com; alpha-2

CACCATCTACACCAGAGACGTCGTGGAGATCGGAGGGGGGAACCACCTCATCTGCCATGT

GACCGGCTTCTATCCTGCTCCCGTCAAGGTCTACTGGACCAAGAACAACGAGAACGTGAC

CGAAGGAACCAACCTCAACACCCCGTACCC

>Laemonema_laureysi_ERR1473862.38712638.1; alpha-2

CTCATGATACGTTTTGGCCCTCGGCCCTCCATGTCTAAAGTTTTGTCCACCCCTGGTTTA

GACTCTTTTCCATACTGTGACTGTTGTGTAGATGCTCCAGAGCACTCCACCTATCCTCGC

AATGAGGTACGGCTGGGAGTGGAGGACATC

>Laemonema_laureysi_ERR1473862.38712638.2 com; alpha-2

CTGGGAGGTGAGAACATCCTCATCTGCTATGTAGATCATTTCTACCCTCCCAACATCAAA

GTCAACTGGACAAAGAATGGCCGGCTGGTGACTGAGGGAGCATCTCTCAGTCTCTACTAT

CCTAATAATGATGGGACCTTCACCAGTTCT

>Laemonema_laureysi_ERR1473862.42883890.1; alpha-1

GTCCATGTGGAAGTTCATGTCATGGGATGTTCAGACTCTGATGGAGAGGAGATGGAGTCT

CTGGATGGAGAAGAGATCTGGGTTGCAGACTTCGTCCAACAGAAGGCAGTGGAGCCTCAG

CCCAGTTTCATGGATCATATCAACTTCCCA

>Laemonema_laureysi_ERR1473862.42883890.2 com; alpha-1

TGCCCAGGAGCTTATGATGCTGCTGAGGCCAATCAACAGATCTGCAGAGACAGCCTGAAG

AAGGTCCGTGTCATCATGAAGGACCCCCCTCTGAACAATGGTAAAGTCTGATCTGGTCCT

CACTTCAGTTAATGAACTTGTTAATATGTA

>Bathygadus_melanobranchus_ERR1473863.13853066.1 com; alpha-2

ATAAAAGCCCTTGGTTAGATAAAACGCTGTATCAGTATGAAGTTTGTTGTTCTGCAGATG

CTCCTCACAGCACCATCTACACCAGAGACGTCGTGGAGATCGGAGGGGGGAACCACCTCA

TCTGCCATGTGACCGGCTTCTATCCTGCTC

>Bathygadus_melanobranchus_ERR1473863.13853066.2

AAATAAGATCATGAAAGGTTCAATTCTGTTCTAGTTAACATAAATATAGGAACAATACCC

AGAATACACTCTGAAGTATTGTGTTTAAAAGCCCTGTCAGGTGTTTTATATTCATTCAGA

ATAAAACATACTAGGTGCTAATAGGAATAA

>Bathygadus_melanobranchus_ERR1473863.33589778.1 com; alpha-1

TTGCCAACAAGAATGTTATTGTATTCTCTACTTCTTCAAATCACAGGCACACATCTAATG

CAGATACTGACTTTCTGCCATTCGAACATGACGGAGGGGGAGTATGACATGGAGTTTGAC

GGCGATGAGCTATTTTATGTGGATTCCTAG

>Bathygadus_melanobranchus_ERR1473863.33589778.2; alpha-1

GAAATTGCCAACAAGAATGTTATTGTATTCTCTACTTCTTCAAATCACAGGCACACATCT

AATGCAGATACTGACTTTCTGCCATTCGAACATGACGGAGGGGGAGTATGACATGGAGTT

TGACGGCGATGAGCTATTTTATGTGGATTC

>Phycis_blennoides_ERR1473867.1216359.2; alpha-1

TGAAGGTAAGATCCTGTCTCACATTGATAACTGACTGATCAACAGACAGTGTGTCAGTAG

CTGATGCTTCTGTTTCTCTTCAGGTCTACATCAGGACATTGCTATCAACGGCTGTTCAGA

TGTTGATGGAGAAATGATGTATGGACTGGAT

>Phycis_blennoides_ERR1473869.6893481.1; alpha-2

TGTGATTCTGTTCAGATGCTCCGTCCAGTCCGATCGTCTACACCAAAGACGACGTGGATC

TGGGACAGGAGAACATCCTCATCTGTCATGTGACTGGGTTCTTTCCGGCTCCTGTTAACG

TCTCCTGGACAAAGAACGGAGAGAAGGTCAC

>Phycis_blennoides_ERR1473869.6893481.2 com

CACCTGATGAACTGACCAAACTACAGCTGATCTGTCTCCAGATGTGGAGGTGAAGCTGCA

TGTTTCTGTCTCCAGATGTGGAGGTGAAGCTGAATTTTTCTGTCTCCAGATGTGGAGATG

AAGCTGCATGTTTCTGTCTCCAGATGTGGAG

>Phycis_phycis_ERR1473871.51739494.1 com; alpha-1

AGTTGTGGTACGCAGACNTCAACAAAGAGGAAGTAGTACTGCCTCTGCCTCCCTTTGTTG

ATCCTATGACCGTCCCAGGAGGTTATGAATCAGCTCTGGCTCAGCAACAGAACTGCAAAG

CTAACCTGAAGGTCTTTAGTGAAGCTCTGA

>Phycis_phycis_ERR1473871.51739494.2

GAGAGAGAGAGAGAGAGACAGAGAGAGACANACAGAGAGAGAGACAGAGACAGGTTTAAC

ATCAGTGTTTGTGTTTTTAGGTCATGGGGATTTTTGTTTACGCGGGTGTTTAGACTCAGA

CAGAGAGGAGCAGATATGCGTGGGAGAAGA

**3.2 MHC IIB**

>Merluccius_merluccius_ERR1473848.1435597.1; beta-2

AAACTGGCTTTACCAGAAGCACTCCTACCTGGAGTATATACCTACAGTAACAGACACCAT

CGAGTGCATGGTGGAGCACGCCAGCCTCTCAACACCTGAGCTTTACAAATGGGGTACGAT

GGTTCCCAGGTCATGATCATGTCATTCAAA

>Merluccius_merluccius_ERR1473848.1435597.2 com; TM

CAAGAAAAAACAAGATCATTGTTGGGACTGCAGGGCTGCTGCTGGGACTGGCCTTTACTG

CAGTTGGTGTTATTTACTACATGAAACACACAAGAGGTATGAATGAAGCAAATGACAAGA

GGAGCGGGACCACACCCTCAGAATAGAATA

>Merluccius_merluccius_ERR1473848.4129854.1 com

CTTTGATGTTTATGTAGTAATCATATAAACATATTTTGTTTTCCTCACTAAATACAAAAG

GTTCTATTAAGGCCATTTGAAAACCTTTTGCTTCTATAATTCCCTGCACATGGGACCCTT

GCGGAGTCAAAAGGTTCTATGAAAAAACCC

>Merluccius_merluccius_ERR1473848.4129854.2; beta-2

ACAGCTTCTATCCAAAAGACATTAAGATGACTTGGTTGCAGAATGGACAGAAGGTGACCT

CAGCTGTAACCTCCACTGAGGTCTTGTCAGAGGCAGACTGGAGCTTACAGATTCACTCGT

ACCTGGAGTACAGCCCGAAGCCCGGAGAGG

>Merluccius_merluccius_ERR1473848.9854968.1 com; TM

CGATGGTTCCCAGGTCATGATCATGTCATTCAAATGAAGTTATTTGTGATAATTTTATTA

ATATTTGTAATTGTTGCACAGAGCACTATTCAGAGTCAAGAAAAAACAAGATCATTGTTG

GGACTGCAGGGCTGCTGCTGGGACTGGCCT

>Merluccius_merluccius_ERR1473848.9854968.2; beta-2

AGTCACCAACCCGGCAGCATCATCTGCAGCGCCAACAATTTCTATCCCAAACCCATCACT

TTGACATGGCTGAGAAATGGGGAGGAGGTGACGTCTGAAGTTATTTCCACTGATGAGATG

TCCGACGGAAACACGCTTTACCAGAAGCAA

>Merluccius_merluccius_ERR1473848.18918991.1 com; beta-1

GGTAACCCGCAGCTGTGTTTTTAACTCCACTCGTCCTGAGGACATACAGTACATAGATTC

TTATTTCTACCACAAACAGGAGTACACCAGGTTCGACAGTAACGTGGGGAAGTTTGTGGG

ATACACTGAGTTTGGAGTGAGGAACGCAGA

>Merluccius_merluccius_ERR1473848.18918991.2

TATTTATCCCACACGAGGAAATGACTTCAGAGCAGAAAAACAAATGAAGAAGCGAGTGGA

GCTCAGTCATAGATGATCACAGATGATCACAGATGATCATCTGTGATCACAGATGATCAC

AGATGATCATCTGTGATCATCTTGAGCAAC

>Merluccius_merluccius_ERR1473848.23186317.1 com; TM

GATACAGATCATATGTGTTGCCATTGTATGACCTCACACATCATGCGTGCCGTGACCTTT

TCACAGACCCCTCCATGCCTGAGCCTGAGAGGAATAAGATTGCCATTGGAGCTGCAGCAC

TGGTGCTGGGGTTAATCGTCACTGCTGCTG

>Merluccius_merluccius_ERR1473848.23186317.2

GCGCACTGCAGCTAAGACACATGTGACGACATGCAGGCTACATGTAGCATTAGCTCTAGT

ATTGCACACGATATAGCTGCTCTTAGTCCTATAGTTAATGTATGCTTTTCATAGTTTTAT

TCAGGTAAGACTGCTCAAATGTAAAGTGCT

>Merluccius_merluccius_ERR1473848.25746747.1; beta-1

CTCTGTTTTCAGATGGATTTCTCTATGTGGTAACCCGCAGCTGTGTTTTTAACTCCACTC

GTCCTGAGGACATACAGTACATAGATTCTTATTTCTACCACAAACAGGAGTACACCAGGT

TCGACAGTAACGTGGGGAAGTTTGTGGGAT

>Merluccius_merluccius_ERR1473848.25746747.2 com; beta-1

CAGAGTACTGGAACAACAACCGTGCCCTCCTGGAGAGAGAGAGACTTCAGAAGGACACTT

ACTGTGCTCACAACATCCAGATCTGGAACAACAACATTCTCTCCAAATCAGGTGAGTCAC

ATGACGCCCCCTTATGGACGTGTAGTGTAA

>Merluccius_merluccius_ERR1473848.34193700.1 com

GTACACACCCAGGTACTGTCCTGTAGGTACTCTCACCTGGAGTACACACCCAGGTACTGT

CCTTTAGGTACTCTCACCTGGAGTACACGCCCAGGTACTGCCCTTTAGGCACTCTCACCT

GGAGTACACACCCAGGTACTCTCCTTTAGG

>Merluccius_merluccius_ERR1473848.34193700.2; beta-2

TTGGACCAACAACCAGCAGCAGATCAGCACCGGAGTGTCCAACTCTGAGACCATGCACGA

CTCAGACTGGTACTACCAGAGGCACTCTCACCTGGAGTACACACCCAGGTACTGTCCTTT

AGGTACTCTCACCTGGAGANCACACCCAGG

>Merluccius_merluccius_ERR1473848.40953759.1; beta-1

TTTNGTGTTTCCAGATGGGTTCCAGAATTATGTTATAGAAAACTGTGAATTTAACTCCAC

TAACCCTGATGGCATCGAGTACATTTACACTCAGATCTACAACCAGATGGAGCTCTTTAA

GTTCACCAGCAGTTTGGGGAAGTTCGTCGG

>Merluccius_merluccius_ERR1473848.40953759.2 com

TCCAGAAGTACACATACTGTGAACACAACATCAACAACTGGTACAAAGCCATGCTGTCCA

AATCAGGTGAGCGGAGCTTAGACGGTGGCGCCTCCTGGTGGATGCAACTTCACCTCTTCA

CCCCCGCCTTCACCGAGCACAGGTTCTCCA

>Merluccius_merluccius_ERR1473848.47786745.1 com; beta-1

GATGCAGTACAACAGCACAAGAGAGAGCTGGATTGGATTTACTGAGTATGCAACAAATAT

GGCACAACATTTTAACGCATATCCAAAAGGTTTGAGCCAAAGAAAAATTGAAGAAAGTTT

TTTCACTGAGTGCATGATATTTGCAATTAA

>Merluccius_merluccius_ERR1473848.47786745.2

TGTATTTTGTTTTCTGTCACCAGTGGTTTCTTCACTGCATCAATGTAAGTAATGGTATAT

TTTTAACTATCAGACAGGACTATATGATTACTAATAAAGTATGAATGTTACAGATAGCAT

CTTATTATAACACCATATATGCTTCTCTGT

>Merluccius_merluccius_ERR1473848.48940243.1 com

GATATTTGCAATTAATGTAACAAGTAAGTACCATGATGACTGCAGATACTGGTCACCTAA

CTCTGAAACTAAATACAACACATCTATCTTTTTTTCTAGAGAACATTACAACAGAACCAG

TTCTGAAAATGACTTCAGTGAAGCAATCTA

>Merluccius_merluccius_ERR1473848.48940243.2; beta-1

ATTATGAAGTAAGATTTCTAATAAACAAAGAGTTGTGGATGCAGTACAACAGCACAAGAG

AGAGCTGGATTGGATTTACTGAGTATGCAACAAATATGGCACAACATTTTAACGCATATC

CAAAAGGTTTGAGCCAAAGAAAAATTGAAA

>Merluccius_merluccius_ERR1473848.50482417.1 com; beta-2

GAAGCAATCTATTGGAACAAAGCCTGCAATACTGTTATGTAGTGCTTACAGCTTCTATCC

AAAAGACATTAAGATGACTTGGTTGCAGAATGGACAGAAGGTGACCTCAGCTGTAACCTC

CACTGAGGTCTTGTCAGAGGCAGACTGGAG

>Merluccius_merluccius_ERR1473848.50482417.2

GAAAGTTTTTTCACTGAGCGCACGAGATTTGCAATTCATGTAACAAGTAAGTACCGGGCT

GACTGCAGCTAGTCGTCACGTAACTCTGCCAGTAACTCGCACAGATCTATCTTTTTTTCT

AGAGAACATTACAACAGCACCAGTTCTGAC

>Merluccius_merluccius_ERR1473849.19921760.1 com; beta-2

CCTCTGGTGGCAGTGAGAGGGTACTGGTCTGCAGCGTCTACGGCTTCTTCCCCCAACACC

TCACTGTGACCTGGACCAACAACCAGCAGCAGATCAGCACCGGAGTCTCCAACTCTGAGA

CCATGTCTGACCAGGACTGGACCTACCAGA

>Merluccius_merluccius_ERR1473849.19921760.2

GTTCATAACGTTGGGATCTGGAACAGCAACATCCTCTCCAAATCAGGTGAGTCACATGAC

CTTCTTGTATGGTTAACTTCATCTTGGAGTCTTCTATGGAATTGGAAAGTGTCAGATTAT

AGTTTCATAATTACTGAAGTGAATTTGATC

>Merluccius_merluccius_ERR1473849.20183195.1

CAGCTCCTGTTGTGTTCAGAGACCATGGAGGTCATTAAAGACTTAAGTGAGTCATCTGAT

GCAAATCATCATGTACCATATAATTCTGTTCACATCACCTGCACAGTGACATAAATGTTT

TTTTCAGGGAGCATGTTGGCAGCCCCAAGT

>Merluccius_merluccius_ERR1473849.20183195.2 com; beta-2

TTACAAAATGGACAGGAGGTGGCCTCAGGCGTAAGCTCCACTGAGGTCTTGTCTGATGGA

GACTTGTACTACCAGATTCATTCATATCTGGAGTACAGCCCAAAGCCCGGAGAAGAAATC

TCCTGCATGGTGGAGCATGTGTCCCTGTCA

>Merluccius_capensis_ERR1473850.3437343.1

AAGCCAAGTTTCCGCATGAACATACCTTAAATACTAACACACGTACTGCATCTGTACATT

TCAAAGTAAAACGCGTGGCTTCTCCTTCACCCTTCAGATTGTGCCGCTGATGGACGCGGA

TACTTCATGTATGCCGACTTCTGGTGTGCC

>Merluccius_capensis_ERR1473850.3437343.2 com; beta-1

CGCGTATGCTCATTGAATCCCGTCTTCTGTTGTCCGTGCCCCACCGGGGACCCGAAGCGG

GTGAGTATCTGGTGTACTGGTATTTCAACGGGGAGTTCATGATGCAGTACAACAGCACGT

TGGGCAACTGGACGGGCCTCACTCCGGCTG

>Merluccius_capensis_ERR1473850.4866249.1 com; TM

AAACAAGATTGCCATCGGAGCCTCAGGACTGATCCTGGGTCGGACCTTGCCTCTGGCTGG

ATTCATCTACCACAAGAGGAAGGGCAGAGGTCAGAACAGGACACTCTTTAGTTTAGTGTT

GGTTTGGATTCAGACCCCTTTTTTCCAGAC

>Merluccius_capensis_ERR1473850.4866249.2

GGCTTCACCCCCTTTCAATGTGTTTTTGGTTACGAGCCTCCTGTCTTTGCAGACAATGAG

CCAGAGGTATCGGTCCCCTCAGCCCACGCCTTGATGTGTGTGTGTGTGTGTGTGTGTGTG

CGCGTCCGTGTCCGAGCCATGCCCGAGTCG

>Merluccius_capensis_ERR1473850.7430792.1 com; beta-1

GCCATGAACGCTCAGAAGGAGACCTACTGTCTGTTCAACGTTGTCATTGACTACAATGAT

GCTCTGACTAAAACAGGTGAGTTTGTTTTCATCACCTCGTCATTTATTCATCATCAACAG

CTGAAAGAACCAGAGATTCAACTTTAATCT

>Merluccius_capensis_ERR1473850.7430792.2; beta-1

CTGAAGGACATCGAGTACATCAGGTCGTACTATTACAACAAGATTAAGTACACCAGTTTC

AGCAGCAGTGTGGGGAAGTATGTTGGATTCACTGAGTACGGAGTGAAGAACGCAGAGGCC

TGGAAAAAAGATGCTTATGAACTGGCTGCC

>Merluccius_capensis_ERR1473850.13196465.1; beta-1

TACTGATCTGACGGACATGGAGTACATTGAGTCGCACTATTACAACAAGATTAAGATCAT

CAGTTTCCGCAGCAGTGTGGGGAAGTTTGTTGGATTCACTGAGCACGGAGTGAAGACCGC

AGAGTCCTGGAACAAAGATGCTTCATTACT

>Merluccius_capensis_ERR1473850.13196465.2 com

AACGTGGGCAATGACTACAATCATGCTCTGACTAAAACAGGTGAGTTTGTTTTCATCACC

TCGTCATTTATTCATCATCAACAGCTGAAAGAACCAGAGATTCAACTTTAATCTGGAGCC

TCAAGTCTCCACCTGCTGGTGGAACTCTGC

>Merluccius_capensis_ERR1473850.19955592.1 com; beta-1

AGAGTTCTGGAACAAAGATGCTTCATATCTGGCTGCCATGAGGGCTCAGAAGGAGACCTA

CTGTCTGAGGAACGTTGGCAATGACTACGATGCTGCTCTGACTAAATCAGGTGAGTTTGT

TTTCATCACGTCGTCATTTATTAATCATCA

>Merluccius_capensis_ERR1473850.19955592.2; beta-1

CTGATCAATACCCTGATTCTTTCCTACAGATGGATACATGGAATATGTAGTGTCCAGCTG

TGAATTCAACTCTACTGATCTGAAGGACATCGAGTACATCGACTCGTACTATTACAACAA

GATAAAGTTCACCAGTTTCAGCAGCAGTGG

>Merluccius_capensis_ERR1473850.30820683.1 com; beta-1

CTGGAGTTCATCAGGTTTGACAGCAGAGTGGGGAAGTTCGTAGGATACACTGACTTTGGA

GTGAAGAACGCTGAACGCCTCAACACAGGTCCAGAGGTGGTGAGGATGAAAGCTCAGAGG

GAGAGATACTGTCTGCACAACGTTGGCGTT

>Merluccius_capensis_ERR1473850.30820683.2; beta-1

GATAATTCTCTGACTCTGCTCCACAGGTGGATTTCTGTGGTATAACGTGCCTCGTTGTGA

CTTTAACTCCACTGATCTGAGCGGCATCACCTACATCGAATCGTTCTACTACAACAAACT

CGAGTTCATCAGGTTTGACNGCAGAGTGGG

>Merluccius_capensis_ERR1473850.33212699.1

CTCTGATCTATCAACACATCTAGACACTATACTGATCAATACTCTGATCTATCAACACAT

CTAGACACTATACTGATCAATACTCTGACTGATAGACTTATCAGTGTATAGATCAGTGAT

CAATGGTCAGTACTGGATTGTCAGTTGATC

>Merluccius_capensis_ERR1473850.33212699.2 com; beta-1

ATCGCCTACANCTACTCCATGTGCTACAACAAACTGGAGTACCTCAGGTTTGACAGCAGA

GTGGACAAGTTCGTAGGATACACTGAGTACGGAGTGAAGAACGCTGAACGCTACAACAAA

GGTCCACAGGTGGTGAGGAGGAGAGCTGAG

>Merluccius_capensis_ERR1473850.36098825.1 com; beta-2

CCAGTTTTGTTGTTGTTCTCTCCCCAAGTGGAGCCCTCCGTAAGGCTGAGGTCAGTTGAA

GCAGCAGACAGCAGACATCCACACATGCTCGTCTGTGGTGTATACGGCTTTTATCCCAAG

CAAATCCGAGTGACTTGGCTGAGGAACGGA

>Merluccius_capensis_ERR1473850.36098825.2

ACTGTAATTTGTTGGCTATTACCGAAAGTATTACCAAAACGTTCAGCATTATTGGTACAT

AATAAGTGAAAGTTATACTATTTCATGCCAGTTTTGTTTTTGTTCTCTCCCCAAGTGGAG

CCCTCCGTAAGGCTGAGGCNAGTTGAAGCA

>Merluccius_capensis_ERR1473850.48098615.1; beta-1

GAAGTTTGTTGGATTCACTGAGTACGGAGTGAAGAACGCAGATAACTGGAACAATAATCC

TTCATATCTGGCTGACGCGAAGGCTAAGAAGGAGACCTACTGTCTGAACAACGTTGGCAA

TGACTACGATGCTGTTCTGACTAAATCAGG

>Merluccius_capensis_ERR1473850.48098615.2 com

AAAGCAGGTGAGTTTGTTTTCATCACGTCGTCATTTATTAATCATCAACATGCTGAAAGA

ACCAGCGACTGATTCAACTTTAATCTGGAGCCTCAAGTCTCTGGTTCCTCCTGCTGGTGG

GACTCTGTTACTACAACACATATACATATA

>Merluccius_capensis_ERR1473850.50479741.1

CACCTGATCACTGATCAATACCTGATCACTGATCAACACCTGATCACTGATCAATACCCG

CTCACTGATCAATACCTGATCACTGATCTTTACCCTGATCACTGATCAATACCTGATCAC

TGATCTTTACCCTGATCACTGATTTATAT

>Merluccius_capensis_ERR1473850.50479741.2 com; beta-1

CTGATCAATACTCTGATCTCTGGTTCAAACTGACTGTGTTTTCCTACAGATGGGTACATG

GAATGTAGAGTGACCGGCTGTGAATTCAACTCTACTGATCTGAAGGACATCGAGTACATC

GACTCGTACTATTACAACAAGATTAAGTAC

>Melanonus_zugmayeri_ERR1473851.25827682.1; beta-1

TCAATACGCTGATCAATACACTGATCAATACACTGATCATATTGTTAATAGTCTCTGATG

TTTTTTTTCTCTTCAGATGGATTCAGGGAATCTGCTGTGGAACGTTGTGTTTTTAACTCG

ACTGATCTGAAGAACATCGAGCTCATCGTG

>Melanonus_zugmayeri_ERR1473851.25827682.2 com

ACGATAATTCTTCATATCTGGCTCAGATGAGAGGTGAGAAGGAGAGATACTGTCAACACA

ACATTGAGATCCGATACCGGACTGTTCTGGATAAGTCAGGTGAGTTTGTGTCTGTGTGAC

ATCATCAACACCTGATCATCATCACATTAA

>Melanonus_zugmayeri_ERR1473851.33898145.1 com; beta-2

CTGAGACGCCCCCTGCTGGTCAACATCCGGGCATGTTGGTCTGCAGCGTCTTCGACTTCT

ACCCCAGACAGATCAGAGTGAGCTGGCGCAGAGACGGACAGGAAGTCACCTCTGATGTCA

CTTCCACTGATGAGCTGGCAGACGGTGATT

>Melanonus_zugmayeri_ERR1473851.33898145.2; beta-2

CCTATGTCAGACTTCACACTGAGACGCCCCCTGCTGGTCAACATCCGGGCATGTTGGTCT

GCAGCGTCTTCGACTTCTACCCCAGACAGATCAGAGTGAGCTGGCGCAGAGACGGACAGG

AAGTCAACTCTGATGTCACTTCCACTGATG

>Melanonus_zugmayeri_ERR1473851.36199669.1

TGCCAACCTCACTGTCACATCAGCCCCTTAGAGAGCTGCACATACTCTCCATTCGCCATG

TTCCTACCCACATGACTGAAACTTCTATGTGAGGTCACTCAAGGTCTTGGGCCACTTGTC

AACATTTCCTCAATTGGCTACATTCATTAT

>Melanonus_zugmayeri_ERR1473851.36199669.2 com; beta-2

AAACTTCCTCNGATTTCAGCTGAGCCCAGCGTCACTCTCGTGGAGTCCACCAGCTCCGGT

AAAAGCACCATGCTAGTGTGCAGCGCCTACGACTTTTACCCCCATAACATCAGGCTGACG

TGGCTCAGGATCGGGCAGGAAGTGACCTCG

>Melanonus_zugmayeri_ERR1473851.39074752.1; beta-2

TATTTAAACAATCTTTGTGTGTGAGTTAAACCGTACGTCAGGCTTCACTCTGTGACGCCC

CCTGGTGGTCAACATCCCTCCATGTTGGTCTGCAGCGTCTTCGACTTCTACCCCCAACAC

ATCAAAGTGAGCTGGCTCAGAGACGGACAG

>Melanonus_zugmayeri_ERR1473851.39074752.2 com; beta-2

CCGGATGTCGAGTCCACTGATGAGCTGGCAGATGCTGATTGGTACTACCAGATCCACTCT

CACCTGGACTACACGCCCAGGTCAGGTCCAGGTTAGGTCAGGTCCAGGTTTTGGTTCAGT

TTCTGGTTCAGTCAGAACGCTGATGTGTTG

>Melanonus_zugmayeri_ERR1473851.39161546.1 com

CACCTGTTTGTCTGTCTCGCGCCTGTCTGTCTCTCACCTGTCTGTCTGCCTGTCTGCTCA

CCTGTCTGTCTCTCACCTGTCTGTCTCTCACCTGTCTGTCTGTCTGCTCACCTGTCTGTC

TCTCACCGGTCTGTCTGTCTGCTCACCTGT

>Melanonus_zugmayeri_ERR1473851.39161546.2; beta-2

GGTTCTGGTTCAGTCAGAACTCTGATGTGTTGGTTTGTGTTGGTTCAGGTCTGGAGAGAA

GATCTCCTGTGTGGTGGAGCACGCCAGCCTGAAAGAACCTCTGATTACTGACTGGGGTAA

ATACCTGTCTGTCTCTCACGTGTCTGTCTG

>Melanonus_zugmayeri_ERR1473851.43976819.1; TM

TGTCTGCTCACATGTCTGTCTCTCACCTGTCTGTCCTCAGACCCGTCCATGCCTGAGTCT

GAGAGAAACAAGATCGCCATCGGAGCCTCAGGACTGATACTGGGTCTGATCTTGTCTCTG

GCTGGATTCATCTACTACAAGAGGACGGCC

>Melanonus_zugmayeri_ERR1473851.43976819.2 com

CGGTGCTATCNAGGTACGCGCAGAAAGAGCCAGTACAGACCAGGACAGACCAGAACAGAC

CAGAGCAGACCAGTACAGACCAGAACAGACCAGACCAGTACAGACCAGAACAGACCAGTA

CAGACCAGAACAGACCAGTACAGACCAGAA

>Melanonus_zugmayeri_ERR1473851.48106054.1 com; beta-2

GCTTGAAGCAGCCCAAGATTTATGACTGGGGTAAGAGGGTGACATGAAGTACGACAGGTT

ATACTGGATTAAAGATTAACAGGGCAAAATTGTTCCGCTTTTCTGTATCAACAATAACAT

GCCACTTTATGATAAATGTGTTTTCANTGG

>Melanonus_zugmayeri_ERR1473851.48106054.2; beta-2

ACTTGGCTGAGGAACGGACAAGAGGCGACATCTGATGTGACGTCCAGTGAAGAATTGCCT

GATGGGAATTGGTTCTACCAGAGCCACTCATACCTGGAGTTTACACCCAGATCCGGAGAG

AAAATCTCCTGTATGGTGGAGCACGCCAGC

>Melanonus_zugmayeri_ERR1473851.50709829.1; beta-1

TCANATCGCAATCCATTGTCAGGTTAGTTCCTATGAAGGTCCTGCGGTGTACCTGGAGCA

GTTCTACTTTAACAAGATATTGGAGCTCCAATACAACAGCAGTTTGGGAAAAGTGTTTGG

CTTCACAAAGAAAACAAAGCCAATTGCAGA

>Melanonus_zugmayeri_ERR1473851.50709829.2 com

GCAAAAGAAATATACATCTGGGTTATCATCTTCTGACGCCAGGTTATTATCACAGAACAT

GTAGAAATTTCCAGAACACAAAGGTTTTTGGGAAATGTCTTTCTTCCTGGGAGTCACATT

GGAAGATCAGTACCACTCTAACATATGTTG

>Melanonus_zugmayeri_ERR1473851.53048439.1 com; beta-1

GTTCAGACGTGTGCTCTTCCGATCTTAGTGCCTATGACGGGCACGATGTCACATTCATAG

AGCAATACTACTTCAATAAGGTGTTGGAGATACAATACAACAGCAGTGTGGGGAAAACTA

TCGGCTACACAAAGAAAGCAAAGGAANTTG

>Melanonus_zugmayeri_ERR1473851.53048439.2; beta-1

TAGTGCCTATGACGGGCACGATGTCACATTCATAGAGCAATACTACTTCAATAAGGTGTT

GGAGATACAATACAACAGCAGTGTGGGGAAAACTATCGGCTACACAAAGAAAGCAAAGGA

AATTGAGATCGGAAGAGCGCCGTGGCGGGA

>Melanonus_zugmayeri_ERR1473851.54447277.1 com; beta-1

ACACTCTGATCAGTGATCAATAGTCTCAGTGAATCAATCTGATCTGATCTGTCCCTTCAG

ATGGATTTCGGAGTTATGTGATGTACCGTTGTGATTTTAACTCGACTGATCCAAAGGACA

TCGAGTTCATCAAGTCTTGGCATTTCNACT

>Melanonus_zugmayeri_ERR1473851.54447277.2

TGTCAGTGTGTGTGAAGTCAACAGAGGAACATGGCTTCATCCTTCCTCAGCTTCTCACTC

CTCTTCATCAGCCTCTACACAGCAGGTAGGATCAATACACTCTGATCACTGTGATCAATA

CACCCTGATCATCACTACACTCTGTTCACT

>Melanonus_zugmayeri_ERR1473852.362026.1; beta-2

ATTAATCATTATATTAATCTTTGTGTGTTTAAGTCAAGCCGTACGTCAGGCTGCACTCCA

CGACGCCCTCTGGTGGTCAGCATCCCTCCATGTTGGTCTGCAGCGTCTTCGACTTCTACC

CCCAACACATCAAAGTGAGCTGGCTCAGAG

>Melanonus_zugmayeri_ERR1473852.362026.2 com

CCTGGAGTACACGCCCAGGTCAGCCCAGGTCCAGTCAGGTCCAGGTCCAGGTCCAGGTTC

TGGTTCTGGTTCAGTCAGAACTCTGATGTGTTGGTTTGTGTTGGTTCAGGTCTGGAGAGA

AGATCTACTGTGTGGTGGAGCACGCCAGCC

>Melanonus_zugmayeri_ERR1473852.15236946.1 com; beta-2

TTCATTCCTACCTTGAGTACACCCCAGCTGCAGGAGAAAAAATAACCTGCATGGTCGAAC

ACCTCTCTCTCTCTGAACCAGTGCTTCAGGTCTGGGGTAAGTGTGCCACTGACTATGAGG

CAAATTGTATGTCGGATAAGAATATGTTTA

>Melanonus_zugmayeri_ERR1473852.15236946.2; beta-2

TTTTCTTTTTTTATAACATTCACTTTTTAGCAAGTAATCTTACAGCTGAACCCACCATCA

AGCTGAATTCAGTAAAGCAGCCGTCCATGCTTGTGTGCAGTGCCTACAACTTCTATCCAA

AACAAATCCACATGACGTGGTTGCGGAACG

>Melanonus_zugmayeri_ERR1473852.18457413.1; beta-2

CAGTTCCACTGATGAGCTGGCAGATGCTGATTGGTACTACCAGATCCACTCTCACCTGGA

GTACACGCCCAGGTCAGGTCCAGGTTAGGTCAGGTCCAGGTTTTGGTTCAGTTTCTGGTT

CAGTCAGAACGCTGATGTGTTGGTTTGTGT

>Melanonus_zugmayeri_ERR1473852.18457413.2 com

GTCTGCTCACCTGTCTGTCTGTTTACCTGTCTCTCTCACCTGTCTGTCCTCAGCCCCGTC

CATGCCTGAGTCTGAGAGAACAAAGATCGTCCTCGGAGCCTCAGGACTGATCCTGGGTCT

GATCTTGTCTCTGGCTGTATTCATCTACTA

>Melanonus_zugmayeri_ERR1473852.18748270.1

ACAGACCAGACAGAAAAGCTTCTTGCTGATATGAATGTTTATAAAAATGCTTTAAAAGCC

CAGAGGGTGTGTGCAGCATTTGGCGCGTTCTTGAAAGTAGAGAAGCAACATCCAGCAGAA

GAAAAGGGTAAGTCAACATTTAGCTCTGCA

>Melanonus_zugmayeri_ERR1473852.18748270.2 com; beta-2

AGTTAAAAACAGACTCATCTGCTTCGTGAATGACTTCTACCCACCGGACATCAATGTCAG

CTGGACCAGAAATGATCGTCCGGTGTCGGAGGGGGTGTCACTCAGTCGGTATTATCCCAA

TGATGATCAAACCTTCCGCCAGTTTGCCTC

>Trachyrincus_murrayi_ERR1473857.5729859.1 com

CAGCCTCCGACTCCTCCTGCTGGAAACCCAACCTCCGCTGGGCTGAGCTGTGAAATCTGC

TACTTGTGAAACGTAGGCCATGAAAAATTAATTAGCACACGACGGTAGCGATGACCTCCG

CTGTATCTGCATGTTGATCATGAAGGAATAA

>Trachyrincus_murrayi_ERR1473857.5729859.2; beta-1

AATTACAACAAACTGGAGTTCATCACGTTCAGGAGCAGCGAGGGGAAGTATGTCGGAACC

AATACGCTTGGAGAGAAGAACGCTGAGAGATGGAACAAGGGTCCAGGCGAGATCTAAGAA

GGCGACTTACTGCTTAAACAACGTTGGAGGC

>Trachyrincus_murrayi_ERR1473859.4175329.1; TM

TCACAGACCCGTCCCTGCCGGAGTCTGAGAGGAACAAGATAGCGATCGGGGCGTCGGGCC

TGGTGCTGGGGCTGGTGCTGGCGTCAGCAGGCCTGATCTACTACCGGAGGAAAGCCACTG

GTGAGGGAGGATGGATGGATGGATGGATGGA

>Trachyrincus_murrayi_ERR1473859.4175329.2 com

CCGCAGGCCTGAGCGACTGCCGGAGGGAGGGGACGGGTGGGGCAGGATGGATGGGGGGAT

GGGTGGATGGGTGGATGGGTGGGTGGATGGATGGGTGGATGGATGGATGGATGGATGGAT

GGATGGATGGATGGATGGATGGATGGATGGA

>Trachyrincus_murrayi_ERR1473859.5695319.1 com; beta-2

CGAGGAGTGGATGAACGGGGACTGGTACTACCAGATCCACTCCTACCTGGAGTACACGCC

CAGACACGGAGAGCACATCTCCTGCATGGTGCACCATGCCAGCCTCCCTGAGCCCCTGGT

GGTGGAGTGGAGTAAGTGTTCTTACAGGGAG

>Trachyrincus_murrayi_ERR1473859.5695319.2

AGTGTGATGTTAGTGTGACGTTAATGTGATGTTCATGTTAGTGTGTTTTAATGTTAGTGT

GTGTTAGTGTGTGTTCATGTTAGTGTGTTCATGTTAGTGTGTGTTCATGTCAGGACCGAC

CCTAAATAAAAGTAATCTGAGCTGTCATTAA

>Trachyrincus_murrayi_ERR1473859.9667592.1 com; beta-2

CGAGGAGTGGATGAACGGGGACTGGTACTACCAGATCTACTCCTACCTGGAGTACACGCC

CAGACACGGAGAGCACATCTCCTGCATGGTGCACCATGCCAGCCTCCCTGAGCCCCTGGT

GGTGGAGTGGAGTAAGTGTTCTTACAGGGAG

>Trachyrincus_murrayi_ERR1473859.9667592.2

AGTGTGATGTTAGTGTGACGTTAATGTGATGTTCATGTTAGTGTGTTTTAATGTTAGTGT

GTGTTAGTGTGTGTTCATGTTAGTGTGTTCATGTTAGTGTGTGTTCATGTCAGGACCGAC

CCTAAATAAAAGTAATCTGAGCTGTCATTAA

>Trachyrincus_murrayi_ERR1473859.14956511.1; beta-1

AATTACAACAAACTGGAGTTCATCACGTTCAGGAGCAGCGAGGGGAAGTATGTCGGAACC

AATACGCTTGGAGAGAAGAACGCTGAGAGATGGAACAAGGGTCCAGGCGAGATCTAAGAA

GGCGACTTACTGCTTAAACAACGTTGGAGGC

>Trachyrincus_murrayi_ERR1473859.14956511.2 com

CAGCCTCCGACTCCTCCTGCTGGAAACCCAACCTCCGCTGGGCTGAGCTGTGAAATCTGC

TACTTGTGAAACGTAGGCCATGAAAAATTAATTAGCACACGACGGTAGCGATGACCTCCG

CTGTATCTGCATGTTGATCATGAAGGAATAA

>Bathygadus_melanobranchus_ERR1473863.7278419.1

TTACAGTTTAGGTTCCTCTCCTTACTTCCTTACCTGCTCTCTCCTAATCTCTTCTTCTCC

TTCTGTTTCACTGTTTCTGGACAAACTAGGACAATTTATGATCAAATTAAAACGTTACCT

CTTGATTGATTGATTGATTGATTGATTGAT

>Bathygadus_melanobranchus_ERR1473863.7278419.2 com; beta-2

TTATCACCTGGAAGAGGGACGGGCAGGAAGTGACCTCAGACGTGACGTCCACAGAGGAGC

TGGCAGACGGGAACTGGTACTTCCAGAGCCACTCCTACCTGGAGTACACGCCCACACCTG

GGGAGAAGATCAGCTGTATGGTGGAGCACG

>Bathygadus_melanobranchus_ERR1473863.15644382.1

GTCTGTCTACCTGTCTATCTACCTGTCTATCTTCGTGTCTGTCTGTCTACCTGTCTATCT

ACCTGTCTATCTTCGTATCTGTCTGTCTACCTGTCTGTCTACCTGTCTATCTTCATGTCT

GTCTGTCTACCTGTCTATCTATCTGTCTAT

>Bathygadus_melanobranchus_ERR1473863.15644382.2 com; TM

TTCTGCCGATCTACCTGTCTGTTTACCTGTCTATCTTCGTGTCTGTCTGTCTACCTGTCT

ATCTACCTGTCTGTCTGTCTACAGATCCGTCCATGCCGGAGTCAGAGAGGAACAAGATCG

CCATCGGAGCCTCAGGACTGGTTCTGGGTC

>Malacocephalus_occidentalis_ERR1473866.15623733.1

ACTGTTACTACCAGATCCACTCACACCTGGAGTACACTCCCCGGTCAGTAAGCCGTCTCC

AACCGCAACTCGCTCGGACCACTCCAGCGCCGCCCACTTGATCCCACTCCCTGCACTGAC

TCCATCATATGCGTTGATCAGCGTGAGTAA

>Malacocephalus_occidentalis_ERR1473866.15623733.2

ANNNNNNNNNNNNNNNNNNNNNNNNNNNNNNNNNNNNNNNNNNNNNNNNNNNNNNNNNNN

NNNNNNNNNNNNNNNNNNNNNNNNNNNNNNNNNNNNNNNNNNNNNNNNNNNNNNNNNNNN

NNNNNNNNNNNNNNNNNNNNNNNNNNGGNN

>Malacocephalus_occidentalis_ERR1473866.32278067.1; beta-1

ACTGATTGTTTCTGTTCTTCAGATGGATTCATGGAATATGTAGTGAGCCGTTGTGACTTT

AACTCGTCTGATCTGAAAGACATCGAGTACATCAGGTCTTACTATTACAACAAGGTGGAG

TACACCAGGTTTGACAGCAATGTGGGGAAG

>Malacocephalus_occidentalis_ERR1473866.32278067.2 com; beta-1

GTACATCAGGTCTTACTATTACAACAAGGTGGAGTACACCAGGTTTGACAGCAATGTGGG

GAAGTATGTTGGATTCACAGAGCGCGGTGTGAAGAACGCAGCGTTCTGGAACAATAATCC

TTCACAACTGGCTAGTGAGAGGGCTGAGAA

>Malacocephalus_occidentalis_ERR1473866.33399068.1 com; TM

GTCTGAGAGGAACAAGATTGCCATCGGAGCCTCTGGACTGATCCTGGGTCTGACCTTGTC

TCTGGCTGGTTTCATCTACTACAAGAGGAAGGCCCGAGGTCAGAACACTACACCTGATTC

AGACCCGGTTCAGACCAGTTGATAGCGGTC

>Malacocephalus_occidentalis_ERR1473866.33399068.2 TM

GTCTACCTGTCTCTGAGCTCACCTGTCGGTCCCTCTACCTGTCTGTCCTCAGACCCGTCC

ATGCCTGAGTCTGAGAGGAACAAGATTGCCATCGGAGCCTCTGGACTGATCCTGGGTCTG

ACCTTGTCTCTGGCTGGTTTCATCTACTAC

>Malacocephalus_occidentalis_ERR1473866.37837891.1 com; beta-2

GTGCAGTGTGTATGACTTCTACCCAAAACAAATCAGTGTGACCTGGCTGAGAGACGGACA

TCCCATCACCTCGGATGTCACTTCCACTGACGAGCTGGCAAACGGCGACTGTTACTACCA

GATCCACTCACACCTGGAGTACACTCCCCG

>Malacocephalus_occidentalis_ERR1473866.37837891.2; beta-2

CCTACCAACCATGTGATTTTCCAGCCAAGCCGTCCATGACGCTGAGCTCGGTGTCGCCCC

CTGGTGGCACGCACACGGCCATGCTGGTGTGCAGTGTGTATGACTTCTACCCAAAACAAA

TCAGTGTGACCTGGCTGAGAGACGGACATC

>Malacocephalus_occidentalis_ERR1473866.50731532.1; beta-2

AGCGTCTTCGACTTCTACCCCAAACTGATCAGAGTCAGGTGGCTCAGAGACGGACAGGAA

GTCACCTCTGATGTCACTTCCACTGATGAGCTGGCTGACGGTGACTGGTACTACCAGATC

CACTCTCACCTGGAGTACACGCCCAGGTGA

>Malacocephalus_occidentalis_ERR1473866.50731532.2 com

CACTCTCACCTGGAGTACACGCCCAGGTGAGTCCACCTACAGCTCCAGGTCCACCTACAG

CTCCAGATCCAGGTCCAGGTCCGGGTCCAGGTCCGGGTCCAGGTCCAGTTTGATGAGAGT

GTGACAGGAACACTGATGTGTTGGTTTGTG

>Phycis_blennoides_ERR1473868.13588374.1 com

AGGCTTTTAATATTTATCAACGTACAGTATGTAAATCTAATGCAGTGTTTGTGATAAATG

TGTTTTCAGACCCGGCATTTGAGTCACAGAGGAATAAGATCGCAGTCGGGACAGCAGGGC

TGCTGCTGGGTCTGGTGTTTTTAGTTGCTGG

>Phycis_blennoides_ERR1473868.13588374.2; beta-2

TGTCTATGGGTTCTTTCCCCAAGTTGAGCCCTCCGTCAGGGTGAGATCAGTTGATGCAGC

GAGCAGCAAACATCCAGGCATGCTCGTCTGCAGTGTGTACGGCTTTTATCCCAAACAAAT

CAGAGTGGCTTGGCTGTGGAACGGAAAGGAG

>Phycis_blennoides_ERR1473870.508665.1

ATAACCAATATGGCGACGGCTGAAATGGCTGCACAAACCAATGGGTGACGTCACGGTGAC

TATCCACTTCTTATATACAGTCTATGTTTGTTATCCTTTATTTCCTGTACTTATTCCCTT

CTGCCTCCCTTTGATACACAGTAAGTATTTA

>Phycis_blennoides_ERR1473870.508665.2 com; beta-2

TCAGTTGAGGCAGCGGGCAGCAAACATCCAGGCATGCTCGTCTGCAGTGTGTACGGCTTT

TATCCCAAACAAATCAGAGTGGCTTGGCTGAGGAACGGAAAGGAGGTGACATCTGAGGTG

ACTTCCACTGAGGAACTGCCCAATGGGAACT

>Phycis_phycis_ERR1473871.3128027.1; beta-2

TCTGAAGTTTGTTTGTGTTGAAGGTCTGGAGAGAAGATCTCCTGCAAGGTGGAACACGCC

AGCCTGAGTGAGCCTCTGTACACTGACTGGGGTAAATACCTGTCTGTCTGCTCACCCGTC

TGTCTGTCTGCTCACCTGTCTGTCTGCTCC

>Phycis_phycis_ERR1473871.3128027.2 com

TGTGGGAGCCTCTGTACACTGACTGGGGTAATTACCTGTCTGTCTGCTCACCCGTCTGTC

TGTCTGCTCACCTGTCTGTCTGCTCACCTGTCTGTCTCTACCTGTCTGTCCTCAGATCCG

TCCCAGCCTGAGTCAGAGAGAAACAAGATC

>Phycis_phycis_ERR1473871.41249285.1 com

AAGATCGCCATCGGAGCGTCAGGTCTGCTTCTGGGTTTGATTTTGTCTTTGGCAGGTCTG

ATCTACTACAAGAGGAAGGCCAGAGGTCAGTAACACTAGATCAGTCCAGACCAGGTTTAC

ACTGGTTCACATCAGTCCACATATACACAC

>Phycis_phycis_ERR1473871.41249285.2; TM

AACAAGATCGCCATCGGAGCGTCAGGTCTGCTTCGGGGTTTGATTTTGTCTTTGGCAGGT

CTGATCTACTACAAGAGGAAGGCCAGAGGTCAGTAACACTAGATCAGTCCAGACCAGGTT

TACACTGGTTCACATCAGTCCACCTATAAA

>Phycis_phycis_ERR1473871.42393311.2 com; beta-1

AATTTATGAGGTTTAACAGCACTGTGGGGAAGTATGTTGGATATGATAAGTTTGGTATCT

ATAACGCAGAGCGCTGGAACAACGATCCTGCAGAGATGGCACGAAGGAGAGCTGAGAAAG

AGAGATACTGCAAACATAATATAAAGATAC

>Phycis_phycis_ERR1473871.42393311.1; beta-1

AACTAAGTAACTTGAACCTGTTTCTCTTTCTGCTCCAGATGCGTTTTTGTGGTATTACCT

GGACACATGTGTGTACACCTCCTCTGAACTTCCTGACATAGAGTACATACGCTCTCTGCA

TTTCAATAAGATAGAATTTATGAGGTTTAA

>Lota_lota_ERR1473872.27519403.1

TTCCCATTCTAAGACACATAGTTCTTGTTAGTGCTTGATTAGATGTGCTTGCCATATGAT

GCAAAGATATGAGGTGTTTATGATTTTTGTGTATGCTGTTTTGTGATTTCTGTGTTTAGT

TTTGAAATTAATNCAATAGCCAGATGTATC

>Lota_lota_ERR1473872.27519403.2 com; TM

GTGTGTGTACCCTCTATACTGTCAGACTCCTCCATGCCCAAGGCTGAGAGGAATAAGATT

GCCATTGGGGCTGCTGGCCTGCTGCTGGGACTGATGGTCTGTGCTGCTGGACTGCTCTAN

TACAGGAAGACATCAAGAGGTTAGCAGTTG

>Lota_lota_ERR1473872.28413271.1 com; beta-1

CAGCAGTGTGGGGAGGTNTGTTGGATTCACTGAGCGAGGTGTGAAGGTCGCTGAGAACTG

GAACAAGGATCCTTCAATACTGGCTAGGTTGAGGGCTGAGAAGGAGAGGTACTGTCTACA

CAACATTAAGATCTGGTACAACAACGTTCT

>Lota_lota_ERR1473872.28413271.2; beta-1

TAATCAATACTACTGTATTTCTCAGTGAGGNTGTGTTTCTTCTTCAGGTGGATTCATGGA

GTTTATGTTGAACCGTTGTGACTTTAACTCCACTGATCTGAAGGACATCGAGTACATCCA

GTCTTATTATTACAACAAGATAGAATACAG

>Theragra_chalcogramma_ERR1473886.15640894.1; beta-2

CAAGAACCTTCTCAATGTAGAGAGAGAGAGAGAATTAAATGAGTGACTAATCTGTGTTAT

TTTGACTTAATTTACAGTGAAGCCCTCTGTCAGACTTAGCTCAGGGACACCCTCTAGTGG

CAGACATCATGCCATGCTTGTGTGCGGCGC

>Theragra_chalcogrammus_ERR1473886.15640894.2 com; beta-2

TGCTCCCCACGGGTGAGTTTGTTGATGGAGACTGTTCCGCCCAGATCCACTCCCAGCTGG

AGTACGCGCCCAGTTCTGGGGAGAAGATCTCCTGCATGGTGGAGCATGCCAGCCTGGACA

AGCCCATTGAGGAGCACTGGGGTGAGCTCA

**3.3 CD4-2**

>Merluccius_capensis_ERR1473850.21701633.1; Ig2

GACGCAGGGACCTGGGGGTGTACGTTCCCCCATGGTGGGACGACGTACAACGTCAGCCTG

GCCATCAAAGTTCAGGGTAGGTACACTGGCTTTTACCAAACTCGTTGCATCTGCTTTTAA

TCATGACAAACACGACCACTGGTAAAAGCA

>Merluccius_capensis_ERR1473850.21701633.2 com

CAGAACCTGCTCCTAAGACAGCTGCCCCGCCCCCTTCCCAAACCTCGGGGGACGTCAATA

AGCCAACCTGCCTCGGCTGTGAGTTCTGCTTGTGTTTGTTTGGGTAAACCCTCTATGTGT

GCACATATTCATGACAATGATTCTTCCCTG

>Merluccius_capensis_ERR1473850.24696544.1; Ig2

TGAGGTTAAAGGTGTGGAGCAAGGCCTCACAGTGAAATGGAAGAGTCCAGATGGAGGAAC

ACACGATGGGACGCATGAGCTCAAGCCTGTAGCTCTCTCACATGCAGGGACCTGGACATG

TACAGTCTCCTACAACAGCCAGACGCACAG

>Merluccius_capensis_ERR1473850.24696544.2 com

AAAGGTATACAACACTTTAGCTTCATCGCAAGTCTCTGTCTGTGCTTCTTAGCACTACTA

AAGCTATCCATGTGGTCAATGGAAAAACAATAAGATGTACTGTATTTTTGCTTCCAGAGC

CTCCTCCTCCTACAACACCAACAACGACCC

>Merluccius_capensis_ERR1473850.28101549.1 com; Ig2

CCCCGGGTCTCGTCACGGTGCTCGGCCCCGGTCCCTACCGTGCAGGGGAAAAGAACCGAT

GGAAGCTCCCCCTCGGGGTCAAAGGCGGTTCAACTCAAACCTGTAACCCGCTCTGACGCA

GGGACCTGGGGGTGTACGTTCCCCCATGGT

>Merluccius_capensis_ERR1473850.28101549.2

CTCTTCCACTGCACCGTAGAGACCAGGTGGAAGCCCACTCTCGGGGTCAAAGGCGGGTCA

CCCCCACCCTGTAACCCGATCCGCGGCAGGGCCACGGGGGGTTACGCTCCGCCAGGGAGA

GACGGACGAGGTGGCGCAGAGCGGACAGGG

>Melanonus_zugmayeri_ERR1473851.41782205.1 com; Ig1

AAAACAGAGCAGACTGTCACCTTGGAGTGTGGATTCACCGTTTTCAAATCTGGCCTGGAG

TGGCGTCATAAAAATGAACGGATTTTTTCCATTGATGGTAGAAATGGCTTCAAACGTAAA

GGTATAAACACAGCGATCTTAAACATTACC

>Melanonus_zugmayeri_ERR1473851.41782205.2

ATGAGATTTTATGGTATGATTGTAGATGTAGATTGTATGTTTTCTATCTGGGAAAATTCT

CCAGTGAATATACCCTACTAAGTGAAGTGTGATTCCCTCTCTTCAGTGCTGGGTGCACTT

TCTGCTGCTGGCAACGTGANCGTCACAAAA

>Trachyrincus murrayi ERR1473859.13105516.2; CY

CATAATTAGCGTGAACACGTCTCCTCTTCTCCTTCCTCTTTATCGCAGCCGTCCAGCTGC

GGAGAAACCACAGCGAGGACGTCAGAAAGCGAGGACGTCCACCCTGGCTCAGAAGCCCCT

GCTAATTGAGTGATGGGAGAGACGGATAGAA

>Trachyrincus_murrayi_ERR1473858.1849697.1 com; CY

AGAAGCCCCTGCTAATTGAGTGATGGGAGAGACGGATAGAAGAGAGCAGGGACCCGGGGA

GAGGGACAGAGGATTAGGTGGAGATACAGAAAAAGAGAGCAAGTGAAAGAGAACCCCTGT

GAAGCATGTGGCCTGAATGTAAATGGACTTT

>Trachyrincus_murrayi_ERR1473858.1849697.2; CY

TTTTTTTTTTCTTTTCCACTGTAGAGAAAATTGCAGAAGATGATGAAGAATGGCCAAAGA

TCTGCGAGGTCCAAGCCGTACTGTCAGTGTAACTGGTAAATTCTCCTCACTTCGCTTCTC

TATAATAATTTTTTTTTAAAAGAGTCTCAGT

>Trachyrincus_murrayi_ERR1473859.13105516.1 com; CY

CCGTCCAGCTGCGGAGAAACCACAGCGAGGACGTCAGAAAGCGAGGACGTCCACCCTGGC

TCAGAAGCCCCTGCTAATTGAGTGATGGGAGAGACGGATAGAAGAGAGCAGGGACCCGGG

GAGAGGGACAGAGGATTAGGTGGAGATACAG

>Bathygadus_melanobranchus_ERR1473863.58116857.1; Ig1

AGTGATCGGATTATTAGTGTTAATTCAAGGTCCGGCTTTCCTCTCAAAGGTAGTCATAAA

AACACATAATAGAGCAAAGTATCATTTTATAATCACCATTGTTGCTGTTTACACTACATG

TATTCTCTGCATTAGGTCCAGCTGCCGTTC

>Bathygadus_melanobranchus_ERR1473863.58116857.2 com; Ig1

TTTACACTACATGTATTCTCTTCATTAGGTCCAGCTGCCGTTCATTTGAGGTCAAAGATG

AAGTCTGAGAAGAACCTTGAGATCTCTGGGGTAAAGAGAGAAGATGCCGGAAAGTTCACT

TGCGTGGCAAACGGGAAATCTGTTGAGCAC

**3.4 CD74a**

>Merluccius_polli_ERR1473847.19205782.2 com; TM Nterm-CLIP

AGCAGATCCACACTTTGCAGAAGAACTCCGAGCAAATGACCAAAGAGATGACTCGCACTT

CCCGGGGTAAAAGACTTAGATGAAAAATGAAGTCCTAATGATCTGTCAGGGCAGTGTGAG

TGTGAGTGTACCAATAATGAGCAATGGTCC

>Merluccius_polli_ERR1473847.19205782.1; TM Nterm-CLIP

CTGTGGGTGAGTAGGGGCTCCAACAGGCGAGCCTTGAAGGTGGCCAGCCTGACAGTCCTG

GCCTGCCTTCTGGTGGCCAGCCAGGTCTTCACAGCCTACATGGTGGTGAGCCAGAAGCAG

CAGATCCACACTTTGCAGAAGAACTCCGAG

>Melanonus_zugmayeri_ERR1473851.1976448.2 com; TM Nterm-CLIP

ATCTGCCTCCCTCGGGGGCACCACCAGCCGTCCTTTTAAGGTGGCAGGCCTGACACCCCT

GGCCTGTCTGCTGCTGGCCAGCCAGGTCTTCACTGCCTACATGGTGTTCGGCCAGAAGGA

CCAGATCCAAACCCTCCAGAAGAACAGTGA

>Melanonus_zugmayeri_ERR1473851.1976448.1

AGCACTTTTGTGAATTCCCCCAAATAACCACAAATAACTAATACATTGTCAAATTTCACA

TCCCACTTTATGTCTTTGTGAATTTAGCTGTGCGTCATGTTTTCCCCAACCCTCTTCTGC

TTTATCATCTTCAAAAAACATTTCTATTTC

>Trachyrincus_murrayi_ERR1473857.5327458.2 com

ATTCATACAGGGGAGAAGTCACCTGCCTACTTTATTTAACTCAATCTTATAATTATAAGA

ATTCAGGTGATGTCATATGGAGTTCCCCCTCATACAATATATCATACAGTTTATTAGCCA

GCATTTAACACATCCACTGTTCCTGCGATGA

>Trachyrincus_murrayi_ERR1473857.5327458.1; TM Nterm-CLIP

CCTGACGACGCTGGGGTGTCTGCTGCTGGCCAGCCAGGTCTTCACTGCCTACATGGTGTT

CGGCCAGAAGCAGCAGATCAACTCTCTGCAGAGGGACTCCGAGAAAATGGCCAAGCAGGT

GACCCGCTCGTCCCAAGGTAAAGGACGTCAT

>Bathygadus_melanobranchus_ERR1473863.46876077.2 com; Thy

TACAAGCCCATGCAGTGCTGGCACAGCACCGGCTTCTGCTGGTGCGTGGACGAGAGCGGC

ACCCCCATCGAGGGCACCTACATGCGTGGCAGACCTCAGTGTCAGAGAGGCAGAGGTAAC

ACGCACCCGCTGAATTAAACGTTGCGTCTG

>Bathygadus_melanobranchus_ERR1473863.46876077.1; Thy

TCTCCATCCCTCAGCCCCTGAGCTGACCAAGTGCCAGAAGGAGGCGGCTCCTAAAGGCGT

GAGGCCCGGTTTCTTCAAGCCGCAGTGTGACGAGCAGGGCCGCTACAAGCCCATGCAGTG

CTGGCACAGCACCGGCTTCTGCTGGTGCGG

>Malacocephalus_occidentalis_ERR1473866.6838980.2; Thy

GCACTTTCCCCTAAGCCCTTCTGCTCGAGACCAAGTGCCAGTTGGAGGCGTCTCGGGGCA

AGGTCCTGGGTTCCTTCCAGCCGCAGTGCGACGAGAGCGGCAACTACCGCGCCATCCAGT

GCTGGCACAGCACTGGCTTCTGCTGGCGCG

>Malacocephalus_occidentalis_ERR1473866.6838980.1 com; Thy

GCCATCCAGTGCTGGCACAGCACTGGCTTCTGCTGGTGCGTGGACAACAACGGCGCAGCC

ATCGAGGGCACCTACACCCGCGGAAGGCCCCATTGTCCGAGAGGTACGCGTCTCAGCTGT

CTCTTTAGGCTTTCCACAATAAAAGCGTCC

**3.5 CD74b**

>Melanonus_zugmayeri_ERR1473851.26907834.1 com; Thy

CAATGGACAGGTCACCTGCGGTTCAGCTGTCATTGTTGGTGAGACATGATATAGATAGAT

ATAGATGATGATATAGATGGTCAAGTCGGACGGAAAATAGATTATGGGCGCGAAAACTAG

CAAAAATTAGCAGAATATCTCAGAACTTAA

>Melanonus_zugmayeri_ERR1473851.26907834.2; Thy

TTAGTCTCTTCACAAATTTTCTGTTGGTCAAAGATGATTTAACTCCTCTCCTCTCCCAGC

CCCCCTTCAGGTCACTGACTGCCAGCTCGAGGCCGCTGGTAAGAAGCCTGTGCCGGTGCC

AGGTTTCCGCCACAGCTGCGACGCGCGCGG

>Melanonus_zugmayeri_ERR1473851.36808274.1 com; TM Nterm-CLIP

TGTGTCCTGATTGCGGGCCAGGTGATGACCGCCTACTTCCTCCTCAGCCAGAGGAGCGAC

ATCAAATCTCTGGAGGAGCAGAGCAACAACCTGAAGACAGAGCTGACGAAGGGAAGATCT

GGTGAGAAGGCCAAATTTAGTTCAGATTGG

>Melanonus_zugmayeri_ERR1473851.36808274.2

AAGACACGAAAAATTCAAGCTTCACTTTGGTGTCTGCTTGGCAACAGCTGATTTCATCGC

TCAGTCATCTTCTCTACCTCCACTCCCCTCACCTCCTTCCTCCTTTTTCCTCTCGCCCCC

CAGTGGCAGCTCCTCCCCGNCCTATACGCT

>Melanonus_zugmayeri_ERR1473851.42003943.1

GTTAAATTTTGAATTAATGGGAATTTCCAAAGGAGGCCTTGCTGTAGTTAAAAAGGGTAA

AAGGAAGACACGAAAAATTCAAGCTTCACTTTGGTGTCTGCTTGGCAACAGCTGATTTCA

TCGCTCAGTCATCTTCTCTACCTCCACTCC

>Melanonus_zugmayeri_ERR1473851.42003943.2 com; TM Nterm-CLIP

CGGGCCATGTNATGACCGCCTACTTCCTCCTCAGCCAGAGGAGCGACATCAAATCTCTGG

AGGAGCAGAGCAACAACCTGAAGACAGAGCTGACGAAGGGAAGATCTGGTGAGAAGGCCA

AATTTAGTTCAGATTGGGAAAAATATACTG

>Melanonus_zugmayeri_ERR1473852.7415532.1; Thy

GTCACTGCTGGTGTGTCAACCCAGCCAACGGAGAACAGATCCCTGGAAGCGTGAACAATG

GACAGGTCACCTGCGGTGCAGCTGTCATTGTTGGTGAGACATGATATAGATAGATATAGA

TGATGATATAGATGGTCAAGTCGGACGGAA

>Melanonus_zugmayeri_ERR1473852.7415532.2 com

AATAGATTATGGGTGCGAAAACTAGCAAAAATTAGCAGAATATCTCAGAACTTCAGTGAA

AGTTCCTTTGGTCAAGAAACACAGATGTATTTTTTGGTGCGGATCCAGATTTTTTATGTT

TTCTACATGAAATATTGCAGTAAATTGGGT

>Trachyrincus_scabrus_ERR1473854.23104984.1

GCCTCAGGAATCACATTTGTTTCATTTTGCAAATTACCAGAGCGGGTTGACAAAGAACAG

ATTCCTTGGTCACTGACTGCCCAGCCAAGGTTAAACTAAAAAGACTGCCCTTTCTCACTT

TCTCCCCTATTTCAATTTCATTTTGATATT

>Trachyrincus_scabrus_ERR1473854.23104984.2 com; TM Nterm-CLIP

CCTTCTCTTTTCTCTCCTTCTCCTCCCCGCCACGCGCCGACTGTGCCCGTCTTTTCCCTG

TGCCCACCCAGTGGCAGCTCAACCCGGGCCTACAAGGTGGCAGGTCTCACCCTGCTGGCC

TGTGTGCTGATCGCGGGCCAGGCTGTGATG

>Trachyrincus_scabrus_ERR1473854.58716562.1; TM Nterm-CLIP

GCTTAGTGGCTCCTCCCCCTCGTCCAGCCGGGCCTGTAAGGTGGCGGGGATCACCGTGTT

GGCGTGTCTGCTGATCGCCGGCCAGGGATTGGTCGCCTACTTCATGCTCCGCCAAGGAAA

TGACATCACAACTCTGGAGAAGACCGGAAA

>Trachyrincus_scabrus_ERR1473854.58716562.2 com; TM Nterm-CLIP

CTTCCTATCTGCTTAGTGGCTCCTCCCCCTCGTCCAGCCGGGCCTGTAAGGTGGCGGGGA

TCACCGTGTTGGCGTGTCTGCTGATCGCCGGCCAGGGATTGGTCGCCTACTTCATGCTCC

GCCAAGGAAATGACATCACAACTCTGGAGA

>Laemonema_laureysi_ERR1473862.22060450.1 com; TM Nterm-CLIP

TGCTGTCCTGTGTCCTGATTGCAGGACAGGTGATGATCGCCTACTTCCTGGTCAGCCAGA

GGAGTGAAATCAAATCTCTGGAGGAGCAGGGCAACAACCTGAAGGCTGAGCTGACCAAAG

GAGGTTCAGGTGAGGAGGGGGCTGAAGTCT

>Laemonema_laureysi_ERR1473862.22060450.2

CTATGAACATGCACAACCATCTTGGCACTTCCTCTCTCTGACTCTGTTTCAGTATCTAGT

TGTCCGTTGTCTAAATGTTTCTCCTGCCGTCCTCTACTTTGCCTGACCTCCTCCTTCATT

CTTTCGCCTCTTGCTCTACTGTGGCCGCTC
