# Supplementary material for: Major histocompatibility complex (MHC) fragment numbers alone – in Atlantic cod and in general - do not represent functional variability
Source: F1000Res. 2018 Sep 6;7:963. Originally published 2018 Jun 28. [Version 2] doi: 10.12688/f1000research.15386.2 (PMC6081975; doi:10.12688/f1000research.15386.2)
Supplement: Supplementary file 4 [file f1000research-7-17714-s0002.tgz › 17cc45cf-b757-4bd0-8e95-9f016036c3c7.docx]

**Supplementary File 2:**

Investigation of unitigs with (partial) MHC class II system genes which are listed by Malmstrøm *et al.*^1^ in their Table S7 for the non-gadiform fishes *S. chordatus*, *C. roseus*, *Z. faber*, T*. subterraneus*, *P. transmontana*, and *P. japonica*.

**Table of Contents Page**

1. Summary 2

2. References used in this supplementary file 3

3. Detailed analyses 4

3.1 *Stylephorus chordatus* 4

3.2 *Cyttopsis roseus* 10

3.3 *Zeus faber* 21

3.4 *Typhlichthys subterraneus* 32

3.5 *Percopsis transmontana* 42

3.6 *Polymixia japonica* 57

**1. Summary**

In regard to their findings of MHC class II system genes in non-gadiform fishes, Malmstrøm *et al.*^1^ present their Supplementary Table 7 (title: UTG ID for each identified ortholog in all genomes investigated). In that table, for each of the listed genes *MHC IIA*, *MHC IIB*, *CD4* (referring there to *CD4-1*), *CD74a* and *CD74b*, a unitig (UTG) name is given. Malmstrøm *et al.*^1^ did not explain their findings at the sequence level, and therefore we here add such analysis.

We downloaded the genome unitig and scaffold assemblies deposited by Malmstrøm *et al.*^1^ for the six species *Stylephorus chordatus*, *Cyttopsis roseus*, *Zeus faber*, *Typhlichthys subterraneus*, *Percopsis transmontana*, and *Polymixia japonica*, from the Dryad repository doi:10.5061/dryad.326r8. The local Blast suite Blast-2.4.0+ was used to make blast databases using the makeblastdb command. Amino acid sequences were then used for tblastn searches against these unitig and scaffold assembly databases using default parameters. Interesting unitigs or scaffolds matching the blasted sequences were extracted using the command blastdbcmd. Then the sequences were investigated by using a combination of gene prediction softwares (GENSCAN at <http://genes.mit.edu/GENSCAN.html>, and FGENESH at <http://www.softberry.com/berry.phtml?topic=fgenesh&group=programs&subgroup=gfind>) and comparison with known MHC class II system sequences in our accumulated datasets as well as by blastx analysis against the NCBI database (<https://blast.ncbi.nlm.nih.gov/Blast.cgi>).

**Table S2.** Summary of the MHC class II system molecule coding capacity of the unitigs listed in Table S7 of Malmstrøm *et al.*^1^

|  | *MHC IIA* | *MHC IIB* | *CD4 (CD4-1)* | *CD74a* | *CD74b* |
| --- | --- | --- | --- | --- | --- |
| *Polymixia japonica* | utg7180000058066 | utg7180001073264 | utg7180001882239 | utg7180001886650 | utg7180000447839 |
|  | non-classical | non-classical | complete | complete | incomplete |
| *Percopsis transmontana* | utg7180000591256 | utg7180000656275 | utg7180000135569 | utg7180000203314  incomplete | utg7180000004539 |
|  | non-classical | incomplete | complete | (scaffold: complete) | complete |
| *Typhlichthys subterraneus* | utg7180001268240 | utg7180001679958 | utg7180001171865 | utg7180000050069 | utg7180000242295 |
|  | incomplete | incomplete | incomplete | complete | incomplete |
| *Zeus faber* | utg7180002667991 | utg7180002225258 | utg7180001946853 | utg7180000026784 |  |
|  | non-classical | incomplete | incomplete | complete |  |
| *Cyttopsis roseus* | utg7180000163186 | utg7180000052012 | utg7180000048768 | utg7180001483868  not found |  |
|  | complete | complete | complete | (scaffold: probably complete) |  |
| *Stylephorus chordatus* | utg7180000350061 | utg7180000955334 | utg7180000079533 | utg7180003723408 | utg7180003206387 |
|  | incomplete | incomplete | incomplete | incomplete | incomplete |

*In Z. faber and C. roseus unitigs and scaffolds no CD74b gene was found (see also Malmstrøm et al.^1^).*

The results of our investigation are summarized in Table S2, which lists the same unitig sequences for encoding MHC class II system genes in the six investigated species as listed by Malmstrøm *et al.*^1^ in their Table S7. In addition to the unitig IDs, our Table S2 contains a summary statement of our analyses of those unitigs; for details of those analyses see the below chapter 3. In Table S2, the word “complete” plus yellow shading is used for the cases where we found that the unitigs can encode full-length classical/canonical type molecules. The word “incomplete” is used for the cases where only parts of molecules were found to be encoded, possibly because the unitig length was too short, and “non-classical” is used for the cases where the encoded MHC IIA or MHC IIB molecules are of the non-classical category. With the exception of *P. transmontana* CD74a, in the cases where the analysis of the respective unitig is summarized as “incomplete” or “non-classical”, we were unable to find other unitigs or scaffolds encoding full-length classical/canonical type proteins. Information for coding a full-length canonical CD74a molecule was found in *P. transmontana* scaffold scf7180001425484. We were unable to retrieve the *S. chordatus* unitig utg7180001483868, listed by Malmstrøm *et al.*^1^ as encoding CD74a in their Table S7a, but found that scaffold scf7180003315926 probably encodes a full-length canonical CD74a molecule (see chapter 3 below). It should be noted, however, that Malmstrøm *et al.*^1^ deem their unitig assemblies more reliable than their scaffold assemblies.

Malmstrøm *et al.*^1^ did not include analysis of *CD4-2* in their study. However, there is no reason to favor CD4-1 over CD4-2 as candidate for mammalian type CD4 function. For *CD4-2* gene in *S. chordatus* the best matching unitig and scaffold are utg7180003164412 and scf7180008089612, respectively, but none of them encodes a full-length canonical CD4-2 molecule possibly because the sequence assemblies are too short (not shown). Similarly, also the *C. roseus* unitig utg7180000023981 and scaffold scf7180003324077 appear to include only part of *CD4-2* gene.

From the results summarized in Table S2, it follows that for allowing their claim that the loss of the MHC class II system only occurred in Gadiformes (Malmstrøm *et al.*^1^), Malmstrøm and co-workers probably should do additional experiments aimed at possible amplification and sequencing of full-length intact genes (or cDNA sequences) from *S. chordatus*. Namely, *S. chordatus* is the non-gadiform fish closest related to Gadiformes, and at the next closest phylogenetic level the non-gadiform fish *C. roseus* appears to have intact MHC class II system genes except possibly for *CD4-2* (Table S2; for the species phylogeny see Malmstrøm *et al.*^1^).

**2. References used in this supplementary file**

1) Malmstrøm, M., Matschiner, M., Tørresen, O.K., Star, B., Snipen, L.G., Hansen, T.F., Baalsrud, H.T., Nederbragt, A.J., Hanel, R., Salzburger, W., Stenseth, N.C., Jakobsen, K.S. & Jentoft, S. Evolution of the immune system influences speciation rates in teleost fishes. *Nat. Genet.* **48**, 1204-1210 (2016).

2) Lim, E.H. & Brenner, S. Sequence analysis of Mhc class II beta-like fragments in the pufferfish Fugu rubripes. *Immunogenetics* **42**, 432-433 (1995).

3) Zhou, Z., Callaway, K.A., Weber, D.A. & Jensen, P.E. Cutting edge: HLA-DM functions through a mechanism that does not require specific conserved hydrogen bonds in class II MHC-peptide complexes. *J. Immunol.* **183**, 4187-4191 (2009).

4) Painter, C.A. & Stern, L.J. Conformational variation in structures of classical and non-classical MHCII proteins and functional implications. *Immunol. Rev.* **250**, 144-157 (2012).

5) Dijkstra, J.M., Grimholt, U., Leong, J., Koop, B.F. & Hashimoto, K. Comprehensive analysis of MHC class II genes in teleost fish genomes reveals dispensability of the peptide-loading DM system in a large part of vertebrates. *BMC Evol. Biol.* **13**, 260 (2013).

**3. Detailed analyses**

This chapter describes the analysis of the unitigs listed in Table S2 and of some other findings when searching for full-length classical/canonical MHC class II system genes in the non-gadiform fishes *S. chordatus*, *C. roseus*, *Z. faber*, T*. subterraneus*, *P. transmontana*, and *P. japonica*. The full-length unitig sequences are shown, as well as the relevant encoded (parts of) MHC class II system molecules. In cases where we deem that helpful information, we show the encoded sequences together with the encoding nucleotide sequences.

***3.1 Stylephorus chordatus***

**For the non-gadiform fish *Stylephorus chordatus* no intact full-length MHC class II system genes were reported.**

*Stylephorus chordatus* is the non-gadiform fish investigated by Malmstrøm *et al.*^1^ which is closest related to the gadiform fishes, and hence important for deciding the phylogenetic stage at which functional MHC class II system genes were lost.

Upon inspection, none of the unitig sequences listed in their Table S7 encodes a full-length canonical molecule, possibly because the unitigs are too short.

In summary, our findings for their Table S7 listings are:

**MHC IIA:** Table S7 mentions utg7180000350061. This sequence only encodes an MHC IIA α2 domain, possibly because the unitig is too short. We were unable to find *Stylephorus chordatus* unitigs or scaffolds reported by Malmstrøm *et al.*^1^ with full-length classical *MHC IIA* sequences

**MHC IIB:** Table S7 mentions utg7180000955334. This sequence only encodes an MHC IIB β1 domain, possible because the unitig is too short. We were unable to find *Stylephorus chordatus* unitigs or scaffolds reported by Malmstrøm *et al.*^1^ with full-length classical *MHC IIB* sequences.

**CD4:** Table S7 mentions utg7180000079533. This sequence contains exons for a leader peptide plus complete CD4-1 ectodomain, but does not include the essential transmembrane and cytoplasmic domain regions. We were unable to find *Stylephorus chordatus* unitigs or scaffolds reported by Malmstrøm *et al.*^1^ with full-length *CD4-1*.

**CD74a:** Table S7 mentions utg7180003723408. This sequence only encodes the middle part of CD74a, possibly because the unitig is too short. We were unable to find *Stylephorus chordatus* unitigs or scaffolds reported by Malmstrøm *et al.*^1^ with full-length canonical *CD74a*.

**CD74b:** Table S7 mentions utg7180003206387. This sequence only contains exons encoding the C-terminal part of CD74b, possibly because the unitig is too short. We were unable to find *Stylephorus chordatus* unitigs or scaffolds reported by Malmstrøm *et al.*^1^ with full-length canonical *CD74b*.

Below the respective unitig sequences are shown with their relevant protein coding capacity:

***Stylephorus chordatus* *MHC IIA***

>utg7180000350061 length=653 num_frags=440 Astat=-220.00

TGACCTTCTACTCCACAATATGTTTCTTACCTGCAAGTATATATATATATATATATATATATATATATATATATATATATTATAATGTAATCTAATGTTTATATTGTAACAGATGCTCCTCACAGCATCATCTTCACCAGAGACGTTGTGGAGGTTGGAGAGGAGAACCTCCTCATCTGTCATGTGATTGGATTCTTCCCTGCTCCTGTGACTGTTGGCTGGACTAAGAACAACATCAACGTGACACAAGACACCAGCCTGAAGGTGCCCTTCCCCAACAAAGATGGAACCTTCAACCAGTTCTCCAGCCTCAAGTTCACCCCCCAGTCTGGAGATATCTACAGCTGCTCTGTGGAGCACCGTTCCCTGGAGAAACCTCTCACACGATTCTATGGTAACACTAGTACTATATACTACTACTGTATATATTACTACATACATATAATACTACACCTCACCCTGGAGTAACACTAGTACTATATACTACTACTGTATATATTACTACATACATATAATACTACACCTCACCCTGGAGTAACACTAGTACTATATAATACTACTGTATATATTACTACATACATATAATACTACACCTCACCCTGGAGTAACACTAGTACTATATAATACTACTGTATATATTACTACATACATAT

In this utg7180000350061 sequence, only an MHC IIA α2 domain exon can be found:

10 20 30 40 50 60

TGACCTTCTACTCCACAATATGTTTCTTACCTGCAAGTATATATATATATATATATATAT

70 80 90 100 110 120

ATATATATATATATATATATTATAATGTAATCTAATGTTTATATTGTAACAGATGCTCCT

A P

130 140 150 160 170 180

CACAGCATCATCTTCACCAGAGACGTTGTGGAGGTTGGAGAGGAGAACCTCCTCATCTGT

H S I I F T R D V V E V G E E N L L I C

190 200 210 220 230 240

CATGTGATTGGATTCTTCCCTGCTCCTGTGACTGTTGGCTGGACTAAGAACAACATCAAC

H V I G F F P A P V T V G W T K N N I N

250 260 270 280 290 300

GTGACACAAGACACCAGCCTGAAGGTGCCCTTCCCCAACAAAGATGGAACCTTCAACCAG

V T Q D T S L K V P F P N K D G T F N Q

310 320 330 340 350 360

TTCTCCAGCCTCAAGTTCACCCCCCAGTCTGGAGATATCTACAGCTGCTCTGTGGAGCAC

F S S L K F T P Q S G D I Y S C S V E H

370 380 390 400 410 420

CGTTCCCTGGAGAAACCTCTCACACGATTCTATGGTAACACTAGTACTATATACTACTAC

R S L E K P L T R F Y

430 440 450 460 470 480

TGTATATATTACTACATACATATAATACTACACCTCACCCTGGAGTAACACTAGTACTAT

490 500 510 520 530 540

ATACTACTACTGTATATATTACTACATACATATAATACTACACCTCACCCTGGAGTAACA

550 560 570 580 590 600

CTAGTACTATATAATACTACTGTATATATTACTACATACATATAATACTACACCTCACCC

610 620 630 640 650

TGGAGTAACACTAGTACTATATAATACTACTGTATATATTACTACATACATAT

***Stylephorus chordatus* *MHC IIB***

>utg7180000955334 length=583 num_frags=30 Astat=48.00

AACATAACATTATTGGTATGAATATATATGATATATGGTATATGATGTATTCTATACTGATACTGCATCAACTAACATATAATAATAATAATAATAATAATAATAATAATAATAATAATAATAATAATAATAATAATATTCTTCTTCATACAACAACTCTTATAACTTCCTGTCAGAGCTGATGAATCTTTATTGTTTTCCTCTTTCTTCCTTCAGATGGATATGAGGAATATACGGTTCAGCGCTGCCTGTATACGTCTACTGAGCTGCAGGACATAGAGTATATTTACTCTAACTACTTCAACAAGGTAGAGTACGTCACGTTTAACAGCACTGTGGGGTTCTACGTTGGCAACACTCCTTTTGGAGTGAAGTACGCTGAGAAGTGGAACAAAGATCCTGCAGAGTTGGGCTACAGGAGAGCAGTGCGTGAGAGAGTCTGCAGAACTAATAGTCAGATCTATTATGAAGCCATACTGGGAAGGACAGGTGAGTTGATCTGTGGTCACACTAACACTGATGTTAATAACCAACTATAACTATCTGTTACATGTGTTGTTATTACTGATTATTATTGTTGGAT

In this utg7180000955334 sequence, only an MHC IIB β1 domain exon can be found:

10 20 30 40 50 60

AACATAACATTATTGGTATGAATATATATGATATATGGTATATGATGTATTCTATACTGA

70 80 90 100 110 120

TACTGCATCAACTAACATATAATAATAATAATAATAATAATAATAATAATAATAATAATA

130 140 150 160 170 180

ATAATAATAATAATAATATTCTTCTTCATACAACAACTCTTATAACTTCCTGTCAGAGCT

190 200 210 220 230 240

GATGAATCTTTATTGTTTTCCTCTTTCTTCCTTCAGATGGATATGAGGAATATACGGTTC

G Y E E Y T V Q

250 260 270 280 290 300

AGCGCTGCCTGTATACGTCTACTGAGCTGCAGGACATAGAGTATATTTACTCTAACTACT

R C L Y T S T E L Q D I E Y I Y S N Y F

310 320 330 340 350 360

TCAACAAGGTAGAGTACGTCACGTTTAACAGCACTGTGGGGTTCTACGTTGGCAACACTC

N K V E Y V T F N S T V G F Y V G N T P

370 380 390 400 410 420

CTTTTGGAGTGAAGTACGCTGAGAAGTGGAACAAAGATCCTGCAGAGTTGGGCTACAGGA

F G V K Y A E K W N K D P A E L G Y R R

430 440 450 460 470 480

GAGCAGTGCGTGAGAGAGTCTGCAGAACTAATAGTCAGATCTATTATGAAGCCATACTGG

A V R E R V C R T N S Q I Y Y E A I L G

490 500 510 520 530 540

GAAGGACAGGTGAGTTGATCTGTGGTCACACTAACACTGATGTTAATAACCAACTATAAC

R T

550 560 570 580

TATCTGTTACATGTGTTGTTATTACTGATTATTATTGTTGGAT

***Stylephorus chordatus* *CD4*** *(CD4-1)*

>utg7180000079533 length=4708 num_frags=654 Astat=276.00

(the complementary sequence is shown)

TGTGTGTGTGTGGGGTGCTTATCTAATTCCCATCGGCTATGGAGGCCCCATTCGGCTATAGGGGCAGAATTAGAATTATCTACTGGGGGTAACTCGGCCCAGGTGAGGGACTGAGACATATTCCTAAGAGCTATATGAGAGTGCAAAAATTAGAGTATAATTTGGTATAATAGTGTTAATTATATTATATTTTAGGTCTAATATGGCATTAGGGGTTTCTAATTACAGCATAATTTAGGTCTATGGTATGTTTGTTTACTGTCTAATAAAGAACAAATTGGTTCTAGTTTTTTTGTATAATTTGATACAAAGTACAGATTATTATGTCGTATTAGTAAAATAAAGTGCGTCCCAAACAACAACAGGTAAAATAAAGTGCGTCCCAAACAACAACAGGTGTGCACATGTTTGAGTGTGACAGTGAGATTGTGTCTTATATGCCATTCTGGTTTCAGAATTATTTATATGTATACGTTATAATGTATAAGTATACTTTATATAAGTATACATTTCCAGAGATAGTATTTTTAATGTAAGGTAATACACTCTTTTGAAAGTGTTTTGGATTTCATAGAGACAGAGTGCGATACCTAAACCACAGTCACAGCCATTGTTACTATTGTGGTCTTCTATGAAAAAAAGGTTTCGCACAAGTAGAAATCCGTCTTTCGTGGAAAAGAAGAGGAAGAGCGGAGGAACAGTGGGGGCGTGAGACGTAGAGGGGAAGAGATAAGGATTGGGAGAATGTCTACTCGAACAAATCAGGGCCCACCAGGCCACCACAACCAGGCACCTCTCTCTTTCTCTCCCCCTCTTTAAATCCCACCACAAGCCTCCTACCGTCTCTGTTTTTTTCTCTCTTTTATCTTTAGCAAATCTCTCTAAATCTGAGACCACCGTGTACCCCTTTCCGCTTTCTGTTCACATTCAACCCTTTGGCACGTAATAGCAGATTATGTGCATGCCTACACTGTTCAGATATAACATGTTCTAAATTCAATCAAAATTGTATTTTTACAGCTTTGAATATTCTGTTTTTGTTTTGCAAAGTTAAACATAATAAAAGTAAACTTCCTAGTGTTATATTTTCTTATCTTTAATTCCATTGGATGTAATTTTATTTTATTTGTTTTCTGTTTTGAATAAATGTCTAAAATTAGTGATTCGTTATATCACAATATAACTTTGCAAAAACTATATTTTTCAGAAGTTTGACCAAAATGAACATTTTCATCCATTGTATAATTATCATCGAGGCTGTTCTGAGCCCAACATCAGGTACGGCCACATGCAGCTATGATGTCATCATATGATGTTACTATACATATGTAGCCATGATGTCATCATATGATGTTACTATACATAATTATGCAGCCATGATGTCACCATATGATGCTATTATACATATGCAGCCATAATGTCATCATATGATGTTACTATACATAATTATAAAGCCATGATGTCACCATATGATGTTATTCTATCTATTTTTGGCAGAGTTCATTCAACGTCACTGTTGTCTTTCCCCCAGCAGCTGTGGTGATTGTCTACGTCTACGGTAAGGTGGGAGATAACATCACGCTCCCCAACGCGGAGACATACAAAGGTTTATATGTGACCTGGCATTCAGGCATTCCTGAAGAAAAAGTGCTTTTGCTGCAACGCCACAAGTTCGGAAGCGATTTGTTCACAGATGGTGAGCATCATCTTTTTTGCCTGTTCTGGGATATGGATCTTTTTTTTTTAGCATCAAAATACATAAATGCAAAAATATGAACTGACATTATAACAGGTGAACTTCCAATTTGAATGATTTAAACCTTTGAAATGTCACCATTCAGATGAGCATTGGAAAGGCCGTCTATCTTTGTCTGAAACCAATTGCTCACTGATCATCAAGGCAATCAAACCAGAGGACTTCCAACCCTTCACCTGTGTGTTGAAAGATAAAGAGAGAGATGTCTCAATCACCACATATGAACTATATGAGGGTGAGAGCAGACAGCAGCGGACGTCACATCATCATTTGATGTCAGCAAAACCCCACTGATGAGTTTGTTCACACATGGACACAGCTGTTAAACTAGTGTCATCCTCAGTGTCATCCTCTTATACTTGCATAGTAAATGTCATTTCATAAGGAATTAATTACTCATTTTATCTTATTTCTTCTATCATTTCCTTTGTCAGTGAGGGTGTCCCCAGGTGTCAGTGAGGGTGTTCCTCTTCTGTTGGCCGGGGAATCACTATCTCTATCCTGCACGGTACAGACGCCTGCGGGTGGACCACAGCCACAGATACACTGGCTCGGTCCAAAGGAGGAGAGCGTGGCTACAAACCGCAGAACCCAGGGAGGGAATCTGACAGTAGAGGGCGTCACGGGCAGAGACCACGGAGAGTGGACTTGTGTTCTTCTGAGAAATGGCATCGAATCACGCACCAAAACATCTGTTATTGTTGTGGGTAAGTCATTAGAACCTCCCGCTTTTTTGATCCGTGTGTGTGGGTGGACACCTGTGTGGGCGCCTCTTTGTTTATAAACTAGAAAATTCCTTGCATGTCAAAATTCAAACCCACCGTTTCTGTATACTATATTAGCGTGGTGTCATTCAGCACATCCAAATGACGGATCCCCCTCTCCTCTTCTCCTAGACCTCTCCCCTGGCCATTCATATCCGTACTATCTGTCCTCCTCTCCTCTTCAAATCAACTGTTCCATTCCCCTTTACCTCACCTGGGAACACCTCATGAAAACCGGCTTGCAGGGAGGATATTGGAACTTCACCCCTAGCTTCCCCTCCAGAGCTCCGCAGAAGCTCCACTCTCTACTCGCGCCAGGGAAGTCGAGCACCTGGACGACGTTAGAACACAAGGGAGCGGGGAGCATGAACACGTTGGACCTCGTGAAGAAAAATCTTTTTCCGAGCAGCGAGCCGGCGTCAGAGGCGGACAGGGGAGAGTACACCTGTGCCCTGGAATTTAAAGGCGGGGTCACACTGCAGAGAAGCGTGCGCGTGGAAGTGTTGCAAAGTAAGTGGGCCCTCCGAGCGCAGGTTAAACAGCACACTGTGAAGATGGATCGGATTTCATTTCCTTTTGCTTTAGGTTTTTTCCTCTCTGCGTATACTACAGCTCGACACTTGATCTGCTTCCTGTCTCTTCTTACTGTGCTTCTTCCTAATTGTCATTCAGGACAAGAAAGCTAATGGTAATCTCTTTGCCACCTTACAACCTTTGTTGCATTGTGCCTCAAAAAAAAAGTTCTCATCTCTCTGTCCCAAAGTTCACTTCTGGAACAGTGTGGCTGTGGCCAGTACGACATACAGTATGTCTAATGTAGTGGCAGTATTCTTTTTTGCCTTTATGTAACAAATAAATGTATCAGTACAGGAGCGGAGTTATTCCCATCGAAGCTCCCTTTCTTCAATACTTAAAACAGTTGGGATCCAGTGCTGATCTCAAAGACCTTCGGAATGGATTACCTAAATCTAAGTCACTTCAAATACCCACACAGCTCAGGGATTTGAACTCTAGAAGTGTCGACTTTATGAAGTGTAATTGTGTCTTGAACAACCATACCTGAAAATTAAAAAAAAAAAAAGCTCTTTCATTCTTTGTTTCTTTCTCTTTCTTTCTCAGTCATTTCCTCTCCAGCTTCAGGATTGATGTCTGGCCAACAAGTCAATCTAAGCTGCAGCCTTGGCCACAATTTGATCTCTAACCTGGAAGTGAAATGGATCCCACCATCATCCCAATCCTCTTTTTCCCATCCCAGATTCTCCCCTCACCCTGCCCATCTCACCATCCCTAATTTAGCTGTTGTAGGAGATCAAGCGAGGTGGAGGTGTGAGTTATGGCAAAACAAATCAAAGCTGACATCCGCAGAAATAACCCTAAAGATAGGTAAGCATTTGCCCCAAGGGGAATTTGTCCTCCTCTTTCAGAGGTTTTCCATTCAACAACAATAGTGCAGTCACAAGAAAAAGTAATACAAATAAACAGTCCATCAGTGCAACCAACAAATCACATCCAATGCACAGTCCATCGTGCGCAACACACAAGACACCAAATCAACGATCCTCCGGTCTGTTAACTACTAGTCCTTGACTGTCCTTGTCCCTCTGTCCCTCTACCCTGCATTCAGAAGTGGCACAGATATATAACACTACAGCCCCTAGTGGTGGGAGTATAATCTTTTTTTTAAAATTATTATTATTATTAACCTTTATTTTACCAGGGAGTCCCATTGAGATACAAGATTACAAGGGCCAAGACAATGGCATCAATATAACAAACAATATATCATACCAAAAAAAACATAAAACATACATAATACAATTCTTTGATTAGATAATTGATTAGGTAATATAATAATAATAATAATAATAATAATAATAATAGATTGTATCTATGGTAAAAGAAAAAGAAATAAAAGATACAAAGACTAAAACCTGGGTGGGCCACTATGAAAATAAAAATTGAATAAAATAAGAAGATGAAGTAAAAAGGAGATAAAAACAAGGGTGGGCCGCTTTAAAAGGCTTAGAAGACCTGCTGATAGAACAAGGTTTTAAAGAGATACTGACATGGCTTTTCTAAGCACAATTCGTGTCAATGTTAATTCCTACTTTTTCAAAACAAAACACAAAGGAGGCGTGATCCATTGCTATTGGCTACTGCTTTCAGAGGGTCGTGTGGGTGTGGCTGG

This utg7180000079533 sequence contains exons for a leader peptide plus complete CD4-1 ectodomain (below), but does not include the essential transmembrane and cytoplasmic domain regions.

The encoded partial sequence is:

MNIFIHCIIIIEAVLSPTSAAVVIVYVYGKVGDNITLPNAETYKGLYVTWHSGIPEEKVLLLQRHKFGSDLFTDDEHWKGRLSLSETNCSLIIKAIKPEDFQPFTCVLKDKERDVSITTYELYEVRVSPGVSEGVPLLLAGESLSLSCTVQTPAGGPQPQIHWLGPKEESVATNRRTQGGNLTVEGVTGRDHGEWTCVLLRNGIESRTKTSVIVVDLSPGHSYPYYLSSSPLQINCSIPLYLTWEHLMKTGLQGGYWNFTPSFPSRAPQKLHSLLAPGKSSTWTTLEHKGAGSMNTLDLVKKNLFPSSEPASEADRGEYTCALEFKGGVTLQRSVRVEVLQVISSPASGLMSGQQVNLSCSLGHNLISNLEVKWIPPSSQSSFSHPRFSPHPAHLTIPNLAVVGDQARWRCELWQNKSKLTSAEITLKI

***Stylephorus chordatus CD74a***

>utg7180003723408 length=1336 num_frags=247 Astat=17.00

(the complementary sequence is shown)

TTAGAAGAGAGTAGCAGAGTTAGAAGAGAGTAGTAGAGTTAAACGGTATTTTCAGCTCACTTGATCAGCTCAAGTTGTCATTCTCTGCACTGCAGGACTGCTTGGCCCCTTCATTGCTGCTTGCAGCTATATTTATTTTTTTATTGTTGTTGTTTTTTTTTTTTGTCCTTGCAGAAACTTGGAGACGTGGCTGTTGTCAGTGTGGAGCAGCACGTGCGCAATCTGCTGAACGTACGTTGCCCCCAATGTCTCCCACGCTATTCCTATCAATTGATGAATGTGTCGTTTCCTGGGAAACATTCTTTTTTTTTTCCTTTTTCTTTTTTATGGCTGCAGAATTCTCAGCTGCCCCACTTCAACGGGACTTTCCAAGCCAACCTGCTGAGTCTGAGAGAGCGGGTGGAGGATAGCCAGTGGAAGGTAATGCTGTGCGCCCACAGAGACGCCAGACATGGGCAGCTGTTTATACTGAGATCTGTAACTCAAACTCAAACTCCGCCTTAGGAGTTTGTGTCCTGGACGCGTCATTGGCTCGTCTTCCAGATGGCCCAGGAGAAGACGCCAACACCCCATGGTAGGCGTCTCTATAACCTTCCCTTCAGCAGTTTATTTACCTCATGTTTCTGGCTGACGCCCCATTACAGCACAGATGCACATTTAATGCCACTGGAGGCTAGCCTTTAATCTTTATCACAGTGAAAGAACAATAAGGTCATCGGGACGAGTGGCGAATGGAATGAGAAGTGAAAGGAGACACCACTTAGTGTATGTAACACTTCCTTCCTCCTCCTGACAAGTCATTCCAGGCCAGATTCACAAATGGGGACTCGGCACTCTTCACATCTGGTATCCCAGAAGGATGAAACCATGCCTGTTTTCTGCTTTGCTTTACGGTGATCCATGTCTATGTTATCCTAACTTCCTCTGCTACCAAACCCCATCCATCCCTCAGAACATCTCCCCCAGACCAAGTGCCAGAAGATTGCAGCCCGTAAAGCTGAGATGGTAGGCACTTTCCAGCCCCAGTGCGATGAGGCGGGCCAATTCATTCCCAGGCAGTGCTGGCACAGTACCGGCTTTTGCTGGTGTGTGGACAAGGAAGGCACCACTATTCCGGGAACCATCATCCGCGGCCGGCCTCAATGTCAGCGAGGTACATGAAGTAACCTATTGTAGAAAATAATTATTAAATAGAGACTCCTGGACAATCATAGAGTGAGGTTTAATTTACGTGATCACGGGTCAAATACAAATTACATAGAGAATGTAATCAGGCAAAAGACAATTGACAAAGGAGAGAAACTACCTTTATACACAGAGAAGAGAAGGGGGATTA

The utg7180003723408 sequence encodes the middle part of CD74a from several exons, but does not have the exons for the N-terminal and C-terminal parts:

10 20 30 40 50 60

TTAGAAGAGAGTAGCAGAGTTAGAAGAGAGTAGTAGAGTTAAACGGTATTTTCAGCTCAC

70 80 90 100 110 120

TTGATCAGCTCAAGTTGTCATTCTCTGCACTGCAGGACTGCTTGGCCCCTTCATTGCTGC

130 140 150 160 170 180

TTGCAGCTATATTTATTTTTTTATTGTTGTTGTTTTTTTTTTTTGTCCTTGCAGAAACTT

K L

190 200 210 220 230 240

GGAGACGTGGCTGTTGTCAGTGTGGAGCAGCACGTGCGCAATCTGCTGAACGTACGTTGC

G D V A V V S V E Q H V R N L L N

250 260 270 280 290 300

CCCCAATGTCTCCCACGCTATTCCTATCAATTGATGAATGTGTCGTTTCCTGGGAAACAT

310 320 330 340 350 360

TCTTTTTTTTTTCCTTTTTCTTTTTTATGGCTGCAGAATTCTCAGCTGCCCCACTTCAAC

N S Q L P H F N

370 380 390 400 410 420

GGGACTTTCCAAGCCAACCTGCTGAGTCTGAGAGAGCGGGTGGAGGATAGCCAGTGGAAG

G T F Q A N L L S L R E R V E D S Q W K

430 440 450 460 470 480

GTAATGCTGTGCGCCCACAGAGACGCCAGACATGGGCAGCTGTTTATACTGAGATCTGTA

490 500 510 520 530 540

ACTCAAACTCAAACTCCGCCTTAGGAGTTTGTGTCCTGGACGCGTCATTGGCTCGTCTTC

E F V S W T R H W L V F

550 560 570 580 590 600

CAGATGGCCCAGGAGAAGACGCCAACACCCCATGGTAGGCGTCTCTATAACCTTCCCTTC

Q M A Q E K T P T P H

610 620 630 640 650 660

AGCAGTTTATTTACCTCATGTTTCTGGCTGACGCCCCATTACAGCACAGATGCACATTTA

670 680 690 700 710 720

ATGCCACTGGAGGCTAGCCTTTAATCTTTATCACAGTGAAAGAACAATAAGGTCATCGGG

730 740 750 760 770 780

ACGAGTGGCGAATGGAATGAGAAGTGAAAGGAGACACCACTTAGTGTATGTAACACTTCC

790 800 810 820 830 840

TTCCTCCTCCTGACAAGTCATTCCAGGCCAGATTCACAAATGGGGACTCGGCACTCTTCA

850 860 870 880 890 900

CATCTGGTATCCCAGAAGGATGAAACCATGCCTGTTTTCTGCTTTGCTTTACGGTGATCC

910 920 930 940 950 960

ATGTCTATGTTATCCTAACTTCCTCTGCTACCAAACCCCATCCATCCCTCAGAACATCTC

E H L

970 980 990 1000 1010 1020

CCCCAGACCAAGTGCCAGAAGATTGCAGCCCGTAAAGCTGAGATGGTAGGCACTTTCCAG

P Q T K C Q K I A A R K A E M V G T F Q

1030 1040 1050 1060 1070 1080

CCCCAGTGCGATGAGGCGGGCCAATTCATTCCCAGGCAGTGCTGGCACAGTACCGGCTTT

P Q C D E A G Q F I P R Q C W H S T G F

1090 1100 1110 1120 1130 1140

TGCTGGTGTGTGGACAAGGAAGGCACCACTATTCCGGGAACCATCATCCGCGGCCGGCCT

C W C V D K E G T T I P G T I I R G R P

1150 1160 1170 1180 1190 1200

CAATGTCAGCGAGGTACATGAAGTAACCTATTGTAGAAAATAATTATTAAATAGAGACTC

Q C Q R

1210 1220 1230 1240 1250 1260

CTGGACAATCATAGAGTGAGGTTTAATTTACGTGATCACGGGTCAAATACAAATTACATA

1270 1280 1290 1300 1310 1320

GAGAATGTAATCAGGCAAAAGACAATTGACAAAGGAGAGAAACTACCTTTATACACAGAG

1330

AAGAGAAGGGGGATTA

***Stylephorus chordatus CD74b***

>utg7180003206387 length=752 num_frags=94 Astat=33.00

CCTCCATAATCCTCCTCTTCTGCCATTAGCTTTTGAAGATGTAACCCAGTGCCAGCTGGAAGCCGCTGGTCTGAAGCTTGTGGATCTGCAAGGGTACCGGCCCGGCTGTGACGATCAGGGACGGTACTTGCCCCAGCAGTGCTGGCAGAGGAAGTGCTGGTGTGTGGACACCGCCACCGGTCAACAAGTCCCCGGGACTATAAGCATCGGACCTGCCCACTGCAGCTCTCTCTCAACCATCATCTCTGGTGGTAAGAAAAAAACGACCCCCGATCACACATCCTGCAATGTCAGCCTGGAATGACATAGAAGCGCTCTTTGCACAATTCCATACGGTGTTCTCCAGATAAAACAAGAAGCGTCTAGTGTCTTGCAATATTGACCGCCGTCTGTGGTTGATGTAGAAATGATGAGAACCCTGAGAAACGCTGGGTCCAATCACAACATCCTGTCCCTTTTATTGTAAACTGGACAAACCTCATCACCCCAGTTTGCTTGTACTTTTTCCTCAGAGCTCATGGGCATAATGGAGGGCCTGGGTGTCATGGAGAAAAGTGGTAAGTATCTGTGCGCTGTGTTATTGTATTTATGGTATTTCATTCAAACTTTACTGCTAGCAATGATTAAAATGTAAAATTCTAATAAATTGGCACCTACAGAAATGAGAAATACAAATTAAGTAAAAATGGAATATTTTTTTGCTGTCTAGTATATTTATTTATATAGCCCTTTATCACTTTTACATTTTCAAA

The sequence utg7180003206387 only contains exons encoding the C-terminal part of CD74b:

10 20 30 40 50 60

CCTCCATAATCCTCCTCTTCTGCCATTAGCTTTTGAAGATGTAACCCAGTGCCAGCTGGA

F E D V T Q C Q L E

70 80 90 100 110 120

AGCCGCTGGTCTGAAGCTTGTGGATCTGCAAGGGTACCGGCCCGGCTGTGACGATCAGGG

A A G L K L V D L Q G Y R P G C D D Q G

130 140 150 160 170 180

ACGGTACTTGCCCCAGCAGTGCTGGCAGAGGAAGTGCTGGTGTGTGGACACCGCCACCGG

R Y L P Q Q C W Q R K C W C V D T A T G

190 200 210 220 230 240

TCAACAAGTCCCCGGGACTATAAGCATCGGACCTGCCCACTGCAGCTCTCTCTCAACCAT

Q Q V P G T I S I G P A H C S S L S T I

250 260 270 280 290 300

CATCTCTGGTGGTAAGAAAAAAACGACCCCCGATCACACATCCTGCAATGTCAGCCTGGA

I S

310 320 330 340 350 360

ATGACATAGAAGCGCTCTTTGCACAATTCCATACGGTGTTCTCCAGATAAAACAAGAAGC

370 380 390 400 410 420

GTCTAGTGTCTTGCAATATTGACCGCCGTCTGTGGTTGATGTAGAAATGATGAGAACCCT

430 440 450 460 470 480

GAGAAACGCTGGGTCCAATCACAACATCCTGTCCCTTTTATTGTAAACTGGACAAACCTC

490 500 510 520 530 540

ATCACCCCAGTTTGCTTGTACTTTTTCCTCAGAGCTCATGGGCATAATGGAGGGCCTGGG

E L M G I M E G L G

550 560 570 580 590 600

TGTCATGGAGAAAAGTGGTAAGTATCTGTGCGCTGTGTTATTGTATTTATGGTATTTCAT

V M E K S

610 620 630 640 650 660

TCAAACTTTACTGCTAGCAATGATTAAAATGTAAAATTCTAATAAATTGGCACCTACAGA

670 680 690 700 710 720

AATGAGAAATACAAATTAAGTAAAAATGGAATATTTTTTTGCTGTCTAGTATATTTATTT

730 740 750

ATATAGCCCTTTATCACTTTTACATTTTCAAA

**3.2 *Cyttopsis roseus***

**For the non-gadiform fish *Cyttopsis roseus* intact full-length MHC class II system genes were reported.**

*Cyttopsis roseus* is a non-gadiform fish investigated by Malmstrøm *et al.*^1^ which is closely related to the gadiform fishes, and hence important for deciding the phylogenetic stage at which functional MHC class II system genes were lost.

Upon inspection, the unitigs listed in their Table S7 appear to encode full-length canonical molecules of MHC IIA, MHC IIB and CD4-1, whereas we were unable to retrieve the unitig listed for CD74a (utg7180001483868).

In summary, our findings for their Table S7 listings are:

**MHC IIA:** Table S7 mentions utg7180000163186. This sequence encodes a full-length canonical classical lineage MHC IIA molecule.

**MHC IIB:** Table S7 mentions utg7180000052012. This sequence encodes a full-length canonical classical lineage MHC IIB molecule. There is an unusual intron within the β2 domain coding sequence which first has been described for neoteleost fish classical MHC IIB genes in pufferfish (Lim *et al.*^3^).

**CD4:** Table S7 mentions utg7180000048768. This sequence encodes a full-length canonical CD4-1 molecule.

**CD74a:** Table S7 mentions utg7180001483868, but we were not able to retrieve that unitig. However, we found that *Cyttopsis roseus* scaffold scf7180003315926 probably encodes a full-length canonical CD74a molecule, although we were unable to predict the exon of the very C-terminus.

**CD74b:** Malmstrøm *et al.*^1^ did not report finding *Cyttopsis roseus* *CD74b* sequences, and we could not find *CD74b* gene(-fragment) containing unitig or scaffold sequences in their datasets either.

Below the respective retrieved scaffold sequences are shown with their relevant protein coding capacity:

***Cyttopsis roseus* *MHC IIA***

>utg7180000163186 length=4473 num_frags=1193 Astat=-3.00

(the complementary sequence is shown)

CAATAAACCTACAAACGAACAATAAACAATAACAATAAAGCTAAACGTTTCTCAGTGTTTGTCTACCTGACGATAGGTGATGCCGAGTCATACTTCAATCTGATAGGTTGATTTTCCAAACAGCGTCTGGACAGAACTCTTACAGTTGGTTCTGTGGCAGTTTCTGTTCCATAAACCCCCAAAAATTAACAACAACAAAAAAACTAAAACAACAATAAAATAACTGAAATAATAAACTAGAAAAATGTGGTCATGTACAATTAATTACATGTACAATCAATGTACAAGAACCCCAATGATACAAATTAAATATAAACCTAAATGTTTACAAAAACAATTAAAAAAATTTTTTTACCTAAAAAACGAAAGCTTAAAAGAGGAAAGCTGGCAAAGAAAATTTTGCAATATACAGTGAATCTAGAAGTTAACTAGAAGATTTGTACAATAATCCTACAAACGAACAATAAACAATAACAATAAAGCTAAACATTTCTCAGTGTTTGTTTACCTGACGATAGGTGATGCCCAGTCATACATAAATCTCATTGGTTGATTTTCCAAACAGCGTCTGGACAGAACTCTCCCAGTTCGTTCTGTGGCAGTTTCTGTTCCATAAACCCCCAAAAATTAACAACAACAAAAAAACTAAAACAACAATAAAATAACTGAAATAATAAACTAGAAAAATGTGGTCATGTACAATTAATTACATGTACAATCAATGTACAAGAACCCTAATGATACACATTAAATATAAACCTAAATGTTTACAAAAAAAAAATAATTTTACCTAAAAAACGAAAGCTTAAAAGAGGAAAGCTGGCAAAGAAAATTTTGCAATATACAGTAAATGTAGAAGTTAACTAGAAGATTTGTACAATAAACCTACAAACGAACAATAAACAATAACAATAAAGCTAAACATTTCTCAGTGTTTGTCTACCTGACGATAGGTGATGCCGAGTCATACTTAAATCCGATTGGTTGATTTTCCAAACAGCGTCTTGACAGAACTCTCCCAGTTCGTTCTGTGGCTTCCATTGACTTTCTATCACTGGATGAAGCTGAGTGGCTCCGAAGGATGAAGATGAAGATGAAGACCTCCCAGACCTTCCTCTTCCTCTGCTACTTGCTGGGGGTCCGGGCAGGCCGTAAGTACAAATGCATTTTCTAATTACATGTATTATTATTTTATTCTATTCTATTTGTTACTCAACTCACTCACCCGGGAGACACTTCCAATCGTTTCTGTCCAATAACCCTCTATTAGACCATCTCATTCTCTTCAGCTGTAAACTTTCCCATGATTTCACCCCTATATAGATAGATTTTATTCATCTAAATAGGCAATAATAATAAGAATAAAGTAATAAGAATAAAACATAGAAAAAAAGAAAATGGCAAAATGCAATAAACCTAAGAAGAAAAATTGGCAATATACAAAAACAGAAGATAGAAAACATGGGAATATACATTAAACAAGAAACAAAGAATAGAAAAGAACCCTAAGATAAACAATGAATAATAAAACAATGGCTATATACAATACCCTAATGATAAACAATACACAATGAATGTAAACCTAAGACACAACAAAAAGCTGGCAAATGTCAATAAAAGTAAACTTGACAGGAGTTTATTAAGCAGATTGTGGTGATGAAACATTGGTATTCTGTATTTATTTGTTTGTTTCTTTGTCTCTGTATCTCCAGCCAGGCATGCAGATGTCAGTATAAGTGGCTGCTCCGACACTGAAGGAGAGCTCATGTACGGTCTGGAGGGTGAGGTGATATCGTACGCAGACTTCCACCTCAAGAAGGGAATGATTGTTTTGCCACCGTTCGCAGACCCCGTGACCCACCCAGGTCTCTACGAGAATGCTCTGGCTCAGCAGCACGTCTGCAGGGAGAACATGAAGGTGCTCCGTGGAGTCTACACCGCCAAGGAACTAGGTGGAGACACACCGTCTGAACTATCCCTCCTCCAGTTGTCTGGTTAATAGTTTGATAACAATAAAGTAATGACTATACTTATGTTTCTCAAATATTACATTTGTGAAGAAAATGGTTTTTTTTAAGACACAAAAAAACACAGTCTGCATTCAAGATCACTTTGGGCATTTGGCATTTGTTTCAACAAAATGTTCTACGCTTCCAATTGAAGCGTAGATGGATGGAGATTTTAGTCAACGATTTCAATGAAGCGTATATCATTTATTAAAATTTTATTATATCATATATATTACATCATAATTATATATTATAATCATTTATATTTTAGGTATATTTTCAAGTTATACATACATTTTCCAAAACAAATCTGTTCAGCCGAGATTTCAATATAGATACCCAATAAGTGTGAAAATCAAAGACTACGCAAATTACATTAAATTTACAACAAACTCTAAGAAGAAGAAGCTTTATTCTGCAGATGTCTTGATTCTGTTTTACTAAATATTAACCAATTATACACATGAACCAAATAACACTAATTAATTCTATAGATTGACTGCATTATTACATATAATTATAGATATTAAATTAAACCTCTATGAACCATATTACTCAACAGATCAGATTATTACAAAAGATGATTTAATGTAGTGAACTGTTGTTTTGCAGATTATCAATCAAAGATTATTGGCAGTAGTGATCAAATTAAAGCTTGTTATGAACCATATTGTTTTCCCAGATTATCAATAATGCATGAAGCCTGACAGTGGTGATTGTGGTGTTCCTCCAGATGTTCCTCACAGCTCCATCTACACCAGAAACCATGTGGCCCTTGGAGTGAAGAACATCCTGATATGTCACGTGACCGGCTTCTACCCGGCGCCCGTCAGAGTCCGCTGGAACAAGAACAACCACAACCTGACGGAAGGCATCACTATCAGCCAGCCATACCCCAACAAAGACGGCACCTTCAACCAGTTCTCCAGCCTCACGTTCACACCGCAAACCAGAGACATTTACAGCTGCACTGTGGAGCACAAGGCTCTGGACGAGCCACTAACTCGAGTTTGGGGTGAGAGCAGAAATCCTACATAGAAATATGACACATACAGTGCATATAGATTATTACACATTACAATTACATTTATTGCACTGCCTGCTACAAAGCAACAGAGCCCTTGAACTCGAGTCTGCCCTAAGAGCTCAAATATTATGCATGATACGCATTATGTGACTAAGAAATATAGCGCTGCTAACTTTATTTTACCCAAAGTAAAATATTTTAATTTACCCAAAGTGCAACAGAAATTAAAACGTCAATGCAATGTTGACTTCTCTGTAACTGACCAGGACAAGACTCCCCCACCCTCCCTTAAATACCCCAAGCCCCGACCACTGGGGTACTATTCAGAAAGGACGGCATCATTGGGACAAAACTCTTCTCTAGAAAGTTATAGCAGCTCTTGTCTATTTATAAGACATTCGTCTATAAATGGAATTTGATTGCCATTGCTATCGACATGGTTGACGTAGCACTGCTTTGTCAGCTCAAGTGACGGTTGGTGCAGTTCATCCGGTTGGTGGTCATGTCAACTTTAGGACTTCGCTCATAGCTACTTCCGTTTAGCGACTAAGGGCTTTTATATAGTAAACATAGATTAGTAAAATTTGTGAACTAACCCTTTAAAGGTCAGAATTAGGAATTTTTAAAATGCAATATTATAATTATGTGTATGTTTCCTCCAGATGTGGAAGAGCAGCAGCCTGGAATAGGACCTGCAGTGTTTTGTGGAGTGGGCCTGACACTCGGTCTGGTGGGCGTGGCTTGCGGGACCTTCTTCCTCATTAAAGGAAACGAATGCAACTAATATTTCCGCTGCTGGCGTTCTGATTGGCTGACTCCTATTTTGGTCCTGTCTGCAGTTGTTAATTCATCTTGCGTTTTGAATAAATAATGAAGTGTTGTTATTAGAGGGCTAATTCATCTTAATTAATGATTGCTAAAAACAATTTTATACCATAATTGATTTATATAGATTGCATAAACTTTATTTTGTTTTGATAAAGATATTTTCATTGAACCCTGACCCCATTTTCCTTTTCCAACCACTGAAGTGATCTGTGTCTAAAATCTAATCCGATGTTTCATCATCTGGGAATATTTTATTATTGTATTTTGTGTATCGTGTACAAATCTAAATAAAGAATTAATCAATATGCACATAATAAGTAATCAATATACCCCCCATGTTTAATGACGCTTTGCTATCCATCTGAAGTGAGAAATTTGTAGCTTCAACTAGAGTTCCTGTTTTGTGTGTGTGTGTGTGTGTGTGTCTGAACTGGGCAGAGTTTCCAATTTGACATTGGTATGAGTAACATTGCTCTCTGAATCAGGTCCCTAGTGGGATGTTCCAGCATAAATACAATAAATAAAATATCAATATACTAACCACATACAATTACATCAAGATATTTAATATACCTGCTACATACAAACTAAATAAAAGGAGGCAGTGTG

This utg7180000163186 sequence includes a full-length intact MHC class IIA gene which encodes:

MKMKMKTSQTFLFLCYLLGVRAGPRHADVSISGCSDTEGELMYGLEGEVISYADFHLKKG

MIVLPPFADPVTHPGLYENALAQQHVCRENMKVLRGVYTAKELDVPHSSIYTRNHVALGV

KNILICHVTGFYPAPVRVRWNKNNHNLTEGITISQPYPNKDGTFNQFSSLTFTPQTRDIY

SCTVEHKALDEPLTRVWDVEEQQPGIGPAVFCGVGLTLGLVGVACGTFFLIKGNECN

***Cyttopsis roseus MHC IIB***

>utg7180000052012 length=4876 num_frags=881 Astat=282.00

AAGGAGGCAGGTTGCTGGGGGGTCAGGAGGCAGGGTGCTGGGGGGTCAGGAGGCAGGGTGCTGGGGGTCAGGAGGCAGGGTGCTGGGGGTCAGGAGGCAGAGCAGACACATTAGAGAACCTCTATTAAAACGGCGCTGCACACTTGCGTTTTTGATCAACGATATCATGTTATTTTGGAGGGTTTTAATACATGATCAGAATGACAAATGAATAGATCCAGGGCTATTTTCTTATTCATCTTATTTTTGGAAGAAAAAAAAATAGATCCTGCTCCGGCTATTCATAAAACATTCAGGGCTGAAGCTCTTAAGGATTTTATTTTATTATGACATTTGAAATATGAGACAATTATGAATAATATATGTATAATGTATAGTATAATATGTAAAATATAATTTGAGTTGACTTGTCCTTTAATTTTCTATTATGGAAACGTTTTTTTTAAATCAAATTAGGACTTATTTGGTGACGTCACATTCAACAAGCGAGTCTTTCGTCCAAGCGTCGGTACCGTAACATCACGGATAGACGCGCATCAAAAATACAAGCGTCGGCTGGCTTGGCTTGACCAGGTTTTCGCACATGACGGCTCACTTGACCGCAGTCTAACGACGCCACTGGCTCTAAGTCTGAATAAATCGTCCAGTTTGCAGCCTCGGTGACGGCTACAACACCGATTTTAAAGTGCAATCATTCTTTGACTACAATATTTTAAACCAACTATCAAATTACAAACGTTTAAATATGAAAGGCTTCATGTTAAACACACTTTGTTTAGTATTTAAGGGATTTAATCTGGGGTCCGCGGCCCCCTGTGCCCCCCTGCCAGGAATTGCCAAATTATTATTATTCTTTTATTTTTATTTTTATTGTTTTCCATGTTTTAATACAGCAATTCATGTAAACAGGCTTGCTTGTCCGAGCAATGCGTTGCAAAGGAATAGGATGCCATGCAATTAAATGCCATTAAATACATTTGGATAAAGAATATTTGGTGTTTTTCTTCTCAATAATAAAATCGTAGTTTGGCTGAATGCACTGGCTTTTAAATCGGAGGAAAACAATTGGGGTAAAATACGAGTACTGTCATATAATAATATGAATCGATTGACGGCCCTAGTCGTTACAAATTGTCCCGTTTCCGTTGTTAAACATGTCATTTAAGGCTCTAGAGTGAGATTCTGGCTCGAGTCTTGTCTCAGCCTGCCCGGCAACAGCTGACGAGGCGACCACGTCACGCCGGTTGTCGCTTCCACATTGGTTTTAAAAGTCGAGTTTCTTAGATTCCTGTCACTTGGCTGTGGAAACTCAACCGGGAAGCAGGGGAACATGAGTCCCGCTCAAGCCTTCCTGCGCTTTTCCTGGATTTTAGTCTTGGCGTGTGGAGCAGGTGGGTCGGAGTCACACATCTTACACCTGCTGTTTGCTTCAGAAACATCGAAATCTGCGGGAATTGCTCGTGTGAAATGGTGATGGTGGAGTACGCGGGGACATTGGAATTAATGAACCTGGTTTGTTTTTTCTTTACTGTCTCAGATGGATTTGAGAACTATGCCATTGGCCGCTGCTTGTTTAACTCGACGGAGCTGCCCGACATAGAGTACATTCGGTCTTATTACTACGACAAGGTAGAGTACATCCGGTTCAACAGCACCGTGGGGAAGTATGTCGGATACACGCAGCTCGGGGTGATGAACGCAGATCGGTGGAACAATGATCCTGCAGAGATGGCCACAATGAGGGGTCAACTGGAGGCTTACTGTCAACATAACATTAAGAACTGGTACGGTGCCGTACTGGGAAAAACAGGTGAGCTCACTAATCACTTTACAAAAGTGTACAAGCAGATGACTTTTTAAGTTTCTTCTTCATTCAAATGTGAATGAAAGAAATGTAGTTCCTACCCTTACATTTAATAACGTGTCCAGAACGGAAAGGTTTATCGTTGTTTTAAGAAGAAGAAGAAGAATGATTTATTATTCACACATTTACCACACACATGCGGAGGGTGACACACACACACACACACATGCAGGAGTGGCGAACGATGAGATACAGTCTCTGCCTTTATCCCATCCTGGCCGTCCTTCCTCCAGGTTCACGTGTTCTTCTTGAATTAAGTTTGACCTCAAGTTAAACGATGGAAGTACCAAATTAAAGGAAATAAGCCAGTTTCCGTGCTTTTGATTCATAACATTTCCCAGAACCTATATAAGTAAAAGTAAACGAGCAGAATATTATTCGCGCTCCTTCGTAACAGCGAACCTCGAGGTCGATCAACTTCGAGAAACGGCACTGGCGCCATCTGGTGGTAAACACCCGCTACTCCGAGTACTTCTCCATTTGCCACTAATATCCGACTAATCATGTGCTTTTCCAGCCACGCCCTACGTGACACTGAGCTCGGTTACGCCCCGTGGTGGCAGCCAAATGGCCATGCTGATGTGCAGTGCCTATGACTTCTACCCAAAACAAATCAGCGTGACCTGGCTGAGAGACGGACGTCCCATCACCACGGAAGTCACTTCCACTAACGAGCAGGCGAACGGCGACTGGTACTACCAGATCCACTCGCAATTGGAGTACACTCCCCGGTCAGTCGGCCGTAATTCGCTCCAACGCCGCCCACTTGAACCCACTCCCTGCACTGACTCCATCAAATGACTCCATCAACTAAAATTTTAGCTTTTACCCGTAATTGTCTTTGCAACACGTAGGATCGCACATCAAATGCAGGTGCATAGCGCCAAACCCTGTGTTATTATTTCCTAAATTCAACTCGACTTCATAAAGTTAAATCCAGTCAAAACATTTTACATACATTTTTTCGGGGACATCGGCGCATGACCATATACTGTATATACAGTGTATATATATAAAATAATACAGTAGGTCTATTATTTATCTTCTAAAGGAATAAAAACACTATGGATTTCACCTTGTGAACAATGCAAATCTATTATTCTCATCAATTATTTTTCTCGATTTAAATTCAGTACAAAAATACGAACATGAATATAGTCTCGTGCGTACAGTGTCTTCAGTGTGGTATTTGTACGAAATGTCAGTAAGATATTACGATCAGTGTGTGGTTTCTATTTCTATGGTTACTAGCATAGCAGGTTGTACCATAGTAGTAGTAACCACCACCCACGCAAATCCCTCCCGCACTGACTGACTCAAATGAACGGGACTCGACGTGGCGACCGAATCTCCCAACATTACAACCAAATTTTAATAACAAAGACCATACATTGACAGTGTCTCACATCATCGCCATCATCAATGCACCGTTGGAATAAATGCAAATCCAAACTTTGAAAGATAAAACCATTTGCATCATTCACATCAGTCTTTTATTGTTTGAATGTTTTATGCGATTCAATTCATTTTAGGTCTGAAGAGAGGATTTCCTGCATGGTGGAACACCCAAGTCTGCAGAGTCCTTTGGTGAAAGACTGGGGTACGGACCCGCCAACCTACTGGATCCATCTGGATCTACCCGTCTCTCCATTTTCCATCTACCTCTCTCTTTGTTTCTCTATTTAGATTTTGAGATTATGGTGAAATTCTTTCAAATCGAAACAAAACGTATCTCTCCATCATTTACTGTGGCCCCATTCCTCTGAGGTTATCATTTTTTATAGCAGCTTCACATTGTAAAATCAGAATTTGCATGGCGGTGCTCTAGTAGTAGTGGTAGTACAGTGCTTGTTGGACTCATGTCTATATTTCTCTGTCTACAGATCCGTCTCTTCCAGAGTCAGACAAGAATAAGATCGCCATCGGAGCATCTGGTCTCATTTTGGGATTGGTGTTGTTTCTGGCTGGATTCATCTACTACAAGAAGAAATCAAGAGGTACCACTTTCTAACCCATTTAAAGCGGTTTAAAGTGTTTATTTTATACACTTTAACACTTTAAGTTACTTCAAACGAACAAAGCACTTTAAATCAGTTAAAAACAGACTAGAATGCATGTCACTACACATAGATACTAAGACTACATATCACTACAGAGATAGAGAATCTGTTTAATGCTTCATGTACTTAAATCAACAAATACATCTCTAATTGACTCTAAATGGTTTGCTTCTTTTTTATTTTTATTGCAGGACGGATTCTGGTACCAAATAACTAAAAATAAGATATTTGCAGTACGGCACCTCACTGGCCGTTTCCAGGTGATCGTGCTGCAGAGGAAGACGTGTTTCAGCCTGAACTCTGATATCTGTGATTTTCTATATTTCTATAATATTGTGTAGGGATGCACCGAAGCTGAAATTCTGGGCCGATACCGATGTTTAGAATTACAATTCGGCTGATAGCCGATGCCGATACCGATGTTTTCCGAGTTCCCGTGAACGCAGCATCACTTGCGACAGAAATTACAGTTTTACGAGTTCCCGTGAACGCAGCATCACTTCCTGCTTGGAGAGCCGTGTCCGCAAGTCCACTTGTTAATCGATGGCGGATGGCGTCGTTCTCTCAGACGGATAAAAAATAAATATAAAAAATGTTAAAATGCGCCCGCCCAAGTAAATTCTATGTGTGGGAAACACTGGATAAACACCAGCCACTACCATCGGTGAACGTCATCTTATTTGTAGATAGCCCGATGGCAGTCAACAAGGCCAATATCGGCCGACACCGATGTTCGGCCGATATATCGGTGCATCCCTAATATTGTGCTTTAGATTCCTAGCTTCCTACACTGTGCTAACAGTTTGAAGACAAGACGCTGATTATACACCAAAGAATTACAATCAATATAGTATATTATAATCAACTTTGCTTGTGTTAAACATAAAGAAGGCTTCCTCTCCTCTCGCTCTCACCGTTTACTATAAGATCAGTCTGCTAGTGTGTGGTGGTG

This utg7180000052012 sequence includes a full-length intact MHC class IIB gene which encodes:

MSPAQAFLRFSWILVLACGADGFENYAIGRCLFNSTELPDIEYIRSYYYDKVEYIRFNST

VGKYVGYTQLGVMNADRWNNDPAEMATMRGQLEAYCQHNIKNWYGAVLGKTATPYVTLSS

VTPRGGSQMAMLMCSAYDFYPKQISVTWLRDGRPITTEVTSTNEQANGDWYYQIHSQLEY

TPRSEERISCMVEHPSLQSPLVKDWDPSLPESDKNKIAIGASGLILGLVLFLAGFIYYKK

KSRGRILVPNN

It probably is interesting for the readers to see the unusual intron within the β2 domain coding sequence. The below figure shows the translated sequence together with the relevant part of utg7180000052012:

1270 1280 1290 1300 1310 1320

GGTTTTAAAAGTCGAGTTTCTTAGATTCCTGTCACTTGGCTGTGGAAACTCAACCGGGAA

1330 1340 1350 1360 1370 1380

GCAGGGGAACATGAGTCCCGCTCAAGCCTTCCTGCGCTTTTCCTGGATTTTAGTCTTGGC

M S P A Q A F L R F S W I L V L A

1390 1400 1410 1420 1430 1440

GTGTGGAGCAGGTGGGTCGGAGTCACACATCTTACACCTGCTGTTTGCTTCAGAAACATC

C G A

1450 1460 1470 1480 1490 1500

GAAATCTGCGGGAATTGCTCGTGTGAAATGGTGATGGTGGAGTACGCGGGGACATTGGAA

1510 1520 1530 1540 1550 1560

TTAATGAACCTGGTTTGTTTTTTCTTTACTGTCTCAGATGGATTTGAGAACTATGCCATT

D G F E N Y A I

1570 1580 1590 1600 1610 1620

GGCCGCTGCTTGTTTAACTCGACGGAGCTGCCCGACATAGAGTACATTCGGTCTTATTAC

G R C L F N S T E L P D I E Y I R S Y Y

1630 1640 1650 1660 1670 1680

TACGACAAGGTAGAGTACATCCGGTTCAACAGCACCGTGGGGAAGTATGTCGGATACACG

Y D K V E Y I R F N S T V G K Y V G Y T

1690 1700 1710 1720 1730 1740

CAGCTCGGGGTGATGAACGCAGATCGGTGGAACAATGATCCTGCAGAGATGGCCACAATG

Q L G V M N A D R W N N D P A E M A T M

1750 1760 1770 1780 1790 1800

AGGGGTCAACTGGAGGCTTACTGTCAACATAACATTAAGAACTGGTACGGTGCCGTACTG

R G Q L E A Y C Q H N I K N W Y G A V L

1810 1820 1830 1840 1850 1860

GGAAAAACAGGTGAGCTCACTAATCACTTTACAAAAGTGTACAAGCAGATGACTTTTTAA

G K T

1870 1880 1890 1900 1910 1920

GTTTCTTCTTCATTCAAATGTGAATGAAAGAAATGTAGTTCCTACCCTTACATTTAATAA

V S S S F K C E * K K C S S Y P Y I * *

F L L H S N V N E R N V V P T L T F N N

F F F I Q M * M K E M * F L P L H L I T

1930 1940 1950 1960 1970 1980

CGTGTCCAGAACGGAAAGGTTTATCGTTGTTTTAAGAAGAAGAAGAAGAATGATTTATTA

R V Q N G K V Y R C F K K K K K N D L L

V S R T E R F I V V L R R R R R M I Y Y

C P E R K G L S L F * E E E E E * F I I

1990 2000 2010 2020 2030 2040

TTCACACATTTACCACACACATGCGGAGGGTGACACACACACACACACACATGCAGGAGT

F T H L P H T C G G * H T H T H T C R S

S H I Y H T H A E G D T H T H T H A G V

H T F T T H M R R V T H T H T H M Q E W

2050 2060 2070 2080 2090 2100

GGCGAACGATGAGATACAGTCTCTGCCTTTATCCCATCCTGGCCGTCCTTCCTCCAGGTT

G E R * D T V S A F I P S W P S F L Q V

A N D E I Q S L P L S H P G R P S S R F

R T M R Y S L C L Y P I L A V L P P G S

2110 2120 2130 2140 2150 2160

CACGTGTTCTTCTTGAATTAAGTTTGACCTCAAGTTAAACGATGGAAGTACCAAATTAAA

2170 2180 2190 2200 2210 2220

GGAAATAAGCCAGTTTCCGTGCTTTTGATTCATAACATTTCCCAGAACCTATATAAGTAA

2230 2240 2250 2260 2270 2280

AAGTAAACGAGCAGAATATTATTCGCGCTCCTTCGTAACAGCGAACCTCGAGGTCGATCA

2290 2300 2310 2320 2330 2340

ACTTCGAGAAACGGCACTGGCGCCATCTGGTGGTAAACACCCGCTACTCCGAGTACTTCT

2350 2360 2370 2380 2390 2400

CCATTTGCCACTAATATCCGACTAATCATGTGCTTTTCCAGCCACGCCCTACGTGACACT

A T P Y V T L

2410 2420 2430 2440 2450 2460

GAGCTCGGTTACGCCCCGTGGTGGCAGCCAAATGGCCATGCTGATGTGCAGTGCCTATGA

S S V T P R G G S Q M A M L M C S A Y D

2470 2480 2490 2500 2510 2520

CTTCTACCCAAAACAAATCAGCGTGACCTGGCTGAGAGACGGACGTCCCATCACCACGGA

F Y P K Q I S V T W L R D G R P I T T E

2530 2540 2550 2560 2570 2580

AGTCACTTCCACTAACGAGCAGGCGAACGGCGACTGGTACTACCAGATCCACTCGCAATT

V T S T N E Q A N G D W Y Y Q I H S Q L

2590 2600 2610 2620 2630 2640

GGAGTACACTCCCCGGTCAGTCGGCCGTAATTCGCTCCAACGCCGCCCACTTGAACCCAC

E Y T P

2650 2660 2670 2680 2690 2700

TCCCTGCACTGACTCCATCAAATGACTCCATCAACTAAAATTTTAGCTTTTACCCGTAAT

2710 2720 2730 2740 2750 2760

TGTCTTTGCAACACGTAGGATCGCACATCAAATGCAGGTGCATAGCGCCAAACCCTGTGT

2770 2780 2790 2800 2810 2820

TATTATTTCCTAAATTCAACTCGACTTCATAAAGTTAAATCCAGTCAAAACATTTTACAT

2830 2840 2850 2860 2870 2880

ACATTTTTTCGGGGACATCGGCGCATGACCATATACTGTATATACAGTGTATATATATAA

2890 2900 2910 2920 2930 2940

AATAATACAGTAGGTCTATTATTTATCTTCTAAAGGAATAAAAACACTATGGATTTCACC

2950 2960 2970 2980 2990 3000

TTGTGAACAATGCAAATCTATTATTCTCATCAATTATTTTTCTCGATTTAAATTCAGTAC

3010 3020 3030 3040 3050 3060

AAAAATACGAACATGAATATAGTCTCGTGCGTACAGTGTCTTCAGTGTGGTATTTGTACG

3070 3080 3090 3100 3110 3120

AAATGTCAGTAAGATATTACGATCAGTGTGTGGTTTCTATTTCTATGGTTACTAGCATAG

3130 3140 3150 3160 3170 3180

CAGGTTGTACCATAGTAGTAGTAACCACCACCCACGCAAATCCCTCCCGCACTGACTGAC

3190 3200 3210 3220 3230 3240

TCAAATGAACGGGACTCGACGTGGCGACCGAATCTCCCAACATTACAACCAAATTTTAAT

3250 3260 3270 3280 3290 3300

AACAAAGACCATACATTGACAGTGTCTCACATCATCGCCATCATCAATGCACCGTTGGAA

3310 3320 3330 3340 3350 3360

TAAATGCAAATCCAAACTTTGAAAGATAAAACCATTTGCATCATTCACATCAGTCTTTTA

3370 3380 3390 3400 3410 3420

TTGTTTGAATGTTTTATGCGATTCAATTCATTTTAGGTCTGAAGAGAGGATTTCCTGCAT

R S E E R I S C M

3430 3440 3450 3460 3470 3480

GGTGGAACACCCAAGTCTGCAGAGTCCTTTGGTGAAAGACTGGGGTACGGACCCGCCAAC

V E H P S L Q S P L V K D W

3490 3500 3510 3520 3530 3540

CTACTGGATCCATCTGGATCTACCCGTCTCTCCATTTTCCATCTACCTCTCTCTTTGTTT

3550 3560 3570 3580 3590 3600

CTCTATTTAGATTTTGAGATTATGGTGAAATTCTTTCAAATCGAAACAAAACGTATCTCT

3610 3620 3630 3640 3650 3660

CCATCATTTACTGTGGCCCCATTCCTCTGAGGTTATCATTTTTTATAGCAGCTTCACATT

3670 3680 3690 3700 3710 3720

GTAAAATCAGAATTTGCATGGCGGTGCTCTAGTAGTAGTGGTAGTACAGTGCTTGTTGGA

3730 3740 3750 3760 3770 3780

CTCATGTCTATATTTCTCTGTCTACAGATCCGTCTCTTCCAGAGTCAGACAAGAATAAGA

D P S L P E S D K N K I

3790 3800 3810 3820 3830 3840

TCGCCATCGGAGCATCTGGTCTCATTTTGGGATTGGTGTTGTTTCTGGCTGGATTCATCT

A I G A S G L I L G L V L F L A G F I Y

3850 3860 3870 3880 3890 3900

ACTACAAGAAGAAATCAAGAGGTACCACTTTCTAACCCATTTAAAGCGGTTTAAAGTGTT

Y K K K S R

3910 3920 3930 3940 3950 3960

TATTTTATACACTTTAACACTTTAAGTTACTTCAAACGAACAAAGCACTTTAAATCAGTT

3970 3980 3990 4000 4010 4020

AAAAACAGACTAGAATGCATGTCACTACACATAGATACTAAGACTACATATCACTACAGA

4030 4040 4050 4060 4070 4080

GATAGAGAATCTGTTTAATGCTTCATGTACTTAAATCAACAAATACATCTCTAATTGACT

4090 4100 4110 4120 4130 4140

CTAAATGGTTTGCTTCTTTTTTATTTTTATTGCAGGACGGATTCTGGTACCAAATAACTA

G R I L V P N N *

4150 4160 4170 4180 4190 4200

AAAATAAGATATTTGCAGTACGGCACCTCACTGGCCGTTTCCAGGTGATCGTGCTGCAGA

***Cyttopsis roseus CD4*** *(CD4-1)*

>utg7180000048768 length=10975 num_frags=2002 Astat=666.00

TCCCTTCGGGCGGGATATATAATATTATTATTATTCTCATATTATTCTCCTACCCATTCTCCGGCCGAAAGTGCCTGGCGCGCAGAAAACGTGGAACCCACGGACACTAAACTAAATTGTATTTGTTGTCCAGATATGCTGTGGTCCAAGTTTGATGAACATCGGACCATATTTGTTGAGTTAGTAAAAAAGAAAATTCTGAAGGAAGTAGCCGAGCGGAGGGACGGGAAGTAGTGGAGCAGAGCGCTTTGTGACATTATCATCCAATTTGGTATATATGTGCAGGGTACGACTCTGAAGGGGCTTTCACATAGGACGTGGTGCCCGATGCGCTCCAAACTCCATCGGTGCGTATTGGTTTCTCAGTTAAGAATGCGCTCCAAGTTTGTATCAGGTCGAGACGAGAGACAGCAGGAAGTAACGTTGCTTTCCATGGGAACTTGGAAAACTGAAATTCGTGTCGGTCATCCCCAGGTCTGTTATCTTGGTGAACTCGCCACTCTCCATGCTCCAGTTTACGTTGTGTTACTAACACAACGTAAACTGGAGCGTTACTTATCGCTTATTTACGTTTCTTTGGCTTGTTTTCACATTGGTGCAATTGCTGCGACTAACCTGGACCCCATGCATTGCTGCCTGCAGCTATATTTATTATTATTATTATTATTATCAAATGTATTTATCTAAATGGGTCATAAAAAAGTGTTTTTAATACAGTTTTTGTTTTTCAATGATGATATATTTCAGAAGTTTGAACGAAATGAAGACTTTGATTTGGTGTCTCATCATCATGAAGGCAGCGCTGAGCTCAGCAGGTACAGTAGACTATTTTTGCAAGAAACAATTTTATTTTGAGCTTTTCTATTCATTTTCTATTCATTTTAAAATTTTAATAAAATGTATGTACACATATAAACAGAAGTTAACTATATTAATATAATATTATGTAACAATATAATGTAATGTAATAATATAGTGTAATAATATAATGTAATAAAATATAAGATATTATTACATTACGTGATTTTTTGGCAGACAGATTGTATTATATTATATTATATTATATTATATTATATTATATTATGTGTATTTTCAGTCCCAGTGTCTGTGGTGATGTATGGCCAGGTGGGAGACGACATCACTCTCCAGAGATCTAAAGTGGGAACAGAAATCGACAAAACCTATGTGACCTGGTCTTTTGGGGAAGAAACAGTGATGAAACGCAACACGTACGGCCGAGAGAGTCCCCCCAGCGGTGTGCACTTCTTTAATAGATGTTAGAGAGTGCAAATATTCATAAGTCATGATATCAAATAAATGAATTAAAATTGAATCAGGTTATCTTTGTAATAATTTTTACAATTTAACCCTTGTCATGCAGTGGGGCCTTGGATAGACAGATTGTCTTTGTCTGGGAGTGATTACTCTCTGACCATCAAGAACATCACACAAGAGGAGTTTGATAAGTTCACCTGCCTTCTGAAGGACAGTTTAGGAAAGATGATTTCCATCACGACGTATGAACTACGCTCAGGTAGGGTCACACATTTGTAAAACTCTAATGTCTGCTTGAAAGTACTTGGCATAGAAACTGAGCTGTTTTAGAAGATTGTAGTAATCCAGGAAAATATTTTTGAAAATGCCTAGAATATGATCATTATTATTTTTTAATTGCCAGTTTTGTCAACACTTTATTTTAACATCTTAATCTATTCTCCTATAATGTAATGATGAATCAGAAAACAATAATGCTTCTTTGTTTTCTTATTTTAAAAAGTTGTAACACACAAGTACATTAATGTGATTACTGCTGAGGTGCGACTCAATTCCAAATATTTATTATAAGACAACAAATTCATTATAATTATCTAACACAAGTGGTTTTCCACCTGGGGGTCCTGGCCCCTCTTAGGAGCCGCCAAAGATCACTGGGGAGGACGCAAGGCCTCACCTGTTCTGAGATTGTCATAAGAAGAATAATGAAACATGTTTTAACTTAAAAAATTAGTTTATAAATCTGTAACCTGTGTTCTACAGTAAATGAGATAGGCCATACCTAACAAACTCCTTCAGGGCCTGTTATTCTGTCATTCTGATGAATTACACTTAGTTTGGTTCTGCTTCTTTTCCCTTACCCGTTTTCCTTGGCGTCCATTTCCTTCATCAGTGCATGTAACACCGGCACCTCGTCTCTTGGCCGGAAAGAACTCGCTGTCCCTGTCCTGCGTCGTGCAGATGTCTCAGAATGGGGCGCGGCCGCAGTTTCGCTGGCTCGACCCACACGGAGAAGACATGGCGAAGGACTCCCGGGGGCAGGGAGCCGCAGGGCAAACTCTGACAGTAAGGCCCGTCACGGGCGGGGACCACGGCCAGTGGACTTGTATCGTGATGCACAACGGAAGAGAAACTCACGCCAAGACAACTGTGACCGTCGTGGGTGAGTTTCTGACAATAAAGATGTGTGATGTCACGCAACAGGACAGTTGTTCATGAATGATTTGAACTTTGTCTTCCCATAGACCTCTCCAAAGGTCTTTCACATCCTTACTATACGTCCGTCTCCTCTGGCCAACTCCGAATCCCCTGCTCCCTCCCTTCTTACCTCTCCTGGGGAGACCTGGTGGAAGCGGGTTTTCGGGGAGGACATTGGAGTTTCACCCCGAGCCGGATCCCCGGGGAAAAGCAGAGGCTCTTCTCGCTCTCTCCGGGGGATCCGCCGACCTGGCAGGCAGACAGCGACAGAGGGCTGAGCGTGTCGGACCTCGGGGAAAACAACCTGTCTCTGCAGAGGGACCGAGTGACGGAGGACGACCGGGGGGAGTACACCTGCGCCCTGGAATTCAAAGATGGAGTAACGCTGAACAGGAGCGTCCGTGTGGAGATGCTACAAAGTAAGTCAACGCAGACAGTAAAGCGCTGTAGCACGTCCAAATCTGTTACTCCAAACACTGAAAGATAGATCAGTTTCCATTTATTTTTCATTAGGTTGTCTTTCTCGTCTTATTTTATTTTCCCCGTTTCCTTGCTCTTTTCTAATTGTCAATCAAAACAAGTCAGACAAGTCGCCCCTGCTCTCCTCTCAGTTGAGGTTTTAACGTGGAACAGGGTCCCTGATGTGACGTGTCTCCGGACTAATTCGGTGGCTGCAGGACTTGTCCGGCTCCATTTGTTTTAATGGGAAATAATGAGCTTCAACACTGCCTGTGGAGCATTGCAACACAATAAATTCAAATTGAATTGTTTCTGTTTATGTTATAAAAAAAATTCTCTGCTTAGTATTCTTTCTTTATTTCTCCTTTATGCAACCAGGGAAATCGATAAAAGCTGAAATTCTAATTACAAGGTTGGTGTGGCGGGAGTTATTCCCGCGGCAGAGAAAGGGCTGCCATACTAAAAAGAGTCATGGTTCAGTGGTTTCATAAAAAGGCATTTTGACTTGAGGAACAGAGAGCTCACCTCAATCGCTTCCAATACCCAGATATGAGTATTGGGATTCTGGTGTACTTTTGTACTTGCTTTTTAACTCTCACGGGGGCCATAGAACGATAGAAAGAGGACTGGAAATCTGAAATGGGTGCACCGGGGTAGGCCTGTTTGAAATGGCACTTTAACCTGTTATCTCCTGTGATTTTATTATGTATTTGTATTTTCTTTTTGTTTTTAGCTTTTTTTATTTCCTATGCTTTTATCCCTCTTGCTTTTCTATAATGTATTTTATTCCTTATGTAAAGCACTTTGAATTGCTGTTGTGTATGAAATGTGCTATGCAAATCAACTTGCAATTCCTACCAATTCCTACCATTTTGGATGAACATAGAAGGTAATAAAAATCTTCCTCTGTTCCTTTCTTTCTTTGCCTCTATAAGTCCTCGCCTCACCGTCTGCAGAAGTCCGCTTTGGCCAACAAGTCAACCTGAGCTGCAGCCTCGGCCACGCCCTGACCTCTGACCTGGAAGTGAAATGGATCCCACCGCGGCAGCCCTCCACCTGGATTCCCCTGGGACCCCCGTCTCACCACCTCACCATCCTGGAAGCGGGCGATAAAGATCAAGGGCGGTGGAGGTGCGAGCTATGGCGAGGCCAAACCAAAGTGACATCAGACCAAACAACGCTGACGATCGGTGAGAGCAGGAAACGAGACGAACAGGAAGTAGAGCATTGGTGTGAACCTGCGGTCGATGCAGTAGAAATTTTAATTTAACATGTAGACATGAAATGTGAAAATTATGTCTTATAGAAATTGTATTATTGTTTTTAATGATTATAACATATATAATATGTTATAATCATTAAACAAGTATGTGCCTACAAGTAACAAAATAATGAAAAGGGGACATATATTATAGGAAATAGGTTTATGGGACGTGATGGCCGGTACAGGATATTACATGGTAACAAACACTGTAACATTGAAATAAGACATAATAGTAAAAGTATATTAAAAAAAAGAAATTATATCTTATTATGGCAACTGTCTGTCTCTGGTTTACAGGGAGAAACCCGATGAGCATTTGGCTTCTGGTGACCATTTGCGCGTCCTCCGTTATTTTATTTCTATTGCTGATCCTGACTGGGATCCTGTTCCAACGACACAGACAGGTACTGGGAAAGATTTAAATCATTGTATCTATACTATTTCCCTCCACTTTGATCCCATAACACTAGGTCGTCAGTACCCTCATCTTCTTTTATCGCTCTTTCTATCTTTCTGTCTGTCTCTCTCTCTCTCTTTCTCTTTCTGACATTAGAGGAAGATGCTGCCGCAACGCCCCAAGCGTAGATTCTGCCGCTGCACAAAGTGAGTGTCACGTCTCAAATCACGATCAAATAACTCCACAGATCCGCGGTGTTATGTATAGTATCCCGAGCACAAATCTCTTTTATCTTCAGACTTACCTGCTTCTTTTATTTCTCGAGTTCTGTCTCACGTCTCGCTATTTGTGTCCTGCGTGTTTTTTTAGTTTTTTTTTTTATGTTTCAGCCCCAAACCAAAGGGATTCTACAGAACGTAGGATCTTCTGAGCCGTTGGAAGATATTTTCAAGAGGACCTCTCACCCAGACGAATTCTCAGTCCTAATATAGCGTGTGATGTCTCCGTCGTAGCGACTGATCCGTGTCCCTCTGTCAAGCTTTTTAAGATGACAAACTCAAAGGCGGAAAAGGACTTGCGCTGTTGAACCTTTTGCTCTTGTATCGTTGTTGTTTTTTATTGTATCAGAACTGTACTCTGCGAAGATCTTCCTTTTAAAACATATCTTTATACTGGCACGGCTGTATCATCATCATTGTATTAAGCGTCGCAGAAACTTTATTTTTCTGGTTTTGTCAGCTGACGGCTATTTGAGTGTTAGATTCTACTTCATCTCAAGGAGTGGGATATTTTGCATTTTATGGTCTGCTTGGAGCCAAATGTATTATGTGTAATATGTTTAAACTATATGATTCTTTGTAAAACTTGGTTGAAAGCCTGGAAATTCTAAATATTTTATAATGTTTTGATCTACTTTGAATAAATAAAATGTGTGATACACACCACACTTTCTTGCATATCTAAACGAGTACATCTATTCAGAACTGGCATAATTACAACGATTCTTCAGTATCACAGAAGTGGCAGGCACCAGATGTAATATAGATATTGTCTACCTGGCAGGTGGCTGAAGGGATGCTGTGTTGGAATACTAAAACCATCAAAATCAAAATTTAAGATCAGAATCTGAAATTGTACCTAGTTGTAGGCCACCGTGGGTACTGCCATTGATGAAAATAAAAGATTTTATATAATGAAAATTGATTTTTCTTTTGTAAATTTAAGCTGAAAATGGGAGCTTATGTCCCTCTTTATGTCTACATGAATGTTTTTGTTTTGGTGAATTGTAAAAAGCTTTTTTTTTAATTAAAGATGAAGAATAAAAATAATGAGGGTGTATGGCAGGGAGACACCCTGAATGGGATGCTGGTATATTCCAGTCTCACCAGAGACACACTTCAGATTTAGGGGAAACTAAGGGTCTCCAATTAGCTGAACCTCCGTGTCCAAACCAGAGAGAGGGAAGCCACAAAAAAACAAAACAAAGAAAAACGGGAAGAATCACAGGCAGACATTACACAACAATGGCTCCTTTTCTTTCTAGGTCATCCTAAAAAGGGGATTAGAGTCAAACTGAAAACAGCTAGTATGTATTTGTATGTGTTAAATGCATATCTATCTATCTATCTATCTATCTATCTATCTATCTATCTATCTATCTATCTATCTATCTATCTATCTATCTATCTATCTATCTATCTGTCTATCTCTCTACTCTGTAGGAAGGAATCTGAAGGAATCTGTCTGTATGTCCTTCAATTTTTGTCCTTCAATTATCTTGACAACCGTTCATCCGATCGTCTTCACACTTGGTGGGTGTATTGCTAGCCACCGAAGGACTTCAGTGTTGAATTTGGTGCAATTTGGACACACGACACGTGTAATATAAATTGTGGTGTGGGGGTGGTGTGATTTAAGCAGCCGCACCTGTCTTGGATCTAGTTAATTAGGCCCGGGATCAGGGGTGGCTGTGCACCCGTTGCTCATCAGAGCAATCGATGAGCACGGGTTAAATCACCCATCCCACATATTTGGGGGGGGCTTTTGTCATGACACCATTCCATCTGCTCCTGGCGTGTTACATCTTGCCAATAAACCCGGGTTGTCCTCACCATCTTCGCCTGTGTCACTGTGTGGGCGAGCCCCTAGAAAAGCCACTCGGGTGGCATGTGGCAGGCGCAACTGCCACATAAATAAACTTTGAACAGCGAGCCAGCGGGGGCGGGGGATTGGAAACCTGCAAGCAGCAATACCGGAGGCCAAGCAATCGGCCTGTTCTGAACAGGCATGTTTTCAATGGGCACTGCACTAGTACATACAATCACACAAACACAGAGGCATACATTTGTTGCATAAATGTTATTGTTTGTTATTTCTGTAAATTTGTGTCAATTTGCTCATCCAGATTGTATTGATTCTTGTGTTTGCTTCTCTTAAAAAATGTTTTTTCAGGATTAGGTCATGGTGGTATGAAGTTGGTGGAATGTATGTTGCAGCTAAACTCATGTAGAAAATAAAATGTTTCTCAGGGGTTCAGTCTTATAAAGCACAAGAGTGGGGTCTCACCGTTTTGGCTGAGAGGCCTGAAAAGCATGACTCAGCAGCAGTTTGTGTACTGTTTTGATCAGCTGCGACAATATAACACACACACACACATAGAGAAACACTGCTATTTCCCCATCAGGCTATTGTCTAACTTCCAGTTTTTTTGTGGACGTTTTCTATTCATGTGAAGAAGTCCAAAGTTTTTCCTGCTCCCTTGCCACCCACGGAATGCTCTGCATCACCCCACGGTCCTCGCCAGCCTCGGGACTCTCTCCAAACCGGAGAGCGAAGTTGATGCAATACTGAGACAGCAACACACAAAGACAGATTTTAGGCTAGCTACGGTAAACTAGTTTGAGATACAACAGGAAACGAGGTGGTACTTGCGGAGTGAAGAGGGATTCCTAACGAGACGCTTAAAGAGAGGCTTAAAGAGTCAAATTATTTGAGGAAAGAGCGATAAGGAGGAAACTTGTGCGATGGAGCCCAGAGAGAGGGAGACCTACACCAGTCTCCAAGAGTTCATTGAAGACACTTCCAACAGGGGGCACAGGCCGTCTTCAACTACATGTACAACCAGGGTATTGAGTGTGTGTGTGTGTGTATTTTGTATGTGTGTGAGATGAGTTCATATTATTGTATACATATCTCTAGTAATATTCACTAACTACCTACACCTACGTTCAGTAGTCGCAGTGACCATACCGCCGTAATCCCTGCCTTGTCATTCATCTTGCGCATAGAGCACAATCTAGATTGTTACGCTGTTTTTGAGGTTACTTGTCTGATTGTGTACAACACTGCACCAACTCCTACCAAGACAAATTCCTCGTACATGAAAATGGGCTTGGCCTAATCCGACCGAATCCGATCCATGTTGTGAGCCTACGCAACATGGCGATGGCGGTTTTAACATCATGCACATACGTGTGTATATACATACATGCATTGCATGTGTCTGATTCTGTCTGGTGTATATAAATGCATGTCTGCGTATCAGTGGATAAACAGAGAGAGAGCATCTGCTTGACCTGATTGCTGTTCCGTGTTATCGTGTTATCTAATGTGTGTAGAGCAAGCCCATGTTTTTTTTTTACTACACTATCTTGTTCTATCAATCATCCCTACTACTACGCTACGTTGGAGAATGACAATGAAACATTGACAGCGAGCGTTGTGTGTTCTGTCTAATCAGGCTCTCTGGGACTGAAGAGAGGCGTGGAGTGTTTGAGGAGCCCGGCAGCTCTTGTTGTACTCGTCGGCCTCCTGGCTTCCATTTGTGCCAACATCGCCCTGACCGTTCTACGCAAGTAAAGTAAACACTGGCTGTCTGACCGCCGGCCTGTCTGTCTGACCGCCTGCCTGTCTGTCGGACCATATTAGCTTTTAGTCTTCTGCACTATTCCCCTTAATTATTTTCTCTTTATTTCTGCAAAGTCTGCATTTGGACGCCTATAATTGGAAAAAAAATAAAAAGATAGAACACCTTGTCTAGTCCAGTGGGGGTCGTATTCTTGTAACAGAGGAGCACAAAAGTCCATAATTGTGAAATATTTCTCCTGTAGGACCCAGAGTCTGTGGCAGCCAGGTCTGATAACGGGCTGTAGGGAGGTGTTCTCATTTAGCACAAACACCACCGGAGGCTGTCCTTGCACAATCTACCCCCCACCACCTCGCGACACACACACACACAGATACACACTCGTACACATGCACAGTGCTTGTGTACGATGGAGGATGTGAGGAATTATGCTTCACATTAGATGACCCAGAATTGTGGTTTATTAAAAAAAAAATTAAAAACACTATCTATGAAGATTGTATCTACAATGCACACTACATTTGAAAGGCTCTCTACGGAGACATGACGCACATTTGGGATGTTTTGGATGTGATGGAATGGGCCAATGACAACGACACAGGGGGAAAACCTAAACAAACCAGGTATACGTATTTCATATGAAATTTACCTCAGATTATGACACAGTTTACTTTAACTCCTACTTTTTATAATATGAAAATTAGAAAGCAGTATCTTAGCAGATGGTAAAGGGAATAGCAGATCACCCACAAAAGCAGATGAGATCCATTTTAGAAAATGATTTAGAATTAAATTCATTATGTAGAATCTTGTTTTGAAACCACTGAGGGGATTAAGTATAAGCTGATGTATTATGATTCATGTCTGGTTGCGTTCCAGCAGCGGCAGCATCACGCAGGGATGCATTGTTCTGAGAAGCACCATATGTTTAGTACGGAGGAGAAAGACGACGCTTAAAGAGAAATAACAAAAGCACCAAATATATACCCTCTGTACATGTACTGTAACATCTCTCTCTCTCTCTCTCTCTCTCTCTCTCTCTCACTAAAATGTGGTCAGTGTTTAGCAGGCCCACGCCAGGAACCCCCCTGGACTCTGCAGCGCTGGCCTTGAAGCTCAACTCGGCACAGAGACGCTACATCCAGCTGTGTGCAGACTACACTGACTTGGCGCAGACCTCACTAAAGGTACGGTGATATACTGTATGCCGTCGTAATATTCTCTTCGCTCCATGTATGCCATTGTGCCGACTTGTTCTTTTTTTCCCGTTTCTCCACAGTCAAAAAGTGTAGGGAACGTCCTGAGGGGTGGCTCCATTATAGGTGATAAATGCCATTACTTCAGTGAGGTCGAGCTGGACTGGCCGAGGGGCAGAGACAGTTGCACAAAAATGGGGAGTCACCTCACCATCCTGCACACCGTGGAACAGCATGTTAGTCTGCACTTATAAGTGGGCGGAGGATCACTTTCCATTCAATTGCATAGAGAAATAGCTCAAATATACCAGATGTACAAAAATGGAGAAAGGAAGAGAGAGAAAGAAGGGTCGAGGGTTCACGCGAGGCGGTTGCAAGATTTTCCGTGGCAGTTTTTCACCCGACAAGACTCCAGAGCCGTCTTTCAACTCAAAGATTCGCCGACCGACATTCACAGTCAATGCGAGTGATCCTCCCAATATGGCGGCTCGCTCCAGCGATGCGAGTGTCAGCGCCGCTTTCCCCCATTCGCCACAGACACTTTGAACGTATATTGTGATAGAGCCACTGGTAGATGGAGGGAAGAGATTAGGGTTTGACAGAAGTTGCAGACAGAAATTGAATCAACATCACCGCGAGGTGGCCCTGTTTCCTAAATGATGCATTCATGTCACATGAGGAAATGGTAACTACGAGCTTCCAAACTGGAAATCCTCTTTACGCCGGCCTTCAAGTTGTAAATTATCATTGTCATCGCGTATTTTTACTTTATACGGTTTTTTCGAGTTCGAGTTTTTTGTTGTTGTTGTTGTTTTTTTAAACTATTTTTTTGCATGTGTCATCACCTACAATAGCGCACTGTCGTGGCAACAAAATTATAATAAAAAAAAAATAATAATAACCTAGTGTATACTACGTTGTAAACCGGCCTACGTATTTGATAGCACTTTACTATACTATTAGATTTTATTAGATAAATGCGAAATTGTGGTGTCACAGCAGCACACATCAGAACAAATATGTTTCGATTGGGCGATCACAGTAAAGTAATTAGAAATGGAAATAGAAATGATTTGGCCCAATGCACAGCATTCCACATAGAAAATGGAAATGGAAATATACAATAAACGATACCCTAAAAATAAACAAAGTAGAACAGTAAATGGTAACAAGTGAGAAGAAATTGTAATAAGTAAGTTCGTAAGAAGTAATTACAGATAGAAATCTTGCCCTATTTAATAATAACATTTAAAAAAAAAACAACAACCCGTCAAACCCGCGAACAAGTCGAGCGAGCGCGTCGTGTAAAACTCCCACCCGAA

This utg7180000048768 sequence includes a full-length intact CD4-1 gene which encodes:

MKTLIWCLIIMKAALSSAVPVSVVMYGQVGDDITLQRSKVGTEIDKTYVTWSFGEETVMK

RNTYGRESPPSVGPWIDRLSLSGSDYSLTIKNITQEEFDKFTCLLKDSLGKMISITTYEL

RSVHVTPAPRLLAGKNSLSLSCVVQMSQNGARPQFRWLDPHGEDMAKDSRGQGAAGQTLT

VRPVTGGDHGQWTCIVMHNGRETHAKTTVTVVDLSKGLSHPYYTSVSSGQLRIPCSLPSY

LSWGDLVEAGFRGGHWSFTPSRIPGEKQRLFSLSPGDPPTWQADSDRGLSVSDLGENNLS

LQRDRVTEDDRGEYTCALEFKDGVTLNRSVRVEMLQKVRFGQQVNLSCSLGHALTSDLEV

KWIPPRQPSTWIPLGPPSHHLTILEAGDKDQGRWRCELWRGQTKVTSDQTTLTIGRNPMS

IWLLVTICASSVILFLLLILTGILFQRHRQRKMLPQRPKRRFCRCTNPKPKGFYRT

***Cyttopsis roseus CD74a***

Malmstrøm *et* *al.*^1^ list utg7180001483868 as encoding CD74a, but we were unable to retrieve that unitig from their dataset. However, we could retrieve their reported scaffold scf7180003315926, which probably encodes a full-length canonical CD74a molecule.

>scf7180003315926 length=8863

(the complementary sequence is shown)

GAGCGCCGAGCGACAGCCAATAGGAGGACGGATACGCTGTCCCTGGCGCTGATCCCTGGAAAACGGACGCCCGGGCCGCTGCCTATTGATCATGATTAGGCTGTTTTTTTAAATGAAAAACGTCCCAAAAACGATCGATAAAATAGGGAACACGTCAGTTAATGTTTCTCCAACAAGGAAAATTCTTTATTTTAACATATGAATGGCAAGATTATTTACGTTTTTTTTTTAATGTCAAATACAAGTGGTTTGTAATATATACTTAAATTATATTTATTTTATTATTATTATTATTATTATTATTATTATTATTTCTTTTTATTATTCGTATTAGAATAACTATTAGTATTAGAATAATTATTAGTATTAGTATTAGAATAATTATTAGTATTAGTATTAGAATAATTATTAGTATTGGTATTTGAATAAGTATTGGTAGATAGAATTAGTGTTATTTAAAAACATTTAAATAAAAGGCTTCTCATGTCAGGGGGGAGTCAACACATTTTTTATAATTGTCCAATAATTCGACTTTATTTTTGTATTTAATCAGTTTTAACATTTCAAATAAGGAAATCGATACATAATATTTAGGAGACGTTTCCATGTTTTCTGCCTCTAACAACAAGTGACCGCGGCTCCGTTCAGCCAATCAGAGCGGCGCGTCCACAGCACCTGAATATAAGGGACTTCCCGGTCCTCTGCGTTTTCACATCGTTTCTCCACGAAACCTGCGTTGGTCCAGATTCCTTGTGCGGATTATTCTGCTCATCGTTCCCTCCAAACCGACAAAGTTCAACCAAGAGATGCAGTCACCGGAGGAAGGCCCGCTGACCAACAGTCGGGAGGCCCTCGTCGATTCAGTCAGAGTGAGAGGGTGAGAAATTAAAAAGAAAAAAAAAAATATTATATTATTAGACAAAAAAAAAAAAAAATTTTTTAGACGGTATAAACACAACTTAAGACAACATAAATGTGTTTATTTAAATTTATACAATTAAAAATAACTATTTCAATCGTTAGTCGTATGTTCGTATAATCTCGTCTCAACTTTGAGAACAGGTCTGCCTAGCAACTAGTGACGCCTGTTTTTTTCATTGGTCCACAGGGAGCCATCCAGAAAAAAAAACAAAAAAGAAAACACCACTTAAGGCCTTAAGGCCCAGAAATATTTGTTGTAGGTTTTTTTTTTGTGTTTTTGTTTTAAGCGTGTTGTGGATGGTTATATGAGTGAGTTTAAGTAAGATGGTTCACATTATAGCCTACTGCTGTGGACTTCCCTATTTCTTTATTATTTCCATAATTTTTTTTAAATGCCTCTATAAACATTTTTTTTTCTTTTTTTTCTAGATCTTTTTGTTTGTTTATTTGTATAGGTGTCTTTTTTCTTCTTTTTTTGTTTGTTTATAATTGTCTATTTTAGTGACTTAATGTTTTTCATTTTTTAATTCCCTGCGATCAGGGGTTCCAACAGCCGTGCCTTCAAGATAGCAGGACTCACCACGCTGGGATGTCTGCTGCTGGCCAGCCAGGTATTCACGGTCTACCTGGTGGTCAACCAGAAGCAGGAGATCCACACGCTGCAGCAAGGCTCCGAAGACTTAAAAAAGCAGCTGCTGGTCCGAAGCTCCCAGCGTAAGAAATAATGTAATAACACAGTAATAACATAGGATTGGTGAGAAATTGAGATTAAAAAAAACAAACTCTCAGTGTCTCCGGAATGTAGGCGGCAGAAACGAAAGAGGAAATCAACAGGATTGTGATGCAGTGACACAAGCGAACACAAAGAGAAAGGACTTTTACAATCGGAGCGTCTGTAGTGAATTAAAAGAGTTAACCAAATCAAATAATTGTCCTTTGCAGCCCAGATGAAGATGTACATGCCGATGCCCGGCATGTCTCTTCTCAAGGGTTTCCAGAATGAGGATGAAACTGCCGAGACATCGGTGAAGGTGGGTCTAAACCTTCAAGTTCAGTAAAGGTGATGTGTAGACAACAGTTATTTAAAGTCTTAGAATACATGTTTTTCTATGTAATTTATAATATGCGGTCGTTCCCTCCAGAATGCGGAATACACGCCCCCCCCCAGTGTGGAGACGCAGATGGAGTATCTCCTACAGGTGCGTCACATTTCAAATCCTGACTACGTCTCACTAGTATTTGTGCCCGGTACTTCTGGGTGTGTCCTTACTAAGCCAACAAAAACTTTTTCACCAGGATTCCCAGCTGCCCCATTTCAACACCTCCACCTTCCTTGACAACCTGCAGAGCCTGAAGGCACAGCTGGAGGAGAGCGAGTGGAAGGTAAAGTCACGCGCACGCGCGCACACACACGTACACACTCTAAGCCGTCCTATAGGTTGTGTGTCGCCTCTGTATCTCAACCTGATGTTCTTCTCAGGGGTTGGAGTCCTGGATGCGTCACTGGCTGATCTTCCAGAAGGCCCAGGAGAAGCCGCCCGCATCCGAGACTGAGAACCCAGGTGTGCGGAGACTCCGCCCGTCCCTGCAACATATTTAAATTGAAATCGGAGGCAAATAACTGAATTCAAACTTTTGAGTGAATATGTGTCTCCAACTTCAAGTCGTTCTGGTCATAAGGTGCATGGCGGTGAAAGCCAGCTAAGAACTAACCAATCCTGAGATCCGGTGTTATGGTAGTTTATACTGTAACTCCATAGATCTTAGATATCTGCAAGGCCTTGTATATATTAAAGTAAGTAAGTAAGTAAGGTGGCATAACACAAAACACATTTGAAGAAAATTTTGGCCACAAACAAAGGTAACACTTAACAAAAGCCTATAAAAAGTATAAAATGCAGAGAAAAACATTTGAACACAAACACATTTGAACAAATAGGACATAAAACCCCAAGATAAGGCTTTATAAAAAGGCTAACCTACAAAAATCTGAAAATAAACTGGTGTGATAACATTATATGAATATACAGTACAANNNNNNNNNNNNNNNNNNNNAAAAAAGGTCTTTCGATAGTTTGTAGTAGTAGTCGTGCGTATAATCAGATCTAAACTTTGAGATCAAGTCTGCCTAGCAACTAGTGATGCCTTCATTGGTTCACAGGGAATCCTCCAGAAGAAAAAAAACAAAAACAATTAGTTAACAATGAGGCCCCCCACTTTTTTCTCCTGTTTATAAGTGTATTGGGGATCAGAAAAGGCAGTTCAAATCAATCAGACAATCAATCACACTTTATTGATACAGCATGTTTTGCACATTATAATGCAGTGCAACATTCCATGGGCACTTTCTCCTCAGCCCAAACCCAGTCCAAGTGTCAGATGGAGGCATCTGTGAAGACCCTTGGCTCCTTCAGGCCACAGTGCGATGACACCGGCCGCTACTTGCCCATGCAGTGCTGGCACAGCACCGGCTACTGCTGGTGCGTGGACGAGAGCGGCACGGCCGCCGAGGGCAGCCACACCCGCGGAAAACTCAGTTGTCAGAAAGGTACGCGTCTCAGTTGTGTCTTTTAGCTTTCCAAAATGAAAGCGTCAAAATAAAAGCGGCGGGTTTATTTATTTAAATTTTTAACGCTTGCATTGCTTTGTTGAGGTACCTGGCCAGGCGGTCTCTCCAAGACACTGATCTCATAGATGCCGCCGCTGGAAACCATCAGAGATGACTGTGAGTAGTGTCACACTCATGATGCGTGGATTTTCGTCTTGTTGGTGACATTTTAAGTTCAATACGATAACGTCCGATTCGTTTTCTTGTTTTTTTTTTTTTTCCAGGGAACTGAGAGAGCTTCATTCCAGAGTGGCAGGCATCTGTCTCCGTGAGCAATATTTCGGGACACATCTGAATAAATCTGATTCTTCATGCAGCTCTCATTTATTGCAATATATTATCTGCGTCTATAACTGATCTTTAATCATATTCTCTGTGTCAAATTAATAACTTTTAGTTTAAGGCTCGGCAACTCCTCGAATGTAATCAAGAAATAGAGGCATGTTACGTTTTGCTGTTGTTTGATTTGATTGTTGTTGAAATATGAAGCTAGAACTGGTTTGCCTAGTCTCTGAAGCATATATCAAGATTGCAAGTTATTCCTTACAGGGTTAGTATGCTTTCCCCTATTAATAACAATGTTATTAACAATTAATGCTCAAATACATACATGGTTTTTCAGTTCCTACATGACTTAAAAATGAATAAAGTTACAACTGATTACAGATGAAATTTTCTGTTTCACTTACTCTTCCGCCACCTTTAATGTTTATTTTGGTATAGATTCTTTATGAAGAATATCAACTATATCCGATAGATTAAAATTTTATACAAAAAACTTGTAAATGGCTTGAAGCTTATCTGTAACACTAAAATGCAGTGAGAGAATAAATGTATCAACTTTGTATCTGTCTGTAGAGTTATACAATTGTCGTATAATTTTAGAGATATTTCCCTACATATATGATTTTGTATCAAGCGACCGGCCGACAACAACAAAATGTTTCATGAATAAAATGTATTTAATCCGGTAGTGTGATTAAGATTGAGACCAAAATCTCTTTTGCAGCAGCGACCTGAGAACAAAGAACAAATAATAATAAATAGAAAAGTAAGTAAGTAAATAAATAAAATACGTAAAAAAAATACAATTATGAAACAATGTAAACATGAATGGCAGGAGTTAAAAAATAAATACATCTAGATTTCAAGAATCTACTCACAATGGCCATAATGTATCCTAAACCCTATGACCTGTTGCCATCCAATACAACATATTGAAAATCTTTAGGAAAAGAATTATCATCTTTTAAATTCTATTCCCAAAGGATGCGTCAGGTAACTGTACCCCCATTTCTGAGTGAAAGTGAAACCCGAGCTCATCAATGATTCAGATCTGCGAAACCCAGAAGTCGTGCTGGACAGGCACGAAATGGCCACACAGTCTTTGACTCAATGAACATGGACATGGATGTAAATGTAAGTGGAACTACTTTGCAGTTTAAGTAGAGATATCTTTAAGTCGTATAGTATTTAAGTAATTACATTTTAGACGCTTGATGATTCCAGTCTTGTGTCCTGTGATTTCGGGTGGGTTTTTCTTTGTTGGTACAGGCGATATCAGGTGCAGCTGCACCAGTAAGAGTGGGAGTTGTACTCATGAAAATCCCAGGTAATGTCACATTAACTCCACCTTTTTCCATATCGAGGACCCTCAATTTGATACGCATTAGGCCACGGACCCCATTTGATGAGATTTTGTCTCTGGGACCCCGATCTGAGAAGATTTGAGAACCTCCCCTGGAACCTCTTGAACCCCCTTCCCTTCCATTAAAAGCCCCAGCCAACATTTGTCTTTGTCACACAGGAGGAAGTGACTTTAACATAGAAGACGGAACCGAAAGTAAAAAGACCTTTAACAGAGAAAAAAAAAAAAAAAGGGGGGGGGAAGGGGAAGAAGGAAGAATCATTTACTGCGTTTTTATACTATGCTTCTTGTAAAATTCTAGTTTATTTTTAGATCCTAGACATTCACATCAGAAACTATTACTATATATTTAACTTCCTACATGTGTGCTTTCAGTATGTCTAGTGAATTCAAAAGTAGACAAATCAAATATTCCCAGGTTAAGTGAGGTCATCCTAAATCCGCTCATTTTGATCACGAATCCCTCTGCGCTGTCACCTCCGGGCCTCATTGTGCTCCGTCTGTCATGTCGACAGCCGTGGGTGGCGGTCGTAGCTGTGCAATTCATGAGGACGGCCGTCGTGTGGACTACACGTCGGGAAAGAATGAGGTACTGTATTGGAAACGGCAACAATCTGTATTCTGACTCGAATCGCCCCATTTCTCAAGTGTTTTCACGTCTTTTCCAACCAGGGGACGACTTCTTTCTCATTCGAGGGAGCTGTGACGGTGGATAACCTAGAGGTACCCGGCAAATTTCGATCAGGATGATTTATGGCAATCCAACTGTCCGGTCGAGTTTTGGAAGTTTTTAAAAACACAGAATTCTGGGATGTTTTGCGTTTCCTGCCGTTCTAAATATGCAGAAATTAGAGCCAGCGTTCCTCGAGCCCCTGGCTGAGTCCGTGTCCTGCGGCTACACCGCAACACTGCTGGTCAGCGGAGTCCACAGAACAAGGAATTCGGGCGCCTTGATAGACGATGACATCGTCAAAAAGGTCAGGTTTGGGGGGGGTGGAGTCAAAAATCAGAGCTTCGGCAGACTTATTCATGGGCCAACTCCTTTTACAGATTCTGGGGATTTTATTCAGTCGCGTAGCAGAAATGAAAGAGGGAATGTTCACCGCCGTGTCGTTCGTCCAGGTAGCCTACGAGCTTCTGCAGCACAGTGCATCAACGGGACCTGGTGTGCACCCGGGGTTCTAGACTAATATTCATACCTTTACTTGTCGGTAGTTCTTCCCGGACGACAACGCGCTGGATCTCCTGAACCCTGAAAGGCGTCAACTAAAACCCGTCTCTCACCCGGTCCTCGGATGGTATGTCCCAATAACGTCCTCCAAGCATCCCTTCGTCGATAAGCCAGTAACATGGACACCACTCATTCATGCGAGCCAGGTTCCTCCTTGATTGCTCATGGCCCCTTGACCGTGAGCGGGTGAAGAAGATGGATGGATGAGAAAGAATGAGATGTTTGAATGATCCCAATACAACTACTGCTCTTTTTCAGCCTTGTGGAAGGGTTGTGCGAGGTGTCCGTGGGCTCGGCGGAAGAGGCCTGCGGTCTGTATGAGACGGGCAGGCGGAGGATGGGAGGCACGGCAGCCGGCAGGTAGGACATCGGTCGGAAAGGCCGTGTAACTCCGCGAACTCTGTTACTTTGACCGAAGTAATTACAACGGTCGACTCATTCGCCGCCAGCTCTTTCACCGCAGATTCAGTCAATCGTGATCTATCACTGGTGTCCACATGCCTGCCCGCGTACTTTCCTTTAGATGCGGTTCCCTGTTCTCAGTCGCCGTTGAACAAAGGCTCCATCCAGAGGACGACGAGCCTGAATTCTGTCGTAGCAGACTACAGCTGTTTAGCTTAGCGGGGGCGGCGAACAAGACTGGCCTCCAAGGGTAAGTCACATCGATATCTATTGTTATGAGCAGGTTTTCTCTGATAATTCACAAAAAAATTTTTTTTATCAATAACGGTACCCAATACCAATAACCTGTACCTTGGTGAATAACCTGACTTGTAGCAAAACAGCAAATTTTGTTAAATGTTGACGCCAACTACTATATATTCCTTAGTGTGAGCCCACTGGTTAAGGTTCTAGAGCAAATTCAACTTGGAACCACGACCAGCACCAAAGTCCTTCCCTTCCTATTAAAGGAAGCATTGACGGGAAACAGCAGGACGTGTCTCCTTCACTGGATCCAACCGCAAGGTCAGAGGTCCGCCTTTCACCTATAGATAAATTCCCTGAAGGTCACCCGTATTCTTTGGATCGGACCAAGGCGCATAGAATTTGGATTGAATTGTTTTGGCTGTGCAATCCCTCAGGTGATTTGGATGACGAGACCCCCGCCGCCCTTACGTTGGCCCGCAGGGTGAAGGGGCTGGTGACTAAGCCAACTAATGGCCGGTGGTGTCCGAGGGCAACAGAGCGAGACGTTCGACTTAAAATACAAGGGCTGAGGACCGTGATGATGTCACAGGAGGAACACGGAGGCCAGAACACACATAGGCTAGCAGAGCTAACTAAAAACCTACAGGTAGCAGGTCGCTTGGTCTTTCTTATGAATGTCCAATGTGAGCCATAGCAACTCCCTTCTTCTGGGTTTGGACTTTTGAGGCTGATTAGCATTCGTGTTATTCCTCCGATAGATTGTGAAAGATCAGTCATGGGAGAAGAGGTGGGAAACGTCAGAGAGGATAAAAGACAAAATAAAGGTCTGCCTAAATTCTTTAGATTTGGTTCTTTAACTTTATTGACTACCTTATTCTATTCTTAACGGGCGTCTTGTGTGCCCCTGATTTCACAGGGATCCCAGTCAACAAAAGGCAAGTTGCACAGTGATGGTGATCCCAGCATCGATTTTAGAGATACCACTGAAAAAATTAAATATTTACAGGAACAACTGAGACAGAAAATGGAGGAACATATCAGAGGTAGAAATGCTGCAATGACTTAAGAAAGTGAAAGATGTGAAAACTCATAGATACAAAGGAATACCATCACCTTACATATTATGTGTTGTTTTCCCTCACAGAGGGCAAAGGGAGTGCGGATAAAGTTCAGGAGAGAGTAACTAGAATCCAGCAGCTACGGGAGTCTCTGAGAGAGGAGATGATGAAACAGGGGGTTGCCTCAGAAACACCTGACCTCTCACAGCAGGTACCTTAAATTACCTGGTAAAGAAAATATGTGCACCGTGTTTTATGGGTCTATTTTCGACAGCCTCAAAACAGGAGCCTCACACCCACATCTTAATCTTTGACAATGTCCCTTAAAGGCCTGAGGTCTGTACTACGAAGCAGGATTCGGGGTTAGCGAGGTAAGTTCAATCCTGGGTTTTCAGTGTCACGGCGGTGGTTCCCTTTTTACCGTGGTATATCGCCATGGTAACTTTTGCTGCACACCTAACTTGCGGAACAGGTTGTTCCAGATAACAGATCAACACGTATAAAAGGATCGTTGTACGTGTCTTGATAGCTTTCAGTTTCAAGAGGTTTACGTCATTCAGATGCAGAGAGATGGGGGGTGGGGGCAGTCAGACACTTAATTTTCAACTCCTGATTATACAGTTATATAGTGGCAATTTTTTTTAATTAGAGTTTATATATATAAATTCTTCTTTATTTTAGGAAATAATTACCGAGGTCCGGGAAAGGGCAGATTGCACAGTGGCGTGCTGGCGCCATCGCAGATGAAT

The scf7180003315926 sequence probably encodes a full-length CD74a molecule, although we were unable to predict the expected two exons coding the short and not well-conserved sequences of the C-terminus. The scaffold encodes at least the bulk of a typical CD74a molecule:

MQSPEEGPLTNSREALVDSVRVRGGSNSRAFKIAGLTTLGCLLLASQVFTVYLVVNQKQE

IHTLQQGSEDLKKQLLVRSSQPQMKMYMPMPGMSLLKGFQNEDETAETSVKNAEYTPPPS

VETQMEYLLQDSQLPHFNTSTFLDNLQSLKAQLEESEWKGLESWMRHWLIFQKAQEKPPA

SETENPAQTQSKCQMEASVKTLGSFRPQCDDTGRYLPMQCWHSTGYCWCVDESGTAAEGS

HTRGKLSCQK

**3.3 *Zeus faber***

**For the non-gadiform fish *Zeus faber* no intact full-length classical *MHC IIA*, classical *MHC IIB* and canonical *CD4* genes were reported.**

*Zeus faber* is a non-gadiform fish investigated by Malmstrøm *et al.*^1^ which is closely related to the gadiform fishes, and hence important for deciding the phylogenetic stage at which functional MHC class II system genes were lost.

In summary, our findings for their Table S7 listings are:

**MHC IIA:** Table S7 mentions utg7180002667991, but this encodes a non-classical MHC IIA molecule. Namely, the encoded molecule is of the non-classical DB category and lacks a second cysteine in the α1 domain and the two asparagines typical for peptide binding function in classical molecules (shaded red in Fig. S1; Zhou *et al.* 2009^3^; Painter *et al.* 2012^4^; Dijkstra et al. 2013^5^). Another unitig, utg7180002375617 (not mentioned in Table S7), encodes a classical type MHC IIA molecule, but the unitig may be too short and appears not to include an exon for a leader sequence. Investigation of the scaffold dataset did not resolve that problem.

**MHC IIB:** Table S7 mentions utg7180002225258. However, this unitig only encodes the N-terminal half of an MHC IIB molecule, possibly because the unitig is too short. We were unable to find *Zeus faber* unitigs or scaffolds reported by Malmstrøm *et al.*^1^ with full-length classical *MHC IIB*.

**CD4:** Table S7 mentions utg7180001946853. However, this unitig only encodes the middle region of a CD4-1 molecule, possibly because the unitig is too short. We were unable to find *Zeus faber* unitigs or scaffolds reported by Malmstrøm *et al.*^1^ with full-length canonical *CD4-1*.

**CD74a:** Table S7 mentions utg7180000026784. This unitig encodes a full-length canonical CD74a molecule.

**CD74b:** Malmstrøm *et al.*^1^ did not find a *Zeus faber* unitig for CD74b. We also were unable to find CD74b-encoding unitigs or scaffolds.

Below the respective unitig sequences are shown with their relevant protein coding capacity:

***Zeus faber MHC IIA***

>utg7180002667991 length=7499 num_frags=1216 Astat=219.00

(the complementary sequence is shown)

TTATACAAATCCAAATTGCTGCCCCTTCCCAAATGAAGGAAGCGATAAAACTATATCTGCAGAGGATGAAAATCTAACCCACCCAGGTTAGAAATAAATGAACTAAATCACACGACGACGATGATGATGACGATGACGATGACGACGATGATGACGATGACGACGATGATGACGATGACAACGATGATGACGATGGCGATGACGATGATGACGAAATGCAAAATCACCTTTGGTTAAATAAGCTTTTTCCAAAGTGCACCAGTGTGAATTCTCATAATTATCTCTCTTCGCGCGGCCTGGAGCTATGCCGCCACCCTGCGGTCACGTTGCAAGGCCCCCAGTCAAAAAGGCCCCCAGTCAAAAAGGCCCCCAGTCAAAAAGGCCCCCAATCAGAAAGGCCCCCAATCAGAAAGGCCCCCAAAATCGATGGACGACAGGTGAATCGATAGACGTCGGAAGGCGTTTAACTGAAGCCGCCGCCCGGGGGCGCTCTGGTGGCCCCCGTCTAATCGTTGCGGTCGCTTTTCCATGCGGCCGCTTCCTGGTGCCTCGGGACGTCGTCGCTGCGGTTGCGGTTTCGGTTTCGTCCAGGTGAGCGGCGGAAAATAAAACCTCTTCGTGGTGTTTGTTTTTTGTTGTTTTTTTTTTGTGTTGTGTTGTTTTTTTTTTCTTTGGTTCCCTCCTGGTCGGTGTTGTGTCATCCGCGCGTTCAGCCCTTGACGTCGGAGGGACGGGGAGCTGTGGAGCCGTCAGCCGCCCTCACGTTATAACGCGCGTCGTTCTCGCTGACGTTGTAACGCGTGTAAAAATACAAAAAAAAAATATATATATATACTAAAATGTTCTTGGGGACCCGCAACAAATTGATATTTTCTACTAGATTTGTAGGGTGTGACATGATTCATACAAGCAAACCCCCCCCTGGCAGAAGCGACATGGGAGTGAAGCTTCAACGCAACTTGGATACAGTGGAGTTCTCATGAAAAAAAGTTCTTCCAGAAAGTTGAGGAACCCGTTTGAATTAAAAGCCGCTTCTGTACAAAACTCACCCGGGTGAGGGCGTAGCCACCGGGTGTCAGGACGTTTCGCTGCATATATGTATGACCGTATGTATATGTACGTGACATTTAATCTCTTGAATTTGAGCTTGAATTTGAGCTTGAACTTCAACAGTGTTTTCCAAGTGGGGTTACTCGTATCCCTAGGGCTGATTTGGAGTACTGCAGGGGGTACTCCCAAAATAAAAAAATACATGTGTTAAAAATATTGGTTATATACGTATCATGTAAAAAAAAACAACCCCACCAACGACGCGCCCACAGTGCATGATTGGTGTTGATTTCATTTCTTCTCTTTTCTCCCAATGCCAGTGAAGGGGGGACTCGGCTAAAGATCCACATCTTGGAGGGGCCTCGCTGCGGAAAGGAGCTTTGCGAACGCGTGAGGGCGGCGGGCGCCTGGTTGGGAAGCCCACCTCTGCCCACCTCTGCCCACCTCTGCCCACCTCTGCCCACCTCTGCCCACCTCTGCCCACCTCTGCCCACCTCTGCCCACCTCTGCCCACCTCTGCCCACCTCTGCCCACCTCTGCCCAACGCACGGGCCGCGTGCGGGACTCCTCCTCTGTGGGACAGTGAAGCTGGAGCCGGTTTCTTCTCCTTCTTTCTAACTTTCCGCTCGTTGGTCGTCAGAGCTTCTCTCCTGCGGAGTGACGGGAGGGGCCGCCGCCGTCATGGCGACCGGGAGGCTGCTGCCGCTGCTGCTGCTTCAGGTGCTTTCCATCAACTCAGGTGACAATCTGATGAAGTACCAGTACTCTCACATACAAGTACCAGTAGACTGAAATAGCAGTACTCTCACATACAAGTACCAGAAGTCTGGAGTACCAGTACTCTGACATACAAGTACCAGTACTCTGGATTTCAAGTACCAGTACTCTGTCATACAAGTACCAGTAGACTGAAGTACCATTACTCTGACATACAAGTACCAGTCCTCTGGAGTACAAGTATGAGTACTTTGGAGTACAAGTATGAGTACTCTGAAGTACAAGTACCAGTAAGTATCAGTACTCTGAAGTACAAGTTTCAGTACTTGGAAGTACAAGTACCAGTTCTCTGACATACATGTCCAGTACTCTGAAGTACAAGTATCAGTACTCTGAAGTACAAATACCGGTACTCTGAAGTACTCTGGTCAAAGCAGGAAGTATAGAAGTAAATGTGCTATTAGATGTATTGGAAGTATTTCAAGAACCTAACTCCCTGACACTCTAGAAAGCACCTGCTCATGAAGTACTTGATGAAGTACTTTACAAAGTACAGATATTTGAAATGCAGTATAGTAAAAGTGGAAAAGTACAGTTCTAGTGAAATGTTTAAAATACCAGAAGCGAGGGTTCCCAAACTGGGTTCTGTGGCCCCTAGGTGTCCTCAAAGGGATTGCAGGGGGTCCACAGCCAAATTATAATTTCTTATTTTGCCTTGCATGTTTCAATATATGTATTTTGTCCTAATAGACATTCACATTGGGAATCTGAAGGTCAACCAATGACATACAAAGAAGATCATATTTCTCTGTCAGTTAGTCAACTCCAACTGGTTGCGCTGGTTTCCTGCAGAGCAAGAAGCAACCTTCCTCTATGGCTGCGCCCAGTCGGGGGAGGGCCAGGCCTCGCTGTACTGGGACGGGGACCAGGTCCTCCGCGCCGACTTTGCGGCGGGGCACGAGGAGTGGACGGGGGCCATGTTGCCCGAGCTGAAGGACAAGCTGTGGCCCGAGTTCTACCTCACGGCCCAGATCAGCAAGGAGAGGGTGTATCACCACTACCTGACCAAGGCCATGGAGGGGGACCGGCTGGTCCCCAGGACCAGAGGTACAGGGAATACTGCAGAGTATATCATATTTTATTACAATATATTATATTGTATCATATTATATTACAATATATTCATATTATATAACTATCTTCTTCATCTTTCGGCTGCTCCGTGAAGTACTGGATTCCGATTGGTGATGACTGTTTTTTAATAATGGCTATTGCCACGGCAACCACTTCTACAGGTTAAAGCTAACGCTAGTTAGTGGCGCCACCAGGTGGCACTACATAGAAACTACAGTGGGAGACTTGTATCTAGTTGAAAATTAATAACATGCAATTGTTTATTATTATGTTAAATTATAGTTATACCAGTAAATAGTTTATTCATATTCATATTTGGTGTTTTCAGACCAAACAGTTCTGGAGACTCCCATTGAGGGGAACTGTGGAGTTCCCCCAGGAACTGAATCTTTAAGTGCTTTATTTCTCTTTGCATCGGCAAGTAGGAACGCTGAAGTGACGTAAGCCAATGGGGTTTCGGCACGAGTTCCCTGTTGTATTAGGCTGTAAACCTTCACTGGGTTCACCTTATCAGTCCCACCCAAATCTGTTCCTGAACAAATTTGAGGCGAGAAATAATCAATCCAGGTGCAGAATCTTCACTCCTATTAGATCTACAAGGTGTCATTTCAAAGTTTTCTCCGACTCTGGTGAGGCGAGGGCACGCAGCGGGACACCGCGCGGGTATTTTTTTCTTTCATTCAATTTGGAATGGGAAATTAATGAAATAAACACACCTACAATAAAAACGCCATCTCTTTTATTGTTTTTTAATTTTTATGAGTTGTTTTTTTTTTATTGGCGCCGAGCGTACGTGGGCCGCCCAGATCGATGCGACTCGGTCGGACTGCGCGGCAAGGCCTGACGCTGTTTTTTTTTTTGTTTGTTTTGGTTGTTTTTTTTACATAGATGTTAATGTTTTGAAAATAAATTGTGTGTAGATAAACACATGTTTACTTTAACTTCAACTTCCAAAGCGTCAGATTTTTGGCCTCGGTTGTCGTTAACGTTTCACACGGGGCTTTTTTGAATACGGCGGTCGCCGAGGCTGCATCGGCTCATGCGTTCGACCCATCACCGTGCACTTACGTCACTCAAAGTTCCTCTTGTGCTGGAAATGACCCGCCCTGGAGAAGGAACTAAAAAAAAGTCCTTCCTCCGAATGTTGTTAGAACTATGAAAAGGTTGCTGTGGTGCAAATCGCCGACTGTGACAGTTTTTCCTCAGTGGAAGATTTGAAACTTGCCCTTCCAGTTATGAAACTAGTCGTACTAGTTATAAAATATATTCTGTCGGTGTGTTGTTTTATTGAGGGAGGATAACGTCCATCCAGATGGTCATTATGTCTAAACAACTCCTCCAGAGTGATGGTGGTCAGTTAGAGGTGACGTCTGTCTGGCTGCACCTTATTCCTTCACTACCTCCCATGTCCTGTGTCCTGGCGAAGCACCGGCAACCTCCCTCTTCCCGGCGGACGAGCCGGTGGCAGGACAGGACAACGTCATGGTCTGCCACGTCAGCCGCTTCTACCCGCCCACCCTCAACGTGACGTGGACGCAGGACGGCGTGCGGGTGACGGAGGGCGCCGTCGTCAGCGACCCCTACCCCGAGGTCGACGGCTCCTTCAGCCTGGTGTCCAGCCTGCCGGTGCGGCTGCGCCGCAACTCGCAGCTGGCCTGCACCGTGGGGCACCAGACGCTCCGACGCTCCTACAGCGCCACCTGGAGTGAGTCCATGCACCGCACTGGACGGAATGAACCCGAACACACTCCGATAAGATAAGATAAGATAAGATAAGATAGACTTTAAAGATAAGATTTTATTAATTCCCAGTTTCAGGCTCAAATTCAGGTGTTACAGCAGTAAAAAGTCTATTTTAACACATGATTGGTACACATTATTACAATATTGATACACATTATTACACTATTGCTACACTACGGTATATTACACTATTGATACACATTGCTACACTATATTACATTACTGATACACATTGTTATACTATATTAGACTATTGATACACATTGCTACACAATATTACACTATTGATACACATTGCTACACTATATTACACTATTGATACACATTGCTACACTATATTTCACTATTGATACACATTGCTATACCATATTACACTATTGATACACATTGCTACACTATATTACACTATTGATACACATTGCTACACTATATTACACTATTGATACACATTGCTACACTATATTTCACTATTGATACACATTGCTATACCATATTACACTATTGATACACATTGCTACACTATATTTCACTATTGATACACATTGCTACACTATATTACACTATTGATACACATTGCTACACTATATTACACTATTGATACACATTGCTACACTATATTACACTATTGATACACATTGCTACACTATATTACACTACTGATACACATTGCTACACTATATTTCACTATTGATACACATTGCTACACTATATTACACTTATAAATACACATTGTTATACTATATTACACTATTGATACACATTGTTATACTTTATTACACTATTGATACACATTGTTATTCTATATTACACTACTGATACACATTGCTACACTATATTACACTATTGGTACACATTGTTGTGCTATATTACACTATAAATACACATTGTTACACATTATTACGCTATTGGTACACATCACATTACTATTACACCATTATTACGCTATTACAAATTATTACCTATTTTTACACTATTATCACAGACAATGAAATTATTAATCGCAATTATTTGTATGATAAGGAATTGCCACTAGAATGTCCCGATCGATGTGTACTGATTGAAATATCGTGTTTTTGTGAATATATTCAGAACATGTATGTGGTGAGTGGGTGGTCAGTGTCACAGGAAGTTACCTCATCTTACTTCCTGTTCCTCAGGGTTGGATGTGGCATGGGCGGACCGCAGCGCGGCCGTGCTGTTCGGGGTGTGCGCGGCGGTGGGAACAATCTGTCTGGTGATGGGGGGAGTCCTCCTCAGGAAAGCACGACAGAGGAACTGAGGCTCCAAACACCAAATACTGCACTATTTACTTGCCCTGTAATACCAGTACTCATACTGTAGTACCAAGTATTTTACCAAATCAAGGTAAAGTTTTAATGAAACACATTGATCATGTACGACTTCTCTCAAAGCGTACTCTGAGAAGTATTCAGTTACTAACAGTAATGCTTAATTACTCAGTAATAGTTACTCAGATTAATACTGAGTCTGCAAAATTTCTGAGAGTAGTACTTAATTACTCTTTGTACTTTGTTACCCAGAGTAGTACTTCGTTTCTCAGAGTATTAAGTATTAACTCACAGTAGTACTTAGTGACTAGGGGTAATGCTTAGTAGTTACCTTAAAAATAGCTGTAATCTGCATTTATAATAATACATCTCAATTTATATGTTAATCTGCAGATTTATTAAACAGAATAAATGTTTCTGTAACCATGGCAACGTTTGCAGTTTTAAACAGGGGCATCGGCTACTATCATTCCTGATCAAAATGATAGATGACACAGAAAACTTTATTAAACTCAGGAGCCCAAATGATGATTTTTGATCAACATATATTATTACACACTTCTTAATTCTGTAACTTTTGCTTCTGTTATATGTTGAATTTACACAGTGACTGAATGAATGAATAAACAATAACCTGTGATGATTGTAACTATTATGAGTCTAATGAATAATACATGTTCAAACTAATGTTTGCATTTAGAATAGATGTCGGCTGTACGTTTCATGGGATAATGTGAACGGAGATTGAATTCAGTTATGAAATGTGGGAGAGTAGATGAAGTCAGTGGAAACATATCCTGAGTGATTATTGGATGTGTTGTTATAATAACCAACATAAGTCACTCAACTCCCGGGATTCCTCGGGACGCTCCCGGTGACGCTGTGCGACTGTCTGCTTTAGTCCTGTTTTCCTTTTCTCCTGAATGTAAAACCGAAGTCGATATTACTCAATGATAGATATGGATCTAAATAACACTAATCGAATATGATTGCAATCCTTTATGACAACTTCAAAATCCCCTCCGAAAAAGGGGATGTGGTGTGACTGGCCCGACCTCCACGCAGAGCGATGGAAATGACTCATCGCGCTCTGTCGCGAGCTGTGGAAATGACCCGTCACCGCCCGCGTGAAAGTAAAACACCGCCTGCCGTCCAACTTCCCCTGTTGCGTTTAATTTTGGAGATTTGAAGCTCTGTGTTCTGGTGGTCGTACTCGGGGACGGACGACTTTTGCTCCTAACCCCGAGAGCCGACGAGGCGCGATCGAGTGTGTCCTTCACTCTTCAGTTTACTTCAAGACCCACCCCTCGTGGGGGATCACACTTCAGATACTTCCTCTTTATTTTGTGGTCCCAAGTCGCTGTCGGTGTGTCATCTCTACCGTGGTTCTGCTTTTTTTTATTGGTTTATATAAGGATTCATAGTCCACCATGGAAACCCTCAGAGCTACAGTATATCCGCAAGAGAACAGGGAATCCGAAACGTCCGAGACACGCATTTGAACACATACTGTACTCACACATACACACACACACACACACACACACACACACACACACACACACA

This utg7180002667991 sequence includes a full-length intact MHC class IIA gene which encodes the sequence:

MATGRLLPLLLLQVLSINSEQEATFLYGCAQSGEGQASLYWDGDQVLRADFAAGHEEWTG

AMLPELKDKLWPEFYLTAQISKERVYHHYLTKAMEGDRLVPRTRAPATSLFPADEPVAGQ

DNVMVCHVSRFYPPTLNVTWTQDGVRVTEGAVVSDPYPEVDGSFSLVSSLPVRLRRNSQL

ACTVGHQTLRRSYSATWRLDVAWADRSAAVLFGVCAAVGTICLVMGGVLLRKARQRN

In this case the organization into exons may help readers to more readily investigate the non-classical character of molecule. Therefore, below this exon organization is shown by translation of the relevant parts of the above nucleotide sequence:

1690 1700 1710 1720 1730 1740

TGGTCGTCAGAGCTTCTCTCCTGCGGAGTGACGGGAGGGGCCGCCGCCGTCATGGCGACC

M A T

1750 1760 1770 1780 1790 1800

GGGAGGCTGCTGCCGCTGCTGCTGCTTCAGGTGCTTTCCATCAACTCAGGTGACAATCTG

G R L L P L L L L Q V L S I N S

1810 1820 1830 1840 1850 1860

ATGAAGTACCAGTACTCTCACATACAAGTACCAGTAGACTGAAATAGCAGTACTCTCACA

(stretch not shown)

2590 2600 2610 2620 2630 2640

AGTTAGTCAACTCCAACTGGTTGCGCTGGTTTCCTGCAGAGCAAGAAGCAACCTTCCTCT

E Q E A T F L Y

2650 2660 2670 2680 2690 2700

ATGGCTGCGCCCAGTCGGGGGAGGGCCAGGCCTCGCTGTACTGGGACGGGGACCAGGTCC

G C A Q S G E G Q A S L Y W D G D Q V L

2710 2720 2730 2740 2750 2760

TCCGCGCCGACTTTGCGGCGGGGCACGAGGAGTGGACGGGGGCCATGTTGCCCGAGCTGA

R A D F A A G H E E W T G A M L P E L K

2770 2780 2790 2800 2810 2820

AGGACAAGCTGTGGCCCGAGTTCTACCTCACGGCCCAGATCAGCAAGGAGAGGGTGTATC

D K L W P E F Y L T A Q I S K E R V Y H

2830 2840 2850 2860 2870 2880

ACCACTACCTGACCAAGGCCATGGAGGGGGACCGGCTGGTCCCCAGGACCAGAGGTACAG

H Y L T K A M E G D R L V P R T R

2890 2900 2910 2920 2930 2940

GGAATACTGCAGAGTATATCATATTTTATTACAATATATTATATTGTATCATATTATATT

(stretch not shown)

4270 4280 4290 4300 4310 4320

AGAGGTGACGTCTGTCTGGCTGCACCTTATTCCTTCACTACCTCCCATGTCCTGTGTCCT

4330 4340 4350 4360 4370 4380

GGCGAAGCACCGGCAACCTCCCTCTTCCCGGCGGACGAGCCGGTGGCAGGACAGGACAAC

A P A T S L F P A D E P V A G Q D N

4390 4400 4410 4420 4430 4440

GTCATGGTCTGCCACGTCAGCCGCTTCTACCCGCCCACCCTCAACGTGACGTGGACGCAG

V M V C H V S R F Y P P T L N V T W T Q

4450 4460 4470 4480 4490 4500

GACGGCGTGCGGGTGACGGAGGGCGCCGTCGTCAGCGACCCCTACCCCGAGGTCGACGGC

D G V R V T E G A V V S D P Y P E V D G

4510 4520 4530 4540 4550 4560

TCCTTCAGCCTGGTGTCCAGCCTGCCGGTGCGGCTGCGCCGCAACTCGCAGCTGGCCTGC

S F S L V S S L P V R L R R N S Q L A C

4570 4580 4590 4600 4610 4620

ACCGTGGGGCACCAGACGCTCCGACGCTCCTACAGCGCCACCTGGAGTGAGTCCATGCAC

T V G H Q T L R R S Y S A T W

4630 4640 4650 4660 4670 4680

CGCACTGGACGGAATGAACCCGAACACACTCCGATAAGATAAGATAAGATAAGATAAGAT

(stretch not shown)

5770 5780 5790 5800 5810 5820

GTGTCACAGGAAGTTACCTCATCTTACTTCCTGTTCCTCAGGGTTGGATGTGGCATGGGC

L D V A W A

5830 5840 5850 5860 5870 5880

GGACCGCAGCGCGGCCGTGCTGTTCGGGGTGTGCGCGGCGGTGGGAACAATCTGTCTGGT

D R S A A V L F G V C A A V G T I C L V

5890 5900 5910 5920 5930 5940

GATGGGGGGAGTCCTCCTCAGGAAAGCACGACAGAGGAACTGAGGCTCCAAACACCAAAT

M G G V L L R K A R Q R N *

In contrast, another unitig sequence, utg7180002375617, encodes a classical type MHC IIA. However, gene prediction software (FGENESH and GENSCAN) and database comparison analysis (blastx at NCBI) were unable to find a leader coding exon in this sequence.

>utg7180002375617 length=3569 num_frags=463 Astat=174.00

CTTCTTCTTCATCATCATCATCATTATTCATCACCATCATCATTAATATTCATCATCATCATCATCACCATCATTATTCATCATCATCATTATTCATCATCATCATCATCCTCATTATTCATCATCATCATCATCATCATCATCATCATCATTATTATTATGTCACGTCATCATGAGGTAGTGGATTCCGTGGTGGTTTTCCAAACGCACCCCGAAAGCAACTTGTTGAAACTGCGACTAACTTTTTGTACAAATAACGCGTTAGAAACGTTGACAAAGACGGTTCTATAATTCCTCGCAAACTCAAAAGATGTCGAACAAACAACTGCAAATCACAGGGAATAGTGATAAAGTCGTCAAAATTGGAACCGCGGATGGCATCGAACCTCGGGAACCACGGTCAGCTCCGCGGGCCGCGAGCCGGAACTTTGTAGAGTAAAATATATTAAATTCACTTTCATCATTTCTACATTGTTATATTAACATTCACTGAGAACTCGAATTTCATTAGTAAAAGTATAGATTTTAGATTTATAGAGAGATATCATCTCTTTATAAATAGATTAAGACATTTCACTGCATATATGTATGAACATATGTATGTGTATGTGACAAATAAACCTCTTGAATCTTTCATCTATCTATCTCTGTCTGTCTGTCTGTCTGTCTGTCTGTCTGTCTGTCTATGTCTCTCTGTCTGTCTGTCTGTCTGTCTGTCTGTCGATATGTATTCATACATATATATATATCTTTATATATCTATATTGATATCTATATCTATATATATTTATATATAAATACATATATATATATCTTTATATATCTTTATATCTATATCGATATCTATATATAGATATATATATATGTATTGTACTTTTATATGTATTTGTACTCATGTGTTTCTTTCTCTGTATTTCCAGCTAAGCATGAAGATTTGAATATAGTTGGCTGCTCCGACACTGATGGAGAGTTCATGTACGGCCTGGATGGTGAGGTGAGGGCCTACGCAGACTTCCTCCTCCAGAAGGCAGTGGTTGCTTTGCCAAAGTTCGCAGACCCCATCGGCTACCCAGGTCTCTACGAACAGGCTCTGAGTGAGATGCACATCTGCAGAGAGAACCTGAAGGTGATCCGTGGATACTACAACGACACCGCTGTGGAACTAGGTGGAGACACACCGTCTGAACTACACCTCCTCCAGTAGATTGGTCAATACTTTGATAAAAATAAACTAAGGACTATACTTATGTTCCTCATATATTAATATTTGGGAAGAAAATGTGTTGTTTTTTTAATCGTACTATAAACACAGTCTGCATTCAAGAACACTTGTAAGTAAAAAACAAAATGTTCTACACTTTCCAATTTAAGGATAGATGGATGGAAATCTTAGTCAGCGATTTCAATGAGCATATCATTTATTTTAATGATACGTCATATATTACATCATATTTATATATTATAATCCTTAATAAGATAAATTATAATATATATATATTTTTAAATATTTTAATCAATTTACCTTTTCTTCGTTTTTCTATTTACGTTTAGTGGAATTATGTCAAATCATTTTATAATAATTTCTTTATCAAATGCAGATGTTGAATGTGGTCTAAATATCGTTTTTATTTTAGGTATATTTTCAGTTTATACATACATTTTACAAACAAATCAGTTCAGCTGAGATTTTATGATAGATGCCTGATGAGTGTGAAAATTAAAGACTAAGCAAGTTACATTCACAAGAAGAAGCAACTTTACTCTGCAGATGTCTTAATGTTTTACTAAATATCGACCAATTAGACACATGAACCAAATAACAACTAATTAATTCTATAGATTGACTGCATTACAGCATACAATTATAGATATTAAATTAAACCTCAATGAAACGTATTATTCAACAGATTATCAAAAAAGATGATTTAATGTCGTGAACTGTTATTTTCCAGGTGATCAATCAAGGGTTATTGGCAGGAGTGAACAAATCAAAGCCGGTTATGAACCATTTTGTTTTCCCAGATTATCAGTAAAGCATGAAGCATGACAGTGGGGATTGTTGTGTTCAGATGTTCCTCACAGCTCCATCTACACCAGAGACCACGTGGCCCTTGGAGTGGAGAACCTGCTGATATGTCACGTGACCGGCTTCTACCCGGCGCCCGTCAGAGTCCGCTGGACCAGGAACAACCAGAACCTGACGGACGGCGCCACCATCAGCCAGCCGTACCCCAACAAAGACAGCACCTTCCACCAGTTCTCCAGCCTCAAGTTCACGCCGCAAACCGGGGACATTTACAGCTGCACTGTGGAGCACAGGGCTCTGGACGAGCCCCTAACTCGAGTCTGGGGTGAGAAACACGACATAGAAATATGACACATACGACGGTGCATATGGATTATTACACATTAAAATTACAATTGTGACAGACTAACAGAGCCCTTGAACTCTAGTCTGCCCTCAGAGATAAAATACTATGCATAATACGCATTATGTGACGAGGCCGCAGAAAGACCCCCCCCCCCCCCCCCAGATTTTTTCCAATTCATCTTTTAGATGCATTTCCATGACGACGTACTGCACTTAACCTGCAGTTGATCCGGTTGGTGGTCATTCGCTGAAAGCTACTTCCTTTTAGAGACAAAGGGCTGCTATATACAAATGTAAATATAGGTTGAAAATCAGCAAACTGCCCTTTACCGAGGTCAGAATCATGAATTTTTAAAAATATATATTATAATTATGTGTATGTGTATTATGTATTTTCTCCAGACGTGGAACAGCAGCAGCCTGGAATAGGACCTGCAGTGTTTTGTGGAGTGGGTCTGACGCTCGGTCTGCTGGGCGTGGCTTCAGGAACCTTCTTCCTCATCAAAGGGAACGAGTGCAACTAATTCTTCCGCTGCTGGCGCTGATTGGCTGACTGCCATTTCAGTCACTTCCTGTCTGCAGTTGTTCGTTGATATTGTGTTTGGAATAAATAATGATGTTGTTTTTTAACTGTTGTTTTTAGACAAATTCATCTTTCTGATTGCTAACAATAATTTTAAACCATAAGTAATTTGTATAGATTGCATAAACTTTATTTTGATAAAGATATTTTCATTGAAGCCGGACCTCATTTTCCATGTCAGACCTTTCAGTAGGGGAAGGGAAGCTGCTGGAAAGTGCATGAAAATGAATACAAAACACCATCAGCAGGGAATATTTTATTATATATTGTTTCGATATCTATTTACAGACATTCAACCTGGGCCAGGAAGAAAATGGAGGGAATTATTAAACTTGAGTGATGTAGAAAGTATCAATCACGAATCAAAAGAAAAACCATGTACAAATCTAAATAAAGAAATAATCAATATAACTATAAGTCATCAATATACATTGCATATTTTAAAATGCTTTTCTATCCATCTGAAGTGGGAAATTTGTAGCTTCAACTACAGTTCCCATTTTGTGTTTGTGTGTGTGTGTGTGTGTGTGTGTGTGTGTGTGTGTGTGTGTGGGAGTGGGAGAGAGAACTGAAACGAAAACGAAGAAACAGTCTTTATTCGTCACGCAACACACC

According to prediction by FGENESH software, the (incomplete) MHC IIA sequence encoded by utg7180002375617 is:

MASNLGNHAKHEDLNIVGCSDTDGEFMYGLDGEVRAYADFLLQKAVVALPKFADPIGYPG

LYEQALSEMHICRENLKVIRGYYNDTAVELDVPHSSIYTRDHVALGVENLLICHVTGFYP

APVRVRWTRNNQNLTDGATISQPYPNKDSTFHQFSSLKFTPQTGDIYSCTVEHRALDEPL

TRVWDVEQQQPGIGPAVFCGVGLTLGLLGVASGTFFLIKGNECN

***Zeus faber MHC IIB***

>utg7180002225258 length=1995 num_frags=223 Astat=122.00

(the complementary sequence is shown)

TGCAGCAGGGTTGCTGGGCTGTCCTCAAACTACTTTGCAGATTTCAAAAAGTCGTAAAACCTTTTATTTTGGAGACGTGAAAAAGGAAACTACATTCTAGCTGCTGACAGGGTCCCCTGTACAAGGCAACGGATGCCAGCTTTTATTTTTGATGTGGTTTAAGCCTGGATAGCCCCAGTGCAGAGACCACGCTAACAGAGATCACTCATGAACGTTTGCTGATTTGATAGGAATTTTTAGGATACTATAATTGCATTGTTATAGAAATGTCACTCTCTACAATAAAACTTGTGTTTTCTACCCTGTGGTCAGTGTCTCTGTGCCAGAGGACCCTGAAAACGGCACCCGGGTGGCGTGCGGCAGGCAGATCTACCACAGAGACATTTGCAGCAAGAAATTGAGCAGCGATTAAAAAGAGATCCACCGAGATTGGCCAACCTTGTCTGATACCACGACAAAGGTTGAATCTGGTAGAAGTAGCTTTTTTTAAATTGAATTGAGCTTGTAGCATTCTCATCGAGAGTTTCAACAGCTTTACAAAAGAAATCCTCAAGGCCCGTTTTTGATTGAGCTTTTAAAACCAATCCATGCTCCACAGAATCAAAAGCCTTGTATGAATCAAGGAAGAAAATGAAGCCATCGCTATCACCAAGATCATTATAACCAAACAAATCAAAGACCAGTCGGATGTTATTGTAACATGACCAAACTGAGTCTGGTCTATGATAGAGTCTCACATATATATTTGAATCTTTGAGTCTGCACATGACAAATAACCTCTTGAAGCTTCAATAATATAGTAGGGCTAAAATATTATAATCATCATTAAGAAAAGTCATGGGGTGCCAATTACCCCAAAAAATAGCCTTTTCTTTCACAGCAAAATTATTATTTGTTATTTCTTTTTATTTGATTGTTTTCCATGTTTTAATATAACATTTCGTGTTGACAGGCTTGTTTGTCTGAGCAATGCAGTGCTGAAGAATTGAGAACGATAGTGGCTTGTTTATGGTGAAAATGCCATTAAATTTCATTTGGATAAAGAATATTTTGTGTTTTTCTTCTCAATAATTGAGAATATTATGCCTTTTTAGCTGCATAATATTTTTAAGGGTACAATTTGCGTACTCTCATGTAATTATTTGAATCGTTTGAAAGCCCGAGTACTTACAAAAAGTCCCATTTCATTATTTTTGGAAATGTCATTTAAGACGAGACTTGGATTGTGGCGCTCGTCTTGTCTCAGTCGTCTGCCGGGCAACCGGTGACGAGGCGGCCATGTGACCGGGCTGTGGCGTCCACATTGGTTTTTAAAGTCCAGTTTGTCAGATTCCCGTCACTAAGTTGTGGCGACTCAACAGGGAAACATGAGCTCGTCTCAGCTCCTCCTGCGCTTTTCCTTGATTTTACTCTCTGTGTACGGAGCAGGTAGGCGGAATCACACATTTTACACCTGCTATCAACTTCCAAAAGCCGTGGGAATTGTTGATATGAAATGGTGATGGTTGAGCGTGCAGGGGCTTCGCGGAATGAAATAACTTTTTTTTTTCTCTTTACTCCGTCAGATGGATTTGAGAACTTTGCCACTTCCCGCTGCGTGTTTAACTCCACGGAGCTGCCTGACATAGAGTTGATTGAGTCTTATTACTACAACAAGTTGGAGATCGTCAGGTTCAACAGCACTGTGGGGAGGTTTGTTGGATACACGCCCTTCGGGACGATGAACGCAGATCGGTGGAACAACGGTGAAGAGCTGCCCAGAAGTAAGGGTCAAGTGGAGGCTTACTGTCAACATAACATTAAGAACTGGTACAACAACATACTGGGCAAAACAGGTGAGCGCAGTAATCCCGTTGTAAACGTTTACAAGCAGGTTACCTTTTAAGTTTATCGTTCATTAAAACGCGGATTAAAGTGATTTTTATTCGTACACTTCTACACATTAAATAACGCGTGCAGAACAGAAAGGTTTTTCGTTGTTTTAATGTGTTCTTC

In this utg7180002225258 sequence, only coding sequences for the N-terminal half of MHC IIB can be found (the figure shows the encoded sequences together with the relevant part of the unitig):

1330 1340 1350 1360 1370 1380

GTTTGTCAGATTCCCGTCACTAAGTTGTGGCGACTCAACAGGGAAACATGAGCTCGTCTC

M S S S Q

1390 1400 1410 1420 1430 1440

AGCTCCTCCTGCGCTTTTCCTTGATTTTACTCTCTGTGTACGGAGCAGGTAGGCGGAATC

L L L R F S L I L L S V Y G A

1450 1460 1470 1480 1490 1500

ACACATTTTACACCTGCTATCAACTTCCAAAAGCCGTGGGAATTGTTGATATGAAATGGT

1510 1520 1530 1540 1550 1560

GATGGTTGAGCGTGCAGGGGCTTCGCGGAATGAAATAACTTTTTTTTTTCTCTTTACTCC

1570 1580 1590 1600 1610 1620

GTCAGATGGATTTGAGAACTTTGCCACTTCCCGCTGCGTGTTTAACTCCACGGAGCTGCC

D G F E N F A T S R C V F N S T E L P

1630 1640 1650 1660 1670 1680

TGACATAGAGTTGATTGAGTCTTATTACTACAACAAGTTGGAGATCGTCAGGTTCAACAG

D I E L I E S Y Y Y N K L E I V R F N S

1690 1700 1710 1720 1730 1740

CACTGTGGGGAGGTTTGTTGGATACACGCCCTTCGGGACGATGAACGCAGATCGGTGGAA

T V G R F V G Y T P F G T M N A D R W N

1750 1760 1770 1780 1790 1800

CAACGGTGAAGAGCTGCCCAGAAGTAAGGGTCAAGTGGAGGCTTACTGTCAACATAACAT

N G E E L P R S K G Q V E A Y C Q H N I

1810 1820 1830 1840 1850 1860

TAAGAACTGGTACAACAACATACTGGGCAAAACAGGTGAGCGCAGTAATCCCGTTGTAAA

K N W Y N N I L G K T

1870 1880 1890 1900 1910 1920

CGTTTACAAGCAGGTTACCTTTTAAGTTTATCGTTCATTAAAACGCGGATTAAAGTGATT

1930 1940 1950 1960 1970 1980

TTTATTCGTACACTTCTACACATTAAATAACGCGTGCAGAACAGAAAGGTTTTTCGTTGT

1990

TTTAATGTGTTCTTC

***Zeus faber CD4*** *(CD4-1)*

>utg7180001946853 length=3280 num_frags=503 Astat=104.00

AGCTCAGAAGGTACAGCAGGTCATCTGGTGATTGGAAGGTTGCCGGCTAGATCCTGGTAGTGTGCTGAAGTGTCCCTGAGCAAGACACCCAACCCCGCAACTGCTCTCAATGAGCGGGTGTGCGCCTTGCACGGCTGCCGCCACCATCCATGTCTGAATGTGTGTGTGTGCTTCGAGTAGTTGCAAGACTAGAAAATACATAAATGCAGCCCATCCATGCCGTCCATGTTTTAAAAAATGAGAGTTTATGACTCTGTAACCATCTGATAGGGGATAAGATGGTGGCACGTGCACAACGCGAGGCTCATCGCACTCCAGACACACACTACTATTTACTGTTTTCACACTATTATTTATTGTTTTCACACTACCACTTATTGTTTTTCTATTCATATTTATACATCCAGCTATTCTCATGTGAATCTGTTAATACTGTATGTATTTTTGCATATTCATATCCACTCTGTTAATACTGCTAATACTGCTCTTACTATGTATACATAGTGTGTCCATATCCATTGTAAATACTGTTAATACCGCTCATTATTCTGTTAATACTGCTCATACTATACAGTATATATTTTAGTGCATTCATATTCATTCTGTCACAACTGTTAATTCTGTACATACTGTACATATTTAATATATCTATACCCACTACTGTTAACTCTATATATATTATTCATATACTTTATACTTTAATTCATATACTGTATATTTTATACAAAACGCTGCATGTTACTGCTTCTCTTGCACATCTGGTTCGATGCTAAACTGCATGTTGTTGTCTCAGTACTTCAGTGCTTTGTGGAATGACAATAAAGTTGAATCTAATTTAATCGGATAGGCCATACCTCACAAACTCCTTCGGGGCCTGCTTTTCTGTTCTTCTGACGGTACTACACTTAGTTTGGTTCTGCTTCTTTGCCCTTACCCGTTTTCCTTCACGTCCACTTCATTCGTCAGTGCATGTAACACCAGCGCCTCGTCTCTTGGCCGGAAACTCGCTGTCCCTGTCCTGCGTCGTGCAGGCGTCTCAGAGCGGGGCGCGGCCACAGTTTCGCTGGCTCGACCCACGCGGGCGGGATGTGGCCAAGGACTCCCGGGGGCAGGGAGCCACAGGACAAACTCTGACAGTCGGGGGCGTCACAGGTCTGGACCACGGCCAGTGGACCTGTGTCGTGATGCACGGCGGCAGAGAGACTCGCGCCAAGACAGCTGTGACTGTTGTGGGTGAGTTTCCGTCAATAAAGACGTGCGATGTCACGCGACAGCTGACAGCTGTTCACGACGAATTTGACCTTTTTGTCTCCCCGCAGACCTCTCCCCGGGTCTTTTGCATCCTTACTACACCTCTGTCTCCTCTAACCGACTCCGGATCCCCTGCTCCCTGCCTCCTTCCCTCTCCTGGAACGACCTGGCGGGGGCTGGTTTTCAGGGAGGACACTGGAGCTTCGCCTCCAGCCTGTTCGCCGGAGCTCCACAGAGGCTCTTCTCGCTCTCCCTGGGGGTTCCGCCGACCTGGCAGGCCGACACCGACAGAGGACTGACCCTGTCGGACCTCGGGAAAAACAACCTGTCCCTGCAGAGGAACCGGGTGGGGGAGGACGACCGGGGGGAGTACACCTGCGCCCTGGAATTCAAAGACGGAGTCACGCTGACGAGGACCGTCCGCGTGGAGGTGCTGCAAAGTAAGACGACTCAGGCGGCAAAGCTCTGCGGCGCAATCCAGATCTATTAATCCAGACGCCAAATAAGATAGATCGGCTTCCATTTATTCTTCATTAAGGTTGTCGTTCTTGTCTTATTTTCTTTCCTCCATTTCCTCGCTCTTTTCTAATTGTCATTCAGAACAAGTCAGACACTGTAATCTCTCTGCCTCCTCGCCAGTGCTCTCCTCCCAGTTAAGTTTTTCAACGTGGAACAGAGTCCCCGGCTGCATTTGGCGGCCGCAGGGCTTGTCCGGCTCCATTTATTTTAATGGCCAATAATGAGCTGCAACACTGCCCGTGGAGCATCGCAACACAATAAATCCAAATTGAATTCTTTCTGTTTATCTTACGAAAAACATTCTCTGCTTGGTATTTTTTCATTCATTTCTCCTTTATGCAAGCGGGGGGAAATCGATAAAAGCTAAAATTCTAATTACAAGGTTGGTGTGACGGGGGGTAATTCCCGCGGCAGAGAAAGGGCCGCACTAAAAAGAGTTATGGTTCAGTGGCTTCATAAAAAAGGCATTCTGACTCGAGGACCAGAGCGCTCGCCTCAATCGCTTCCAATACCCACATATGAGTATTGGAATTCTGATTTACTTCGCTTCATAACTCTAAGGGGGAGGTCACAGAACGATAGAAAGACGACTGGTAATCTGGAATGGGTGCACGGGGGTAGGCCTGTTTAAAAAGGCACTATAACCTGTTATCTCCTGTGATTTTAATATGTATTTGTATGTTTTTATTTCCTACGCTTTTATCCCTCTTGTATTTCTATAATGTATTTTATGCCTTGTGTAAAGCACTTTGAGGTGCTGTTGTGTATGAAATGTGCTATACAAGTCAAGTTACCCTGCTTAGTAATTTCTACCATTTTGGATGAACATAGAGGGTAATAAAAATCATCCTTTCTTTATTTGCCTCTAGTCCTCGCCTTGCCGTTTGCGGAAGTTCGGTTCGGCCAACAAGTCAACCTGAGCTGCAGTCTGGGCCATGCTCTGCCCTCTGACCTGGAAGTGAAATGGATCCCACCCCATCAGCCCTCCCCCTGGATCCGCCCGGCATCCCCGGCCCACCACCTCACCATCTTGGAAGCGGGCGCTGGAGATCAAGGGCATTGGAGTTGCGAGCTATGGCGAAACCAAACCAAAGTGGCGTCGGGCAAAACCAAGCTGACGATCGGTGAGCACAGGAAATGAGACGAACAGGAAGTAGAGCGTCGGTGGGACCCTGCGGTCGATTCAGTAGAAATTTAAATAGACACGTAGACACAGAAATGTGAAAATGATGTCTTATAAAAATGATATTATTGTTTTTAATTATTATAATATATATTATATATATTATATATTATAATTTATTTACAGGTGCTCCAAACTTACAACCTGTTTAACGACCGTTCAGACTTACAACCAGCTCTCTAACCAGTCAGTATTGTGCTCTGCGTACAATACCGTGGCGTCTCAGTGTCGAGCATCTCATCACACATCACCCTTTATATACTATATATACTATAAATACTATATATACTATATACACTATATACACTATATACACTATATATACTA

The sequence utg7180001946853 only encodes a middle part of CD4-1 (Ig-like domains 2-to-4):

HVTPAPRLLAGNSLSLSCVVQASQSGARPQFRWLDPRGRDVAKDSRGQGATGQTLTVGGV

TGLDHGQWTCVVMHGGRETRAKTAVTVVDLSPGLLHPYYTSVSSNRLRIPCSLPPSLSWN

DLAGAGFQGGHWSFASSLFAGAPQRLFSLSLGVPPTWQADTDRGLTLSDLGKNNLSLQRN

RVGEDDRGEYTCALEFKDGVTLTRTVRVEVLQILALPFAEVRFGQQVNLSCSLGHALPSD

LEVKWIPPHQPSPWIRPASPAHHLTILEAGAGDQGHWSCELWRNQTKVASGKTKLTI

***Zeus faber CD74a***

>utg7180000026784 length=13101 num_frags=2283 Astat=306.00

TGATAAAGATTTGTAATTGTTGTCAATAATTTTTACAGTGTAACTAATATTGTCGGTGCTTTTTAATTTGCAAATAAAACACCACTGTATTTTTCAGCTCTCGCTCTCTCATTGTGATCCGACCGGTTTTGTGGTTCAGTCAGCATATTGACGGAAATCACTGGTAGGAATTTCTCCGTCTTATCTCTGAGGGTGTTTGTCACTTTGCCAAATAGTCAAACTTTCTGTTTAATTCAATAAGATCTTATGGGAATGGAACGGTTGCTATTCGGCATATCGTGGATTAAAAGGGCTGTATGATATTTTCCACCCAAATAATAAGTGTCAATTCCAAAATGCTTGTATATTCTCGCCAATTTTTCATTATAGAGAACAGTGAAGAGAGACAGTAAATACCAGGGTAGAGGGAGTGACGTACGGTTCCATGTTTGCGTGGATTACACTGCGGCTACCTCCCGTGAATCAGTGCGTTCTTCCTGGGAAAGGCAGAGCCCGCCACCAACAAGGAAACGGCAAGTGAAGGAGTACCCCGAGCACAGTGGTTGTTTCCTCAAAAGTCACAACTAAAAAACTATCAAACATGGGTTTTTTTAACAAAATAATCTTACCGATTTCCACTTCAATCGCTGAGCCTTCCCCATGTTCAGGTTGTGGTGTAAGGCTTTATCGCCATACGACCTTACTTGGCTTTGTTTAAAAGGTTGTTTATTTACCAGGTCGGCCACAATTCATTACACATTGCACTGCTCCAAAAATGGAAGCATGCTATATTTAATCTAAAGTTTGATTCGAAGGAAATTCCTTTAAAGAAGGAATTTTGTAATTCTGTTTTTTTTATTTTTCTTATTTGATTTCTTTTAACAAATCTTTCCGGAATATTAACACAGCAGCGAGTGGCATATACAAAGATCACACAGATAACACTGCAAAATCTAAAAATATAGGATCTTGTCCGTACTCGTTTCCAAAAGAAAGTTAAATACAAAAGATTTGTAATGTATGCGGTGTATTTATTATCACTATCATTATCATTTTCTTTATCATTAGTATTAGAATTGAAAATATCATTAGTATTGGTGCTATTTAAATAAAAGGGTTCTTATCCCGGAGAGAAGTCAACGCATTTTTCACAAATGTCCAATAATAAGTCATTATTTTTGCTTTTAACAAGTTTTAAGATTCAACTTAATGGACGATAAAGCACATTTGGGAGACGTTTCGATGTTTTCTGCCTCTATCAACAAGTGACGGTGCCTCCGTTCGGCCAATCAGAGCCCGGCGTCCCACAGCACCTGAATATAGGGACTTACCAGTCCCAGTGGTCGCGTCACTTCTCCGCTCATCCTCCACTGGAGCAGGTAACCTGTCGAATTAAATCAACCATATTATTTAACATTTCCTCGAATACTGAGAACATAAATAAAGAAATGGAAACACCGGAGCAAGCCCCACTGGCCGGCAGTCAGGAGGCGCTCCTTCCTTCAGCAAGAGTGAGAGGGTGAGAAAACGTATTTCAATATTATCAGACAACAAACTTTTTTATTTTTATTTTTTTTTTTTTTGACGGTGTGAACAACGTGTGAGACTACATAAGGTTGTATGTTTTGTTTAGTTTAATTTTCACGATTAGAAATACGTGTTTCAATCGTTAGTCCGTATATTCACGTAATCGCGTCTCAACTTTTGGATCTAGCCTGCCTAGCAACTAGTGATGCCTGTTTTTTTCATTGGTCCACAGGGTGCGGGCCCAAAAATAAATAAATAAATAAAAACACTATTCATTTGACTAATAATATTAATAAAGCCAGAATATTTGTTGTAGTTTTTTTGTGTTATTGTTTTCACCGTGGTTTTGTTTTGTGGATGATTATATGAGTGAGTTGAAGTCAGAATGTTCACATTATCGCCTATTCACTGGGGGCTGAGTGAATTATAAATAAAAATTATAAATAAAAATCATCCTTTTTTTTTTTTAATGCCACAATAAACAGAGAGGAATTCTTAGGGGAGTTGTTGTAATGATAGGCCATTGTACAACTATATCACATTATATATGAATCTATTATTTTTTGTATAGGCATCCTTTTTCTTAGTTTTTGTTTATTTATAATAATGTCTATTTTATTTACTTAATTATTATTACTTTTTATTCCCTGTGATCAGGGGTTCCAACAGCCGTGCTTTCAAGGTTGCAGGACTCACCACGCTGGGATGTCTGCTCCTGGCCAGCCAGGTGTTCACAGTCTACCTGGTGGTCAACCAGAAGCAGGCGATCCACACTATGCAGAAAGACTCGGAAACCCTGCGCAAGCAGCTGACCCGCAGTTCCCAGAGTAAGAAATAATGTCATAACACAGTAATAACATGGGATCGGTGAGAGATTGAGATTCAAAAAACAAAAATATTGTAAACCCGTCTTTAATCTCTCAACCGACGTTGGTCAAATGTCAAATGAAAGTGAAGGGGGAAATGAACTGGGCTGTGAAACTGCGCCACAAGGAAATACAAAGTGAAAGGATTTTTCCAGTCTGTGTCTATAGTGAATTAAACAACGAAGCAACGAATTGTCCCTTGCAGCCCCACCGATGAAGATGTACATGCCCATGCCCAGCTTGCCTCTCCTCAAGGGTTTCCAGACTGAGGCTGAAACTGCCAAGACATCAGTGAAGGTGGGTCTAAACTTTTAAGTTCAGTTAGCAGCTATTGAAACAGTGGCCAAACCCCAAATTTCATTTCTAAGAACAGCCCTCTACTGATTAGTTACTGCACTTGTTTTATCGAATTGCGTGAAATTAATACCAGATGACGTCACTTGGTTGTAGTTTGAAGGTTATTATTACCTTACGTTTTGAAGTGAGTCTAATAATACATGTTTTTAGATGAAACTTAAAATATTTTCGTTTTCACTCTCCAACCAGAAACAGGAAGACACGGTGGCCACCGTCGGTCTGGAGACGCAGGTGGAGCGTCTCCTACAGGTGTGTCACATTTCACGCCCCGACTACGTCACGCCAGTATTTTTGCCTGGGAGTTCTGGGTGTCCCTAATGAACCGCAAAACCTTTTTCCACCAGGATTCCGTGCTCCCCCGTTTCAACGCTTCCACCTTCTTTGACAACCTGCAGAGCCTGAAGGAGCAGATGGAGGAGAGCGAGTGGAAGGTAAAGTCTCTCTCTCTCTCACACACACACACACGCACGCACGCACGCACGCACGCACACACGTCCTGTAGGTGGTGTGTTGCCTCTGTATCTCAACCTGACGTTCTGCTCAGGGTTTGGAGTCCTGGATTCGTCACTGGCTGATCTTCCAGAAGGCCCAGGAGAAGCCACCCGCACCCCCTACTGAGAAGCCAGGTGTGCAGAGACCCCCGCCCGTCTCTCCAACATATTTAAATTCAAATCTGAGGCAAATAACTGAATTAATTATCTGAGCGGATATGTGTCTCCAACTTCAAGTCGTTTAAGTCGTTAGGTGCAAAGGGATGAAAGCCAGCTCAGAGCAAACCAATCCTGAGATCCGGTGTTTTGGCAGTTTATAACTCCGTGGATGTTAGATATCCGCAAGGCCTTGTATATAATAAAGTTAGCAAATAAAACGTTATTTACAGAGCACTTTTTGAAACACATTCTTAGAAATTGACCTGCAGGCAGTCAGGTGGCATAACATGAAACACATTTGAAGAGTGGCCACAAAACAAAGGTAACACTTAATAAATGCCTATAAAGCCTATACGAAGTATAAAACGCAGAGAAAAACACATTTGAATGAATAGGGCGTAAAAACAAAGATTAGGCTTTATAAAAAGGCGAAAATAGACTGGCAAACCATTCAATCAAACTTGTGTGACAACAGTGTGTGATTATGTAATATAATGTAAAAGATATATAATCCAAAAAAGGTCCTTCGATAATGAATAATTTGTTCACATAATCCCATCTAAATTATGAGATCAAGCCTGCCTAGCAACTAGTGATGCCTCTTTTTTTCATTGGTTCACAGGGGATCCTCCAGAAAACAAAAAAACAACAAAAACAAATTAGTAAACAATTAGGCCTCCCAAAAATATTTTTTGTAGGTTGCCCTGTTTATAAGTGTATTGGGGGATCAGACAGGGTAGTTCAAATCAATCAGCCAAGCAATCACACTTTATTGATACAGCATGTTTTGCGTGTTAAAATGCCGTGCAACTTTCCTTGGGCACTTTCCCCTCAGCCCTTCCGCTCCAGTCCAAGTGCCAGATGGAGGCGTCGATGAGCGGCAGGCGGCTGGGCTTCTTCCAGCCGCAGTGCGACGAGAGCGGCCACTACCGCTCCGTGCAGTGCTGGCACAGCACCGGCTTCTGCTGGTGCGTGGACGACAACGGCACAGCCATCGAGGGCACCTACAGCCGCAACAGGCCCCATTGCCAGAGAGGTACGCGTCTCAGCTGTTTCTTTAAGCTTTCCGAAATAAAAGCGTCAGAATAGAAGCGGCGAGTTTATTTATCTTGGGGTTTTTTTTTTTTGTTTTTTTTAACTCCCGCTTTGTTGGGGTGCCTGGCCAGGCCGTCTTTCCAAGGCAGCGCTCCCGCAGTTGATGTCGCTGGGAGCCATCACAGATGATGGTGAGTAATGCCACACTCATGACGTGTGGATTTTCGTCTGGTCCTCGTCATTTTGAGATCACTACGATAACTTCTCTGATTCGCTTACTTGTTTTTGTTTTTCCAGGGAACTGAGAGAGCTTTACTCCAGAGTGGCAGGCATCTGTCTCTGTGAGCGATATATACGGACACAATGGGAAAATCTTTATGCGGCTCTCATTTATTGCAATATATGTCTGCATGGTTTTGGGCCGTCAATCCTTCTATCATATTCTTTGTGTCAAATTAATATTTGCTAATGATTGGCTTGCCAACGCCTCGAATGTAAGCAAGAAATAGAGGCCTGTTACGTTTTTCTGTTGTTTGATTTGATTGTTGAAATATGAAGCTATAAGTGGTCTGCCTAGTCGCTGAAGCATATATTAAGATTGCAGGAAGTGATTTGTGATAAGGACTGGTGTATCTATTCAGTATGCTTTTTCTCCTATCAATAACATGCCTTAAATTTTAATAAAGTTACAATTGATTACAGATTCAATTTTCTGTTTCACTCATTACTTTCACTCTTTCGCCGGCTTTGATGTTTTAATTCTTTGTGACGAATGATTTAACTGGTACATTTATTCTCTCACTGCATTTTACTGTTACAGACAAGTTTCAAGTCATTAAAAGAAAAAGTTAAAAAGAACAAATCTGTAGGATGTTTGAAATGGAGTGTTGTACTTTTAGACTTTGTATGTCTATAGTTGTCCAATTTTTTTTTTCGTATAATTTTACAAGGAGAAATTTCCCTACATATATGATTTTGTATCAAGCGACCGGCCTATTTTTTAGCGCAAGAAACAAACTGTACATGCTTTGTACACAGTATCGTGCAATAGTAATCTTTAAGCATTGTGTAATTTAATAAAAGTTTCATGAATACATTTAATTTAATAGATTTGTATTTAATCTGGTAGTGTTATTAAGATTGAGACCAACATCTCTTGCAGCAGAGACCTGAGAACAAATAATTAAAAAAAAAATAAAATAAAATAATAATAATTATAATAATAATAAATAGAAAACGAAATAAATGAATAAAATAAATAAAAAATAAAATGATAAAATAATGTTAACAAAAACAGGCTGTGTTAAAAAAATAAATAAACCTAGATTTCAAGAATCTACTCATACTGCCCGTAATGTATCCTAAGCTCTATGACGTGTTGCCATCCAAGATAACATATTGAAAATCTTTAAGAAAAAAAAAATGTTTCCTCTTAAATTCTACTGCTAAAGGATGTGTCGGGTTTGAACTTTCTTTACATCCTGGTAACTGTATCCCCTTTACTGAGTGAAAGTGAAACCCAAGCTCATCAATGATTCAATGTTAAATCCAGTTGACCCAGAAGCCGTGGTGGACAGTCACAAGATGGCCACGCAGTCTGACTCAATAAACATGGACGTGGATTTAAATGTAAGTGGGTCTACTTTGTTGTTTAAGTAGAGATATCTTTAAGTAGTATAGTATTTAAGTCACTACATTTCAGACGCTTGATAATTTCAGTTCTGTGTCCTGTGATTTTGGATGGGTTTTTCCTTTGTCGGTACAGGCGATATCAGGCGGAGCTGAACCAGTAAGAGTGGGAGTTATACTCGTGAAAATCCCGGGTAACGTCACAGTAACTCAACCTTTTTTCACATCGAGGATCGTCAATGTTATATGCATTAGGCCACCGACCTCATTTGATGAGATTTTGTCTCTGGGACCCCAATCCGAGAAGATTTGGCAACCTTCTCCTGGAACCCTTCCTTCAAGAGGAAGGGTCTCCATTAAAAGTCCCAGCCAACATTTGTTCCCCCTCACGCAGGACACGCACCGAGTTTCCTTTTTAGATCACAGACATTCACATCAGAATCTATTATGGGATATTTAACTTCCTACATGTGTGCTCTCAGTATGACAAGTGAATTAAAAAGTAGACAAATCAAACATCCCCAGGTTAGGCGAGGTCATCCTAAATCAGCTCACTTTGATCACGAACCCCTCGCCACAATTACCCCCGGGCCTCATTGTGTTTCATTGTCACGACGACAGACGCGGTTAGCGGGGGCGACTGTGAAATTCATGAGGACGGACGTGGTGTGGACTACACGTCAGGAAGGAATGAGGTATAGGAAGCAATGACAATCTGTATTCGCACGGAATCGCTCAATTGCTGAAGTGTTTTCACGTCTTTTCCAATTAGGAGACGACTTCTTTCTCGTTCGAGGGAGCTGTAATGGCGGAGGACCTAGAGGTATCCAAAACATTTTAATAAGGTTATTCATGGCGATCCAACTGTCCGATTTTTTGTTTAAAAACACACAGCATTCTGGGAAATTTGCGTTTCCCGTCAAACATGCAGAGATTAGAGCCGGCGTTCCTCGAGCCTCTGGCCAAGTCCGTATCCTGCGGCTACACCGCAACCCTGCTGATCAGCGGAGCCCACAGACGGGGGAATTCGGGCACCCTGATAGACGATGACATCATCAAAAAGGTCAGGATTGAGGGGGAGGAAAACAAACAAAAAAAAAAAAAAAACGACTTCAACTTAAAAAAACAGAGCTTCCACATACTTATTTAAAGGCTGACTCATTTTACAGGTTCTTGGGATTTTATTCAGCCGCGTAGCAGAAATGAAAGAAGAAATATTCAGCGCTGTGTCGTTCGTTCAGGTAGCCTATCAGCTTATACAGCACAGTGCATCAACAGGACCTGGCGTACACCCGGGGTCCAGACTAATGTTCATGCCTTTTACTCGCTGGTAGTTTTTCTCGGATGACGACGCACTGGATCTCCTGAACCCTGAAAGACGTCGACTAAAACCTGTCTCCCACCCGGTCCTCGGATGGTATGCATGTCTGAATAACGTCCTCCAAGCATCCCTTCATCGATAAGCCGGTTACATGGACACCAATCATTTGTGCGAGCCAAGTTGCTCACATTGATTGATAATGGCCCCATGACAGTGAGCAGGTGGAGAAGATGGATGCATGATAAAGAATGGGATGTTTGAATGATCCCAATGTAGCTACTTCTCCTTTTTCAGCCTAGTGGAAGGGTTGTGCGAGGTGTCTGTGGGCTCGGCGGTAGAGGCCTGTGGTCTGTATGAGACAGGCAGACAGAGGCTGAGCGACACGGCAGCCGGCGTCGGCGGCAGGTGGGACGCCGGTCAGAAAGACTGTTTAATCCTCTAATTCCATTACTTTGACTGAAGTAATTACTACGCTCGAGTTAAGCTTGTAAGCGGCAGCTCTATCACTGCAGATTCTGCCAATCACGATTTATCAGTGTCCACATACATTCCTTTAGATGCAGTTCCCTGTTCTCAGTCGCCATTGAACAAAGGCTCCATCCGGAGACCGACGAGCTTGAATTCTGTCGTAGCAGACTACAGCTGTTTAGCTTGGCGGGGGGAGCAAGCCAGACCGGCCTCAAAGGGTAAGTCACATCGGTATCTATAGTTATGAGCAGGTTTACAGAACATAACCTCCTCTTTACCTGGCAATTTACCGGAAAAAAAAACAAAAACAATAACTGTACCTAATACCAAATAACCTGCACCTTTGTGCATAAACTGACTTGTAGCAAAACAACAAATTGTGTTAAATGTTGATGCCAACTGCTTTATATTCCTCAGTGTGAGCCCGATGGTTCGGGTTCTAGAGCAACTTCAACTTGGAACCACAGCCAGCACCCAAGTCCTTCCTTTCCTATTAACAGAAGCCTTGACGGGAAACAGCAGGACGTGTCTCCTTCACTGCATCCAACCACACGGTCAGAGGTCTTTATCTTTCACACAAAGATAAATTCCTGAAGGAGACTGGATTGGACTAAGCATGCACGGAATTTGTGTTGAATTGTTATGGCTATGCAATCTGTCAGGTCGCTTGGATGAGGAGACCCCCGCCGCCCTCACTTTGGCCCACAGGGTGAAGGGGCTGGTGACCAAGCCAACTAACGGCCGGTGGTGTCCGAAGGTAACCGAGCGAGACATTCGACTTAAAATACAAGGGCTGAGGTCCCTGATGATGTCACAGAAGGAACACGGAGGCCAGAACACTTATAGGCTAGCAGAGCTAACTGAAAACCTACAGGTAGGCGGGCACTTGGACTCTCTTACGAATGTCCAAAGTGGGCCATAGCAACTCCCTTGTCCTGGGTTTGGAGTTTTGAGGCTGATTAGCATTGTGTTATTCCTCCGGTAGATTGTGAAAGATCAGTCATGGGAGGAGAGGTGGGAAACATCCGAGAGGATAAAAGACAAAATAAAGGTCTGTCCTGAATGCTTTTGGTTTGGTTCTTTAACTTTATTGACTACCTTATTCTGACGCAACTTTACAGGTGTCTTGTGTGCCACTGGTTTCACAGGGATCCCGGTCAACAGAAGGAAAGTTGCACAGTGATGGTGATCCCAGCATCGATTCTACAGATGCCGCGGAAAAAATTAAATATTTACAGGAACAACTGAGACAGCAAATGGAGGAACATATCAGAGGTAGAACTGCCGCAGTGGGTTGAGAAAGTGGAATATGGAAACGACGATGGAAGGCTTGTCTGAGGACGACCTAGAGAGAGATATGTAACACTCAGGAATACCATCACCTTATATACAATGTGTTTTCCCCTCACAGAGGGCAAAGGGAGTGTGGAGAAAGTTCAAGAGAGACTTACTAGAATCCAGCAGCTACGAGAGCGTCTGAGAGAGGAGATGATGAAACAGGGGGTTGCCTCAGAAAGATCTGACCTCTCACAGCAGGTACCTGAAATTACCTGGTAAACAAAACCATTTGTGCACCATGTTTCATGGATATATCACAAGCCATTTGTTATGTTTTGATCATACCCCCAAAGACCTTATGGTTTTAAATTATTTTTCCTTTTATTGAAATGGTTGTCTGCTCCAGCCGGAATACAGCAGAGCGTCACAGCAGCGGAGGCAAATCAAGGAGGATCATGACAGACTTATTCAGGAGGAGGTAGAGAAGATGGAGGAAGAGCTGAAGCAGGCACAGGTACCAGTAAGATGAATAAACCAACAGTTCTTTATGACTTTATAACTACGCCTTTCCTCACTTTAGCAAGTATAACGTCACTTTTCTTCCAGGCGGAGGGCGTCCAACGGGAGCTGCTGGTGATGGCCAGGGAGAGGCAGGTCCTCACGCTGCAGATGGAGGCTCTGCGCACTGAGGCTCAGCTGGCAGAGGGAGACCTGGAGGAGCAACGTCGCAGGAACCAGCAAGAGCTGCTACATCTAAGAGAGGAGAGTCTCCAGGTGGGAATTATTTTGATCCATGTTTGAAAATAAGATTTTATTTTAAACAGCGGAATAAATTATCGCCTCGATCAATATGGATGACTTATGCTTACGTGCTACTGTTGCTACATGAAAGGAATTTGTCAGTGAAGAAGCACCTTCATCCTGTCTTTCCTGTGTCCCTAGGTGTTCAGAGTGTTTCGTCAAGTGAGCGAGGAGCAAAAGAGAACGATGGAGGGCAGGTACAGAAGTCTTCTGCTCGAAGCGGTCCAGGACGCCGTCTACCTGTCCGCCCAGAACCAGCAGCTACAGGCCGACAACACGCAGCTTCGCACCGGTGAGCTTAACTACTCATTGAGACGAGTGTCTTGACTTCCATTCAATCTGGGCGTCTGTGCTTGTGAGTAGTGTTATTGCGCTAAAATTCACTTGAAACTTGGTGGTGTAATGAAAATACATAGCACAGGACGCTCACTGACTGCCGTTTACCCTTTTGCAGTTACTGTCGAATTTAATATTTATTTTATCTCCTGTAGCACTAGGGGAGCTGAAGGACGCTCAGACCACACGAGGTGACACCAAGGCCGAAATTTCACCTCAACAGTGAAACAGCGTATTCTATTAAGAATTTCAGTTGATCAACAAGTCTCTCAAATGAATTGACTTTCTGCGCCAAATGAATTGAATCTTTCCAGAACATTGACTTTATTGTGTCCCCATAAGATATCACCCCTGTAACAATATTAGACCACTCTTGACAATTTGTTGGCATAAAGACAGAATTGCATGATTCTTCGATTAAATTTCTCCTGTACCCGCATGAGAATAAAATGGATGCTATTTTACATAACGTTTAAAGAAGAAAAAAAACAAAAACAAAAAAAAAACAAGGCAACTGAAAACACCAAATCAAATCAAATTTTGGTATATGATGGCATAAAACTGATGGCATCTGCAGATGGTACTTGTAGGCTCAAATCTGAAATACAGCTGATTAACTAAATCAGGACAATATATATACTTTTTTTTTTTTAACAGAAGTGGGACATTTGATGTTAGCCTAGATAATAGACAATGACAACTAAAAATAAAAGAAAAACTAAACCATTTCAGAAAGCAAACCTGACCTATTTAGATAAAAATAACTGAAAAAAACAGCTGAATTGAAAAGCAGTTTTGGTGGACTGAAGCGCAAGATCCTTTGGGTTGACTTGGGCCTCCAAAGAGGGCTTAGCGCTTTACGTGTAATATAAATAAGCTGCCAGTCCGATCAGAGATACAGGCAGGAGGAAGAAAGGCGTGAGCTGCAGCCCCATGATGGCCCAGGACAGCAGGGCATTGACCAGCATGGAGCTGGAAATGACAAAGAGTCTGGTGATCCCGCTGCCGTGCTTCAGCACCACAGACATCAGGAGTCCGTTAGCCACCTGTCCAGCTATGATCGCCCACACCGCCCCTGAGTAACCTCCCAGGAAACCCCTTTCGCCTGCTCCACTGGAAAGGTGGCACACTCCGTTGATAACGACTCCAAATACATACAAGTAGAGATTCTGCAAGCTCAGGGGCAGCTGCTGGCTCTTCAGCACCCTTTCTGTGTAGACAGCTGCCAGCCCTGAAACAAAGCAGTATAGCAGCACCAGAAAAAGCCCCAAGCCTGTGACGTGAAGCCTGGTGCTCGCTGCAGAGTCACCCTGCTCAGGGTCCCCCAGGTCCAGGCTGCTGTAGCTGTGGCAGACGCCGGCCCCCAGGAGGAGACCCAGGGCTGACCACTGAGCCAGACGAAGCCTCTTGCCCAGGCAGACGGAATACAGCAAGGCAGTCGAGGCAATCTTGAGGTTGCTGAGGACCTGGAAGGAGCTCGGGTCCATGTAGGCCTGCATGAGAACCACTAGGTTGTTGTTGAGTGCGTAGAGGACGGCGGGGACTGCGTAAGGGGCCACAAATATCAGGGAAGGAGGAGCAAACAAGGCAGATGTGTCCCCGGTCAACAGGAGGGTGGCCAGGGAGATCAGCAGTTTGGCCAACTCGATAATGACAACACAAGAAGACGTGCTAAAAGGAACTCGACCATTAACCTTGGTCAATGCAATGAGCGGTGCGTGGGAGCCATAGATAAGGATCATGAGGCCGAGGAGCACAGTCCACTGAAGTCCCTTCATCCACCTTCTCCGCCTGGGGAATCCCACATTTGGGATCACAATCATTCCAAATACTGCACCTTTTACACTTTGGTAACTTCTGACAGAGTTTGTGGACTTAACAGATACACTTTGAGTCATACATAATTACAATGGTTCTATTATAGAAATGAAGATATGTGCGTACCCCAGCCATTGAATATGACCAATATAAAATGATTTGATAAAATTTGATAGATAAGCATTTTCTGATGAAGCTAAATACTTAAGCAAGTGGATAAATCTATGGGATAACACTATAGATCTTATCGACATCAATTGTCATTTGAATTTATCAATAGAAAATACAATAACAGCAAAACTATAAATCAAGCCAGTGTTGCAATTTGATTCCCATATTAAATTCAAGTTTCATCATTATTCCATAGTCCAGTTAACACAAGGGTTTCAATTATTCTCTATTCATGGGTCAATAGCAAACCAGCATCGGAAATTCAAGTTACTTGCCTTTGTTCCCCTATACTGCAGACAAAAGATAATGTGGGATAGTCATCGCTCAAAAATTATTTAGGTCCTTTTTTCAAGTTCTGCAAACAATCTTTCACTGTCCTTTCAATGTTGGGCACTTGGTAGCTCTGAGCTGCATAAATGCCGGTGATTGTTCCAATAAGCACTCCTAAAATTGCAGAGGAGCGCAACTTGGCCACCACATACCCAGCCAGAAAACCCTTTAGAAAGGGAGATCCCAAGACAGAGCCCCCCTAAAGCAGGGGGAAAAAAAAAAACAGTATAAAAACGATGAAGAAATGTGACAAAGATCAGTTTATTTAAAATGTGTAACCGATCAAAAGTCAGTGCATTTCCTTGATGCACAACAACGGTTATTTGCAGGCTGATTACGAGTTCAAAGTTAATTCAAGCCGCACAACGTACTTGAGGTATCCCGATGGAGACTTCGTTCTCTACCCGCTTCTGGATCTCCTCCAGTTGGCTCTTGAGCTGCGTCAGATCTTTCAACTGCTTCAGAGGATCCTGGAAGTGTAAAGGAATCCAAAACATTACAAAAACAACATTGACACAACCCATTGAACACTGTG

The utg7180000026784 sequence encodes a full-length canonical CD74a molecule:

METPEQAPLAGSQEALLPSARVRGGSNSRAFKVAGLTTLGCLLLASQVFTVYLVVNQKQA

IHTMQKDSETLRKQLTRSSQTPPMKMYMPMPSLPLLKGFQTEAETAKTSVKKQEDTVATV

GLETQVERLLQDSVLPRFNASTFFDNLQSLKEQMEESEWKGLESWIRHWLIFQKAQEKPP

APPTEKPALPLQSKCQMEASMSGRRLGFFQPQCDESGHYRSVQCWHSTGFCWCVDDNGTA

IEGTYSRNRPHCQRGRLSKAALPQLMSLGAITDDGN

**3.4 *Typhlichthys subterraneus***

**For the non-gadiform fish *Typhlichthys subterraneus* no intact *MHC IIA*, *MHC IIB* and *CD4* genes were reported.**

*Typhlichthys subterraneus* is a non-gadiform fish investigated by Malmstrøm *et al.*^1^ which is closely related to the gadiform fishes, and hence important for deciding the phylogenetic stage at which functional MHC class II system genes were lost.

In summary, our findings for their Table S7 listings are:

**MHC IIA:** Table S7 mentions utg7180001268240. This sequence encodes a classical type MHC IIA sequence, except for its transmembrane and cytoplasmic regions, possibly because the unitig is too short. We were unable to find *Typhlichthys subterraneus* unitigs or scaffolds reported by Malmstrøm *et al.*^1^ with full-length classical *MHC IIA*.

**MHC IIB:** Table S7 mentions utg7180001679958. This sequence encodes a classical type MHC IIB sequence, except for its transmembrane and cytoplasmic regions, possibly because the unitig is too short. There is an unusual intron within the β2 domain coding sequence which first has been described for neoteleost fish classical MHC IIB genes in pufferfish (Lim *et al.*^3^). We were unable to find *Typhlichthys subterraneus* unitigs or scaffolds reported by Malmstrøm *et al.*^1^ with full-length classical *MHC IIB*.

**CD4:** Table S7 mentions utg7180001171865. However, this unitig only encodes the middle region of a CD4-1 molecule, possibly because the unitig is too short. We were unable to find *Typhlichthys subterraneus* unitigs or scaffolds reported by Malmstrøm *et al.*^1^ for which a full-length canonical *CD4-1* was predicted, although depending on the leader sequence the scaffold sequence scf7180003323411 might include such full-length gene (not shown).

**CD74a:** Table S7 mentions utg7180000050069. This unitig probably encodes a full-length canonical CD74a.

**CD74b:** Table S7 mentions utg7180000242295. This unitig only encodes part of CD74b, with the coding part for the N-terminus missing because the unitig border maps within an exon. We were unable to find *Typhlichthys subterraneus* unitigs or scaffolds reported by Malmstrøm *et al.*^1^ with full-length canonical *CD74b*. The scaffold scf7180003318360 covers the entire region but has a gap (denoted “NNNNNNNNNNNNNNNNNNNN” in the sequence report) within the predicted *CD74b* coding sequence (not shown).

Below the respective unitig sequences are shown with their relevant protein coding capacity:

***Typhlichthys subterraneus MHC IIA***

>utg7180001268240 length=1232 num_frags=85 Astat=83.00

(the complementary sequence is shown)

CACACACACACACACACACGCATACACACACACACACACACACACCTCCAGACTCCAGTCTGCCAGATACAGATGATGCTTTAATCTGATTGGTTGATTCAACATGTGAGACTTTAAACAAGTCCTTCATTTCACCAGTCAGGACGGAGCTGAACACACAGCTGGTAATAGGGTTATACTGGTCTCTACTGGTCTACTGGTCTACTGGTCTCTCCTGTGGTGGTGTAGTGAAGATGAAGGTGCTTCAGACCATAGTCTTCCTCTCATGTGTGTTAACAGCAGCAGCAGATGGTCAGTGGATTGTTTATATTCTACTTTACTACTAATGTTTACCTCTCATGTGTAATATTGACCTCCCTGTTTATAATAGAACCATTAATAATCACAAGAAATAATGAAGAAACTGTTGTTTAGATTAAAGGTTAATAATATGTGTTATTGTGTGTTTACAGACAAACATGTAGATCTAAATATTGATGGCTGTTCCCTAACTGATGGAGAGGCCATGTATGGTCTGGATGGAGAGGAGGACTGGTATGCAGACTTTGTCCACAAGAGAGGAGTCGTGCCCCTGCCCCCGTTTGTAGATGAAGTCACCAAACCAGGAGGTTATGAACAAGCTGTGGCCAATCAGGCCATCTGTAAGGATAACTTGGGTAAACTTATGAAAGCAATGAAGGACGTCCCACTGGAACAAGGTAATTACACATCACAGTACTTTAATGTGTTTATAAACACATTAAAGTACTGTGATGTCATGTTATAACTGCTGCCAGACTGGATATGATTTACTACATTATGTAAAGGATATACACGTATGGATCTGTCTCCTGACATGTTCTCCAGATGTTCCTCACCATAGATCTGTCATGTTGTCCAGATGTTCCTCACAGTTCCATCTACACCAGAGACGTGGTGGAGGTGGGGGTGCAGAACGTCCTGGTGTGTCATGTGACAGGCTTCTACCCAGCACCTGTCCAGGTGAGCTGGACCAGGAACAACCAGAACGTGACAGAAGGTTCTAGTCTCAACACACCCTACCCCAACAAAGATGGAACCTTCACCCAGTTCTCCAGCCTGAAGTTCACCCCACAACCAGGAGACATCTACAGCTGCTCTGTGTCACACCCCTCCCTCAGCGAGCCAGTCACACGAGTCTGGGGTACAGTACTAGTACTGCATGTATACTATAGTACACACTATATAGTACACACTATATACTACTTACTGTG

The utg7180001268240 sequence encodes the leader sequence plus entire ectodomain of a classical-type MHC class IIA molecule, but does not encode the transmembrane and cytoplasmic regions. The translated sequence shown together with the coding sequence is:

10 20 30 40 50 60

CACACACACACACACACACGCATACACACACACACACACACACACCTCCAGACTCCAGTC

70 80 90 100 110 120

TGCCAGATACAGATGATGCTTTAATCTGATTGGTTGATTCAACATGTGAGACTTTAAACA

130 140 150 160 170 180

AGTCCTTCATTTCACCAGTCAGGACGGAGCTGAACACACAGCTGGTAATAGGGTTATACT

190 200 210 220 230 240

GGTCTCTACTGGTCTACTGGTCTACTGGTCTCTCCTGTGGTGGTGTAGTGAAGATGAAGG

M K V

250 260 270 280 290 300

TGCTTCAGACCATAGTCTTCCTCTCATGTGTGTTAACAGCAGCAGCAGATGGTCAGTGGA

L Q T I V F L S C V L T A A A D

310 320 330 340 350 360

TTGTTTATATTCTACTTTACTACTAATGTTTACCTCTCATGTGTAATATTGACCTCCCTG

370 380 390 400 410 420

TTTATAATAGAACCATTAATAATCACAAGAAATAATGAAGAAACTGTTGTTTAGATTAAA

430 440 450 460 470 480

GGTTAATAATATGTGTTATTGTGTGTTTACAGACAAACATGTAGATCTAAATATTGATGG

D K H V D L N I D G

490 500 510 520 530 540

CTGTTCCCTAACTGATGGAGAGGCCATGTATGGTCTGGATGGAGAGGAGGACTGGTATGC

C S L T D G E A M Y G L D G E E D W Y A

550 560 570 580 590 600

AGACTTTGTCCACAAGAGAGGAGTCGTGCCCCTGCCCCCGTTTGTAGATGAAGTCACCAA

D F V H K R G V V P L P P F V D E V T K

610 620 630 640 650 660

ACCAGGAGGTTATGAACAAGCTGTGGCCAATCAGGCCATCTGTAAGGATAACTTGGGTAA

P G G Y E Q A V A N Q A I C K D N L G K

670 680 690 700 710 720

ACTTATGAAAGCAATGAAGGACGTCCCACTGGAACAAGGTAATTACACATCACAGTACTT

L M K A M K D V P L E Q G

730 740 750 760 770 780

TAATGTGTTTATAAACACATTAAAGTACTGTGATGTCATGTTATAACTGCTGCCAGACTG

790 800 810 820 830 840

GATATGATTTACTACATTATGTAAAGGATATACACGTATGGATCTGTCTCCTGACATGTT

850 860 870 880 890 900

CTCCAGATGTTCCTCACCATAGATCTGTCATGTTGTCCAGATGTTCCTCACAGTTCCATC

V P H S S I

910 920 930 940 950 960

TACACCAGAGACGTGGTGGAGGTGGGGGTGCAGAACGTCCTGGTGTGTCATGTGACAGGC

Y T R D V V E V G V Q N V L V C H V T G

970 980 990 1000 1010 1020

TTCTACCCAGCACCTGTCCAGGTGAGCTGGACCAGGAACAACCAGAACGTGACAGAAGGT

F Y P A P V Q V S W T R N N Q N V T E G

1030 1040 1050 1060 1070 1080

TCTAGTCTCAACACACCCTACCCCAACAAAGATGGAACCTTCACCCAGTTCTCCAGCCTG

S S L N T P Y P N K D G T F T Q F S S L

1090 1100 1110 1120 1130 1140

AAGTTCACCCCACAACCAGGAGACATCTACAGCTGCTCTGTGTCACACCCCTCCCTCAGC

K F T P Q P G D I Y S C S V S H P S L S

1150 1160 1170 1180 1190 1200

GAGCCAGTCACACGAGTCTGGGGTACAGTACTAGTACTGCATGTATACTATAGTACACAC

E P V T R V W

1210 1220 1230

TATATAGTACACACTATATACTACTTACTGTG

***Typhlichthys subterraneus MHC IIB***

>utg7180001679958 length=2504 num_frags=147 Astat=208.00

CTGAGTCCTGGAGAGAGACCTGTCTACATCTGACCTGTCTGTCTCTAAGAAGACCTGTCTGTCTAAGAAGATACCTGTCTGTCTTTAAGAAGAGAGACCTGTCTGTCTCTAAGAAGAGAGACATGTCTTCAGTCAAGCTGTGTGTGTGTGTCTTGTTGCTCAGCAGTTTGTATGGAGCAGGTAACTGTTATTAATACTAACATGATTATTAGTATAGTGGTGACTAGAGCAGAGACAGTAGCATGTAGTATATACAGTATAATACAGTATATACTACATGTATATACAGTATATACTACATGTATATACTATGTATATACATGTAGTATATACTGTATATACATGTAGTACATACATGTAGTATTCTAGTGTGTACTTTATTATGCAGACACAATAAACATTGTTTTTCTTTTCAGATGGATATTTAAACTACAGGGTGGCAGACTGTGTGTTTAACTCCACTGAGCTGTCTGACATCGAGTACATTGAATCTTATTACTACAACAAGTTGGAGTTCTTAAGGTTCACCAGCACTCTGGGGAGGTTTGTTGGATACACTGAGTTTGGTGTGAGACAAGCAGAACAAGCTAATAAGGATCCTTCAGAGATAGCCAGGAGAAAGGCTCAGGTGGAGGGTTACTGTAAACACAACATTGAGATCTGGTACAGAGCCATCCTGGATAAAACAGGTCAGACACTTTACCTTCTACATGAATAATCACTGACTCATTATAATAATAAGACATGATTATTCTCTAATTATACTGAACATCATAATATGAAGCATGTTTACTGTTATGATAATATCATGTTAAACACACACACACACACACACATGATGTGTTTACAGCAGGACAACCTGCTGAGGTAGGTGAAGAACCTACACACACACACAGCTGTCCTGTTTATGGTTTACGTAGCTCATTGGCTCCACCTAGTGGCACACTGCTGGTCCTCCCTCCCCTCCTCCGCCTGGCCCGGGGGAAGGGGGGTGGAGGGGGGGACAGGCAGACAGACTGAATCAACAGGATTGAAAATCTTCTGGCTCTGTCGCCATCACTGCAGTACCTCACTCCGCCTGTAGCGCCCCCCATACCCCCTATCCATTATAATGGGATACATGTACGGCAGCCATACATACTCTTCCTCTACAAATATAAAGCTAATAAGCTAGTAAGACATATGGCTACAGCTTTATATTTGTAGAGGAAGAGTATGTACGGCAGCCATACATACTCTTCCTCTACAAATATAAAGCTAATAAGCTAGTAAGACATATGGCTACAGCTTTATATTTTTAGAGGAAGAGTTATAACACAAATTCACCCACCAGTTGAACAGGAAGACGAGCTATAGAGACTAAAACGTTTTATTGACCAAGCAGTACATTGTTGCTGTGAAGTCAGACATTTTAACATTGTAGTCTATGAGGATTGACTCCCTTTTGCCACCAGCCTCTAGTGGCCATTTGATGAACTGCAGGGTTTGGTAATGGAGGTTCCCCCTTGCATTCAACACACCTGTGCACTGCACTGTATTATATACTTTACAATCAGTTTTATCACATTAGTATTAGTACATTAGTACCAATTATGGGTTGTGTGTTAACGATGACAAGTGTTGTAGTTGTGTAACTTCTGCTGCATCTTGTAACACAAGTGAATCAGTGTTAAAATGTGTTTTATGAGTAAGAATGTCTATGCGCTTTGCAATAAGAGGGTTTAGGCCACTGAGCACTTGGTTTAGAGAATGGGGTTAAGTGTTGTAGCAATTCAGAAAAACTAATAATAATATTGTGTCATCCTAGCTAAGCCCTATGTGAGGCTGAGCTCAGTGTCGCCCCCTGGTGGCAGACACACAGCCATGCTGGTGTGCAGCGTCTATGACTTCTTCCCTGAACAAATCCATGTCACCTGGCTGAGAGATGGACATGCTGTCACCTCTGATGTCACTTCCTCTGACGAGATGGCTGATGGTGATTGGTACTACCAGATACACTCCTACCTGGAGTACACACCCAGGTCAGTACCAGTACCCTAACCAGTATAGTACTGATACTACAATACTACTGTAGTACACCAGGTCAGTACTAGTGATCTAACCCTAACCCCTGGAGAAACAACTGGAGAAGAAACTCATTGTCTTATCCAGTTAACCATGTTAATCTGCTAAATTAAAAACCTCTGGCTGAAGTAACTTCTATGGTAATCTGTGTCCCTGGATTCCTAGATCTGGAGAGAAGATCTCATGCATGGTGGAACACAAAAGCCTGCCTGACCCTCTAGTGGAGACATGGGGTTAGTACACACACTATACATATAATAATACATGATGTGACCCTCTAGTGGAGACATGGGGTTAGTACACACACTATACATATAATAATACATGATGTGACCCTCTAGTGGAGACATGGGGTTAGTACACACACTATACATATAATAATACATGATGTGACCCTCTAGTGGAGATATGGGGTTAGTACACACACTATACATATAATA

The utg7180001679958 sequence encodes the leader sequence plus entire ectodomain of a classical-type MHC class IIB molecule, but does not encode the transmembrane and cytoplasmic regions. The translated sequence shown together with the coding sequence is:

10 20 30 40 50 60

CTGAGTCCTGGAGAGAGACCTGTCTACATCTGACCTGTCTGTCTCTAAGAAGACCTGTCT

70 80 90 100 110 120

GTCTAAGAAGATACCTGTCTGTCTTTAAGAAGAGAGACCTGTCTGTCTCTAAGAAGAGAG

130 140 150 160 170 180

ACATGTCTTCAGTCAAGCTGTGTGTGTGTGTCTTGTTGCTCAGCAGTTTGTATGGAGCAG

M S S V K L C V C V L L L S S L Y G A

190 200 210 220 230 240

GTAACTGTTATTAATACTAACATGATTATTAGTATAGTGGTGACTAGAGCAGAGACAGTA

250 260 270 280 290 300

GCATGTAGTATATACAGTATAATACAGTATATACTACATGTATATACAGTATATACTACA

310 320 330 340 350 360

TGTATATACTATGTATATACATGTAGTATATACTGTATATACATGTAGTACATACATGTA

370 380 390 400 410 420

GTATTCTAGTGTGTACTTTATTATGCAGACACAATAAACATTGTTTTTCTTTTCAGATGG

D G

430 440 450 460 470 480

ATATTTAAACTACAGGGTGGCAGACTGTGTGTTTAACTCCACTGAGCTGTCTGACATCGA

Y L N Y R V A D C V F N S T E L S D I E

490 500 510 520 530 540

GTACATTGAATCTTATTACTACAACAAGTTGGAGTTCTTAAGGTTCACCAGCACTCTGGG

Y I E S Y Y Y N K L E F L R F T S T L G

550 560 570 580 590 600

GAGGTTTGTTGGATACACTGAGTTTGGTGTGAGACAAGCAGAACAAGCTAATAAGGATCC

R F V G Y T E F G V R Q A E Q A N K D P

610 620 630 640 650 660

TTCAGAGATAGCCAGGAGAAAGGCTCAGGTGGAGGGTTACTGTAAACACAACATTGAGAT

S E I A R R K A Q V E G Y C K H N I E I

670 680 690 700 710 720

CTGGTACAGAGCCATCCTGGATAAAACAGGTCAGACACTTTACCTTCTACATGAATAATC

W Y R A I L D K T

730 740 750 760 770 780

ACTGACTCATTATAATAATAAGACATGATTATTCTCTAATTATACTGAACATCATAATAT

790 800 810 820 830 840

GAAGCATGTTTACTGTTATGATAATATCATGTTAAACACACACACACACACACACATGAT

850 860 870 880 890 900

GTGTTTACAGCAGGACAACCTGCTGAGGTAGGTGAAGAACCTACACACACACACAGCTGT

910 920 930 940 950 960

CCTGTTTATGGTTTACGTAGCTCATTGGCTCCACCTAGTGGCACACTGCTGGTCCTCCCT

970 980 990 1000 1010 1020

CCCCTCCTCCGCCTGGCCCGGGGGAAGGGGGGTGGAGGGGGGGACAGGCAGACAGACTGA

1030 1040 1050 1060 1070 1080

ATCAACAGGATTGAAAATCTTCTGGCTCTGTCGCCATCACTGCAGTACCTCACTCCGCCT

1090 1100 1110 1120 1130 1140

GTAGCGCCCCCCATACCCCCTATCCATTATAATGGGATACATGTACGGCAGCCATACATA

1150 1160 1170 1180 1190 1200

CTCTTCCTCTACAAATATAAAGCTAATAAGCTAGTAAGACATATGGCTACAGCTTTATAT

1210 1220 1230 1240 1250 1260

TTGTAGAGGAAGAGTATGTACGGCAGCCATACATACTCTTCCTCTACAAATATAAAGCTA

1270 1280 1290 1300 1310 1320

ATAAGCTAGTAAGACATATGGCTACAGCTTTATATTTTTAGAGGAAGAGTTATAACACAA

1330 1340 1350 1360 1370 1380

ATTCACCCACCAGTTGAACAGGAAGACGAGCTATAGAGACTAAAACGTTTTATTGACCAA

1390 1400 1410 1420 1430 1440

GCAGTACATTGTTGCTGTGAAGTCAGACATTTTAACATTGTAGTCTATGAGGATTGACTC

1450 1460 1470 1480 1490 1500

CCTTTTGCCACCAGCCTCTAGTGGCCATTTGATGAACTGCAGGGTTTGGTAATGGAGGTT

1510 1520 1530 1540 1550 1560

CCCCCTTGCATTCAACACACCTGTGCACTGCACTGTATTATATACTTTACAATCAGTTTT

1570 1580 1590 1600 1610 1620

ATCACATTAGTATTAGTACATTAGTACCAATTATGGGTTGTGTGTTAACGATGACAAGTG

1630 1640 1650 1660 1670 1680

TTGTAGTTGTGTAACTTCTGCTGCATCTTGTAACACAAGTGAATCAGTGTTAAAATGTGT

1690 1700 1710 1720 1730 1740

TTTATGAGTAAGAATGTCTATGCGCTTTGCAATAAGAGGGTTTAGGCCACTGAGCACTTG

1750 1760 1770 1780 1790 1800

GTTTAGAGAATGGGGTTAAGTGTTGTAGCAATTCAGAAAAACTAATAATAATATTGTGTC

1810 1820 1830 1840 1850 1860

ATCCTAGCTAAGCCCTATGTGAGGCTGAGCTCAGTGTCGCCCCCTGGTGGCAGACACACA

A K P Y V R L S S V S P P G G R H T

1870 1880 1890 1900 1910 1920

GCCATGCTGGTGTGCAGCGTCTATGACTTCTTCCCTGAACAAATCCATGTCACCTGGCTG

A M L V C S V Y D F F P E Q I H V T W L

1930 1940 1950 1960 1970 1980

AGAGATGGACATGCTGTCACCTCTGATGTCACTTCCTCTGACGAGATGGCTGATGGTGAT

R D G H A V T S D V T S S D E M A D G D

1990 2000 2010 2020 2030 2040

TGGTACTACCAGATACACTCCTACCTGGAGTACACACCCAGGTCAGTACCAGTACCCTAA

W Y Y Q I H S Y L E Y T P R

2050 2060 2070 2080 2090 2100

CCAGTATAGTACTGATACTACAATACTACTGTAGTACACCAGGTCAGTACTAGTGATCTA

2110 2120 2130 2140 2150 2160

ACCCTAACCCCTGGAGAAACAACTGGAGAAGAAACTCATTGTCTTATCCAGTTAACCATG

2170 2180 2190 2200 2210 2220

TTAATCTGCTAAATTAAAAACCTCTGGCTGAAGTAACTTCTATGGTAATCTGTGTCCCTG

2230 2240 2250 2260 2270 2280

GATTCCTAGATCTGGAGAGAAGATCTCATGCATGGTGGAACACAAAAGCCTGCCTGACCC

S G E K I S C M V E H K S L P D P

2290 2300 2310 2320 2330 2340

TCTAGTGGAGACATGGGGTTAGTACACACACTATACATATAATAATACATGATGTGACCC

L V E T W

2350 2360 2370 2380 2390 2400

TCTAGTGGAGACATGGGGTTAGTACACACACTATACATATAATAATACATGATGTGACCC

2410 2420 2430 2440 2450 2460

TCTAGTGGAGACATGGGGTTAGTACACACACTATACATATAATAATACATGATGTGACCC

2470 2480 2490 2500

TCTAGTGGAGATATGGGGTTAGTACACACACTATACATATAATA

***Typhlichthys subterraneus CD4*** *(CD4-1)*

>utg7180001171865 length=3702 num_frags=350 Astat=234.00

(the complementary sequence is shown)

GAATCTGCACATTGTCAAATCAGGGCCCACCACAACCTGGCATACCCCCCCCTCTCTCTCCTCTCATCCTTCTTTTCATCTCATTCAAAGCCCACCACAGCCCTCCTACGCTCACGTTTCTGAACTCTTCCTCTTTTACCATTGTCAACCTTTCTAAATCTGAGACCACCACAAACCCCTCTTCCACCCTCTGTAGACGGTGACAATGATTTTGACATCTTGCAGCGGAATTTAGCATGCCTGCAATGTTCAATGACAAAACACAACAGAGTGAATTTGTATTCTTTCACATATTTGCCATTTTCAACGTTTTATACTAATGCTAATTAATGCTATGTAATTAAAGGTGTTAATTACATATTTATTTTCAAAAACTATGCAGGAAGGATTAAGATGATTCTATGTAATTAACTAATACTTACCCAGTAGGATATGGCAGATATTAGGTATATTGTATATATTATATATATACTCTTACATCTCTATGGTGTCACCACCGCCGCGATGAAGCTGCTGTAGGCATATGCTGCACTGTTGGAGTCATTGCATCTTGTTTCAATATATTGCTTGTTTGAATGTATTGTAGTATTATGTTGTAATTATTAGTTAATTGTTATTATACCTATATTTTTCAGGCTTGGAACAAAAATGAAGAATTGTATCCAGTCACTAATCATCTTTTGTGCCATGATAACAACGGGTATGATACTGTATATTATACCATGTGATTAGCGCAATCAGGCGTTCACTGTTCTTAAATGTCTGATTTTCTTCCCATTCAGAAGCTGCTCAGAGGATCTGTATATATGCACAGGTTGGTGAAACGGTCACCTTGTCTTATAGCCCACAAAGAGAACAAAGCCAAATGTACCTGTACTGGTTCAGCAGCACTGGAGGAGACTCTTATATTATCTCACGGAATTCATTTGGCCACATCTCTAAGACAGGTGTGTGTGTGTGTGTGTGTGTGTGTATGCGTGGAACATTGTTAGTCAAACTACCATGAATACAATCTGTGTAATAATAATAATAATAATAATAATAATTATAATATTTGCATAATTTATTCCAGATGGGCAGTGGAAGGACCGGCTGTCATTAATTGACAACTCTCTGACCATCAAAAATCTCAAACCAGATGATTTTCAAACATTTACTGTACAGTTGAAGTTTAACAGGGAGACTAGATTCACCGCTACCTATGACCTCAACAGGGCCAATGGTAAGAGTAAACATGCACACACCAACAACTAATGTGGTTGTCAATATTTTCAAGCCATAGTTTCCCTGGCCTATACAGTACAATAGCATTATTATTATTATTATTGTATTAATGACAATAGAACACAATTAAAAATAAGTTTTAGATTTATTCATATGCTACTACAGGCTAATTATTTTTTGCAAATGTTTCAAAAGTCACAGTAAAAAAAGTTATAATTCAATGTGATGATACAATGATACAGTGATATTATGAATCTTTTATTTATATTTGATTACATTTTGCTTTTACTCTTGTTCCATGTCATATCAGTGAGTGCACAACCAAGCTCTCTTTTGCTGGCTGGTGAACCCCTCACACTGTCCTATACAAAGATGTCTCAAGGTTGTAATCAGCCAAATATCCACTGGCTCAATCCCCAGAGGATTAAAATGGTCCAACCCAGTTCGTACACATTAAATATAAAGCATACCAGTGCCCATGACAGTGGCGAATGGACCTGTGTTTTGACACACAATGGGAAAGAATCCGAACTAAAAACTACTGTTACCGTTGTGGGTAAGTGAAGCATATTTATTTGATGACGATTATTTGTATTTAGGATGATACATATCATTATTCCTAATATTGATTTTGGCATGACTATTACTGTTATTATTAGACCTATGTGTGATGTCAACAATGTGTGTTAGCGTGCGTGCATGTGTGTTGATCATTCTGTAACTGTATATATATATATATATATATATATATATAACTATAAGTGTCAACATCAGCTACAATTACATTGTCCATTCTATCTTCCTATCCAGACCTTTCCCCGGCCCCGGTGGATCCTGTCTATGTCCCTCTGTCCACCACCACCCGTCTACGCATCCCTTGTTCCTTTCCTCCTGAGCTCACCTGGGAACACCTCAGGAGCAAGGGCATAGTGGGAGGGCACTGGAAATTCACCCCCAACTGGTCCCCTCAGCCCGGGGGCCCTCAGAAGCTCTTCTCCCTCTCTGTGGAGGATCCGCTGGCCTGGAAGGAGGAACAGAAACGAGGGCTGACCGGAGCAGACCTCATGAAAAACAACCTGTCGCTGACGAAGACGTCTGTGACGGAGGAAGACAGGGGAACGTACACCTGCGCTCTGGAGTTCCCACATGTAACACTGAGCAGGAGCGTACGCGTTGAGGTGTTACAAGGTACGCCAAAAGCTGATGCACTCCTCAACTGTCAGATTAACCACGCACACAAGATGGATCACTTTTGATTTCTTTGTTTTGCTCGTTCTCCGAGGCCAGCACTGCCTCTTCATATATCTTTGCTTTATCTCGGTCTGCCTTGTTTTGGGTCTGAAGCATTGCATCAGGACCACTATATATTTTATGCTTTGAAATTTTTCATTTTACAACGGTGGTTCATACAGTATGTATTTTAATTATGATTTTAAGTAAATATATATTTGCATGCTTCTATGCCATATAAGATGTAGTTTCTTTTTTTAATTTCTTTTTTTTTTTTTTAAAGCAAGCAATTAATTACTTCTGATTGTGGGTCGTGGGTCATTGCTATCGATAACAAGTGCGTCTTCTGTCCTGCCATGCCTGGCCAGGGTTCTTGTGCATCTGTATCTGTTGTGAGGTTTGTTCGGAACAGTTTCCCTGGTGCGACATGTGTCTGCCTTCACTTGTTTTAATAGATAAAGCAGAATGAGCATTCACTCTGCCAAAAGAGTTGGGATAACCAATACATTTTAATTGGATACTGCTGACCAGTTTTTTTTTATATAAATGTTTTTTTTTCCTAATTTAATACACTTTATGCTGATTGGAAATGCATTTAGAATTCCAACAAACCTTTACTACAGGTACAGGTAGGGTTTGGTCACTACTTAACTATTTACATCTTTCTTTCTTTATTTCTTATCTGCAGTCCTCAACTCCTTGGAGTCTGTGCTTGTTGGGCATCAAGTCAACCTGACCTGCAGCCTTGGCCACAGCCTGACCTCTGGTCTTCAGGTGAAATGGATCCCACCCAGGTCATACGTGTCCGATCTGAGGCCTGTGGCACACCATGCCACCATCTCCATCCGGATAGAAAGTGAAGACCAAGACAAATGGAGGTGTGAACTGCTGGGGAACGGATCACAGCTGGCGTCAGCCCAAATCAAGCTGAAGATAGGTGAGCACAGGAAATGAGAGGAACAGGAAATACACTTGTAAGAAGATGTGGTCACATCTTTTAATCTGTCTTCAATTTAATGTATATGCCTAATGTATGTGTTTTTATGGTGCTTATGTGTCTCTTCTGTGTACAGAGAAACCTCCACTAAACATATGGCTGCATGTGACCATCTGTGGCACCACAGCCATCTTCATCCTACTGCTCTTTGTTACGGTTATCCTTGTTCGAAGACACAGACAGGTATTTGCAGGCTTTGCCATTCCGTCATTGTCAAGTTTCTCCTGTCTAGAATATTCCTTATGTCTTAT

The utg7180001171865 sequence encodes three Ig-like domains (2-to-4) and the transmembrane domain of CD4-1. Information is lacking about the leader sequence, Ig-like domain 1, and the cytoplasmic tail. The sequence encoded from the unitig is:

SAQPSSLLLAGEPLTLSYTKMSQGCNQPNIHWLNPQRIKMVQPSSYTLNIKHTSAHDSGE

WTCVLTHNGKESELKTTVTVVDLSPAPVDPVYVPLSTTTRLRIPCSFPPELTWEHLRSKG

IVGGHWKFTPNWSPQPGGPQKLFSLSVEDPLAWKEEQKRGLTGADLMKNNLSLTKTSVTE

EDRGTYTCALEFPHVTLSRSVRVEVLQVLNSLESVLVGHQVNLTCSLGHSLTSGLQVKWI

PPRSYVSDLRPVAHHATISIRIESEDQDKWRCELLGNGSQLASAQIKLKIEKPPLNIWLH

VTICGTTAIFILLLFVTVILVRRHRQ

***Typhlichthys subterraneus CD74a***

>utg7180000050069 length=11297 num_frags=1374 Astat=532.00

CACGTACTGATTCTTAGCAGTTGTATAAAGACATAAGCTTGAGCTGCAGATTCAGAAATGCTTGGGAAAATGTAGCTTCTGTGGACGCTACCGCACTACTTGATCAATTAAAGAGCTTTGCTACTGAAAAGCTATTTGATTCAGAAAACAGCGTCGCCACGGCCGCGCTACTACTACTAAACGTAGTTTAACTAGTAGCGTCGCTACAAGTAGCGGCGCTGACATAGGAATATAGCTTCTTCTTCGTTTTAGGTAACCTAGGCTAGGGCCGGTAGTAGTAGCTCCAAACACACCCCTATAACCTTCTTAACATTTACGGCCACGGAAATACGAAAATAAATAAAACAAGGAAATGTGGAAATACGGAAATAAATAAATAAATGTTGAAATTTAGAAATAAATACATAAATAAATACATAAAATAATGTCTAAATAAATACAATTAAGAATAAATAAATGTATGTAAAAATAATTAAATAAATAAATGTAGAAATGCACAAATAAATAAATACATGTAAAAATACAGAAATAGCCTTTTTTAATACAAAAATTTAATTATTTTTTCAATTATGTATATATTTATTTAATTATTTATACATTTATTTATACTTTTATATATTTATTTATTTATTTTTTATTATGGCAGTCCTGGTCCTCCATATATATGAGGTTAATATTTTAATTATTAATTATTTTATTCGAAATGTGTGGATAAGCTGAACAAGACAAACTGACGAAGGAAAGTCCTTCTCTCCAGAGCATTTAGTACGAATACTATGACGTATACTCCATACTGGCACAAAAACAGAATTAAATCATATATCATCTGGTACAGTATCGTTAAATCCTTAGCTTTTTTACTTGTGTATAGGTTACAGTTGACAGTTAACTGTATTTCTATCCCAGTCCCAGGTCTTCCTCAGGGAATACTACAGGGATCACAACATTGAGTTGTCCAAGCTGCTATACAGGATGGGCCAGCCACTACCCAGCTGGCTCCGAGAGGAGTTGGTCCACACCAGGTAGCACTTCCGCACGGCAAGCCACGGCTCCGGAGAGCGAACCACAGCTCACATCCGCTGAGGGGAACGCAGCCCAACGCCATGGAAAGTCCCGGGCGATCCAGGATGTGAAAGCTACCAAAAACATAGATTATAACAGTGGTTCCCGAAATCGCTGAACTCGCGCATGTTGAATACATGTACGTGATCTCCATGAGATGGCTGTAGGAAGGGCATCGCCAGCCATGAGTAACTGGAGAGGGGGGGCTTGCTGAATGTTCCAGAACAAAAGTCACCATGGCAGACTGGAGAGGGAAGGTTTATCCCTATAAATTATAAATCTAGAACCCCCGACCCCACACACACAATCACCCTGCCTTGCGTCTTTCATCAGACCTTCTGTTCATATGTCCACAAAGTTCAAGAACATAAATCAGTCTCCCTTAGTCTTAACAGTGCCAGCCCAGAGGAAAGGGGTTGGCGCACATTTGAAGAGACACTTGGCTCCAGTGATGAGTCCTGGTGTATAATCTGTGTCGTTTGGAGCGCATTGGAGCAGTCGATGCCAAATGTGCTGAGACGGATGCATACTTGATTGACAGACGAGGCATTAGTTGCAAATGCCTTTTTGGTTTCGAAAGGAATTGTGTGGGAAGAGAAGGGATTGACTCGGGCTACATGTTGCAGCCTTTTTCGATAGGCCTTGGCAGTATCTAGCTATTGCATTTAAAAAGTGCCAGGACAGAATCTTCTAAACAATATCACATGAACTTTGGGGGGGAAGGGGGAGGTGCTTCTGTTTTGTTTGTTTTGTTAATATATTTTTTCATAGTTTTCTTTTTCTTCTTTTTTTACTGAGATTGTATTTGTCTTAATGTTTTTATTATGCCAATTGTGGCATGTTAGTAGAATGTACCACAGTTATAAAACAACCGCTCTGATTATTATAATTTCTTTCTGAGGGGCCAGATGTACAGTTGTCTGATTTGTTAAAACTGTTGTTTAAAATTTCCCTTTAGTCCGTTTCATGGCAATGGAAGTGGCTAGTAGGAAAGTTAGGGAAGCATTTTTGAAAGCACAATATTTAAGGCACTCTAAAGTGAACACCATTCTTACACAGGTTATTCGGAAGCACACTCACGAGATAGATCCTGCTACAGCCTGTGAACATAACTGTAAGCTCTGGTTTAAAAGTTTCAAATGGGGTGTGCAAAATAATTTTCCCACCTTTTATCGTACCTGTTGGTTTATCCAGAACTGTGTGTTTTGTCCTCTGTTTATTTACATACTAGAGCAGAGTTGTCTCTTGGGGGAAAATGTGTAAAAGCTGCCAAGCAAACCAATAGTGTGACGAGAAGCTCAGTTGTTGGTCAAGGTATTACCACTGCAACTAAATGGAAGAAGCATACCGGTAGTAACAGAAGCTACGCCTACTGCAATAGTTTTGTACAGTGTACTGACTTAATAACGATGCACATGTCGGTCCTCTTATTTCCTGACAGTAATGTACATAATACATACGCGATGCTTGGTAAGCCTAAATTATTGAATGTGTTTGGGTTTACACACCCTGTATCAGCCCCCTTTTACACATTGGCTACATTTACACACACTTCACTATCTTTCGGAATATGACAATAGGATATGGTTTGGATAAGCAGTATTGTTAAAGTACCTTTTCCTAATAAGGATGTGCTGATAAAATCCTGTTTTGTAGATGTGCAAACAGTGCATTCGTAATGCCTGTTTCAGAACAAGTGCAATAACCAGCCTATTGTGTCTATGTAAATGTAGCCAGTGTCATTTTAGTACCATAATAAGAGGATTAGGAGCGTGTTGTTGTCATTGACATTTCTTTTTTCTGTTATGTTGAAATTATTGATCCCTCTCACATCTTCTCACTGCCAACTATTGTACAGTGGACGGATTTCTTCATATAAAGTATTAAGTGATAAAACGCATAACAGCAACGATGGTACTAATCTCCTGGACTGTATGATGAAGATTTGTAATTGTTGTCAATAATTTTTACAGTGTAACTAATATTGTCGGTGCTTTTTAATTTGCAAATAAAACACCACTGTATTTTTCAGTAATGCTTCTTTTTTCTTAAGTATCCAGTGAAAAGTAGAACACACCTTTCTCCCTTATAACTAAAAAAAAAAATACTTTAATGGCTTCAGTTCTGTGGGCATGTTGAATACATATTCTGTATTGTGTTTGAAGCCTTGAGTTTTACACAAAATCTTATTCAACAGAAAAAAAACTGGTAAAATTAAACAAACCCCCCCATGGAAAAAAAGACCACTGCACTGTCAATTCTCCCATTTTATAGAGGCAGTAACTGAGCAAGAAAGGGTTAAGGGAAAGTACCCTGACCAAAATCTTTTTTTTTTAACCCAATGCCAAAATAGTTAAGTTACCTCCATATGATCTGTAATATTTTATAGGCATGACCAAATTGTAGTTACCAAACCAGATATGTTGTAGAAAGTAATTAAATAAATGTTAGGGGCTACAAATATTTACTAAATGAAGCATTACTACAGATTGATATTATTTTCTAATCTATTAAATGTAGACTATAAATGGCAATAAAATAAGGTAATATGGTGCTCAAACGTTATCTGAACCTAAACTGACATTTAATTTAATTGCTCAGTGTAAAAGTTAATAACTTTTAAATTTTTATTTTTGTCAGTTTAAGTGGCTACTTTAAAAAAAAGAATGTACTTTGTCATTATTTTGAATTATTTTATTTTATAAATAAAGGCAGGTTTCAGCCATTGTATCCGTTGCTTTGTAGTCTGCCTCTATCAACAAGTGATGGGGAAATATGCTAGCCAATAAGAGTGCAGAAAAAAGTCAACAGAACCAGCCCACTCCTTTATAGCCTGGGACTTTCCATTAATTTCACTTCATCTTGTCCTGATACATCCTGCAAAAGTACCTCACGTAGCAGGGCTTTTAAAACTGCTTTTGACACAGCTTCAGTTCTATTGATCAAATCCAGCAGACCCTTATCACAGATGGACCAGCCCAGTGAACATGCTCCCCTGGCCACAGAAAGGTCTCCCAGTTTGGCTGGGAGCGAGGAGGCCCTCATTCTGCCTGAAAGAGCAAGAGGGTGAGACAACATTTTGTAATAAATACATAGGCAAATGTAAAATATCTTTATTCAGTCTCTCGCATGTACCATTCATTATGTTGTTGGTAAGGGTAAGCAACCACCTATTTCACATTACTTTTTTGGAAAAATTTATTACATAAACATTTTTTATTTTTATATTGGGTGTTAAAGTTAGACAAAACTTTAACAAGACAAAGTCATTGATGCAAAAAAAAAAACGTAGTGAGAAGTAATGTGGATACAGTGGCTGAAACCATGCTTTCTTTTGCACAAGCAGAATGTATCTGACTAGAAACAGACGATGCCTCTCATATTATTGGTTCTTATTGAGCCAAGTTATTTTTAATTTAGAAAATATAACTGTATAAGATTAAGGAAAAAATATTGTAAGCACTTTTTAAATATTAAATAAACATTACCCTATACAGGGGGGTTCACCATGATGTTTAGGTATACAATTTAAAAACTAAATGCGTCTTAAAATAACATGTAGTGAGAGGTTTGCACAAGGAAAATGTGCCTGCCTAGCAACTAGTGATGCCCCTCAAAGTATTGGTTCATAAAACAAGATACAATTCTAATTTCGTTTTCTCTGTTGTTTGTAATTTTACTTTAGAACGACTTGTTTAGAAGAACCTACGGTAGTGTTTACTAAAGACTTATGATTTTGATTTACTTTTAATTTGAAAAATCACTGTGGGCAATATCATGAGAGTTTTAAGTTAAATAACTTTCTGCAACAACAACAAAAAAAAGAATACATTACAATTTTACCATTTAGAGTAGAGCATTTTAAATATAAAATAAATTGAACATATAGTGGTTACTATAGTAGACTACTTTTGCTTTTGTTTACACTGGAATATGTCTGCCTAGCAACTAGTGATGTATCTAATATTATTGGCTCAAAAAGTTTTATTCATGTATGATTATTATTACAATATTAGTTGCATTAGTATCATTATTATTGTTATTTTATTTAAACATTTTTTAAGAACTTTACACATTAATAAAATGTATAACTTCATGACCAATTTAAATATTTCAGTTTTAGGGTTTTATTCTTAAATGCAGCTCAACACCCACAAAGTTACAATATGAAAAATGAAATCATAAAAACACAAAATTATTTAAAACTCTGCTACTGCTTCCTTACATTAGGTTACGTCCCACACTATATATTATACACACATTAGCAGGCCTACTACTGTTTGTTATCTCACCGCACATTCCTTTCCTTTGGTGTGAGCAGGGGCTCTAACAGCAAGGCCTTCAAGGTGGCAGGGCTGACCACACTGGCCTGTTTACTGCTAGCAAGCCAGGTCTTCACAGCCTATCTGGTCATCAACCAGAAGGGCCAGATCCAAGACCTCAAAAGGACCTCTGAAAGCATGCAGAAGGATCTGACTCGTAGCAACCATGGTAAGGATTGTTCCACTGGTGTTCCCTGTCATAAAGGCTTACATTAATGTTTGATTTGCAGGAATTCAGAAGGATAAAACATTGAAACAGATATATTTTTTAGAGGGGGCAAAAGGGACTGACTTAACAAGTGAAGTGAAAGCGGTGTAGCTTCTTCAAATTAGTCATGCCAGTATAGGTCAGATAATTAAAGGGTAACTATTACTACTAAGCCATGGTATTCCAGCAGAAGTGCATCCACATTGAGGAAGTACTATTCCTCAACTAAAATGTGTTTTGGGGTTTCTGTTACACCTTAAGTAAAACATAGTTGTACTTAAATAGCTGCAGCAGTGTCCCAAATGCGGATGCAGATGCCCATGAAAAGCCTGCCCCTTCTGACAAACTTCTTTGACGAGGATGAGAAAGCGGGCAAAATACCGCTGACGGTAAGTCCCAACTAATCTCCATGCAGCAACGTTCATTTAAAATATTGTATTAAATATTACATTTAAATTACATTTAAAAGTATGTTTTTATTATGTAGAAACATAATAGCTACTGTATGTCACATTTTCACAAAGAAAAATATGTTAATAATACTTAAAATACTTAATACTTAATTTAAATGTCTCAATCCCAACAGAAGCTGGAGGATACTGCTATTGTCTCTGTGGAGAAGCAGGTGAAGGATCTCCTGGAGGTACGTTTTATATTTACTGAATATATATTCTCATGCAGTAGTGGGAAGAGTGGGACGACAAATGATCCAAAAACCGCTGGTTTGAATCCCTCTAACAAGGACAAGATGCCACCGTGTATGAATGTGTGTGTTAATGTGAGGCATATGTTATATAAATGAAGTCCATTTATCATATTTAATATATATATATATATATACAGTATAAACACTGTCTTGCCAGTCCACACACATACTGTCCCATGTTATCTGTATTAATACAGTAACCACTGTCTTCTGTTTGGTCCAGAGCTTCCAGCTGCCACAGTTCAACGAGACCTTCCTGTCCAACCTGCAGAGCCTGAAGGATCAGGTGGAGGACAGTGAGTGGAAGGTGATGTCTGCCCCCACCCCCCTCCTCCAGAAACACACACACCCATGCATACTGTACCCATACGTGTCTTGTCCAGGGCTATATGTGTATCTCAACCCTCAGGGATTGGAGACCTGGATGCGCTACTGGCTGATCTTCCAGATGGCTCAGGAGAAGCCAGCTCCTCCCACAGCTCAGCCACGCAGTCAGTGGTGGACCACTGCAGGTGAGGATCACCGCAGCAATAGAATACATTTAAAAGGGATATTCCAGGATCCCCCCCCCATCCCAAAGAACCCCAAATGTCCACCTCGATCTCACTGTGTGTGTTTTGCAATGCAACCGCGGAGGTGTAGAGAGCCGTGAGCCCCTCAGTAATGTGTATCGGTGGAGGAAGGGCGGTTGCATGCGGGAGTGAGGGGGAGGGGATGAGCTGAGACATTAACATCAATTGATTTTGATGACACACTGTATAAACGCCCAAGTGTACTGAAACCAGTAAGATCCTTGGGCGATTTGTTTTACGGATATGACATGTAGAGCAATTTTCACACCATAAAAATCCAGTGAATTTTGTCAATATTAACACCTGCATTTATGTGTCTCATCACAGTTCAGTCCTATTATTTCGTTAACAGCTAAATAATAAATACCCAGTTTAGGGTGCAGCTACACTTTAACAGTAAAATAAAACATTTGAATGTGATTCAAATGAAGTACTGTTGGAAACCAGCGAAGTCCTGGGACGATTTTAAACGGTGTGAATTCTTCTGACATATTATATAGTACTATAATAGTATATTATAATAATATTATGCGTTTTGTTTATAAAGTTCATAAAAGAACAAAGACAGAGCTCTGTGTTTGTTGAGAGCAGTTGGAATGGACACATACTGCAATAAACCATTTCACGTTTGGCACTAGGACAGAGCATAAAGTCTACTAAAGCCTGTTTAACCCCACATCGTTTCACTCTCCGTCCCTCAGGCAAAGGCCTCTCCAAGTGCCAGGTGCAGGCTGCCATTGTGCGGCCCGGCTACTATCAGCCCCAGTGTGACGAGCAAGGCCACTACAAGCCCATGCAGTGCTGGCACAGCACTGGCTACTGCTGGTGCGTGGATGTGAACGGTAACACCATCGAGGGGACTCTGATGCGTGGCAGGCCTGTCTGCGCCGGAGGTAGAATACACACGCCCGCACGCACACACAGTAAACACAAAGCAGGGTACAGTTTTGGGGAAGAAGTAAGCCTTCAAAATGTTATGATATAAATATGCTAGGGACGCACCGATCGATTGGCAGGAGATCGATTTCCACTCACAATGCCAGATCAGTGGTAGTCCGATTTTCCTTTCTAATGGCCAATATTTTATATTTGTTGCTGTGGAAATATGTTAGCCAATAAGAGTGAAGAAAAAAAGTCAACAGAACCAACCATCTTGTTAATTTAGTTTATGTGTTTAATTTAATTAATGTGTTAACCTGGATTAAAAATGGACCTCACATTTTTTTATAATAAATAAAAAGTATAAATTCAGTCAAATCTGAAATCGGTATCGGCCACAAAAAGAAAACAGATTAGTGCATTACTATGTAAAACATTTTACATAAATAGAATTATTTAAAAAATGTATCTAAAAGACATACAGTACTGTATATTGCATGTAAGTAAATTATATATAGTATAATTGTAAGAAACTGCTTTAGTGGTCTCTTAATTGTCTCTACGGCTGTATATTAGTTTAAGATTTACAGTGAAAGGACATTGACTAAATGAATGGGTTTAATGGCTAGGCCCTCATATGAAGGCGTTGCCGAGACCCATGCTTCAGAAAGCACTCAAGGTGGACGGTGAGTCAAACACCCTTTACAGCCTAACAGGGACTTTTTTTGGAAATGCTTGGGTGGCTACATTTTGTTCTCATTTCTACTCTTCTTTTTCCAGACAAGTAAGAGAATTAAAGCAGAATGCTGGGCCGGAAAAGCAAGACTTCCTTCACACAGCTCTCGTTTTGTATAGCAATATATATCCATATAACTCTAGTTTATGCATTATCCTCTTTGCAGCTTGCTTTATACAATACAATTATTAAAATGAGATCTGTTACAAAATCACACACAAAAATGTTCTTTATGTATGATATTATTTAGGGTGTTTTCATTTAATATTTACATTTATATATTTACATTTAATTTGTATATTCTACCAGTTGAATAGGCATAGGCATTACGTATTTGGGGTTGAGAAACCTGATGAAGTATACTATTACATTAACAATGATGATTTTTTTTTACTGATTTACAGTACCGTCAAATACTGGCTGAACATGTAACATGCCAAAATAACAAAATAAACTTTGGATTGGATACTGTGAAATAGCTGACATTTTTAAAGGAGACCATCTGCATCTGTTCCTTGAGACGCTACAGAGCTTTTTTGGATTGCGTGACGACAGCATTCACCAATGTGAACTTGGTTATGGCAATATTTCTTTATGGGAAAAGTAATGTGAATGTGCAAGATAGGTTTGTGGTTGGAGTGATCAACTCGCAGCCAAAAGGTTGTGGGTTAGTTCTGCAATATATGGGGTAAGCATCCTTACGCATCATGCCTAACTCATGGTAGCAACAGAAATGTCAAAGCATATACTCTATGAGGAAGATGAGTATAATTATCATATTCTCAGATGGGGGTGAAATGTGATAAGGTGGTTTCAAATATTACTAGAGGAAGTGTATAAATATCCATTTTAATAGGCCGTTGTCAAATTGTGCAAGCAACCGCAGACATCAATCGAAAGTAACGTTGGGTGAGTCTTAAGATGTTTTGTCTACATTTTAGTAGTGGGGGGTAACCTAATATTATTTCATACATTTAAATACATCAATATGTGTTTTACGGAGTGTCTCATAGGAACAGACATCATGATGGTGAACAACAGGTTTCACACTGTTCCTGCTTCTGGCATAGTTGAAGGCATGCATTCAAGGATGAACCCCATTGTATATGCAGCACAGAGGAGTACACCTGTTACAGATACCTAAGTGTATAAAAAAACCTATAGGAAAAACCCTTAACCTGAGTTACTAAATTGGAAAATAAAAACGTTGGATGAGCAATCAAGGCATGTGTGAGTGAGGTGAGTTTTACCGGAGAATTATTGAATGATAGATTTCATATCTTCAGCATGATAACCCCTAGAATCAGATAAGCTAATTTTCGCACTACTTCAAATAAGCAGTCATAGCATGTATTCATGCTCTTTAGATTCCAGGTTATGGTTACACACTATTTGTCTGTCATGGTCTATGGACACCCTGAAATAGCATTGGCCACCCCATAGTTAAAACTCTAGTTCCGCCACTGCCTCTGGCTACTTCGGCCCCTGAAGATGTTTAGCCTGCCGTTTGGCTGGGTTGCTAAGCAACGTGATTCACCTGGATCTGTCAGGGAAGCACAACTCAAGAGACCTCTGTGCTGAACTGTCATCCATTCACATTCCTTTCCAGTTATTAAGAGCTTGGCTTCTCTTGCTCGGAGAGACAACAGTCTGACATCTATCCTGTCACTGTGAACTTCAGAATCACACCCCTGACCTCACTGAAATAGATGTCAGTTGTTGGCTGTCCATTCTGTGCAGCCCAGGAAGTATTGTATCTTCGTGCAAATGCTCTGGAGGTATTCAGTAACCCACCACAGGGATTAGAGGCTCGCCCTGTCCAAGAACCGTCTTATAAGTCTCCCCTCTTTGGCTAAACTCACTATTCTGGAGGTCCTGGAGGTGGATAACAACCAGCTGACCATCTTGTACATTTGTTGTAAGATAGGTCAATTCCAACAGTTACTGGAGAAACCCTTATCGCCTTGACTGTTCCCTGCATGCTTTTCCTGAAGAACACAACACACTGTGTGTGGAGGATTGGCCTGAAGGGTTTGTGTGTCTCACTCCAGAGACCCAAATGAGGAACTAACCTGACAAATGTTCACACAACACTAAGAGGAACGAAAGTCCGGTTTCAACTTGCTTCCCTGTTTGGCTACTATTAGCTTTATCATCCTCTTATTTTATTTTGTCTGATTGATGTTCCTTGCGATGTGTCTCACTGAAGCTTTCTGTCTTTATATTTTAATTGCCCCCCTCCCTTCCCATAATTATTAAGCATTAACATAAAATAGTCATTATGCCCCCATATTGGTATAAAATAAATCTAATTTCAAGAATGATTTTTGATCCTGTTGCCATTTTAATCTTTTTGGTTAAGCATACTGTATCCCCTTGCATAGTGAAAGTTGAGTCTAGTTCATCTTATTAATAATTCAGAACAGTGTAATCAAATCCAGCAATCATGAACACAGTCATGGGGTGGCTGTAAACATGGATACGCTCGTAAGTACCTGCTGTTTACGTAAATTTGCGGTGACTTTGAGATTATACGCTGTTGTTTGTCGTGTAATGGCTGACTGTTTTCTGTGTAGAACTTACCTCTTGAAGATAGTGTTAGAGTTGTGCTCACAAAAATTCCAGGTAAAGACTACAGAATTAAAAAAAAAGTAAATTCAGCAACACTAATGCAAAGGTATTTTGAACTGCGCATTTTCTTAATTGCCTTTAGACGCACAGGATGTGCACTGTGTTGTCGACGAAGACAGGCATATAGAGGATGCAACTAATGAGGTAAAACAAATATGTATTACCTTGTAAGAAGAATGTA

The utg7180000050069 unitig probably encodes a full-length canonical CD74a molecule (we were unable to predict the expected last exon encoding very few residues of the C-terminus and the stop codon):

MDQPSEHAPLATERSPSLAGSEEALILPERARGGSNSKAFKVAGLTTLACLLLASQVFTA

YLVINQKGQIQDLKRTSESMQKDLTRSNHAAAVSQMRMQMPMKSLPLLTNFFDEDEKAGK

IPLTKLEDTAIVSVEKQVKDLLESFQLPQFNETFLSNLQSLKDQVEDSEWKGLETWMRYW

LIFQMAQEKPAPPTAQPRSKGLSKCQVQAAIVRPGYYQPQCDEQGHYKPMQCWHSTGYCW

CVDVNGNTIEGTLMRGRPVCAGGPHMKALPRPMLQKALKVD

***Typhlichthys subterraneus CD74b***

>utg7180000242295 length=4347 num_frags=559 Astat=180.00

(the complementary sequence is shown)

GCTCGGGCCTACAAGGTAGCAGGCATCACCCTGCTGGCCTGCCTGTTGATCGCAGGCCAGGCCCTGACAGCCTACTTCGTCCTCAGCCAGCGGAGCGACATCAAATCTCTGACGGACAAGAACAACGACCTGAAGGACGAAATGACCAGGGGCCGCGCTGGTATGTACAGAACGCCCATGACACTTCACTACATCAGCCTTTATTGTCCCTTCATTATCCTGGGAGATCTGCAGCAGTGGAAGTAACTGATGGGATTGATCAAGTCCTTGAGGTCTGCTTGGCTGATTGACATTTAGGATGCTGTCAAATGCAAGACTGATACATGTGGGTAAAACAATGCTTTGTCAGTTAGGCTGAGAGTTTCAGAAATTCTCAAAACAAGCAGGAAAATGGAAAAACCTGCAGTTGTCTATAAAAGGAAAGAACTACACCTGCATTTCTCTTATTCCATTATGATAATAGTAATGTCAGAGACACTAACGTTCAAAATAGCCAATGGTAACGACGTTCCCTATTGCTTTCCCAACCCGTCTTTCTCTCTTTGGCTCTTTAGCTGCTGTGCCAATGAAAACACACATGGCCTCCCCCTCTCTGATTGATTTTGATGAAGAGGTAAATAACTGTAAATTAAAACGTTAACGTTTTCTAAAAAAGTAATGAAGCTGTTAAACAATACAATAGAACATTTTCAGTCTTGTCACTATTTTATGACGTTGCTGTGTGTGTTGCAGGCTGAGGCAGATACTACTGAAAAGACAGGTATGCATTTATATATATGTGCCTGGTTGAAATAATATTATTATTATTATTCATATTATGTTTTAAATATAACAATTTAAAATGTTTACACAAAGTCTTAAAATTTCAAATACATTTCAAATTTTAAAGGTAAAACAAAACGTCTTTGCCGAAACGATGGATCACGGCTCAATTTGATGAATTTAAACGCACCTCTGAGGTGCGTTCAGGATTGACAGTCAAAATGATCAGATTAAAAACTGTCGCCCTTCATCCATCGCCAGTGTATGTTCACCGAGCTCCTCCTCAGACCATTTGCTTATGTTCGCAAACGTTCCCGGCAAACGTAAAGCAAATGTAAAACTTTGAATATGAATATATACAGTATATATTTATATATGTGAAAATCAACGTTAACATTTTCCCCAAGCTTCGGAGAAGACCAACTGCCAGCTGCAGGCTGCCAAAGGCACGCCAAACTTCCGTCCCGCCTGCGACGGACAAGGCCGGTACAAGAGCCAGCAGTGCTGGATGGACCAGTGCTGGTGTGTGGACACGCTCAGCGGCACAATGATCCCCAACACCATGGCAACAGGTCCGGCACGCTGCCCTGGTGCCAACAGAGCCATGTCCGCCGGTGAGACTTTACTACATTTAATACGTCAGTCACCATCCTAGCCCAGTGGTGCCAACTCTCACGCTTCAGCCATGTGACACGCGCTTTTATGTTCTGTCTCACGCTAGCATGAAAGACCCCATTGACTTCTATGCATCACCTGGGTGCTTTGAAAGGTGACCTTGGGTGTCTATGAATAAAATGTTTTACTGTTGTTATTATTATTATTCTTTGTCAATACTACCGATTCTCACACCAAGTCAGGCTTCAGATTTGGGTCACTGTCCTAGCCTGACACGCCAGATGGTTTGAGACACCGCAGAACCATCTGGAACAGCAGGGGGTGTTGAACTGTGCGATCAATGGCCCACATTCTGGAGGGTTTGATCAATAATTTGATCAAACTTGACTATTTAGAGAAAGAAAAGTCAAAATAAGGGTTAGGGACCAATATTTTAATTTAATGGTGTTGGTTTTCTGTGAGTGGTCATAATGTTTTGGCTCATCGATTTATGAGTATATCTACTTTCTTCTACATAAAACCTTCGGTTTCAATTATAAGTAGTGATGTAATGTATTCCTCTCTCTAGGTGCTCTGCACCCTTTGATGGCTATGGCTGATGTCGTGAGCTCTGGTGGTGAGTAGAGGCACACACCGCCTTCATACTTGTGTGTTCACTACAACATACACTGTACATTGTTGTTTCTTTTGTATCCCACAGATGAGTAATTCATCCATGAAGACCCCATTGAAGCATCTAAGACCAGCTTGATAAATCATAATCAAACCATAATATTTAGACGTGTGGTATTTTACTGGTGTTTGTGCTACTATATCAAGCATCTCCACCTATGCTTCCATCCTGTTATTACAATATTACTCATAGTATACAAATGGGTATTTGAACTGTCACTGTCTCTTATCCAGCTACAGCATATACAAATATTTTCATATTACTGACCATGTCTCTCCTTTGCAGGCAGGAATAGTCATTACTGTATGTATGACAACATAGTTAATTACGTAACGACTTGAAATTGCAGATCAAAACATTGTATCTGATTATTTGCTTTTAATTGGTAATCATCTGAGATACTGAAATGTTTCCTATTACAAAGTGGATTTATTAACTATAGTGAGACAAATATACAGTATATGGTCCTCAAGTCAAGCTAATAGGTATTTTTAAGAAGTAATAATCTACAGTTTTATATTAGATTAAAATACTTACATTAGATCTAGTGATAAGCATTGTTGGCTTTACAGACTGCGTAGAGAGGCACTTTTAATTCAGTGTATTTTAATTGTTATTTAACACAATAAAGACAATGTTGGGGTCATATTCTGTTTAAATACAGTTCTGATTTATTAGATACATTTCGAGATGAATGACTTTGATAATAAACTTTGCTGCACCTGAAGTAATAAAGTTCAACACATCCCGAGATTACATGGTATTGAGATTTGGACATTTGACAGCCAAATCATTAACCAAATTGTTCAACACACATACATGTTAAGTTCGAGAAAAAAAACAGTGACACAAAATGTAACGGCATGCAATCAATTAAATATACGCAATATACTGTATTTAATAAAAAAATATGTGGGCAAGCTCCAGCCGTTTTCACAAGATGATTTTAACAGTAGCCTGGCTAACACCGTCCTGATACTGTAATCTCTACAATTATCTCTCAAATGAAAAATTAAAGTTAACCAGTGACTGGCGTCAATCAACACAGCAGGCTTGGACCCTCCCTAAATCAAATCAAAACATAGCGATGGTGATTTTCAAATAGAGAATAAATCCCACAAGGAAATGTTTTTTCCCATGAGACAAACTTTATTGCTATAAGCTCTAAATGCACCTCATACTCACCTGGACCCTGATTGGTCAGTTGTTCGCTAGCTGACTTGTTGGCAGTTTTCAATTGAGAATTCCTAGACCGATATGTGAAGCAAAGCTAAAAATATAGGCAGCCCTTGCAATATCATGATGGTTTTAACCAGGCAGTAGTTAACAGTACTATATTCGGTCTTTAATTATGAGTTACATTCACCAACTGGGCATACTAGAAGTTTTTACTGTATTTTAATGTCTGAATTAATTTTTTTTTAAACTTCATTTACAAATATGTTTTTCTAAAGACAGCAGCAAGATATATATACTGCAGCATAAATCAAACTGTACCAGATTCAGCCCATTTAGTGCATTGTTTAAAAATGTACCCAGTAAGATGGTTAATGGCCGCATAAAGCTGTCCAATCAGCTGCCTATAGTTTGCAATACCATCGTATGAGCATTACTCCACACACACAAAAACAAAATGATTAATCAACATAATTACAATCTAAAGATAAAAGACATATAAATGATTGTTTTCCCAATGACAAATAAAGCCGTTAGACCAGCAGTTAGACCGGCTTTCACCCTGTAAAATACGGTAACTTTCTTCCAGCATATAAGAAACAATGAAACCAAATCCCTTCAGGAACGGTTATTGCACACATGGCCGAGAGATGACTGCAAGGTCGGTAAGTATAGACAATTGTTCACACAATTCATATGTCTACCATTACAACTTATTAAAAGGAAAAAGTGTCATATGACCAGCACACACTGCATATAAGGCAAGTAATTTTTGATATGACAAAACTGAAAGATGTGAGATTTGGCATTTCAGCGTACCCTTCCCAAATCCCTTAAATGTGCAAAAAAAATAGATAACAATAATACAAATAATTGCAACAGTAACTACAAGATTATAATATTGGTTAACAACGTGTCCTGGCAAGTTTGGACTCCATCCACCTCATTTCCCTCCCCCCCTCAGGTTGTTGAAGTAGTCTTTCAGCGTCTGTTCGATGTTGGGCACGGCGTAGGTCTGTGCTGCGAAGATGCCTGTGCATGTTCCCACGGCGACGCCCATCAATGCTGAGGAGCGTAGCTTGGCAACAACATACCCAGCCAAGAAGCCCTTCAGGAACGGC

The utg7180000242295 sequence only encodes part of CD74b, with the N-terminus missing because the unitig border maps within an exon. The prediction for the encoded part of the molecule is:

ARAYKVAGITLLACLLIAGQALTAYFVLSQRSDIKSLTDKNNDLKDEMTRGRAAAVPMKT

HMASPSLIDFDEEAEADTTEKTASEKTNCQLQAAKGTPNFRPACDGQGRYKSQQCWMDQC

WCVDTLSGTMIPNTMATGPARCPGANRAMSAGALHPLMAMADVVSSGGE

MHKTGQALTAYFVLSQRSDIKSLTDKNNDLKDEMTRGRAAAVPMKTHMASPSLIDFDEEA

EADTTEKTASEKTNCQLQAAKGTPNFRPACDGQGRYKSQQCWMDQCWCVDTLSGTMIPNT

MATGPARCPGANRAMSAGALHPLMAMADVVSSGGE

**3.5 *Percopsis transmontana***

**For the non-gadiform fish *Percopsis transmontana* no intact classical *MHC IIA*, classical *MHC IIB* and *CD4* genes were reported.**

*Percopsis transmontana* is a non-gadiform fish investigated by Malmstrøm *et al.*^1^ which is closely related to the gadiform fishes, and hence important for deciding the phylogenetic stage at which functional MHC class II system genes were lost.

In summary, our findings for their Table S7 listings are:

**MHC IIA:** Table S7 mentions utg7180000591256. This sequence encodes an MHC IIA molecule of the non-classical DB category, which does not have the two asparagines at positions -4 and +3 relative to the second cysteine in the α1 domain which are typical for peptide binding function in classical MHC class II molecules (shaded red in Fig. S1; Zhou *et al.* 2009^3^; Painter *et al.* 2012^4^; Dijkstra et al. 2013^5^). *Percopsis transmontana* unitig utg7180000690903 (not mentioned in Table S7) contains an *MHC IIA* gene of the classical lineage DA, but the information for a full-length canonical gene is incomplete, possibly because that unitig is too short (not shown). We were unable to find *Percopsis transmontana* unitigs or scaffolds reported by Malmstrøm *et al.*^1^ with full-length classical *MHC IIA*.

**MHC IIB:** Table S7 mentions utg7180000656275. This sequence encodes a part of an MHC class IIB molecule. The 3’-part of the coding sequence appears to end within the unusual intron located in the β2 domain coding sequence found in classical *MHC IIB* genes in many neoteleost fishes (e.g. Lim *et al.*^3^). We were unable to find *Percopsis transmontana* unitigs or scaffolds reported by Malmstrøm *et al.*^1^ with full-length classical *MHC IIB*.

**CD4:** Table S7 mentions utg7180000135569. This unitig encodes a full-length canonical CD4-1 molecule.

**CD74a:** Table S7 mentions utg7180000203314. The sequence only encodes the C-terminal part of CD74a, possibly because the unitig is too short. However, scaffold scf7180001425484 published by Malmstrøm *et al.*^1^ has the coding ability for a full-length canonical CD74a molecule.

**CD74b:** Table S7 mentions utg7180000004539. The utg7180000004539 sequence encodes a full-length canonical CD74b molecule.

Below the respective unitig sequences are shown with their relevant protein coding capacity:

***Percopsis transmontana* MHC IIA**

>utg7180000591256 length=7645 num_frags=1809 Astat=526.00

(the complementary sequence is shown)

TTTAGTATTCTGGAATGAGACTGCACTTGTTACATAAGTAGCCTACTGCTGTATTTCAAAGGATGCGGACCCTGAAATCAGACACATCCAGGGTCATGGCTACAGGCCATGCATATCCTTTGTTGGCAAGAGGCTAATGAAGCCTCATAATGCATATGTGATGTGATGCATATGTAAGCTATGTGATGGTGACAAGCTAGAGGTAATTGGCTGCAGCAATGCTAAGCTAACTGCTAATTATTTTTGCTCTGACCCAGAGGCAGGGGCGAGAGACTTGTTGAGCACTGGGCAGTTTGGATATAAATGAATACTGGCTATTTTGATACATCCATGGACACCCCCCTTGTGGCACACGCTGCGGGGGCAGCAGTTTTTGGTCTGGGTCTTCGGCTCCCAGCCATAAGGTCATGGGTTCGAGCCCTGGAGTTGCCCTACTTGCAAGCATCCAAGTTGCCTAACCCCTATCTGCTCCTTGTAAAAATTGTAATTGTATGCATTGTAAGGCACTTTGGATAAAAGCATCTGATAAATGACAAAGTTGTAATGACAAAGTGCTTAGAAACAAACAGGTGCAGCATAAAAAATGTCATTGGAACAAAAGCTTGATTTTTCAATTGACTTTTACTGAACCTTCTGTGGATGTTCGCTATGAATTAACCCCTTGTTCGCGATTGTACCAGGTCCCGCCACTCACAGTTGTATTAATCTGTAAATATATATGAAATGCTACAGCAACTAAATTCTTCCAACTCAAGCAATTTGAGTATACATACACCATAGTGATGACAATCAACTTTGTGTGTTTTGTGTTTTTTAGAAACAAATGTTAGTCAATGGCACATGGTTAAATCCTGCTTTATCCTGCTTGAAGAGATTTAGGTGTGCAGTCAGGGTGTTACCACATCATTATGCAGAATGCTTACTGTGATTATGATGTTGGACACTTCTTTAATATATAGATTTAAAATAATAGATTTAAATCTACAAAAATGAATCCAATCAACTTTTGCTAGCCCAGTTGGTACAATTAAAAGGCTGTGTGAAAGGTTGAGTTCACCGGGCTCTACTTAAGAATGAGGAAAGATACTACAACAAAAAATTGCATGGCACATGCATCATAAACAAGCTATAATTTCCTGATTGACTGCATGGCAACCAGCAGTGGCTGGTTTCACAACAGATTGTATCTTGACTTTACCAATCTCATTATAAGATATGAAAGAAGAAAATACGTACATTTTGCTATGTTTTTTCTCTTTTAAAAGTATGCTTATGTGAGTGTGTATCTTTTACTGTTGCCAATTCTCTTCTTTTCACTATCTTCTCAGTTTTTCATTATTTTCCCCTTAATTGACATGATTCCATATAATTTCCAGAGAAGTGTTTTCAACACACACACACACAAAGAACATTTTCTACAACAGATTCAGGTCCGTATGTATTCAGCTTTAATTTTATCAATAGGTACAGTAATAACCATAACCCCATTTCCTTTGGTGCTGCTGGTGTTTTTTTCATTCATAGTCCATAGTTCTATAATGCACCAATGGACTATAAATGTGAACATTTCTTAAACCCTCTATTAGACATACCATATGCACGTATATTATTAAATATAGATCATTCAAAACAAGTCTGTTTTGAATTATCTATATTTAATAATATAGCTGAAATCTTTTTGCTTAAAACATTATTGGTCTAATTCAATAATAGTCAATACCAGATGTTAATTAGACTGAAGCAGCTCAACTGTCTTTCCACATTGTTCGTTTCCTCCTGCCTGTCCCCTACCTCCTATTGCTCGTCTCCTCTGTGTGAAGTGAGCCAAGGGAATGACTGAGGTTTGGGCCGCAGGGTCAGTCCTTAAAGATGATGATGCTGCAGCTCAGCTGACATTTTTGCTACACTGCTTGTTTACTCCTGTCTTGCTCTTCACGTACCTCCTGCCCTTTCTACCTGTCATTTAAAGTGTCTAGTGAGCGAGGGGAAGGATATAGGAAATAATGGTAAATACTTGGGACACTAAAATACTAAAGAAAATCAATGTTTATTTTAAAGTTGAAAATACATGAGTAACACCATCAAGCCACACCAACTTAACTACTTACTTGTATCAGTATATGTAATTATTGACTTAATAACAGCTAAATTATTACTTTATTTTAATAGATTCTACAGATGAGATACTGCAGGGGAAAACACCAATGCATTGCATTGTGGTCTCTTTACATAGTGAGTGTTTATTCTGATGAAGTACAACTCCATTTCTGTGTGTGAACCACTATAAGCTGAGTACAGCGTGAGCTGAATACCTTGTTTAATGTATTTCAATAATGTAATATACTGTACTTGTTTCTGTCAGATCTGATATACTATCTAAACATCAACCTGTAAGCAGTAGAGTGGCCTAATGGTAGATATCCCTCTATGCTTGTGTGTAGCTTGTACAACTTTAACCCCAAACACATCAGAGTGACATGGTTAAGAAATGGACAGGAGATGTATGCATGGCCATGTGATCAAGTCAGAAACACTGTATCTTATTACAGAATTTAAGATAGTTTAAATTGCCCTGTCTTTATTGTATGCCATAATTTAATTTTTGGGGAACAGTCTGAAAAGCAAGAACAAAAAAAACAGAACAACAGCCCTTTATTGAAGTATCTAAATTAAACAAACAAAAAACAAGGAACCTCACCTCATGCTACAGCCTGACTGACACATACAGTTCATAAGTGGAATGCTACACAACATTTCCTGGAGAAGAAAACTGACCTGGTGGATTATGAGGTCTGAAGGTATGAGTAAGAAATCAAACAAACATTTGGTACTTATTTTTCTGGATAGAGGTAGGAGTGTCTCAATGTACTGTACATCATAGAGGTAGGAGTGTCTCAATGTACATCATAGTCTTGGCCTGTTATTATAAAATACTTGGCTCTGCATGCATGTGTGCATTTGTAATGTCAGTGTGAGTTGTTGAATTTGTTTTGAAGCAACAATCTTTGCATTGCAGTTTGAAGGCATCCATAGTGGTCAAATGCAGTGTGGACCACTGGAGGGATGTACAGCACTTTGGTCAATAGAAGTTGTCTTTTAAACATGCTTAATGAATACATATAACTTCTGGCTCGACAAACAACTTAGTGGTGTTATATTTAGAAAACGATTTGCATAAGTAATCCCTTCTCTCCATAATGGAGTTCATTCAATTCAAAATACCTAAGCAGAGAATCACTGGCTATGTGATTTAGTTCTTCTTCTGATTGGTTGAAGGTCCAGTCGATTGTTGTAATAATATGTCAAGGAATTAGAGGATGTCTATTTAACTTATACACAATGAAAAGTACATAGTGGAAACGGTATCTTATCATCTTAGTCTGGCTGAACTCCCAGGATACTGGGCAGGGGAGTGTGACAAGGACTGACTACATGTTGTTCCTATTCAGGGAATGTTGAGTAATGTAATGTAACCTATAAAATGATACATATGTACATTAATAATCAAACACGCTGACTTTCATTAGGTTACTTTGCTACCTGACCAGGCTGAGTAACTTCGATAGGCAAATTTAACGCAGTCAAAATGTTTTGTTGTACAGAATTAATTGGGTTTTTCGCTAAGCCTCCACACATATTAACCAACAGCAACAAGTTTCTCTGCATTGGACTGCATTGTAATGAAATCACTGTTTTTGATGTAGTATTTACCTCTTTACCATACTATATAATTGTACACTATTTCTTCAATGGAATAATGTGCACCCTCTCGACACACCTCTTTCCCTTGAAGTTTGCGATGGCAAAACAGCGTTGCATGCATGCATAATTTGTACTTACTTCACTCTCAAAGTCACATCAGAGTACAGTAGTGTTAGCTAGTAGGCCTATACCAGGCTTTGACTACTGACGAAGATATTATGGATAAAATTCTTCTTTTCATTGGAGCAGTTGCAACCTGCACACAATGTAAGTGGGTTCTTCTTAAAACTATTTGCGAATTATTTGTAAACTTTCATGTGTCCAACAAGTTCCTACAACTGTTAAAATGTTTTGAACCTAATTGCTAAATTAATGAATAAATTAAAGTTATGTATTTTTTTTCTTAACAGCAAATGTGGAACTTTGTAACTACGTTGGATGCTATGAATCAGGCGATGCTGAAGTCTATATGACAATGAATGATGATGAATTATATCATTTCAATTTTAAGCAAAAAGATGTTGTGTGGAATCCTAGGGTCCCAACGTATATTCGCACTGCTTTGGCCTATGAAATTGCACTGTTTACCAGAAACAATTGTCAACATGACATTAATAGATACAAACTGGAAAATAACACTACGCCAAAGTCTAGAGGTAAGGAAAATCAATTCTTATATAGAATAGTTTCCAATGCTTATTCTCTGTTGACTGTGTTATTGCTGTGGTGATTTAATCCTCAGAAGCACCTGAAACTGTCATCTACCCCAGGGATAAGGTGATTATGGGAGTAGAGAATACTCTCATCTGCTTTATAAACCATTTCTACCCTCCAACTATCAAAGTCAACTGGACCAAGAATGGTATAGAGCTCCCTGCGGACAGATCTTCAATTCAACGCTTACCTAATCATGATGGAACATTCCACATGTTCTCCAGGCTTCAGTTCACTCCCCAACATGAAGACATCTATGCCTGTACTGTAGAACATGAGTCTTTGGAGTACCCTCAGTCACGGTTTTGGGGTAAGAACTACAAAAAGAAAGAAACAAATCCACCCCTGTATCTGTATAGCTGAAATGAGAAACAAGATTGACGCATTGAGTTGCTATTTGAATGTATTGTTATTCACAATCTGACAATAAGTTATTAGTTACAATCTAAATTCAGTTATTTGATTGCTACAAACGTTGTTATATCTTGCAAAACTAATGTTAAGACACTTTATTTTTATCCAATGGAAAATTCAGCTGTTAGTAGCATAAAGGGAAGAGTGCAAGATCGTTTGTAAAAATGATTGTCCAGCAACCTTTTTTTGTGGAGAGGTGATGGGATGCACATGATGGTGATGGAATAGCCTGACTAGGTAATATGCCCTACACACCAACTATTAGAAGCAGTAAGTGACCTAACTTGTGTTAATATCCCACAAGTACACTACATACAGTACATACGCATGGAACATAAACACCAATTGGACATAATGGGTGCTGCACCTTAACACAAGCCAGGCAGGCCTACATGAACACAGGTGCAGCATTAGAAAAAGTCCACCGCCCACGTTAGCTGTGTGCTAACTTGTCCTACTAGCAACATACTGTAGGATACTCGGTGACGGAAGTATATCACTCACAAAAAAACAAGGAAGAAGAACAATACCTAGTTCTCCTTTTTTCTGCGGTGTAGTCTAAGTTAAAAGACAGTTCTACAATCCCCATGGTTATCTGATTTTAGAGGGGGAGGTTGTTTGGTGCAGAGACGTTGGGGTAATCCTCCTAGTCTAGTCCTCTCTCATTTTCAGTAATAATTATAGCAGTAGTAATTGCTTAACTATTTAAAAAAATAACCCAACAATGGCCCAAGTGCTTGAGAAATACAGTAACCATCAAAAAACTATTAAATGTGTACTCGTGGCTCACGACTCATAACTTTCTCTGCTACTTCTTGCCAACCATTGCCCCAAATATTGTAACAATGAAGCATTACAATTTGGCTGAAAGCTTGTAAATAATATGGATATTTTTCTTTTTTTCATCCATAGAGTCTGAAATAAAAGGGTCCATATCAGGTCCAGATGTCTTCTGTGGAGTTGGTCTGAGTCTGGGACTTGTAGGTGTTGCTGTTGGAATATTCTTTTTTGTCAAAGTGAACAATGGCTCATGAAAAATAACAGAACAGGGTATATTTGTGATCTCCTCCCTGTATTGTACAGAAGGCATTTTGTGTTCATGATGCATTGTTAAAATCAAAAGTAAAAACAAAAGTTTTCAGTTTATGACAATACTACCATTCTCTAAATATGTCACATATACAAATATATATAATTTTAGAAAATGTGCTGTAATTGTTAATGAATAATAAAGAAATAAAGATGTTTATGTCAGAACTTTGCTTAACTGTGCTTGTGTTACAATGGCAGGATAGTCTAGTGGTCTAGGTGTTGGACTCTCAGCCGTAAGGTTGTGGGTTTGAGCCCCAGAGTTGCCCTACCTGTAAGCAAATCTAATTTGTTGAGATGAGCTCATAGAGAAAAGTAGGGTTGTGTAGCAGCCATTATTGTTAATGGCCTGGAAGAGGACTGTGTGTAAGATCTACTATCATTATGTCATTTCATAAACCTTCCATTTGGGCGAAGGGTATCGCTATGCAGAGTAATGTCGTTTACACAGTATACGTGGAGCGTTTTTTCTCACATTATTGGAGCCTTTTCTCCAATTTGGTTTGCACACTGCCTGGGAATTGCCAGTGGATTTTATCCGCCCCGCACCCCAACATTCTCTATGTAATTTGCTCATGATCTTAGTTGCACTGCATTGATCTTGTAGTATTAACATAATATTACTGTAAGCATGTTTTAGAACAGTTATGTAGAATCACAAATATATTAACATATTATGATAACAATTCAGGCGAGCAACAAAGTTATACAAAGTTTGACCAAACAGTCTGACCAAATGTTGTGGCAAGGGGGGTAAAAACCAAGCACAATTATTTCTTTTAAATTATTCATCATCATCATCTTCAACCACTTACCTGGGTTCAGGTCGTGGGGGCAGCAGCTCCAGCAGGGGAGCCACATTAACCATCTCTGACTGGGGGATCCCGAGGCATTCCCAGGCCAGTGTGGAGATCTAATCTCTCCACCTAGTCCTTGGTCTTCCCCTGGGCCTCCTCCCAGCTGGACGTGCCTGGAACACCTCCCTAGGGAGGCGCCCAGGGGGTATCCTAACCAGATGCACGAACTACCTCAACAGGCTCCTTTCGATGCAAAGAAGCAGTGGCTATACTCCGAGTTTCTCACGAATGGCTGAGCTTCTCACCTTATCTCTAAGGGAGATGCCAGCCACCCTACTGAGGAAACCCATTTAGGCCGCTTGTACCCACGATCTAGTTCATTCGGTCATGACCCAGCCGTCATGACCATAGGTGAGGATAGGAACAAAAATTTACCAGTAGATCACAATACCGCCCCCACCGCTCCGATTCTCCAGCCAAATTCTTCCCTCACTTGCAAACAAGACCCCGAGGTACTTTAACTCCTTCACTTGGGGTAGGGACTCATTCCCTACCCGGAGTAGGCACTCCATCGGTTTCCTGCTGAGACCCATGGCCTCAGATTTAAAGGTGCTAATCCTCATGCCAGCCGCTTCACACTCGGCTGCGACCCGATCCAGTGAGTGCGGAAGGTCACTGACCGATGATACCATCAGGACCACATCATCTGCAAAAAGCAGCTATGAGATCAATAGCCCACCGAACCGCAAACCCTCCCCACCACGACTACGCCTCAATATCCTGTCCATGAATATCTCAAACAGGACTGGTGACAAAACGCAGCGACTGCCTTGTGTGAGCGAGCAAACACTTATTGATTAACTTGGGAGCGGATTGAAAGCGGGTCCGCTCCACGTGTGGCGTGCAACAGGCTTAAGCCTGTGCCTATT

The utg7180000591256 sequence encodes an MHC IIA molecule of the non-classical DB category, which does not have the two asparagines at positions

-4 and +3 relative to the second cysteine in the α1 domain which are typical for peptide binding function in classical MHC class II. We were unable to find a trustworthy prediction for the leader peptide exon. The detected part of the encoded MHC IIA molecule is:

NVELCNYVGCYESGDAEVYMTMNDDELYHFNFKQKDVVWNPRVPTYIRTALAYEIALFTR

NNCQHDINRYKLENNTTPKSREAPETVIYPRDKVIMGVENTLICFINHFYPPTIKVNWTK

NGIELPADRSSIQRLPNHDGTFHMFSRLQFTPQHEDIYACTVEHESLEYPQSRFWESEIK

GSISGPDVFCGVGLSLGLVGVAVGIFFFVKVNNGS

Below shows how this sequence is encoded by relevant parts of the unitig.

4030 4040 4050 4060 4070 4080

TGTCCAACAAGTTCCTACAACTGTTAAAATGTTTTGAACCTAATTGCTAAATTAATGAAT

4090 4100 4110 4120 4130 4140

AAATTAAAGTTATGTATTTTTTTTCTTAACAGCAAATGTGGAACTTTGTAACTACGTTGG

N V E L C N Y V G

4150 4160 4170 4180 4190 4200

ATGCTATGAATCAGGCGATGCTGAAGTCTATATGACAATGAATGATGATGAATTATATCA

C Y E S G D A E V Y M T M N D D E L Y H

4210 4220 4230 4240 4250 4260

TTTCAATTTTAAGCAAAAAGATGTTGTGTGGAATCCTAGGGTCCCAACGTATATTCGCAC

F N F K Q K D V V W N P R V P T Y I R T

4270 4280 4290 4300 4310 4320

TGCTTTGGCCTATGAAATTGCACTGTTTACCAGAAACAATTGTCAACATGACATTAATAG

A L A Y E I A L F T R N N C Q H D I N R

4330 4340 4350 4360 4370 4380

ATACAAACTGGAAAATAACACTACGCCAAAGTCTAGAGGTAAGGAAAATCAATTCTTATA

Y K L E N N T T P K S R

4390 4400 4410 4420 4430 4440

TAGAATAGTTTCCAATGCTTATTCTCTGTTGACTGTGTTATTGCTGTGGTGATTTAATCC

4450 4460 4470 4480 4490 4500

TCAGAAGCACCTGAAACTGTCATCTACCCCAGGGATAAGGTGATTATGGGAGTAGAGAAT

E A P E T V I Y P R D K V I M G V E N

4510 4520 4530 4540 4550 4560

ACTCTCATCTGCTTTATAAACCATTTCTACCCTCCAACTATCAAAGTCAACTGGACCAAG

T L I C F I N H F Y P P T I K V N W T K

4570 4580 4590 4600 4610 4620

AATGGTATAGAGCTCCCTGCGGACAGATCTTCAATTCAACGCTTACCTAATCATGATGGA

N G I E L P A D R S S I Q R L P N H D G

4630 4640 4650 4660 4670 4680

ACATTCCACATGTTCTCCAGGCTTCAGTTCACTCCCCAACATGAAGACATCTATGCCTGT

T F H M F S R L Q F T P Q H E D I Y A C

4690 4700 4710 4720 4730 4740

ACTGTAGAACATGAGTCTTTGGAGTACCCTCAGTCACGGTTTTGGGGTAAGAACTACAAA

T V E H E S L E Y P Q S R F W

4750 4760 4770 4780 4790 4800

AAGAAAGAAACAAATCCACCCCTGTATCTGTATAGCTGAAATGAGAAACAAGATTGACGC

4810 4820 4830 4840 4850 4860

ATTGAGTTGCTATTTGAATGTATTGTTATTCACAATCTGACAATAAGTTATTAGTTACAA

(stretch not shown)

5650 5660 5670 5680 5690 5700

ACTTCTTGCCAACCATTGCCCCAAATATTGTAACAATGAAGCATTACAATTTGGCTGAAA

5710 5720 5730 5740 5750 5760

GCTTGTAAATAATATGGATATTTTTCTTTTTTTCATCCATAGAGTCTGAAATAAAAGGGT

E S E I K G S

5770 5780 5790 5800 5810 5820

CCATATCAGGTCCAGATGTCTTCTGTGGAGTTGGTCTGAGTCTGGGACTTGTAGGTGTTG

I S G P D V F C G V G L S L G L V G V A

5830 5840 5850 5860 5870 5880

CTGTTGGAATATTCTTTTTTGTCAAAGTGAACAATGGCTCATGAAAAATAACAGAACAGG

V G I F F F V K V N N G S *

5890 5900 5910 5920 5930 5940

GTATATTTGTGATCTCCTCCCTGTATTGTACAGAAGGCATTTTGTGTTCATGATGCATTG

***Percopsis transmontana MHC IIB***

>utg7180000656275 length=1442 num_frags=275 Astat=107.00

GACCTGTCTGTCTCTACCTCCTGAAGAGACCTGTCTACCTGACCTGTTTGTCTCTACGTCCTGAAGAGAGACCTGTCTACCTGACCTGTCTGTCTCTACCTCCTGAAGAGAGACATGTCTTCAGTCCAGCTGTGTCTGTGTGTCTTGTTGCTAGGCAGCTTGTATGGAGGAGGTAACTATGATTAATATTAATGTGATTATTAATATAGTGATAACTAGTATCCCTAGAGACACTACCATAGTGTAGTAGTAGTACATACATAGTTTTATAGTAGTATTCTAGTGTGTACTCTACTGTACAGATAAAAAACATGTTGTTGTTTGTTTTTCCTCTCAGATGGATTTTTGGAATACATGGTGTCTGAGTGTGTGTTCAACTCCACTGAGCTGCCTGACATAGAGTACATTCTATCTTATTACTACAACAAGATGTTGGATGTCAGGTTTACCAGCACTCTGGGGAAGTATGTTGGATACACTGAGTTTGGTGTGAAACAAGCAGATGCATGGAATAAGGATTCTTCAGTGATAGGCAGGAGCAAAGCTCAGCTGGAGGCTTACTGTCAACACAACATTCAGATCTGGTACAGTACCAAGCTGGATAAAACAGGTGAGACTCACTTTAACTTGTACCTTTATAATCACTGACTCATTAACATCACAAGAGATCAAGGATTCTTCTTAATGATATTAACATCATTATATGAATCATGTTTATTATTCTGCTGGTATTAATATTAGTCTGTTGTTAATACTGTTACACACACACACGCGCTTCTTTTTGGAATAGCGCCCTGGCTCCACCTAGTGGCACACTGCGGTACTGCTACCACTGATTAACCAAATAACATTATACCGTGTTGTTCTAGCTAAGCCCTATGTGAGGCTGAGCTCAGTGACGCCCCCTGGTGGCAGACACACGGCCATGCTGATGTGCAGTGTTTATGACTTCTACCCTAAACACATCCGTGTCACCTGGCTGAGAGACGGACTTCCTGTCACCTCTGATGTCATTTCCTCTGAGGAGATGGCTGATGGTGATTGGTACTACCAGATCCACTCCCACCTGGAGTACACACCCAGGTAGTACTAATACTACTGTAGTACACACCCAGGTAGTACTAGTACTACTGTAGTACACACCCAGGTAGTACTAATACTACAATACTACAGTAGTACACACCCAGGTAATACTAATACTAATACTACTGTAGTACACACCCAGGTAGTACTAATACTACAATACTACTGTAAAACACACCCAGGTAATACTAGTACTAATACTACTGCAGTACACACCCAGGTAGTACTAGTACTAATACTACTGTAGTACACACCCAGGTAGTACTAGTACTAATACTACTGTACTACACACCCAGGTAATACTAGTACTAATGCTACTGTAGTACACAACACAGTAGTACTAATACTACTGTGGTACA

The utg7180000656275 sequence only encodes the N-terminal part of an MHC class IIB molecule. The encoded sequence, shown together with the coding sequence, is:

10 20 30 40 50 60

GACCTGTCTGTCTCTACCTCCTGAAGAGACCTGTCTACCTGACCTGTTTGTCTCTACGTC

70 80 90 100 110 120

CTGAAGAGAGACCTGTCTACCTGACCTGTCTGTCTCTACCTCCTGAAGAGAGACATGTCT

M S

130 140 150 160 170 180

TCAGTCCAGCTGTGTCTGTGTGTCTTGTTGCTAGGCAGCTTGTATGGAGGAGGTAACTAT

S V Q L C L C V L L L G S L Y G G

190 200 210 220 230 240

GATTAATATTAATGTGATTATTAATATAGTGATAACTAGTATCCCTAGAGACACTACCAT

250 260 270 280 290 300

AGTGTAGTAGTAGTACATACATAGTTTTATAGTAGTATTCTAGTGTGTACTCTACTGTAC

310 320 330 340 350 360

AGATAAAAAACATGTTGTTGTTTGTTTTTCCTCTCAGATGGATTTTTGGAATACATGGTG

D G F L E Y M V

370 380 390 400 410 420

TCTGAGTGTGTGTTCAACTCCACTGAGCTGCCTGACATAGAGTACATTCTATCTTATTAC

S E C V F N S T E L P D I E Y I L S Y Y

430 440 450 460 470 480

TACAACAAGATGTTGGATGTCAGGTTTACCAGCACTCTGGGGAAGTATGTTGGATACACT

Y N K M L D V R F T S T L G K Y V G Y T

490 500 510 520 530 540

GAGTTTGGTGTGAAACAAGCAGATGCATGGAATAAGGATTCTTCAGTGATAGGCAGGAGC

E F G V K Q A D A W N K D S S V I G R S

550 560 570 580 590 600

AAAGCTCAGCTGGAGGCTTACTGTCAACACAACATTCAGATCTGGTACAGTACCAAGCTG

K A Q L E A Y C Q H N I Q I W Y S T K L

610 620 630 640 650 660

GATAAAACAGGTGAGACTCACTTTAACTTGTACCTTTATAATCACTGACTCATTAACATC

D K T

670 680 690 700 710 720

ACAAGAGATCAAGGATTCTTCTTAATGATATTAACATCATTATATGAATCATGTTTATTA

730 740 750 760 770 780

TTCTGCTGGTATTAATATTAGTCTGTTGTTAATACTGTTACACACACACACGCGCTTCTT

790 800 810 820 830 840

TTTGGAATAGCGCCCTGGCTCCACCTAGTGGCACACTGCGGTACTGCTACCACTGATTAA

850 860 870 880 890 900

CCAAATAACATTATACCGTGTTGTTCTAGCTAAGCCCTATGTGAGGCTGAGCTCAGTGAC

A K P Y V R L S S V T

910 920 930 940 950 960

GCCCCCTGGTGGCAGACACACGGCCATGCTGATGTGCAGTGTTTATGACTTCTACCCTAA

P P G G R H T A M L M C S V Y D F Y P K

970 980 990 1000 1010 1020

ACACATCCGTGTCACCTGGCTGAGAGACGGACTTCCTGTCACCTCTGATGTCATTTCCTC

H I R V T W L R D G L P V T S D V I S S

1030 1040 1050 1060 1070 1080

TGAGGAGATGGCTGATGGTGATTGGTACTACCAGATCCACTCCCACCTGGAGTACACACC

E E M A D G D W Y Y Q I H S H L E Y T P

1090 1100 1110 1120 1130 1140

CAGGTAGTACTAATACTACTGTAGTACACACCCAGGTAGTACTAGTACTACTGTAGTACA

R

1150 1160 1170 1180 1190 1200

CACCCAGGTAGTACTAATACTACAATACTACAGTAGTACACACCCAGGTAATACTAATAC

1210 1220 1230 1240 1250 1260

TAATACTACTGTAGTACACACCCAGGTAGTACTAATACTACAATACTACTGTAAAACACA

1270 1280 1290 1300 1310 1320

CCCAGGTAATACTAGTACTAATACTACTGCAGTACACACCCAGGTAGTACTAGTACTAAT

1330 1340 1350 1360 1370 1380

ACTACTGTAGTACACACCCAGGTAGTACTAGTACTAATACTACTGTACTACACACCCAGG

1390 1400 1410 1420 1430 1440

TAATACTAGTACTAATGCTACTGTAGTACACAACACAGTAGTACTAATACTACTGTGGTA

CA

***Percopsis transmontana CD4*** *(CD4-1)*

>utg7180000135569 length=4852 num_frags=933 Astat=484.00

GTGTCAACACCACCACCAACACAACCTGCTACATAGGCACATGCTGTAGTATTATAGGAGTTGCTAGTTGCATATAGTTTCAATGTATTGAAACTTCATACTACTAATTATAATAATAATTACCTAATATACTAGATATTAGGTAATTATTATTATAATTAGTAGTATGAAGTTTCATCCTTTCCCTGCACATTACTCAGTAATTTTCAGGAACGATATGTAATTGACGGGCTGTAATTTAAAGCGTTTGGTAATACTTTAAATGCATGTGACCAAACTGGGCACAAAGGAAAAAAAAATAATTATTTTGACATACCTATAATATATGCAAACTGTTTTTAATACTTTTTTTTTATTTTTCAGGCGTGGGACCAAAATGAAGAATTGTATCCAGTCTCTCATTGTCTTTAGTGCCTTTATATCAACAGGTAGGGTAAATTGTAGCATGTGATTAGCACACTCTGTTAAGGACAGGTATTTAAACATCAGAAATGTCTAATTTTCTTTCCGTTCAGCAGCTGTGCAGGAGAAGTACTGTATGTACGCACAGGTCGGTGAAACGGTCACCTTCACTTATACCCCACAACAAACCGAAAGCCAATCATATTATATGTACTGGTACAGCAGGGCTGGAGGAGACGCACCTCTTATTTCACGGAATCCTTTTGGCCATATTTCTAAGCCAGGTGTGTGGAACCTTCCATTGTTCTGGCTAGTGCTTTTACATTTGTTAATCAAACTAATTAATTACAATCTGTGTAATAACGACGATGGCATGACTTATTTCAGCTAAGCAATGGGAGGACCGGTTGTCATCCACTGGCTACTCTCTGACCATCAAAAACGTCAAAGAAGAAGACTTTCAAACCTTCACCTTGAATCTGAAGATAAACAGGGAGACTCAAATCACCGCTACTTATGAACTAATCATGGTCACCAGTAAGTGTTAACATGTGCAGTCATCAACATCATCTAATGCAGTGGTTGTCAATAATATTAATAATATTATTTTTATGCCATGTCCCTTTAAAAAAGTTTGTCATAGCTTTAAACCCACCATATTGGAATAGCATAACAATAATTGCGATAAACAACACTGAAAAACATTATTAGTATTTTATTCAAAAATCATTTTAAATTAAGTTTATTACAAAATTTGGATTTCTTACGACCCCCAAAAACTGCCCCAGGCCCCACTTTGGGAACCACTATGTTATGTAACCAAAGCCCTGCAAATTCTGCAGGGACCAAAATTGACAGTATGTTGAAACAAGTTAGAGGCTACATTTTTTCCCCCAAATGTATTAAAAATCACAGTTAAAAAAAAAAAACGCTACATGAAAATGTGATGATACAATTACAATTAATGTTTTATTAAGGTACTTTAATTCCTTTTTTATTCTGCTTTTACTCTCGTTCCATGTTTTGACAGTGGGTGTACGACCAGACTATATTCTGCTGGCTGGTGAATCCTTAACACTGTCCTGCACACAGATGTCCCATAGTTGTGCCCAACCACAGATTCACTGGCTCAGTCCCCAGAAGATGTCAATGGGCCAACCCGGTCAGCCCACTTTAATTGTTAGGAATGTCACGGGTCAACACACTGGAGTGTGGACCTGTGTTTTGTCACACAATGGCAAGCAATCCTTTGTCACATCTTCTGTTAAAGTTGTGGGTAAGTAAAAAAGTATGTATTTTATTTATTATTATTATTATTATTATTATTATTATTATTATTATTATTATTATTATTTTTATTATTATTATTACTATTATTGTTGTTATTATTATTATGTGTAATGTAAACAATGTGTGTGTGAGTGGATGTGTGTACACATACACATGCACACAGGCTACCTTCATATTACTGTGGCTTCGTGAAATAGTTGTTCTCACTATCAACTAAAGGTTATTTGGTTAATTCTCTCTATCCCTATCCAGACCTCTCCCCATCCCCGCCACATCCTGTCTATACCTCTCTGTCCACCTCCCCCCGTCTCCACATCCCCTGTTCCTTCCCTCTTCTCTTCACCTGGGAAGACCTCAGGACCAAGGACATAGTGGGAGGTCACTGGAGCTTCACCCCCGACCAGCCCCCTCTCCCTGGGGGCCCGCAGAAGCTCTTCTCCCTGTCCACGGGGGATCCACCGGCCTGGCAGAGTGATCAGGAGCGAGGGCTGACCGTAAATGACCTTGGGAAAAATGACCTGTCTCTGACGAAGAAGTCTGTGACGGAGGGAGACAGGGGAACGTATACCTGCTCCCTGGAGTTTAGAAATAACGTCATGAGCCGGAGCACACGCGTTGAGGTGTTACAAGGTAAGTCAAACTAAAAGCTAATGCAGCCCCCAACTGTCAGAGGAACAACACACAAGATGGATCGCTTGTGATTTCTTTGTTCTGTTTGTTCTCGGAGGCTACCACAGCCTCTTCATCTATCTTTGCTTTATCTCTGTCTGCCTTGTTTTGTTCCTTATTGCAGTTGTTCTGAAACAGTCAGGATTCCCTGGTGGGTCACAAGACACTTTCAGGGGGGGTCGTGAAAAATGTTTTGAAATATTTTATTTTACAACAATATTTTTTTATGTTTTTAAATTATTATTGTTTTCTAAATAAAGACATTTTACCTGATGTTGAAAGATGTGATACATTTTTGGCGAGCAAGCAATGAATCACTTGTGTTTGTGGGTCACAGGTCAAAAAGTTTGGCAACCACTGCCTTATATCGATATCGATAACAAGTTTCTCTTCTGTCCTGTCATGCCTGGCCAGTGTTCCAATTGATCTGTTATGAGGTTTGTTTGGAACAGTGTCCCTGGTGCGACATATGTCTAATTCACTGGCTTTCACAGTTGCCTGCCTCCACTTGTTTTAATAGATAAAGTGTAATGAGCATTCACTCTGCCAAAAGAGTTGGGAGAAACAATACATTTTAATTGGATTCTTTGAGTTCTGACTATTTATAGAGGAAGATTTTTTTTCATTCATTTTTTTCCCCTTGTTAATGTATTTAACTATGTTGATTAGCAACACATTTATAATTGCAACAAAACTGTAGGCAATTTGAGGGAATGGGGTTACAAGTAGGGTTCAGTCGCTGTTTTGCTAATTTGAACATTTTTCTTTCTTTCTTTTGTTCATTGTTTGTGTGTTTGTTTTCTTTCTTTCTTTCTGTCTGTCCTGCAGTCCTCCTCAACTCCTCAGATTCCGTGATTTTTGGCCATCAAGTCAACCTGACTTGCAGCCTTGGCCACGCCCTGACCCCTGAACTTCAGGTGAAATGGATCCCACCCAAGCATTACTCCCTCTCCTCTCTGGGGCCTGAACCCCACCCTGCTACCATCCTGATCCGGGTAGAAGGAGATGGAGACCAGGAGAAATGGAGGTGTGAACTGCTGCGGAACGGATCAAAGCTGACGTCAGCCGAAATCAAGCTGAAGATAGGTGAGTGCAGGAAATGAGAGGAACAGGAAATACACTCACGAGAAGATGTGGTTGCAGCTTTTCACCTGTCTTTTATTAAATGTATGTGCAGCTGTAGATGTTTCAAGGTGGTTATGTGTCTCTTGTGTACAGAGAGAACCCCACTGAACGTATGGTTGTTTGTGACCATATGTGGCGCCACAGTCATCTTCATCCTCCTGCTCATTGTTACTGTCATCCTTATTCGAAGACACAGACAGGTATGGCATTTACCTAATTGTAATATTTCTCCAGTTTAGAACAGTCCTTATTTCCTGGGTTGATTAGAAATAATGCCTTCTCACACAGCCATTTTTTAGCTTATTTGTTCAGCTACATGCTCAAAATGTTCTCTCACCTCTGTTCACTAACCCCTATTAATCTTTCTTTTTCTCTTCCTCTCTTTCATTGGCTGACATTAGCGAATGTGTAGACGCCCCAGGCAACGATTTTGCCGCTGCAAAGAGTAAGTCATTTCTGGGGTAAAACATGAATAAAGTCTATGGTGTTATTTATACTAACTCACAAAGTAACCATAGTCTATTGGCATTTTTTTAAATTTGTACCTCTTCGCTTCGGTGTCTGTGCCTCTAACCAAAGCCATGCTTTTTGTTTCAGTCCAAAACCGAAAGGATTTTATAGGAACTAAAGTTGCAAAAGAGACCTTCTCAAGAGGACATCTCATGCCACTGATTTCAAATGAACTGCATAGCACTGACAGTTGTTTTAAACGATAGACTCTCAGATTCACACAATGGTGAGATATCAGTAAACACTGAACATCAGATTGTTATCCTTCAGAATTTTTGTTAATATACAATGAAAACATTTTAATGTTATTTTTTATCACAATCAATTCACTTTGATTTGTATTCTCTTTAATATTACTTGCTACGAATGCTGTTTTTGTTGTCCTTTTTTAGTTTTGTTCTTGTATTGTATTGATGATATTGTTTATATTACATTAATGCATATTTTTATACAGTTTGTAAATATTTTAACTGTGAAATAAACTTATTTAAAATTCAAAAATACTTTTCATAGGTACATTCTCTCTTTGCTCTTCTTTAAACCAAGTTGACATGCATATTGAATTATGTGTGTTCAGTACAGTTTGCAGAACTGTACTGGATTAGGCCCAGACAAGAAATGTGAACTATATTGGCCATCCAATCAGAAACTTTTATGATGCAATATTATTTGCTGAATCATCCATGACCCTCTATGCCTAGAACTCATAAACAGCATTTGATGGTTCCACTGGAGATCTGCCAAACACTTTGGTCATTTTCTACTAGTGTTAAAAACTAAGGTCTTCTTTCCCTTTGTATCTGAGCATGAGAATGATTTTCGAGGCTTGCGAAGAAGAACTCAGCATTAGGCATCT

The utg7180000135569 unitig encodes a full-length canonical CD4-1 sequence:

MQTVFNTFFLFFRRGTKMKNCIQSLIVFSAFISTAAVQEKYCMYAQVGETVTFTYTPQQT

ESQSYYMYWYSRAGGDAPLISRNPFGHISKPAKQWEDRLSSTGYSLTIKNVKEEDFQTFT

LNLKINRETQITATYELIMVTMGVRPDYILLAGESLTLSCTQMSHSCAQPQIHWLSPQKM

SMGQPGQPTLIVRNVTGQHTGVWTCVLSHNGKQSFVTSSVKVVDLSPSPPHPVYTSLSTS

PRLHIPCSFPLLFTWEDLRTKDIVGGHWSFTPDQPPLPGGPQKLFSLSTGDPPAWQSDQE

RGLTVNDLGKNDLSLTKKSVTEGDRGTYTCSLEFRNNVMSRSTRVEVLQVLLNSSDSVIF

GHQVNLTCSLGHALTPELQVKWIPPKHYSLSSLGPEPHPATILIRVEGDGDQEKWRCELL

RNGSKLTSAEIKLKIERTPLNVWLFVTICGATVIFILLLIVTVILIRRHRQ RMCRRPRQR

FCRCKDPKPKGFYRN

***Percopsis transmontana CD74a***

>utg7180000203314 length=2563 num_frags=639 Astat=144.00

CACACACAAATATGCGTACCCATACACTTCTGGTTGAGGCTATATGTGTATCTCAACCCTCTGTGATTCTCAGGGATTCGAGTCCTGGATGCGTTACTGGCTGATCTTCCAGATGGCCCAGGAGAAGCCGGCTGAACCTCAAACAACTCAGCCAAGTGAGTGAAATCCCTTCACTACTCGCCACCGCTGCATGTCAGTAGAATAGAACCAGAATCTCCAATTCCCACCACTGTTTATATTTTGAAATGAAACTGCACAAAAGTGAATGTCACTGTAGAGGGAGGGAGGGACAAATTGAGGAGTGTAGATTTAAATAAGAATCATAAACAAGAGAGAGAGAGAGAGAGAGAGAGAGAGCTGGGTTTGTTTAGAGCGATCAAGACATAGACATGCACCGCCATAAACCATTCACTCTTTGCTTTAGGATCAAGCCTGAACTCTACTAAAGCCCATTTAACCCTACAACATTACACTCTCCATCCCTCAGCCTCCGTCCTCACGAAATGCCAGGTGCAGGCTGCCATTGTGAGGCCCGGTTACTACAAGCCCCAGTGCGATGAGCAGGGCCACTACAAGCCCATGCAGTGCTGGCACAGCACTGGCTACTGCTGGTGCGTGGATGAGAGCGGTGTCACCATTGAGGGTACTCTGATGCGTGGCAGACCTATCTGCACCCAAGGTAGAGCACACACGTACACGAAAAGGATGTTATAAGGTTTTAGTGTAATTTATTATTATGATTATTGTTTTTGTATGAATAGTTTTTGTTTTATTATGTTTAAGACATTGATTAAATGAATGGGTTTTATCGCTAGGCTCTCGTCGCATGATGGCTTACCACAGACCAATGATGCTGAAGGCCCTCAAGGAGGATGGTGAGTATCAAACACCCTTTACAGGGCCTTGTACTTTATTTGGAAATGTTTGGATGGCTATTTTTTAATAATTTATTTGATACTTTTCTTTTTCCAGGCAAGTAAGAGGATTCAAGCAGAATGCTGGGGCAAGAAAGCAAGACTTCATACAGCTCTCCTATTATATAGCAATCATACATATATTCATATACAGTATATCTTTACATTATTCTTTTTCCTCTTTGCAGCTTGCCTTATACAATAAAGTGATTAAATAGAGGCCTGTTACAAAATCAAAACAAAGATTTTCTTTATGTACTGTATCATATGCTTCAGTGTTTTCCTATTCATATTTTTAACTTTAATTTGTATATTCTACCATTTGAATATGGAACCAGTTCTGGAAGTGTTTCCTCTAAAAAGGTGTTGAGAAGTGTGATGAAGTATTCTATTATCGCATTAACAATGACGACTTATAACTGATCTACAGCCAATTACTTACTGGCTACACATGACCATGTAACATGCCAGAATGAAAAAATAAACTTGGTTGGATACTGTGAAATAGCTGCCATATTTTTAAAGGAAAGTGTTCCTGTTAAAGCTACATTATTCATGCTCACATTTGTAAATCTTACCTATGAAAATAAGAAGGTATCATAAAGCCATGGTATCCTATTGTGGCTAAGCTCCACCTATGCAATTCAGCAGCCAATCCGTCTGCACCCGAGAGGGAGCTTCCACGTTGCATGATGTCACCATGTCCCAAGTTGCATGACATCAGCATACCCCAATATGGACTTCTGGTTCTGGGGTGATTTCTCTATGGGTAAATTAATGAGAATTCACAAGATAGGTTTTTGGTTGGTGTTATTGACTCCCAACTGAAAGGATATGACTTCGACATGTGCAACCAGCTTGTAACGTGCATCCTTGTGCAAGATGCCCAACTCCTACCGGCTCCTTAATACTTGTATTTGTACTTACTGTAAGACACTTTGGATAAAAGTGTGTGGGCTACTAATTGTGTCTGATGCAGACAGTCAGCTTTCAACACAGGCTTTTTAAACTCAAAGTTTCAAAGTTTTACCTGCATGTATTTGACTCTCTAATGGCACTGGGCCTGTTGATTTCTGTAACCACTTTAAAGTTGAAGCAGTAACTTTGCTAGCAACATGATGCATCACCCCCCCTGTCATGACAGGACAATCTGTTTCTGTGTTACTTGGAACAAGATTACAGAAATAAATGGACCCAGGTGCCTTTAGAGAGACAAGCTGTTTTAAACGATGTCCTCTAGTGGTCAGTAAACGGTTTATGCTTTTTTTACTTTGACACATTTCTTCAGATTGTTTTATTATTATTTATACTTGGTTGGTGATATAACATGATCGATGCAACACTTGTGTATGAGGCAGTTGTATTAGATTAAATTATATTAGATTCAACTTGTCATTGACAGAATACTGAGACAACGCAAACTGTAAAATGCAGTTTAGCATTAGTGCAAATAGTAGAAAGTTCTGTTTACAAGTAGAGCAACAGACATTTCCAAGCACATCACTACTGTATGAGGAAGATTGGTACTTCTACAATTCTCAGATAAGGGTAGTATCATGGATGGCTTAAATATAATTAGAGAACATATACATTTCCATTTTAATAGAACCAGCCACCACAGACAGCAACACCTAAACTTGAGCTGGGTG

The utg7180000203314 sequence only encodes the C-terminal part of CD74a, possibly because the unitig is too short. The encoded sequence together with the coding part of the unitig is:

10 20 30 40 50 60

CACACACAAATATGCGTACCCATACACTTCTGGTTGAGGCTATATGTGTATCTCAACCCT

70 80 90 100 110 120

CTGTGATTCTCAGGGATTCGAGTCCTGGATGCGTTACTGGCTGATCTTCCAGATGGCCCA

G F E S W M R Y W L I F Q M A Q

130 140 150 160 170 180

GGAGAAGCCGGCTGAACCTCAAACAACTCAGCCAAGTGAGTGAAATCCCTTCACTACTCG

E K P A E P Q T T Q P

190 200 210 220 230 240

CCACCGCTGCATGTCAGTAGAATAGAACCAGAATCTCCAATTCCCACCACTGTTTATATT

250 260 270 280 290 300

TTGAAATGAAACTGCACAAAAGTGAATGTCACTGTAGAGGGAGGGAGGGACAAATTGAGG

310 320 330 340 350 360

AGTGTAGATTTAAATAAGAATCATAAACAAGAGAGAGAGAGAGAGAGAGAGAGAGAGCTG

370 380 390 400 410 420

GGTTTGTTTAGAGCGATCAAGACATAGACATGCACCGCCATAAACCATTCACTCTTTGCT

430 440 450 460 470 480

TTAGGATCAAGCCTGAACTCTACTAAAGCCCATTTAACCCTACAACATTACACTCTCCAT

490 500 510 520 530 540

CCCTCAGCCTCCGTCCTCACGAAATGCCAGGTGCAGGCTGCCATTGTGAGGCCCGGTTAC

S V L T K C Q V Q A A I V R P G Y

550 560 570 580 590 600

TACAAGCCCCAGTGCGATGAGCAGGGCCACTACAAGCCCATGCAGTGCTGGCACAGCACT

Y K P Q C D E Q G H Y K P M Q C W H S T

610 620 630 640 650 660

GGCTACTGCTGGTGCGTGGATGAGAGCGGTGTCACCATTGAGGGTACTCTGATGCGTGGC

G Y C W C V D E S G V T I E G T L M R G

670 680 690 700 710 720

AGACCTATCTGCACCCAAGGTAGAGCACACACGTACACGAAAAGGATGTTATAAGGTTTT

R P I C T Q

730 740 750 760 770 780

AGTGTAATTTATTATTATGATTATTGTTTTTGTATGAATAGTTTTTGTTTTATTATGTTT

790 800 810 820 830 840

AAGACATTGATTAAATGAATGGGTTTTATCGCTAGGCTCTCGTCGCATGATGGCTTACCA

G S R R M M A Y H

850 860 870 880 890 900

CAGACCAATGATGCTGAAGGCCCTCAAGGAGGATGGTGAGTATCAAACACCCTTTACAGG

R P M M L K A L K E D

910 920 930 940 950 960

GCCTTGTACTTTATTTGGAAATGTTTGGATGGCTATTTTTTAATAATTTATTTGATACTT

970 980 990 1000 1010 1020

TTCTTTTTCCAGGCAAGTAAGAGGATTCAAGCAGAATGCTGGGGCAAGAAAGCAAGACTT

G K *

1030 1040 1050 1060 1070 1080

CATACAGCTCTCCTATTATATAGCAATCATACATATATTCATATACAGTATATCTTTACA

However, scaffold scf7180001425484 published by Malmstrøm *et al.*^1^ has the coding ability for a full-length canonical CD74a molecule.

>scf7180001425484

(the complementary sequence is shown)

TAGCATCTTGCAGTTGATGGAGATTTGTGGGATGTACATCCAGGGCACGAAGCTCCCGTTCCACCACATCCCAAAGATGCTCTATTGGGTTTAGATCTGGTGACTGTGGGGGCCATTTTAGTACAGTGAACTCATTGTTATGTTCAAGAAACCAATTTGAAATGATTCGAGCTTTGTGACATGGTGCATTATCCTGCTGGTACATGGTGGCCATAAAGGGATGGGACATGATAAGGGATGGACATGGTCAGAAACAATGCTCAGGTAGGCCGTGGCATTTAAACGATGCCCAATTGGCACTAAGGGGCCTAAAGTGTGCCAAGAAAACATCCCCCACACCATTACACCACCACCACCAGCCTGCACAGTGGTAACAAGGCATGATGGATCCATGTTCTCATTCTGTTTACGCTCCCACAGGACTGCCGCATACTGGATGTTTTTCCCTTTTCACACCATTCTTTGTAAACCCTAGAAATGGTTGTGCGTGAAAATCCCAGTAACTGAGCAGTTTATGAAATACTCAGACCGGCCCGTCTGGCACCAACAACCATGCCACGCTCAAAATTGCTTAAATCACCTTTCTTTCCCATTCTGACATTCAGTTTGGAGTTCAAGAGATTGTCTTGACCAGGACCACACCCCTAAATGCATTGAAGCAAATGCCATGTGAATGGTTGATTAGATAATTGCATTAATGAGAAATTGAACAGGTGTTCCTAATAATCCTTTAGGTGAGTATATACATACATACATTTACCAAAAATTTGAAATTATTTATTGTCCCAATATGCCGGTAAAGCATTGAAGCAGTCATCAATATCTCCGGGTATTCTCAGATGACCTGCCTGTGTAGCTTTGACACAGTGGCAATGTGCAGTAGTCACACACTACACTAAGATACAGACATGTGATGGTTTATCATTTATCTTTTTATTATTGCCAAGCAATTTCTGAAGGATTCTATGTAATGACGTGTGTGTGTGTGTTATTTAAAGTGTTACTGTTTTAGAATTCAATACAAAATTAACAAAACAATAAGGAAGGAAGATAGGAAGCCTATCTTTGTGTAGTTGCATTGTCTACCATGCCCATATAGGACAGTTCACCATACAGGTGAACTGTCTTTGTTAGCCTGTGATGCAAAATTATAGGTAGGGGCATGAGCCATTACCATAGATTATATCACCCTGTTCTACTGTGCTTTAACATTTTTCATCTGAAACTCATTTTAATATTTGTTGGTTATGAAATACATGCTACATATTCATTCTCAGCTGGATTTTCTTTTTTCTGATTTCCTCCGGATTCATAAAAAAAGAAAATAAATATGTTTCTTATTTGCGGTTACAGGATCCTTGTGAGGATAAAAGGCACAAAGACATCTGGTCCAAAGAGAAAACTTGTGACCGCTTCCCTAAGCTCCTTGTCATTGGACCTCAGAAGACAGGTATGAGACAGTACTTCAGCTTCCCCTACAGGTCAATGCTGGAAGTGTGATTGGGGATTTGCATTTCATATTGCATTGATTTAGCCAGTATTATTTTGAGATTTATGGAGATTTATACTTGTCTTAGAGGCTGAGAACATTGTATGTATTTTTTCCTTTATCGTTGAATAAAATATACATTATTCCCACAGGTCTAAAATTGTCCTAATGCAAAATTATGTTTGCTTTTGAGCGGTTGCTGTTAGACTTGTAACTGTTATCGTTTATTGAGATGTACTTTGCACTACTCTTGAAATGTTTTACCCTATCCTGCTTTTAACCAATTTTCCATCTTCCCAGGCACAACAGCACTCTACCTGTTCCTGGGCATGCATCCGGATCTGACCAGTAACTACCCCAGCAAGGAGACGTTTGAAGAGATCCAGTTCTTTAACGGACACAACTACCACAGAGGCATTGACTGGTAAGAAACCACATTCTTGTTGAATAATAAATGGTATGTAAAGTGAGATAAAAGAAATCGCAAACTGTAGACTTGTAGGCGCAGCTTCTGTTATTCAAAGCGGAACTGAATCTTTGTCAAACCCGCTCTAGCTGCCTAACTCTCTAGCTGCCTTACCTCTATATTACGCCCAAACGCATGATTTTTTTCGACGCCGCCATAACGACAAGGCTGGTATACGTGATCCATACTCACTACCGAGTCAACCTCTGCAAGTTTGCAATGTAGGCGACATTCTAGGACACTCTAAAACGCTTACTGTGAAAAAGGCTTAACGTGGCCTAATGTTCCAAAACATCAATAATCACCAAATTTCCCTTAGACATCTCTTGTCATAAACACTTTTCAAAACACCTGTCAAAAAAATCTAAACAGAAATCCTAGGAGTACGGACATTAAGCGTTTTCCTCTCTACTGATGCCCATTATAAAAATAAAAAAAGGTTAATGTGGCTTAATGTTCCAAAATAGCGTCCAATGATCACCAAATTTCTCTCGGACATCTCTTTTCATAAACGGTTACACCTGTCAAAAAATCTAAATGGAAATCCTACAAATATGGACAATCAAAACAGAAATCCTACGAATATGGACAACAAACTGGTTGCATGCATTCTCAAGTTACTCCAGTCTATCAAAAGGTCAATTCCACCTTAATGGCGGGTGCAAAAGAGGAACTTGCTTGGTTATTCTTTTTCTATTGAATTCATATAAGCATATAATGTGCTTCCAGTATTTAAGCAGCCTTTCGCAAATTATTCTGTCAGGGCAAAATGGCTTCAATAGCGATGTTTATAGCTAGCTGTAGATATGAGACCCTACGTGGTAGTGGCCCCAGGTCCCGCACATTCCCCTACAATCTGTCTGCTGTTCCTGCCAGGTATATGGAGTACTTCCCTCTGCCCTCCAACACCAGTTCAGACTACTACTTTGAGAAGAGTGCCAACTACTTTGACTCTGAGGTGGCTGCTCAACGCGCTGCGGCTCTCCTGCCCAAGGCCAAAATCATCACCATCCTCATCAACCCCGCCGACAGAGCCTACTCTTGGTACCAGGTCTGCCACAAGCCACCGTCAGCCACGCATTGTCACTCTTTAGCCTCTTTGCTCATTAACGAGCAGTGGGAATGTACGCTAGCAGTTAGCTTCTTACCTTATCCGCGTGTTTTTATTTGCGTTTTGTGCCCACAGCACCAAAGGGCGCACGATGACCAGGTAGCGTTGAAGTATTCCTTTCATAGCGTCATCACTGCAGGCCACGACGCACCCGTCAAACTACGGGTCCTCCAGAACCGCTGTCTGGTTCCCGGGTGGTACGCCGTCCACCTGGAGCGCTGGCTCAACCACTACCACTCCAGTCAGGTACAGTGGAGCAAATACACTCAACTAATGTCTGCCTTTTATGCTGGGTGTTTTCTCAGCATATAAAATAACTAATATTAATAGTTATTGCACATTGTCTCCTCCACAGTTGCTGGTCCTGGATGGGCAAATGCTAAAGACTGAGCCGGCCTCGATCATGGATAAAATCCAGAAGTTTCTAGGCCTGGCCAACGCTGTCAACTACCACAAGATATTAGCGTGAGTTTTTACTTCCCCTCTTATGTCCTGTCTTCCCTCCGGAAATACTAACTTCATGTATTGCATTCTGACCTCGCATTTACTGTAGTAGATAATTTCTGTGTATATAGGGGGTTTAGATATCTACAAGTTAGATATAAGAGCAGGGGTTTTACTACTGCATTGAAACAGGACACCGGAGGAACAGAGAATGAGCTCATTTTTTAAGGTAGAAAATGAAACCCACTTTAATAACTGTCCATTTTGTGACAATCTTCAAAAGCGTTTCTATACATTATTTCTTGACCGTCATGCTGTAATTATGTAAAGAGTTATTTTATCAAATAAAATGTAAATCCACAGGTTTGACCCCAAGAAAGGCTTCTGGTGTCAGCTGCTGGAAGGAGGGAAGACAAAGTGTTTGGGGAAGAGTAAAGGAAGGAGGTATCCCGATATGGACCCTGAGGTACGACAGTACTTCAGCTAATACTCATTGGTTCCGGATGCCCACTCTACTTCCGTTAGCCATGTTCATCATTTACACTTGCATCATTTTGCAGACGCTTTTAAACAAAGCATCTTACAAGTACATTTCTCAAGGAAGTTAAAAACGGCGATACCAGTCATACTAGTCACTAAGTAAACACATTCTATTAATAAAGCGCAGTCCTCAGCGCTAGATTATTAGGAAGTACTTTCAAATGTATTGCTAAGAACCAGTTTGAACATAAAGCAGAAGAAGATTTAATTTAGTTTGAAATATAGAAACAGACAATAGAGGATAGATAAGGAGGGTGGGGGTGCTATGTGGTGTTGCAGATGCTTGTATACAATTTTTCCATTTACCTGTCTTCCTTGCCCAGTCCCAGGTGTTCCTCAGGGACTACTACAGGGATCACAACATCGAGCTGTCCAAGCTGCTATACAGGATGGGACAACCGCTACCCAGCTGGCTCCGAGAGGAGTTGGTCCACACCAGGTAGCACCTCCGCACAGAGGGCCACAGCTCTATAGAGCGAACCACGGCTCAGACCCGCTGAGGGAGCACGGCCGAATGCCATGGAAAGTCAGAGGCCATCCCGGAAGTGATTTCAAAGCTATCAAAATGGAGATTAGAAGAGCGGTTGCCGAAAGCCCCGAACTCGATACATGTTGAATACATGTACATTATCTCCTTGAGATGGCTGCGGGGGGAAGAGTGCCTCGCCAGTGATGAATAACTGGAGCAAGGTTCTCCTGATTCCCCGGGCTCCTGTGCAGAGCCTTGCAGTGTTCAAGAACAAAATTCACCATGGCACACAGAAGGGGGGAGGTCTCTCCCTAAAAACCTACACACAAACTGTCTCCCTGCCTTGCATTTTCATCAGACCTTCTGTTCATATGTACACAAAGCTTGGAAATTTAAAGAACAGACATCCAAGTCTCGCTTAGTCTTAACAGTTCCAGCCCCGAGGATAGGGGTCGGAGCACATTTGAAGAGACATTTGGCTCCAGCTATTAGTCGTGTTGTATAATATGTGCCGTTTGGAGCACATTGGAGCTGCTGATGCCAAATGTGCAGAGATGGATGCATACTTGATTGACAGGCGAGGCATTAGTTGCAGATGCCTTTGGTTTCTAAAGGAATTGTGTGGGAGGAGAAGGGGACATTTTGGAGTATTGACTCAGGCTACATGTTGTAGCCTTATTCGATAGGCCTTGGTTGTATCTAATAACTGCATTTCAAGTGCCAGGACAGAATCTTCGAAACAATATCACATGAACTTCGGAGGGTTCCTTCTTTTTTGTTTGTTTTTTGTTAATATATATATATTTTTTTTTTTTACTGATTGTATTTGTTTTAACGTTTTTACTATGCCAATTGTGGCATTTTAGTAGAATGTACCACAATTATAAAACAACCGCTCTGATTAGTTGTTTTTTTTCCGAGGGGCCAGATGTATAGTTGCCTGATTTGTTAAAACTGTTGTTTAAACTTTCCTTTTAGTCAATTTCATGGCAATGGAATTGGCTAATGTAGGAAGGGTAGGAATAATGTTGAAAGCACAATATTTAAGGCACTCTAAAGTGAACACCATTCTTACACAGGTTACTTGGAAGCACACTCACAAAATGGATCCTGCAACAGTCTTTTAACATTACTGTAAGCTTTGGTCTCTGAGATACAAATGGGGTGTGCAGATGTATTTTTCCCTTATCGTACCGGGTTTCTCCAGGACTGCGTGTTTTGTCCTCTGTTTATTTTCATACTACAGCAGAGTTTGTCTTTCGGAAAATGTGTAAAAGCTGCCAAGCAAACAAACAGTATGACACGTTAACCATACAGAAGCTCAGTTGTCGGTCAAGGTATTACCACTGCAACTAAATGTAAGAAGCATAGGTAACAGAAGCTGCTCCTACTGCAATAGTTTTGTACAGTGTACTGACTTAATAACGATGCACATGTTGCTCTTCTTATTTCCTGACTGTAATGTACATAATACGCAACGCTTGGTAGGCCTAAATTATTGAATGCGTTTGGGTTTGCACACTCTGTATCAACCCCCTTTTTTCGCAGTGGCTACATTTACACACACATTTCACTATAATTTGGAATATGCTGATATCATATGGTTTGCATAAACTGTGTATTGTGAACAACTCCCTGTTCCGAATAAGGATGTACCGATAATAGTCCTGTTTTTAGGCGCATGTATACAATGCATTCAGAATATTACCTTTTTCATAACAAGGGCACTAACCGGCCTATTGTGTCCATGTAAATGTAGCCAGTGGGTCATGTTAGTACCATGTTAAGGGGATTAGGAGCGTGTTGTTGTCATTGGCTTTTCTTTTCTTTTCTTTTCTTTTTTTCTGTTCTATGTTGAAATTATTGTTCCCTCTCACATCTTCTCACTGCCAACTATTGTACAGTGGATGGATTTCTTCATGTAAAGTATTAAGTGATAAACGCATAACAGAAACGACAGTACTAATCTCCTGGACTGTATGATAAAGATTTGTAATTGTTGTCAATAATTTTTACAGTGTAACTAATATTGTCGGTGCTTTTTAATTTGCAAATAAAACACCACTGTATTTTTCAGTAACGCTTTGTTTTTCTTCCCCTCCCTTTTCTTGTTCACTGATAGTCTCTGCCTCTGTAAAATGGGCGAAGTGCAGGGCTATTTACTAGTTTCTTAAACTGTTAAACTGATCAAATTCAAGACTTCAGAATCAATGCAGAATATGTAATGAACATGCTCACCGAACTGAAGCCTATAAAGTATTTAGTTTTGGGTTATAATGGGAAAAGTTGTGTTCTTATTTTTCAATGGATACTTCCTTAGGGGAAAGTACCTTAACAAGCACTGAAGAGCAAAGAAAATCTCTAGAAATTCACCTTTTTTTTTTTAACACCAAAATAATGTTGCATTTTTTACCTCCTCATGATGCAATGGCTCTGTATATTTGTTTATTGCCCTGACTTCGATTACCAAATATCTACCAGATTTTACTGTATGAAGTTATTCAATAATTTGGGCTACAACTATTATTTCTCTAGTTGATTAATATGTAATCATATTTTTGGATTATTTGACTAATCTATACGAAATTTAGACCATAACAGTAAAATAAGGTAATATGGTACAAAAATAATGGTAAATGCTAAAGGTCTAAAAAACTACTTCTCATTTTATAATATGGAAGTTTAAATTGCCAAATATCTTAACATGATTTATGACTTAATACATTTCTAATTGGTAATTTTGGGTTTAATCTATCGATTAAACGAGTATTCCATTGTTTCACCCATTAAATTACTATATACTTCGTCATTATTTGTCATACATTTTTCTGTTAGAAACATAATTCATCATTATAACATATATATATAAGGCCAGGTTTTGCTGTTATATTGTATCCTTTCCTTTGTAGTCTTCCTCTATCAACAAGTGATGGGGAAATCTGTTAGCCAATAAGAGTGCAGAAAGAAAACTCAACAGAACCAGCCCACTCTCCTTTACAGCCTGGGACTTTCCACTGTTAGTTTCACTTCATCTTATCCTGGTACCTCCCGCAAAATTACCTCAGCTCACAGGTCTTTTAATACTGCCTTGGACACAGTCGCTTCAGTTCTATTGATCACATCCAGAAGACCCTTAAAGATAGATGGACCAGCCACGTGAAGATGCTCACCTGGCCACAGGGAGTTTGGCTGGGAGCGAGGAGGCCCTCATTCTCCCTGAAAGAACAAGAGGGTGAGACAAATTTATCTCTTAATCAGGGACCATAAAACAATCACCTAATTTAACTTTAGCTAGATCTTTTATTTATCTTTTATTTATTATAAAGTAAAAATTGCCTTGTTTGCCCTTAAAAATGTTTAGGCTAGCTAACAGACCATGTTAGTATTAAAGTTGGAAACAACAAATATTCCATAAAGCCAAAACTGTTTACATACCTTGTAGAGAGAAGTAGGTAAGCTTTCTTTTGCACAGGCAAAAGTTATCTGCCTAGAAACGGATGATGCCTCTCATATTCGTGGTTCATAGTGACCAAAATTAATTATTTCCCATTAAGAAAATCACATTGTGAACAACTTTAAGGAACTGTATTTAAGACTGCAATAATTAAACTTAACACAAAAATATTGGAATCAATATTAAATATGCATAATGGGTATCACCATGCTATTGTGGACAACCTTAATGTTTGTATTTAGGCATACAAGTGAAAATAAAAATTAGTATATATAATATAGTATACATAGCAAAATATAATATATATAAGAAATCATTATTGCATAATACCTTTCAAAACATTGAAAATAACATAGGAGAAGTTTTGCACAAGGAGAAGTTGTCTGCCTAGCAACCAGTGATGCCTCTCATATTATTGGTTCACATAACACGATACAATTACATTTTCTCTGTTGCTTACAAAGACATTTTTATAATTTTTCCTTGAGAAATAACTTGTTTGAAAGAACTTACAGTAGTGCTTAGCAAAGAGTTAAGATTTTGTTTTGCTTTTCATTTCGAAAATAACACTAAAAGTCACTTCATGGCACAGGTTTCAGGGCCTTAAGTTTAAAGAACTCAAAATACATTGCAATTACAACAATTTATACCATTTAGAACATTTTAAATATAAAATAAATAAATGAAATATCTTATAGCAGAAGTAGACTACTTATACTATCGTTTGCACAGGAATAAGTTATCTGCCTAGCAACTAGTCATGTCACTAATATTATTGGCTCAAAATAAGCAGCAGAGTTTTACATTTACATTTACATTTAAGCATTTAGCAGACGCTTTTATCCAAAGCGACTTACAATTAGTAGCCTAAATTACTTCATCTATTAATTTGCACAATATTTTTATTTTATTATTATTTTTAATATTATAGTTTATGTTTGGAAGAACTTAACAGGAGGAACATGCTTTTTGTTTGGAAAACCACATTAATCAAATAATGTAATTCAGCTCAACACACAAGTTATGATACAAATATGAAAAATGTAAAAAACCCAAATTATTATAAACTCTGCTACTGCTTCCCCATTTTAGGTTACTTTTACTTTCACAAACCTCCCCCCTTATTTTATACATGTATTATGTACACCTACTGTTTTTTTCACATTACATTCTTTCTTTTTGTGTGATCAGGGGCTCTAACAGACATGCGTTCAAGGTCGCTGGGCTGACCACACTGGCGTGTCTGCTGCTAGCAAGCCAGGTCTTCACGGCCTACCTGGTTATCAACCAGAAGGGCCAGATCCAAGACCTCCAAAAGAACTCTGAGAGCATGCAGAAGGATCTGACTCGTAGCACCCATGGTAAGGGCGGTAATGCTATAGCAATGAGCAATCCACTTGTGTTGCCTATTATAAAAGCTTCAATTAATTTTTGATTTGTAGGAATTGAGAAGGTTAAAACATATAAACAGATTAATTCTTGAGAGGGGGCATTAGGGACTGAGATATTTATCCCCATGAGCATTCAGGAGTGGGCTGTCTATTTCCTCTTCTTCAAATGAGTCATGCCAATACAGTAGGTCTGATAATTAAAGGGTAACTAAACCTGATGTTCTGGCGGAAGTACATCTACATTGAGGAAGTATTCCTCAACTAAAAAATCTTTTGGGAGTTCTGTTACCCCTTAAGTAAATGATGAATTGTTTTACCTAAATAGCCGCAGCAGTGTCCCAAATGCGGATGCAGATGCCCATGAAAAGCCTGCCCCTTCTGACAAACTTCATCGACGATGACGAGACAGAGACCAAAACACCACTGACCGTAAGTCCCCACTAGTCTCCACATGGCAGCAACATTATTTAAAAATATGCTATTAAATTTCAATTAAATTAAAAAATTATAATAATGATTAAAATATTTTTTGATTATGTAGAAATGTAAGGAATTCTTATGTCACATTAGAATCATAAATAAGAAAGGATTATATAATGTTAATAATTTTTTTTAAATAATAATATTACATTTCTCAATCCCAACAGAAGTTGGAGGACACTGCTTTTGTCACTGTGCAGAAACAGGTGAAGGATCTCCTGCAGGTAAGCTGAATATATACCGTCTTGCCATGCCACACACATACTGGCCCATGCTATCTGTGTTAACCGCTTGCTTCTGTTTGGACCAGAGCTTCCAACTGCCACAGTTCAACGAGACCTTCCTGGCAAACCTGCACAGCCTGAAGGAACAGGTGGAAGAGGGTGAATGGAAGGTNNNNNNNNNNNNNNNNNNNNCACACACAAATATGCGTACCCATACACTTCTGGTTGAGGCTATATGTGTATCTCAACCCTCTGTGATTCTCAGGGATTCGAGTCCTGGATGCGTTACTGGCTGATCTTCCAGATGGCCCAGGAGAAGCCGGCTGAACCTCAAACAACTCAGCCAAGTGAGTGAAATCCCTTCACTACTCGCCACCGCTGCATGTCAGTAGAATAGAACCAGAATCTCCAATTCCCACCACTGTTTATATTTTGAAATGAAACTGCACAAAAGTGAATGTCACTGTAGAGGGAGGGAGGGACAAATTGAGGAGTGTAGATTTAAATAAGAATCATAAACAAGAGAGAGAGAGAGAGAGAGAGAGAGAGCTGGGTTTGTTTAGAGCGATCAAGACATAGACATGCACCGCCATAAACCATTCACTCTTTGCTTTAGGATCAAGCCTGAACTCTACTAAAGCCCATTTAACCCTACAACATTACACTCTCCATCCCTCAGCCTCCGTCCTCACGAAATGCCAGGTGCAGGCTGCCATTGTGAGGCCCGGTTACTACAAGCCCCAGTGCGATGAGCAGGGCCACTACAAGCCCATGCAGTGCTGGCACAGCACTGGCTACTGCTGGTGCGTGGATGAGAGCGGTGTCACCATTGAGGGTACTCTGATGCGTGGCAGACCTATCTGCACCCAAGGTAGAGCACACACGTACACGAAAAGGATGTTATAAGGTTTTAGTGTAATTTATTATTATGATTATTGTTTTTGTATGAATAGTTTTTGTTTTATTATGTTTAAGACATTGATTAAATGAATGGGTTTTATCGCTAGGCTCTCGTCGCATGATGGCTTACCACAGACCAATGATGCTGAAGGCCCTCAAGGAGGATGGTGAGTATCAAACACCCTTTACAGGGCCTTGTACTTTATTTGGAAATGTTTGGATGGCTATTTTTTAATAATTTATTTGATACTTTTCTTTTTCCAGGCAAGTAAGAGGATTCAAGCAGAATGCTGGGGCAAGAAAGCAAGACTTCATACAGCTCTCCTATTATATAGCAATCATACATATATTCATATACAGTATATCTTTACATTATTCTTTTTCCTCTTTGCAGCTTGCCTTATACAATAAAGTGATTAAATAGAGGCCTGTTACAAAATCAAAACAAAGATTTTCTTTATGTACTGTATCATATGCTTCAGTGTTTTCCTATTCATATTTTTAACTTTAATTTGTATATTCTACCATTTGAATATGGAACCAGTTCTGGAAGTGTTTCCTCTAAAAAGGTGTTGAGAAGTGTGATGAAGTATTCTATTATCGCATTAACAATGACGACTTATAACTGATCTACAGCCAATTACTTACTGGCTACACATGACCATGTAACATGCCAGAATGAAAAAATAAACTTGGTTGGATACTGTGAAATAGCTGCCATATTTTTAAAGGAAAGTGTTCCTGTTAAAGCTACATTATTCATGCTCACATTTGTAAATCTTACCTATGAAAATAAGAAGGTATCATAAAGCCATGGTATCCTATTGTGGCTAAGCTCCACCTATGCAATTCAGCAGCCAATCCGTCTGCACCCGAGAGGGAGCTTCCACGTTGCATGATGTCACCATGTCCCAAGTTGCATGACATCAGCATACCCCAATATGGACTTCTGGTTCTGGGGTGATTTCTCTATGGGTAAATTAATGAGAATTCACAAGATAGGTTTTTGGTTGGTGTTATTGACTCCCAACTGAAAGGATATGACTTCGACATGTGCAACCAGCTTGTAACGTGCATCCTTGTGCAAGATGCCCAACTCCTACCGGCTCCTTAATACTTGTATTTGTACTTACTGTAAGACACTTTGGATAAAAGTGTGTGGGCTACTAATTGTGTCTGATGCAGACAGTCAGCTTTCAACACAGGCTTTTTAAACTCAAAGTTTCAAAGTTTTACCTGCATGTATTTGACTCTCTAATGGCACTGGGCCTGTTGATTTCTGTAACCACTTTAAAGTTGAAGCAGTAACTTTGCTAGCAACATGATGCATCACCCCCCCTGTCATGACAGGACAATCTGTTTCTGTGTTACTTGGAACAAGATTACAGAAATAAATGGACCCAGGTGCCTTTAGAGAGACAAGCTGTTTTAAACGATGTCCTCTAGTGGTCAGTAAACGGTTTATGCTTTTTTTACTTTGACACATTTCTTCAGATTGTTTTATTATTATTTATACTTGGTTGGTGATATAACATGATCGATGCAACACTTGTGTATGAGGCAGTTGTATTAGATTAAATTATATTAGATTCAACTTGTCATTGACAGAATACTGAGACAACGCAAACTGTAAAATGCAGTTTAGCATTAGTGCAAATAGTAGAAAGTTCTGTTTACAAGTAGAGCAACAGACATTTCCAAGCACATCACTACTGTATGAGGAAGATTGGTACTTCTACAATTCTCAGATAAGGGTAGTATCATGGATGGCTTAAATATAATTAGAGAACATATACATTTCCATTTTAATAGAACCAGCCACCACAGACAGCAACACCTAAACTTGAGCTGGGTG

The full-length canonical CD74a molecule encoded by scaffold scf7180001425484 is:

MDQPREDAHLATGSLAGSEEALILPERTRGGSNRHAFKVAGLTTLACLLLASQVFTAYLV

INQKGQIQDLQKNSESMQKDLTRSTHAAAVSQMRMQMPMKSLPLLTNFIDDDETETKTPL

TKLEDTAFVTVQKQVKDLLQSFQLPQFNETFLANLHSLKEQVEEGEWKGFESWMRYWLIF

QMAQEKPAEPQTTQPTSVLTKCQVQAAIVRPGYYKPQCDEQGHYKPMQCWHSTGYCWCVD

ESGVTIEGTLMRGRPICTQGSRRMMAYHRPMMLKALKEDGK

***Percopsis transmontana CD74b***

>utg7180000004539 length=14838 num_frags=3794 Astat=851.00

(the complementary sequence is shown)

GCTCGACTCTCTCTGTCATGTTGCTAGTGGGTCGGCCTGACATTTAGTGTTTAGGCTAGACTGGTAGATTCTGTTCCTTTCCAGTTCGCGCGTCATAACTCAAGGCTGGAGAGGGATCTGTGGTTTTCCACAGTAAAAGAAAGTAACTGGTCCCCCCACCTGGAGGACGGGAGCAATTGAAGTGTTAAAGCTGCACAATACCGATCGTAGCGACGGAGGTGCCAGTCTGCCAGTCGAGGTGGTAGATCTAAGTGCTCTGGCTGGGGCTTGGAATTTTACCATAGCTTGGAGGTAGAGTGGGGCAGTTCCTTTGGCTGCTTTGTAAGCCAACACTAATGTCTTAAATTGAATGCGGGCTGAGACGGGGAGCCAGTGAAGGTCTTGAAAAAGAGGGGTTACGTGGGAGAACATAGGTTGGTTAAATACTAGGCGGGCTGCTGCATTTTGGATTCGTTGTAAGGGTCTGGTAGCAGAGACCGGTAACCCAGCGAGTAGAGATTTGCAGTAGTCTAGACGTGAGATTACTACAGCTTGGACTAGGAGCTGTGCTGCTTCGTTTGTGAGGAAATGTTGGAAAAATATATGCTTATTATCCAATTTTTAAAGAATGCTTGATATATCACTCTAACCATTGTTCTTTCTTTAAGTAAGAAAGAACAGGATGTACTCTTTTCAAGGTCTGCGCCTAAGATGTTTAATTGTTTTTCACGTCATAATTTTCTTAGTGAACTTCAGTTGACTCTAGTAGACCATTGTATTTTAAGAGTTATGAGGGGAGTACAACATAGGGATGTCTCCCGGTGCATCGGATATCAGGCACAATAGAAACGACGTGACCATGAGTATCACACATACACACACACAAGCCCTCCCCTATTAAAATGATTGTGTGAGCTGAAGTGAAATTTACTGACTTACAACCAGTAAACGGTTCCCTTGCTGCAAGTTTGAATAAAAGATCTACTTGGAAAGTGGTTGTCCTGAAGGTTTGCCGGGAGGTAGACTAGCTCTGTCTTTTCAAGGTTGAGCTTGAGGTGATGCAAAGACATCAAAGCACTAATGTCAGCGAGACAATCAGAGATTCTGGTTGTCAGATGGGGGGAATGAGAGGTAGAGTTGGGTGTCGTCAGCATAGCAGTGGTAGAAGAATCCGTGAGAAGAGATTACAGAGCCAAGTTATCTAGTGTACAGTGAGAAAAGTAAGGGTCCTAGTAGAGATCCTTGCGGAACTCCAGTGGAGAGCGGGCAGGATTTGGACAGGCGTCCATTCCAGAGGACTCGATCTGTCAGATAGGACTCAAACCAGGAGAGCGCCAAGTTGGCAATGCCCAATTCAGCAAGGGTCGATAGAAGGACCTGATGGTTTACCGTGTCGAATGCTGCAGATAGGTCTAGGAGAATAAGGACTGAGGAGAGGGAGGCGGCTTTAGCGGTGTGCAGGGCTTCCGTTACCATGAGAAGAGCGGTTTCAGTGGAGTGAGTAGCCTTGAATCCAGATAGGTATGGGTCCAGGAGGTTGTATTGTGAAAGGTAGGAAGATTGTCAGAGACTGCACGTTCAAGTGTTTTGGATAGGAAAGAAAGTAGAGATACCGGTCTGTAGTTTTGAGGTGTAGATGCGTCTAAGGATGGTTTCTTAAGTAGCGGTCTGACTCTAGCCATTTTAAAGGCAGCTGGGACCAAACCTGTGGTCAGGGAGGAATTGATTAGGGATGTGAGGAATGGTAGGATGTCATTAGAGATGGTTTGGAGAAGAGAGATAGGGATAGGGTCAAGTGGGCAGGTAGTGGCCCGGTTGGATACAATTAGTGTTTTTATGTCTTCTGTGGAGAGGGCAGAGAAAGTGTTTAAATTATCAATAGGCAGCGTGGGAATAGAAGGGATAGAGGGAGGTGCAAAGGATTGGCTAATGTCATTTACTTTCTCTTGAAGGTTTCAGGTTTCTTAAAAAAGTTCCTGTATATACAGTAGGCTATATATAGTTGCTATATAGAGTAGATAGGGTTAGGTTAGTGACTACAGTAGTAGTCACTACTATACTATTACTATAGTAGTGATTATACAATCCTCATCACTTTGTGAATATAAGGCTAATAATGACTTTTGTATATCCCGACATCGTATGTACTGTAAGACACCCCAACATGTCTAAATATCCATCTCTCCTCTGTTCCACTAGTTAATCATAGTTTATTTGTTTATTTTGCTGTGCTTCCGTGACACTACCAGACAAAGAAATGTACATGTTTACTGCCACCACACTTTTCTGGTCCTATGGGCCATTAACTAATATTAACAAATCAGTTTGTTAACAGTAAGCATGTGCAAGGCAGAGATATAAAATTTAATGAAAGTTCATAGTTAATACCTTGTTGGAAAAGTGAAAGTAAAAGTAAAAGTAACCCATCACAATATTACTTGAGAAAACAGCATAAATTATCTTCCATTTATTATCTGATAATTAATGTATGTAAGTATTAAAAACACAAGTAACAATGAAGTGTGCATATTATGTGCTAGTAGGGAGTAACAAGGTTGTGATAGATATTGTAATTTACCATATAATTTTAAAAAAGTAAACAAAATGTAATGTAATCTTCATGTCAGAAGATAAAAATATCTACATGTGGAAATGTGAATCTTATTTACTTAATTCATTGGCCATAGTGGAGATTACCATTTGCATGCAGACAGCAGTCTGCTCTGGAAACTGATGATGCTTACATGAAGAACCAATCAGGAATTAGCTGTGGAACAAAAAAATGATCTAGGTGGTTTTTCCCAGACTACTATTCTCACTTCCCTCTTTCTACTAGATAAAACTTTGTGGACTGTGATATAGTGATTGACGTAGTGATAAAGGCAGCTACTTAAACCAAGGTATAGATTGTTTGTGAATTGTCTGTGTACGTATGTGGCTATGTGTATTGTTTCTCACTATCTTTAGACTCCACCTGAATGCCTATGCCTGTAGTGTGTGTTTGTGTATTTCTAGAATTCTGTAAAGATGTCCCATTCCGACAACCAGCCGCTGATGTCCACTCCTAGTGCGCAAACTGCCATTAATGTAGGCCAGACAGCTGACAGGTAAGGTGTGTGTGTGTGTGTGTGTGTGTGAGTGTTAGAGAGAATTTGTTTATTTCATTTTCAAAGTTTGTTTTGTGTCCATGACACAATGTACTACTACTCTGTAACCCAGCGCTTTTACATGTAATTCAGGCAATGGCCATATAACTAAGCAAATCAATGTGATCATATACAGTAAATGTGCTTGACTCTAACAGCTTAGGAAATAACACTCTACACAAGTCTAATTTCCTTTTCTGGTTTAGCTTTGTAGGTTATTATTTTAGGTTATTTCTCTTGAGACAGTGTCAGACTTAAAGGTCCAATAAAAAAATTGTTCTACACAAAAAAATTTGAATATACAGTATTAGTAATAATACATATTTTCTTAAATATGTTTTAAATCAAGTACAAATAATTTTAGAGGTACTGAAAGTGAATGCTGAAGTGAAACGGAACTTCCACATTCAGTAAGAAGTCTGCGTTCAAAATCTCATAGCAAATTTCTAATAACACAAATTCCCAATATTATAGCTCAGAAACATACTCAATATTATTTTTGTCAACGTTATATTTGTCTAGACAAAGTTTTAAAAAACTGGAAACGGTTCCAACTAATTTGATCATTTTTAGCCGGCATTAATGAACACTGCTCATTAATGTAAAATAACACTGTAAAATAAAATAATCCAGCAGACTCTGATATGTCTCAAGTGAGTCATTTTAATAGCTTGACCAAAGGAAGAAGGCAGAGCACAGACACTGAGGTCAGGAAGTAAAAGAAACACAGATATATACTGTACAGTGGGGTCCACTACCTGTTTCTCTTTCAATTCTTTCCTCCTGCCCTGCTCCAGTCTGGTCATACCTTTCTATCTTTACCAATCTTGGTTTCATCACTCCTTAAAATATAACACTACATCAAGACCTTATAACATGATAAAAAACTTTAGGATGATTGTTTGGCTTCAGATTGTGTGCCTTATTTCCTTACATTTATTCATTTAACTGTTTTCTTTTGTTTTCCAGTGGATCCAATTCTCGTGCCTACAAGGTGGCAGGCTTCACTCTGTTGGCATGTCTGCTGATCGCAGGCCAGGCCGTGACAGCCTACTTTGTTCTCACCCAGAGGGGTGACATCAAATCTCTGACAGAGAAGAACAACGGCCTGAAGCAAGAGCTGACCAGGGGCAGCGCTGGTACATATGGAACATTTCTCACACTTCTCGGCCTCCGAGATTTGAAGCAGTGGAATGAAATAATGTGATTGTTCAAGTCCTTGAACTCTGTTTGGCTGAGTGACATTTAGGTGTCAAATACCTGGTAAACTAATATTGTGAAGGCTTAGCAGGCTGATAAAGACTGATATGATTATTGTGTTAAGTGTTCATCAGTTGTGCTGAGATTTAGAAATCCTCAAAATAGGCTACATAACGAAAAGGTAATACCTGCATTAGCAGCAGCTGTCTATACAATTAAACCTGCATCTCCCTCCTTTGAGGGAGGGAGATCCATTATTTTATCTATGTTATTATTTTATAATCACGTTATACCCTTCCCTATCGCTTTCTTAATCCGTCTTTCTCTCTTTTGGGTGCTTTAGCTGCTGTGCCAATGAAAACACACATGGCCTCCCCCTCTTTGATTGACTTTGATACAGAGGTAAAAGTGTAACTGATCAATTATAGTTGAATATTGTAATATATTGAATATAAAAGTTAGTCTTGTCCATTGACTATGTTATTTGACCTATTCTGTGATGTTTCTGTTCTGTGATGTTTTGCAGGGTGCGTCAGAGGCCTCTGAAAAGACCGGTATGTATTTCTAAATGCACACATCTGCCCATTCATCCCTCTCTCCATTTACCCTCCACCTAACCTAGCTACAGTATCTCTTAGCAACTGCAGTGGTTGTCAAACTGGGGGACAGGCTAGGTGGTCGCGAGATGTTTTTGATGGTTTAAAAAAAGTGTATTTTTTTTAAATAAAACATTGCATGTGTTTTTCAGTGACAGTTAAACTATCCTGTACTAACTGTAGTTTAGAGCAGGCTAAAATGAGATTAGATTTTGAGATTAGAGATTAGATTTTAGTGTTGGATGGATGAGTCGTCATTAGGTTTGTCTCAGTCAGCCAGTAGCAGACTAAACAGCACCCTTACTATCTTCTCTCCTGTTCTTCACCAAGCTTCAGAGAAAACAAAATGCCAGCTGCAGGCTGCCGACGGGCCGCCAAACCTCCGTCCCATCTGTGATGAACAGGGCCGGTACAAGAGCCAGCAGTGCTGGATGGACCAGTGCTGGTGTGTGGACACTTTCAGTGGCGAAATGATCCCCAACACCATGGGAACAGGACCAGCACGCTGCCCTGATTCCACCAACACCATGTCCACTGGTGAGACTTTATTACATTAGACTATCTCAATCCATTCAAAACATGCTGAAATCCATCAGAAACATATCCATCATATCCATCAGAAACATAACATACTGTAGATTACACAATAAGGTGTCCACTATAATTAAGTTTAATATCTACTGCAAGTCATGGCTTTCTCTTTTTAGGGGCTCTGCATCCTGTGATGGCTATGGCTGATGTCCTGAGCTCTGGCGGTAAGTACACGCCTCCTTTGTACCTCCTTCATTTTTGAAAATGTGTGTTCAATACAACATATAGTTCAGTGTTTCTCTCAACTGGTGAGTCCCCGACCAAAAAGTGGGTCGAGAGCTGGTGTGGAGTGGGTCGCGGACGCGTGCTTCGGCTCGGTCAAAAAGTCAAACTTATAGACGACTTTGGGTCGTGACCTAATGACCAAGGACAAATGTGGGTCCTGAGGTTAGACCAGTTGAGAACCACTGATATGAATGTTTTTTTTGTATCCCACAGATGAGTAATTCATCCTGAGAAGACCCCATAGAAGCATCTAAGACCAGCCTGTGAAATCATAATCCTACAATAATATTTAGATTCTACTTTAGATGGTAGTCTACTGGTGTGTAAGCTTCTGTACTAATTATCTTATTCTTGCATTATAACTAATATATACTCTATGCCAATGAGTACTTGAACTGCCACTGTCTCTTATCAGTGCATATAAAAGGTCTTTCATAATGCTGACCATGTGTCCCTTCATTGCAGGCATTACTATAACCACTATTTATGACAACATAGTTTGATAACTATTGCATTACCATTTGGCTGCAAATAGCTAAAAAGACAAAATAATTTAATACAAAAAAGTTTTTGTATTGGTAATTAATTAATAATTAATTGTCATCAACCGCTACTGAAATGTTTTCTAGAATAAAACACTCCCTAAATATAGTGAGACAAGATGGTCCTCAAGTCAAGCTAATGGGTATTTTCAAGAACTAATAATCTACAGTATTAGAGTAGATAAAAAAGCACTGACATTATAGCAAAAAGCATTAGGGGTTTTACAGACTGCATAGTGAGGAACTTAAAATGGTGTATTTCAATTGTATTTTAACACAATAAAAGGAATTTTTGGGGTCAATTTCAGTGTATTTAATTACATTTAGATAAGTTGCGAGATGAATGACAATTTGATGATACACTTTGCTGTAGCCGAGGTTATAATGTTCAACACACCCCGAGATGACATGGTAGGAAGTAAGATACGTACATTTGAAAGTTAAAATCATTAACTGAATTGTCAATGTAAATTGTTCAACATATAAAAATGTTTAGTTCCAAGGTATTTTTGTTTGAGAAAATAACAGAAGAAACATGCATGCAACTATGTAACAGACACAATACAACACATATAAGAGAATGCAATAAATAAATATCATTTTCTGGATTGTAGAGGCGGAGCTTGCAATATCAGGATGGTTTTAACCAGGCTATCACTTTTTAGCAGTATATCGTCTTTAACATCCATCATCTGGGCCCACTAGAAATGATTTCTGTACATTTTAAATTTGTACCTGAATTAGTTTTTAGGTTTTTAAAAGACAGCAGCATTCTCTCTAAACTGTACCAGATACAGCACATTTTAAGTGCATTCATTGAATCTAGCAAGTAAGATGGTTTAAGGACTGGATAGAGCTGTCAATTCCCCCTTTTTCTGGAAGAACAAGGTTTACACTATAATAACATCACCAGCATTACTACACATAATTGACTAGATACAATTATTCATCAACATAATTACAATCTCAGATAAAATAATAATAAAAATGATTTCCCAAATGGTAAAAAAAAAGTAGTTAAACCAGCTTTCAGTAAATTAGCACAGCAAAATATTTTTTTCTTCCAGCATAAAAACTATTAAACCAAATCCCTTGAGGACAGGTTATTGCACATATGGCCAAGAGATGACTCTGTCAGGTCAGTAAGTATAGGCACCCTAGGCAGGAAAGGGGCCATGTTCACACAATTCCAGCTACTATTACATACTTTTTAAAAGGAGTGCACGTGAAATGGAAGTGTCATACAGCCAGCACACACTGCATTCGGTTTAAGTGTGTTGTTTTTGATTTAACAAACTGAATTGTGTAAACATGACTTGAATTGGTCTTGGCATTACAAGTACCATCCAAAATCCCTGAAATGTAAAAGAAATACCAAGTGCAACATTAACTACAGGTTTATAATTATTGGTAACATTTTTTTGTGGCGTAGCACAAGTTTTGACTCCCTCATTTCTCTCCATCTCATTTACCTCCTCCTCTCAGGCTGCTGAAGTAGTCCCTGAGGGTTTGTTCAATGTTGGGCACAACGTAGTTCTGTGCTGCGAATATTCCTGTACAAGTTCCTACGGCGACGCCCATTAATGCTGAGGAGCGTAGCTTGGCAACAACATACCCAGCCAGGAAGCCCTTCAGGAAAGGCGAAGCTAGTAGACTGCCTCCCTGAAGAGGAGAAGGAGTAAGGACAGGACAGCAGAAGTGAGGATAAGAGACAAGACAAAGAGAGGGGGGAACAAAGATGAGGAGACTTACACAACTACTTTGTTTGTGTGTACTGGGGATAGGCTAGTGGTACTTCCTTGAGCAAGATGCCTAACCCCTACCTGCTCCTTGGAATTGTACTAACTGTAAGTCGCTTTGGATAAATAAAATAATTGTAATTGTGTCTATCCATGTCAATTATATATGACATAGATATTATAGTTCATCGGACACGGTGTGATTGACTGCACCCTCCGATAGACGCACTCACATTTTGGGACACTTGGTAATATGGTGGGACAAATCTGCCACCAGTGAACAGTGAGCTGCGTTACGCACATTGGTAAACATTAAGTCCTCTAACACACGATGTCACAACTAGGTCGCACGCATGTTTGTCAAGCCAGATTACATTTAGATTTGTTCTAGGTTATGATCACAGGCATGTAATTAGGACTTAAAAGACCTAATTAAAATTACATGACTTCTTTCTGGACAGGAATTCCACAAGAAAGCAGAGCTAATACTCTTTGAGAAGAACAAAGTTAAGTCTAACCTGCGGACACGTTTCTGGGTGCAGTAGACGGAACTTTTGCTGCGTTTGGTGAATTTTTTCAGTGAAAATGTTTGCTACGTTCTTCAATTCACTCAACTGTGTTTGCTGCGTATTGCGGGAGTGACAGACCACAAACAATCAGTTTGTTCTTAAATCAGTCATGTTGACTATTTGCGTTATTTAACAGTATACGCCAGTGTGAATCTTACTGGCGGAATCCCGGCCTGCATCTCATTCTCCACTCGTCGCTGGATATCCTCCAGCTGGTCCTTCAGTTCCACCAGGTCCTTTAGTTGGCCTATAGGATTCTACATACATACACACGTGCAGAAGTTAACCAATAGTAAACAAATGGTAACTTTCTGCTCTCTATATCATGCCTTTTGTCAAGCCTTTGTTACGTCAGTAAAACATGTTTGACATTTGTAAACAGTGCAATTGGGTGCAAGTTTGGGTAATAACCAAGAAACATAAAGTATTCCCTAGAATCAATAAGGCACATATCCATAGCTTGCCAACAGCCATTATTGTAGCTTTTAGAGCCTAGATTTTTTTTTTCTGTTCACGTTATAATTGATTTACAGAGATTGTATTTATGGAGCTCAGACAGCAACGATTTTGTTGACCTGTATGAGTCAAATAGAATGCCCGTTATATTAAAACATGCACTCTAGCAATATCATAATGTATATTATAACATAGAAGCCTGAGCTAAAAAGCAATTGATCATCTATGAATCTTCAGAATCAATTAATTTATTCGTTAGGTTTATTCGTTAGGTTTATCTAGAGGGGCAGGATAGTCTAGTGGTCTAGGTGTTGGACTCCCAGAAGGTCGTGGGTTCAAGCCCCAGAATTGGCTCACCTGTAAGCAAGATGCCTAACATTAAACCCTATCCGCTCCATGTAATTGTAAGTCGCTTTGGATAAAAGCGTCAGCTAAATTACCTAATTGTAATCTTTACCTTTTATCTGATAATCAATTTGGTTTGAATAGTTCTTAAATCTAAATTATAATAATATAAACAAATATGTGATTTCAAGTTTTCACTGAAACTCTTACCAAGAACTAACGTTAGTTGGGAAATAAGCTAGCTAGCAGTACAGCTAAAAGGTACAGTAACTAGCTCCTTTTTCGTTTCTAGCTCAGACTGAAGATTATGGACTCATTGATCGTGGACTTGCAGCTGAATGATATGTAGTATACCACTAACACACAATGTAAAACAAAAAAGTTTGAGTTACGTGAAAAGTTAGAGCTACTAACCTTGTCGTTCCCCATAATTTCTCACTCTGGTGTTGTTGGACGCTTCTTCTTCCACTGACTAGCAAATAGCGCCCTCTACAGGTTAATACCTTTATAACAGATGACAATTTTGTATCAAGACATGATACATAATTGTAAAATGAGAAACGAAGAAATACAAAATATCACACATTAAGGGAGTCAGCAACAAAATCCAAGGGGGAGGTAACTATGGTTAGAGAAGGGACATACAAAACTAGAAAACAAACCATCAGTGTTCTTATTATTATCAAACAAACTATGACTAAGATTATTTTCACAAAATAATCATTAAAACTACACAGTCTATTCAGCTCCAATCATAGCTCACCATACATACAGCTGGCCAATATTTAAATGCTGTTTAATAGAAGTCTTTGTAAAGGCATACTGTAACCTATTTTCAACAGAACAAAGTGCCCATTAATCTCCCGTTATTCAAGTTATCACAGACCCAAGTAGATTTGGGATCCAATATCGAGATTCCTTTTAACCTAAAATAAAATAGAGTTCTTGGTTTTGCACAAGATGGAATACCACCTGCACACTAGTTCAAACTACAAGGGAAAATTAGAGGTAAGTCCTTCTGATCTTCCCTGGCCTGGATCGTTTGGGAAGATGTTGATTTTGGCGAAGGCATATATGAGCATTCTTTGGACAGCATTTTGAAGGATAGGGCACAAAAGCACACTGCGTAGTCATTACCATTACCTACCTACAAACCTATAAACAGCCAACCTGGAAGTGAATGATGGACCTGCACTTGCTTGAGTGTCAGTTTGCGTAGATTTTGTGCAAGGTGCTAAAAAGAGCAGGGAGAAAGATGAAAGATTTGGCTCTACTTTAACTTTTTGTTAATTGTTCAGTACAGTCTACTCTTCAGCCTCAACTAAGAGCAGTGAAGGAAATTAATCTCCATGCCGTTACTGTATAAACAGTAATTAGAGACATAATTTGTGTAGGGGCAAACAAACAAAAAAGCAATTTGAATGTAAGGACATTTTCAGAGGCAGGGAACATAAAACAGTGTATAGTTTGCCGTGGGTATTGATTGACGCAGAATGTTTCCAAAATGGAATCAGTCGCCACTCTAGCAAAAAGGGAAAACATAACTAGTCCACACGTTATGCAGGCAGTAAGATACAGTAGAATTAATTTATTCAAATGAGCATTCAATGGCAAATAGAAGAAAGTGCTTTATGAGGTTAGGAGCACAGCAAGAGTTTAAGTGCCAGGACACCACTATAGTTGTATATACTATATAGTGTATAATATCCTTATTCTATCCTTATTCTATCCTCCCTTTTTTTTTTATAAAATAAAATAAAATAATATCACTTCTTTCTACTCTGTTAGATCAGCTCTAAGAATTTTAAGCACTTTGCAGTTTAGCAATTATTGACTGTTTGAAGAGTGAATGATAACTTTCGATTATTGTAATTATTGACTGTTTTGAGGAGCGAATGATAAACCTTCGAGTAATGTTGTACAAATGCATAGGTGAAGTAACCTTTTTACATGATGCTGTCCTTATTCTAGGAGAATGTCATCTGCTTGCAAAAACTGGTGGTCAACCAGTTCAACTGTTGACATATGTAATAATTGTAAGTCGCTTTGGATAAAAACGTCTGCTAAATGCTGTAAATGTAAATGTAAATGTAAATGTAAATATATATTGGCTCGGATTGTCCGTTCTAGTTTTGTAGCATGGTATTGCATTGCATCTGATCGACTTGGTATTCATGTAAAAACCTAATCACTGGTAAAGCTGTGTGGCTTCTAAAGTCTTTTTGTGTTTCTTGATGGTAAGTAAGGAAGGAGTCTCTAGTTATATCTTAGTACAGGCTGGCTGACTGATCGATGTGTGAAACCTAGGTGGTTTGATAGATGTTTCAAAAATGTAAGATCATTGTGTGAAAATAGTGTGCATGGCTAATCTTAACACAACATGTCGGGGGGCAGGATAGTCTAGTGGTCTAGGTGTTGGACTCCCAGCCGCAAGGTTGTGGGTTTGATTCCCCAAGGTAGACTACCTGTAAGCATCCTTGAGCAAGATGGCTAACCCCTCCCTGCTCCATGTAATAGCACCATGGAATTGTGTAAGTCACATTGGATAAAAGCATCTGCTAAATGATCTAATTGTAATGTCAAGGGAAAGGTTAAAACAACAACCTGCAAGAAGATGACACGTGATTGTGTGATCAACAGAAAAATGTGTTTAACTATGGCCTAACTCATGGAGGTGATGTACACTTGTTATCACTGCAGAAGATATTGTGATGTACATTTTCATTCACAAATGTTATTAGGCTAAAAATAAATGTTATTAGTTCCAGAAAATGGAATTTTCTAACCATGGGTTAAAATAGCTCCAAAGTTTTGTTGGATTGTTTTTTGCTAAATGTCCTGGTGTTGTGTTCCCTCATTATTCAGAGTTCAGCCAAGATTTCCCCAATGGTTCTTTGAGACGCTTTATTAGCAGCCAGGCACGTGCAGAGGGGGGTGCTCAGGGGGCCAAAGCCCCTGCGCTTTTCCCCAGAAATCAAAAGTGCGCCTCTCAACATTCATTTATTAAATAATTATATAGTGGTCAATGAAACAAAATGCATTTAAATTTAAATGAACATGACAACATACATAAAACAGTAAAAGGCATCATAAGAAACTCTGTTTCTTTGTCGTGCCTACCGCACAGGGGGTAGAGGTAGGATCTTCAGTAAACTACATTAGTTGACATGGTTATCACCCACTGTTATCCCATAACAGAGAAATTCTAGACAGGATTTGGCAGACAATATATTTAGCAATGTTGGAAGTGTTTAATCCTCATGGAGGTGGAAAGTGTATGCCCATGTCCACATGATCAGATGTCCCTCCCGGATACAATGAATGCTGGATGCCTGGACATATCTCCAGAGGATTGCCAGGGATGGATGAGGCATGCAAAGAGATTCTGTCCCAATCTAAATTTATCTTTTCTTCTCTGTGCATAATCAAGCTGGATATAGTCCATCATAATATTATGTGTGTAATAGCCTATCCAGATGGTGCTACAGTGTGTGTTTATTTATTAATGATTGGATTTTCCTGTAAAGATCGTGTTGGATTCATCTTTCCAAGCTTCCCTTTTTGGTAATTATTTCTATCAAGGTAAAATTAAAATACCAATACCAGACCCCACACAGTCTGTGTACAATGTTTACCAGGGCTATACAGTAGTAGATGCAGGGTTATAATTAAAACGTGTTTTTTATTGATAATTGTAATATTGGAATTACTTGAAATGAGCTCCATCGTGTTAGCTGCCCCTTTTTTGTTACATTTGAGCCCTTGCCCCTGTGAAATTCTTTGCATGTCCCTGTTAGCGGAGCAAAATGTGCTTGGAATACCGTGTAACTAACTGAGACAACTTTTCTGATAGTGCTAGATTGGAGATGTGAGATTCCTCGCAAATAAACATGACTTGTGTCATTAAGCTAAGGCTACTAGAAAGACCAGATAAAGCCTTTTGAAGCAGTTATATGAATGAGAAACTCATTCTCACTCTCCACTCTGAGAGTGGAACCTAGACCATATGTCTATAGAAGCCTGTCCTGTACCTTCAGTCTTGCCTAGGAGGCACAGTCACCACCCATTGTAACGGGTCAGGACGTACGCTGAAATAGGGACTACTACAAGCTATGCTATGAAAGCCTTCCATTTATACCTTACATTGATATAGAGCATAAACACATCTAGAGTCATAAGGCAGTGAACTACATTACCCACACTGCCGAGCCACAATGTCTTTGCACGTGTGGAGCTCAAAATACTAAGGGGCGTATCCTAAATGCCAACAACACTCAGGCTGTATATAGTTCCTCCCTGTATTTATAATGAATACCTGGTTTACAACATACATGGTGTGTATGTGTGTGTGCGCGCGCGTGTGCGCGCAGGCATACTTATATGCGGTTTGATCCGGTTGCCAGTTAGGAGCCAATTAGAGTTTTCACGCTCTGTTGGCAAGGATAAGGGGAAGAAGAGCTACTGCAGTTTCACTGCACCACTCTGCTGAGATGTGCCAAGGTGTGTGTGTGTGTGTGTGTGTGGAGGGTATTATAATAAAAGGAGAGGGAGCATTTTAGCAAAATACCAACTAAACAACATATTGTCAACCTAATCAAACAAAACTTACAACGTTCTTCTCCTACAGACAAGCATTGTCTGTGTAGTCAACAGCAGACACTGCTGTTACTGTAGAAGTACCTCCATCGTTGTCTAAAAAACAATTGAAACGAGTCACATAGCTGCACTGTCGTTTTTGTTGGCATAATTCATCATTAGGATGAAAACTAAGATCACACTTTTTCAAGAAATTACTAATTACTAATGAGTTTCTGCCAAACTCTTGTGTGCACCTCGCTGCTGCTTCATTAAGTTGTTTTGGGTTGCTCAACATAATGATAAATTATGCTGTACAGCTCTGTGGCCTGATTTTATATGTTTCTTTGTCTACAGCCTACAAAAAGAGAAAACACTACAATGTTTTTCTTCTAACCAGAACTGCCAATATAACCAAAAGCAGTAATAATCCTCACCTTAAAAGCTGTTGCTTCTTAAGAAGTTTCAGGATGAAATAGTATGGCTCTGCTCTTGTTATATTACTCTTTAGGACAACAACAGAGTCCTGTGGAAGAGCTATATCTGTTTCTCATCATTTTTGACCAGCAGTGGAGCTCTATGGCTGCCAAGGTACAGTAAGAGCGGTATATACTTAGTTAGTAGGAGAAACGTATTGTAGATTTCACCCTTTGTTGTTTAGGCTATGTTGACAATAGAGGCGAGTCTATCACATATATAAACATTGTTCTGCCATCTTTAATTTATTTTTACCCTCTTTTCTTTCACCTTTCAACCTCTCGTATTTTTATCACCGTCTCTTCTTCTCCTCTATCATTCTCTCTCTCTCTCCTCCCTACACTTCACCCCCCCCCTCCCCCACACACACACACACACACACACACACATGTATACTC

The utg7180000004539 sequence encodes a full-length canonical CD74b molecule:

MSHSDNQPLMSTPSAQTAINVGQTADSGSNSRAYKVAGFTLLACLLIAGQAVTAYFVLTQ

RGDIKSLTEKNNGLKQELTRGSAAAVPMKTHMASPSLIDFDTEGASEASEKTASEKTKCQ

LQAADGPPNLRPICDEQGRYKSQQCWMDQCWCVDTFSGEMIPNTMGTGPARCPDSTNTMS

TGALHPVMAMADVLSSG

**3.6 *Polymixia japonica***

**For the non-gadiform fish *Polymixia japonica* no intact classical *MHC IIA*, classical *MHC IIB* and canonical *CD74b* genes were reported.**

*Polymixia japonica* is a non-gadiform fish investigated by Malmstrøm *et al.*^1^ which is closely related to the gadiform fishes, and hence important for deciding the phylogenetic stage at which functional MHC class II system genes were lost.

In summary, our findings for their Table S7 listings are:

**MHC IIA:** Table S7 mentions utg7180000058066. This sequence encodes an MHC IIA molecule of the non-classical DB category, which does not have the two asparagines at positions -4 and +3 relative to the second cysteine in the a1 domain which are typical for peptide binding function in classical MHC class II (shaded red in Fig. S1; Zhou *et al.* 2009^3^; Painter *et al.* 2012^4^; Dijkstra et al. 2013^5^). The encoded sequence is also incomplete and lacks a predicted leader sequence, possibly because the unitig is too short. There are several other *Polymixia japonica* unitig sequences published by Malmstrøm *et al.*^1^, not shown in their Table S7, which contain *MHC IIA* gene fragments of the classical lineage DA (utg7180001526699, utg7180000038310, utg7180001357096, utg7180001269542, utg7180000601579), but they are too short for encoding full-length canonical proteins (not shown). We were unable to find *Polymixia japonica* unitigs or scaffolds reported by Malmstrøm *et al.*^1^ with full-length classical *MHC IIA*.

**MHC IIB:** Table S7 mentions utg7180001073264. This sequence encodes an MHC IIB molecule of the non-classical DB category. The encoded molecule does not have the two amino acids motif, HN, at position +3+4 relative to the second cysteine in the β1 domain, which are typically involved in peptide binding by classical MHC IIB molecules (shaded red in Fig. S1; Zhou *et al.* 2009^3^; Painter *et al.* 2012^4^; Dijkstra et al. 2013^5^). There are several other *Polymixia japonica* unitig sequences published by Malmstrøm *et al.*^1^, not shown in their Table S7, which contain *MHC IIB* gene fragments of the classical lineage DA (utg7180001350710, utg7180000400371, utg7180001690934, utg7180000381861), but they appear to be too short for encoding full-length canonical proteins (not shown). We were unable to find *Polymixia japonica* unitigs or scaffolds reported by Malmstrøm *et al.*^1^ with full-length classical *MHC IIB*.

**CD4:** Table S7 mentions utg7180001882239. This sequence encodes a full-length canonical CD4-1 molecule:

**CD74a:** Table S7 mentions utg7180001886650. This sequence encodes a full-length canonical CD74a molecule.

**CD74b:** Table S7 mentions utg7180000447839. This sequence only encodes a middle part of a CD74b molecule. We were unable to find *Polymixia japonica* unitigs or scaffolds reported by Malmstrøm *et al.*^1^ with full-length canonical *CD74b*.

Below the respective unitig sequences are shown with their relevant protein coding capacity:

***Polymixia japonica MHC IIA***

>utg7180000058066 length=3132 num_frags=309 Astat=150.00

GGAATCGTTTTTCACTAATTAGATCCGTACTTTGCAACAACTTACCAGGGACAGTGCGGGCCAATCCATGCCATCTACATCATTCCACTCAACTACAAGTTATCTTCCTGAGAACATGTAGAATAGCCAGATGGTTCTCTGTTGGATCATCAAATCTTCTCAACTCACTTCTTCCATCTCAGCTCCCTACCACGAGTTACACTACATCTACGGCTGTTATGACTCTGGAGACGTGCGAGTGGATGTGCTCATTGATGGGGACCAAGTCATGTACGCAGATTTTAATAAGCAGACGGCAGTATGGACGATGCCTCACATTCCCCCTGTGAAAGACTGGAACGTCGCTTACGACATCGCCAAGGCTGCCATCACACACTGCCACAGTGTCCTGAACAAGGCCAGACGGGCTGAACCCGGTGTTCCCACTAGACAAGGTACTTGTCCTGCTGTGTCATTGTTGGTTTAGCATGAATTTATCTTGGATAGAAGGAGAGAGTGCAAGAGGCAATCTGAGGAATTTGTGGTTAGTGTTAACACAACAACACACACACACACAGACATGTTAGATTAGATTAGATTAGATTAGATTAGATTAGATTATGCCATGGCCTGGCAAAATACTAAATTCCCATTGGCTCGATGCTGCCCATTATTTTTGTGTAATGTACATAATACATGCTGTGTTCTATTCTGCTCTGTTCTATTCTATTTGTGACCCAGAGGCTCCAGAAATCTCCATCTACAGCAGGTACAAGGCAGAGCACGGTGTGGACAACACTCTCTTCTGCTTGGTCAGTCACTTCTATCCCCCCAGCATCAACGTCACATGGACCAAGAACAGCCAGGTGGTCACAGAGGGGGCGTCCCACCTGCGTTACCACAGTAACAAAGACGGAACGTTCCACAGGATTTCCAGACTGGATTTCACCGCGGGGAAGGGAGACGTCTACTACTGTAGTGTGGAGCACCAGGCTCTGGAGAGCCCTTTAAACAGGACCTGGGGTGAGAGAGAGAGACTGTCTGTGCCAGGACTGTCAGTGCTCTTTGCTGTATCGTGTTGTTATTGGTTTTTTTTGTCATCTGTCTACTAAAGTCATCCACTTCTCTCCCTCTCCATGGAACTCACTGTGAAATGGGTTTTAGTAAAATGCGTATGTAGAAAATTACGTCAAAGTAGTTATTTATAAAAGTTCACTTGACGTGGAACCTGACGTGGAAAAGGCCATAAACCTATGTCAGGTTTTCATTCGACCTACACACATGACCTGCTGGTTAGGCTGTGGTATTACAGGCGTCAGGGAGGAGTGAGCTACGTGCAAACCTCAACGGTGCACTGTGAAAACTGAACTGTTAATTAGTCAATAAGGACATAACAACTCAATAACAACTCAATAACGACTCAATAACAACTCAATAACAACTCAATAACAACTCAATAAGGACTCAATAACAACTCAATAACAACTCAATAAGGACTCAATAACAACTCAATAACAACTCAATAACAACTCAATAAGGACTCAATAACAACTCAATAACAACTCAATAAGGACTCAATAACAACTCAATAACAACTCAATAACAACTTCATAACCACTCAACCTTCTAGATGTTTTACTGTTTTGAATCCCTGAGAGAACATAGTGTGTCAATGTCCTCTATGTTGTTACTACTGGGTTGTGTCATCCTGTTATCTTCATGTCTGTTGTTCTTTCTTAAATTTGCTTTGAGGTGAGCTGGGGCCTGAACACGTGCCTTCTCTTTCTGCAAAACTAAACTAAACTAAACTTTATGATTAAGCTACTTGTTTGCTGTGTCCCACAGAGCTGGAGGTGAGGGAGACCAGCGTCAGTCCTGGACTTGTCTTCTTTGGAGTCAGTCTGGTTTTGGGGCTGGTGGGACTCGGGACCGGAACGTTCTTCTTCATCAAACAACCAGCAGCAGTGATCTGACTGGGAAGAGTCATCATGGACAACCGGGAGTCTTGTCTTTTCAGAGATTTCTGGAGTAATTCAGCTATACTATTACTAGTATTCAGCTAGGATTATTATTCATTATAATAACCTTTATTTGTAGAGCAATTTTGCTGTGCTGAGAAAGGAGAATATCAGAATCCTTGTACCAGTTGTTCCTGTACCATGTGGGTCCTTGGGAGGATTAATAAAGTATATCAAATAAATGTTGTTGTAGTTTGAGTTTGAAGCTGTACTTGTGTGTTTATGTTTAGTCCGCTGTCATGTAACTCGTCTACCATGTGTCTGTCCTGTAATGTACTTGACAAATAAAAGCAATTCTGGTTCTATTTTTTTGTGGTTGAGTTGCTGTAGTTTTGTTAAGAAACGTCCTATACAAATCGAGTCACATTTATGGGATTATTTTCATCATATAGGATGTAAAAACGATGTATGATGATAGCTGAGTTTTAACCAAAACATATCCTGTGTGGTGTCACTTTAAATGTCCCTGGCCATTTTTTGCTTTAGACAAAAATATAGTTTTACTTTTCTTTCTTCGGAAATTGCGTTGTTCTAGTTATACACACACAGACACGATTTTTATTTATTTTATTAAATAAGTAATTAATAAAATAAAATAAAATAATAAAATAAATAAAAGAGAGAGAAGGCTTTGGAATCCCCAGATGTAAAAATAGAAAACGTATCTGATGAATTAATACATTGAAGGTCCTGACAGTCACTGACTGAAACTGATGTTTCTGGCTGTTTTCACTTTATAGTGGCATCCTTAATGTGAGACTTGACGTGTATTTAGGAGCTTACTTTATCGAATGCCTTCAGTAGACAGGAGCAATCTTTTGGTTCTGGTTTCCAGTGACCACGAAAAAATGCTCTTATAGAAAACTATTTTCTTTTTCTTTGATGCAATATGTAGGATATGTATCTTTATGATATCATGATGTTTACATCCTGGCTTTATCTAATGTCTTGTATGTATTTTTATGCATCTTGAACATCTTATTAAAATGATTGTTCAAAAATAATGATTCATTCACTAAGTTCACATCAAGGGTTGGTGACTGATATGTTGTAGTGATGGGAGGCCCGGCTCTTTTCAAAGATTCGGCTCTTTTGGCTCGGCTCCCTGAGAAGAGCCGGCTCTTACGACTCCCAA

The utg7180000058066 sequence encodes an MHC IIA molecule of the non-classical DB category, which does not have the two asparagines at positions -4 and +3 relative to the second cysteine in the α1 domain which are typical for peptide binding function in classical MHC class II. The encoded sequence is also incomplete and lacks a predicted leader sequence, possibly because the unitig is too short. Here the encoded sequence together with the relevant part of the unitig is shown:

10 20 30 40 50 60

GGAATCGTTTTTCACTAATTAGATCCGTACTTTGCAACAACTTACCAGGGACAGTGCGGG

70 80 90 100 110 120

CCAATCCATGCCATCTACATCATTCCACTCAACTACAAGTTATCTTCCTGAGAACATGTA

130 140 150 160 170 180

GAATAGCCAGATGGTTCTCTGTTGGATCATCAAATCTTCTCAACTCACTTCTTCCATCTC

190 200 210 220 230 240

AGCTCCCTACCACGAGTTACACTACATCTACGGCTGTTATGACTCTGGAGACGTGCGAGT

A P Y H E L H Y I Y G C Y D S G D V R V

250 260 270 280 290 300

GGATGTGCTCATTGATGGGGACCAAGTCATGTACGCAGATTTTAATAAGCAGACGGCAGT

D V L I D G D Q V M Y A D F N K Q T A V

310 320 330 340 350 360

ATGGACGATGCCTCACATTCCCCCTGTGAAAGACTGGAACGTCGCTTACGACATCGCCAA

W T M P H I P P V K D W N V A Y D I A K

370 380 390 400 410 420

GGCTGCCATCACACACTGCCACAGTGTCCTGAACAAGGCCAGACGGGCTGAACCCGGTGT

A A I T H C H S V L N K A R R A E P G V

430 440 450 460 470 480

TCCCACTAGACAAGGTACTTGTCCTGCTGTGTCATTGTTGGTTTAGCATGAATTTATCTT

P T R Q

490 500 510 520 530 540

GGATAGAAGGAGAGAGTGCAAGAGGCAATCTGAGGAATTTGTGGTTAGTGTTAACACAAC

550 560 570 580 590 600

AACACACACACACACAGACATGTTAGATTAGATTAGATTAGATTAGATTAGATTAGATTA

610 620 630 640 650 660

TGCCATGGCCTGGCAAAATACTAAATTCCCATTGGCTCGATGCTGCCCATTATTTTTGTG

670 680 690 700 710 720

TAATGTACATAATACATGCTGTGTTCTATTCTGCTCTGTTCTATTCTATTTGTGACCCAG

E

730 740 750 760 770 780

AGGCTCCAGAAATCTCCATCTACAGCAGGTACAAGGCAGAGCACGGTGTGGACAACACTC

A P E I S I Y S R Y K A E H G V D N T L

790 800 810 820 830 840

TCTTCTGCTTGGTCAGTCACTTCTATCCCCCCAGCATCAACGTCACATGGACCAAGAACA

F C L V S H F Y P P S I N V T W T K N S

850 860 870 880 890 900

GCCAGGTGGTCACAGAGGGGGCGTCCCACCTGCGTTACCACAGTAACAAAGACGGAACGT

Q V V T E G A S H L R Y H S N K D G T F

910 920 930 940 950 960

TCCACAGGATTTCCAGACTGGATTTCACCGCGGGGAAGGGAGACGTCTACTACTGTAGTG

H R I S R L D F T A G K G D V Y Y C S V

970 980 990 1000 1010 1020

TGGAGCACCAGGCTCTGGAGAGCCCTTTAAACAGGACCTGGGGTGAGAGAGAGAGACTGT

E H Q A L E S P L N R T W

1030 1040 1050 1060 1070 1080

CTGTGCCAGGACTGTCAGTGCTCTTTGCTGTATCGTGTTGTTATTGGTTTTTTTTGTCAT

(stretch not shown)

1750 1760 1770 1780 1790 1800

GGCCTGAACACGTGCCTTCTCTTTCTGCAAAACTAAACTAAACTAAACTTTATGATTAAG

1810 1820 1830 1840 1850 1860

CTACTTGTTTGCTGTGTCCCACAGAGCTGGAGGTGAGGGAGACCAGCGTCAGTCCTGGAC

E L E V R E T S V S P G L

1870 1880 1890 1900 1910 1920

TTGTCTTCTTTGGAGTCAGTCTGGTTTTGGGGCTGGTGGGACTCGGGACCGGAACGTTCT

V F F G V S L V L G L V G L G T G T F F

1930 1940 1950 1960 1970 1980

TCTTCATCAAACAACCAGCAGCAGTGATCTGACTGGGAAGAGTCATCATGGACAACCGGG

F I K Q P A A V I *

1990 2000 2010 2020 2030 2040

AGTCTTGTCTTTTCAGAGATTTCTGGAGTAATTCAGCTATACTATTACTAGTATTCAGCT

***Polymixia japonica MHC IIB***

>utg7180001073264 length=10650 num_frags=1280 Astat=383.00

(the complementary sequence is shown)

GATTTTGTACATTTGTTCTCTCTAAATGTCTCCGTTAGCTGATTGGTGGTTGTGTGGTGTCCCTGGGGTCCCAGAGGTCACAGGGGTCAGAGGTCAGAGGTCACAGTCCAAAACACTTTAAAATAAATTAAATTAAAATGTCTTTTTTCTTTTTCTTATTATTCTTACCTGTAGTTATGTTAATGTGCCTGTGTCTGACAGGTGTGTGTGTGTGTGTGTGTGTGTATTTGTGTGTGTGTGTTTGTTTTTGTTTGTGTGTGTGTGTGTGTGTGTGTGTGTGTGTGTGTGTGTGTGTGTGTGTGTGTGTGTGTGTGGGTGTGAGTGTGTGTGTGTGTGTGTGTGTGTGTGTGTACTAGAGCAAAACAGATAGAACCAAGAGGAGAGAGGGGAGAGAGAATAAGAGTGAGTCCATGTGTTTGTGGTTCTGCGCGCCAGAGGATTAATATGTCTTGTTTGTGTGTATTGGTCACATATTACTATGAGGAAGTGCATCGCCAACCACAATAATGAAGTGAATTACATGAGAAATGCTTGGTATGGTTGGCCTCCGCTGGGCGAGGGGTCTGGGGGTCTGGGGGTCTTCCACATAGGGAAGGGGGAAGCAGATGCACGCTGCACCGCAGGGAGACAAAGTGAAAAGCTAGCTTGTTCAACACCATCTGTACTTTATTTATAGCCATCCTGGCACAGACCCCCAGATATTACATTCAGTGGCTTTGTATAAACAAAATGATGAAATAAATCATCTTCCAGACGCAAAGCTATAAAGGTGTCTGTCAGTGAATAAGAACAAGCACAGTCTACATGCAGGGCGATGATATGTAGTCCTGTCTGAAGAGGATTGTGGGAGTTGTATGAAAATTAGCACCTGATGTGGGAAGATTCCCAGTTAAAAGTTACATTTTAACTTAATTAATTTTAAATAATAGAACTTTTAATTCCTTTTATTAATGAGATTCTTTGCATCACAGTTTAGGATGTGACTCTCTTAGACTTACACTGTAAACGTCACCAACGTCAAATCTCCATTCAATGTTGTTATGGATAAATGTTGTTATGGCGACAGAAAGCAGAGTAACTTCTGTTAAGAAATATAAGGAAGCCGGCAACTGACATTTTCAGAGAATATAAGTGATGAAGTTCTTGAAAGCAGTTATTCAGGTAATGCTGCTCTTGATTGAGCCTGTTAGTTAAACACACACAACACACACAATTATCCAGTGAACTAAGGTTCTGACAATATAAACAAAGGAATAAGATATCAATATCTTCACTGCCTGCTGTTTTTGCTAGTAGTCTGGATTAAAAGTAAGATTACAAAGTGGTTGTCAATCTCATTAACTCAGCAGGGGACAGAGCTATTAAGTTACATCATCAGGTTCCACTTTGCTGCCAGGCTGGAGGGCAAATGGACTCTATTCATCTTGCCTCAGTATGCAAACCAGCCTTCAATCTGTAGGCCTAAACATCCTACAACTTCTGGTTCCTTGGTTAATCAGATGGTTTTCTCCCACCCCAATTGCTATGCTGCTCTCAATGTCAAACAGGAGTAGACCAGAGGTACCCTACCCAATGTGGGCCACTGTTAGCACTCACTCAATATATCATGACTAAAATCTATCTTGGCTAGCTGGCGACAATATGGTTTGGTCGTCTGGTTCCTGGCAGCCCTTGCCCAGTTTGAGCAAGTGTTTTCCTGCTGGAGATGTAACGCCATGCCTATTATGGCAGCAGTAATCTGAGTGAGATCTATATCTGTGGGTAGAGTGTCTGTCTGGCTCCACAGCTGTGTTCTCTGCTGGACCAGGAGAAACCCTCAGTATCGACCTGACCACACCCCCACCCCCAGGGACAACTGAAGGTCAGTCTGGGGCAAAGGGAAAGAGAAAGAAAGACAGAAGTACAAGTGAGGAAAAAGAGAGAAGGGAGATAGAGATAGGCAGAAAAAAGACTTCAAAGCACAGTAGCCTCATCTTGTGAATCGCGAAGGCAAATAGACCTTGTTCCATTTCTCTTTGCCCTTAACAGAGCTTGTTGCTGCGAGCAGCCGTTGACTAGTGTCAACAGTAGCCTATTTTTATGATACAGAGAACACCAGATTAAAAGCCTTACAGACAAACTCCCTCAAACACTCTTCTGGTAGACTAGGCTAGGGATTATATGTAACTATCAACTACTGGGAACCAACCAAACTCTGAAAAGGAAACCACCATTCTCCCAGTATTACATACCTAATTTTACATACCGTATCTTGCAGACTGCGAGCACTGTTACAGCAAGACGACCAAGTCTTGGCCAGTCTTGAACTTTTTAAGACTTTAACTTAAATCTAAGAGCACAGAACTAGATTGTTCTTTTGAAGTCTATGAGTGCACGTGCGTGCAAGAGAGAGATAAAGAGAATGGATAAAACAGATGTGGCTTCTCTTGGAAGTAGCTATGAAAAGGCAGTTTTGGAGTGGTGGAATGAAATACCACTAGAATATTATGATTCATGCTACTTTTCAAATTTTTAGTGTCGGTTGCATTGTTGCACAGGTGGAGCTGAAGGCCTACACCTTGCAGGATTTGTATGAATCTGAATACCGCCTTATTTTTTTGTGATATTAAACTAATCTCCTGAAGGAGAAAACTGTCTTTTCACTTGCCACCCTGCAAAGCAATGTGCAGGGTCTGCTGCAGAAAGGTACCTCTGGAGTTGGTAGGGATTTAGTGTCTTGCTCAAAGACACTTCAGCAGGACAGATGTTTCTGTGACACGGGGCTGTGTGCACACATGCAATGTTCTGTGCTTAAGAGCAAACGCAGCAACAATATCAGGCAAGCACTGCAGCTTGACTCACTTCACTGTTTGCTCTAGCAATGTGGATGCAATTTTAGTCATTAAACTATCAGCAGATTGTACTCTACAGGAGAACTAAGAATGTTTTCAATTGTGATTCACCACGCAAACAGAAACGCAATAAAGGTATCAGGAACATTTCAACGAGCAGTTTGCTACCACAAGGCTAGCCTGCTAGCTGACGGACTAGCACCACCATTACCAGCAAGTTAGCAAGCTAGCCAGCTAATAGACAAGCTAATAACCATATGTGCTTAGATGCCACAAACTGAGCAAGTAAAAAAAACATTTTAATAAAATGTTTACCTTCGGTAGCAACATCCTCCTGTAGTGTTTTGGTGACCGCTGTGGAGACGTGGTATCCCAGCAGGCAGAGTAGGACCGCAGCGGTGCTGCTCCAGTCCACAACCCGCATCACTGCAGATGTGAATCAACAGAAAGGATTTAAAAGTAGCAGCTAAAATCTTTGCGTTGGATAAAAGTCTCTGATTAAAGCAGCGCACAGTCGGATCCTCCGTGAATCAGGCGATACTCCATGTTTAAGGAGCGGGCAGCCTCCGATTTATTTCTGCCTTTATTGTTTTTCTTCCGTCCCGTTTTTGCATCCAGCCCCGTTTTTGCCGCTCGGTGGGTTTTTTTCGTCCAGGGCTCAGAGCGCCGCTCCTCCGCCTCCTGCCCCGCCGCCGCAGCACCGACAGGCGGCTTGTGGAGACGAGCAGCACCGCTGACATCCATGGATCTCCGACGCCATCTGCAGGGCGCTCGACATTACTGCAGATATACTGTCACGAATTGAAACAGAGTTATGAAAACAAATCTCCAAAACAGTATCTATGAATATATAAATGTAAATACATTACAAAACGTCACTATTATATTATATGCATGCATATAAATACACTAGGTTACGTATTATACTGCATATCATTGACCATTTTGAGGCTGAGGATCACACTTACCAAAACTAGTTCAGGTTTACCATTTGATTAACAATTTAATCTTAATCTTAATTAATTTGATCACCAGATGGATCCATAACACACATCCCCTGTGATTACAATTATTTATTGGCCAGCATTTTGATGAGCTTGTTCCAATTTCACTTCGCTCTCATAATTAGCTTTTACACCTGTGGTTGAGACCAAGCCAACAAGAGAATTTCCTAATTTACACGTTTACTTTATTGAACCTGCAGCAGTCCAGGGACGTGCACAGACATTTTGAGGGGCTGTTGCTCTAACCTGCGCAGGAGAGGTGTTTAAACGCCTGCTGATATTAGCTTATACCTTTGGTTGCTAGCAAGAGTAAATATTTAATGATTTAATAGTGTTGTAAACTAACTCACATGCTAACAACCAATTGCACATATAGAACGGCTGTTTCTTGGCTATATCAAAACTGTACTTGCATTTTTGAAGTTCAACCTGGTTGATGGCCAATCATAGACTGGGATTTTTGGATGGGCACCAGGGGCGGCCAATCAGATCACCAGGGTGGTCTGCGCCCCCTTCATGTGATGCCATAATAAGGCAATGTTACGCGACATGATGCAAACGACAAGCTGCACATGTTCGGAAGCACATGTGGAGATTTATTTCCCACATTTTTGCTAAATAATTTTAGCATCAATCATTTCATTATCCTATTTTTCAACTGCACCTTTAGGATCTTTATTTTTAGTTAACAAAGGAGAAGTATTAACTTGTAAGTGAAAGAAGGTTTTAGATGGCAGTTGCACTAATCCTTCACTAACTCTAGTCGATGACATATTTGACAGCTCTTTGGTTGGGATTGAGTTTTACTGTACATCCTGTTCACTGTTCTGGCTGCCAATTAGAGACAGTAGAGATACTGGAATTATCACTATTGCCAATACACATGACCCTGTGTTACTCTCCCTTGTGCTACAATTTAATTCAAATAACTGAATGTTAGACAAATAAGATCAATAACTGGTGTCATCAAGTGGGCATCTCCCATCATAGAAAATTCATTGAACTATCACCATTACAACTATAATTGAACAAAGGCTCAACGGTTTATTCTGCAGCACTTGAGTTGTACCAATTATTGAGATTTTCTCGTTCAATTCTAGCAGTTTCACTGTTTTTCCCTGTGTGCCCTGTTAATTTCAAACTAGAGAGTGAACTAGAGAATCTCGCTCCTGCAAAACCAGTCAGACTGCTAATTATAAAACCTCCCGGTTCAATAACAATCAAAAGATTTATATACTTCATTCATATAGACCTAGGGCCCAGATTCCATCTCCTATTGGCTAATAATGAACACATGACCTAGCTATGCGATTTATTTCAGAGCTTCCTCATTCGCAAAATTATCAGATTCTTCTGTTCAAACCTAGAGTTTCTCTTCGATGTCTGACTGAGCTGAATCTGGCAGCCTTACAACATGAAGCACTCTGCCATAATCACACTGATTCTCAACATCTTCTGTGCATATTCACAGGGTAAGATTTAAAAGTACTATTTGTCTGTTCTGGTTAACATTAGGAAAGATAAAGTTTATGTTAACATGGGAACACATGCAAATGAAAGAAAAACTACCTGCTATTGCAGCACGGTTCTCTAATCTATACTCTAATTGAAACCTCTGACTATAGCAATAGACTTTGTTTTGGCTCCTATGAACACAGATGAATATAAATGTATGAAATAAAAGCATTGAAGTGTTGAGATAGGGCATGAACCTTTTGGCAATGATGAGAAACAAAGTCAGTGGTTTTAAATGCATTTCCAACTGTTTTATTTTTCAGTTCCCCATGAGACTCTCTACATGGTGGGTTGTTTTGTGAATGACTTTGAGGCCGAACTGGAGATCGATGGTGAAGAGGTGTTTTATATCGATTTCCAAAGGCAAAATGTGCAATTCACTCTGCCTGCATTTTTTGCAACAATTCCTTTGGATATTGACAGAATATTTCATAACGCTGTGAAAAACAAGAAAGCTTGTTCTGGAGTGTTGGCATTTTTAACAGTAGAAGAAAAACATCCACCAGAAGTAAAAGGTAGGGCAGAGGTATTGTGTTAACCCATACATTTGCTTCTGAAATGACATGATTGTTGGTCTGTGACTGTATTCATCATCATCTGATCCCCCCCAAGATCCACCTGCGAGCGCTATCTACACCTCAGAAGATGTTGAGCTGGGAGTGGAAAATAGTCTCATCTGCTTTATAAATCATTTCTACCCTCCTTCGATCAAAGTCAACTGGACCAAGAATGGCCAACTGGTGACAGAAAAGGCATCTCTCAGTCGGTACTATCCAAACAGCGATGGGACCTTCCACCAGTTGTCCACGCTGACATTCACCCCAAGGGAGGGAGACATCTACAGCTGCAGGGTGGACCACTTAGCCCTAGAGGAGCCTATAACTAGAACCTGGGGTGATTTCTTGTTTTTTAGAGTAAATCAAATATAAAAAGAGTTACTCTTTCACCCTTTTATTCTGAATGTGGTGATTCATATGTTCACTTCCCATTCCTTCACAGAGCCTAACCTGAGTACTCCCAGCCCCAGCCATGGACTTGATGTATTCTGTGGCGTGGGCCTCACGCTGGGGCTACTAGGGGTCGCAGGTGGAACGTTTTTCATTGTCAAGGGATGGACAATGGACACATAATAATTCCTGTCTGGAGGAGATCTCTTGTACATATGGTGTCATAAAGATGGAATGAGATACAGTAACATTTTGTATATTCATTTCTGTTTGTAACGGTGGCAAGCTTGAGGCATGAGACATAACGTAATGACATTGCCAACATAGAAAACACCTTTATAAATTTGTTCTTTCAGTTCCTTGGCTGTGATGAACATGTGAGATTTGTTCTTTAACTGCATAGACATGTCTTTCATTAATTTAGAGAACTTAATTTTCTATCACAATCTCCTCCTGTGATGATTAATGATCAGGCTGTTGAGGTTTGACCTTCATGTGGATGCTGTGTGTGGAAAAGTACTTTTATCATAAGCTTCAATGTTTTAATGTTGACAATACCTTCATGAAAACGTTTTATTCCTGTTTTATTGAATCAGTCCTCGCGTTCTCCTTTGTCAGCTGGTCCGGGTCACTTACCCTCAAAAATAGAAACAGATATCATCAAAGGTATCATCAAAGTGTGCAGCAAAATAGCAGGCATGACCCTGAATGACCTTCCTCCTCCATCTGTACAAAGTCAGGACTTTGAATAAGGTCCAGTCAGTCCTGGTTGATCCTAGTCAGTCCTGGTTGATCCTAGTCACCCCCTGTCCCTGTATGGTCGCAGATACAGTCTGCCAAGATGCAGGACCAATAGACTTAAGAACTCATTTGTCCCTGCTGCCATTGGCCTTTTAAATAATTGAATGTGATTTTTATATTGTATTTATTGTCTATTACTGTTTGTGTGATGGTTATGTGGTTACTGCTGGCTGTACAACAAATTGCCCCTTGGGGATAATAAAGATATCCTTGACCATGACCCATGAACCTTCAACATTTTTGAAACAGATGACTTAACATTAAATAACATTATCTTGGTCACTGTGCTTAGTGCATGCATGTATTATAAGTATGATTTTCGGCCTAATAAAATAAAACCTAGTGAATGCCATCACTACAGGTTAACATGGTCAACTAAAGACTGGAGAAGTAGTCAACACAACACAATGACAGCAAGCACAAAAACTAAACATGATTGAGAGCTGCACCTTAGATATGGTATTGTGTGCCAGTCAACTATTTAACTTCCTCATTGCTCCCTAGGACAAATGCTCGCACAGATGCTTCCTGTTTTTGTATCATTGACACACTAGCATGTTAGCATGTCTGCTCTGAGCTCCTCTTCTATCCTCCTGTTGCTATTACTATCATTATCTGGAGTGGGTGAGTAAACATCTTCTGGTTTAACACTAATATTTGAGAGTCTGTCAATGTCTATTGTACTTACCATTAAAATGAATATGCGGACATCAACAAACAATCCTAATAGCCACTTGCTACATTACAAAGTGTTACATGAGGTTATTAGACACCTGCAGCGTTCGCCTTAGTAGCTCAAACAAGCATCTGTTCACAGATTTTTCTTTTTCATTCTTTTGTGAAGTTTTGTTAAAGAGCAAACAAAAATATCCACATTAACTACTGTTTGTGAACTTTATCTTTCAGATTCAGGTCTCTTCGCTCATGGTATGATGCGCTGTCAGTTTAGTTCCAGAGATGGTCACGACGCTGTGTACATAGAGCAGTACTACTTCAACAAGATGCTTTTAGGACAATACAACAGCACTTTAGGGAATTTCACTGCCTACACAGAGAAAGGAAAAGACATTGCAGATGCCCTCAACAAAAATCCTTATTTTTTGGAACAGGAGAAAAAGAATATGCAGCACAATTGCAGACCCTTCATCTCGGAGATATATACAAATATATTGGACAAGACAGGTGATTTCAGGCAATTGCCTTGGATGCAACTCCAATCAGTAGATGCATAACTCCATTATTAATAGATCACCTTTTTTTAATTTAATGTACAAAATATTACAATTACATCCCATTGCAAATACATAACTTTTAGGATGTTGTTTAGTGGTTTGTGTCTTTTGGTATTTTTCTCCAGTTAAGCCCTACGTCAAACTGAGGTCCGTTGAGCCAGACAGCAGCAGGCATCCTGCTATGCTTGTTTGCAGTGTGTACAACTTCTATCCCAAAGCAATCACAGTGACATGGCTGAGGAACGGGGAAGAAGTAACATCAAATGTGACTTCCACTGAGGAGCTGCCCAGTGGGAACTGGCTCTATCAGATCCACTCCCACTGGGAGTATACACCCACAGCTGGAGAGAAAATCACTTGTATGGTGGAGCACGCCAGCCTCGTGGAGCCCATGATCAAGGACTGGGGTAGGAGTGCAGCAGCATACAGGCTATGAGTTTTGACGAAGGATGTAAAATAAGATAGGATTGATAAGATCAACTTTATTGCCTCTATGGGGAAATTCTTCTTGGATTCCATGCAACTGTTGTTATCAGTAAAAAGCATTAACATCAGTACACAGCCTATAAACAGTATACATATGTACATTGCTTTGTATACATAGAAAATGTATTCATTTAAATGGCCTAGATTAAGTCAAGTAAAGTAATGTATAAGGTATACCAGCTTAGAGCAGAGGTTTCTGGGATGCAACAGCGTTAGGTTGAGTCGTAACAGTGAAAGAGACATCAATCCTAAGCATGTTATAGGTATTATGAAATGTTTCTGATAACTGTCCTCTTTCAGACCCTATGCTTGAATCAGAGATGAATAAGATTGCCGTTGGGACAGCGGGGCTGCTCCTAGGGCTGGTCTTTGTAGCTGCTGGGCTGATTTACTACAAGAGGAAATCTACTGGTGAGAGACTAGATGTCAAATCAAGACTTCACAAAAAATAAATAAATAAATAACTATGAAATTATATCCAACTCCAACGATGACAGCTGAGGTATAGCCCATGTGTGTAGAGTTGGCTCTTTTCAAAGACATATAATTAATAATTATAATATTTACATAATTCATATATTGCATATATTTATATTTATAATAGCCTACATAATTAATAAATAACTATTTCACAGCTGGGCGGATATTAGTACCAACAAGTTAAGGTAGGCCTTTACTTTTTTTTTTTTTTTTGTATGATTTATTTATGAGATTTTGAGCATAACTGATATACAATAAGGCCTTTACTTTTAACAATGATGTACATATGTCAACTTTGCCTGTTGCTTGAGGTATGAGGGGTTTTTATCTGGTGTCCACAGGGTTTCAAGAGACGTCCGAAGTCACAATAGAGGATGGCAGGAGAATGTTATGGAGCGTTCTGTGTGCAGATATCTTGAAACACATCTCATTCTCCTGGCTTTAGCAGTTTACAATACAGAAATGTAGCCTAAAATGTATTTATTTGGCATCGGCCTTCATCAATTTTGCTATTTGATTAATGGATAAAAGATCAAATCTTAACTTATTTACTTATTATACTTATTATAACTTATTTATAACCTTATAAAATCTTTAGTGTCTGTCACTGATCTGGAGAAAGTTGTTCATGTGTTTATTTCTTCACGCCTTGATTATATGAACTCTTTGTTCACCTGTCTCAACCAGAAGTCAGTTATAGCCTACAAAATGCTTCTGCTTGGCTCTTAATGTGCACCAAATAATCTGACCATATTACACCTATCCTGCTTCTTTAAACTGGCTACCTGTCACCTTCAGAATAAGCTATTCAAATTGTACTAATCACGTTTATGGCCAGATTTGGTTTAACACCAATATAAACTATCTCAGAAACTATCTCAGAATTGTGTCCACGGTGGCGGAGTGCTGCCTCACAGCACGAAGGTCCTGGGTTCGATTCCCACCTGGGGCTGGTTGGCCCCGTGCTCAGGTGGGTTATCTCCCTGCCTTTCTGTGTGGAGTTTGCATGTTCTCCCCGTGCTCACATGGGTCTCCTCCAATATGGACCCCAACTAAAAACATGCAGAACATTACCCTGTCCGGTCAACTGACCAAGACTGGACAATACTGGAACTGGTCCCCGGGCACCGACAGACAAGGCTGCCCACTGCTCCTGGCTGCCCTCCGGAGGAAGGACAGAACAGGATGGGAGAAATGCAGAGGACAGATTTCGCGGCCACCTGTGTGTGTGCATGTCTGGCTGCCCTTGCATGTCATGTACACATGGTGTGTTGTGACACAATAAAGACTCACTAGACTCCTGTTAATTCTTAATCGGGAGAAGCTGGAAGGTTCCCGCTGGGCCACAAGGACC

The utg7180001073264 sequence appears to encode an MHC IIB molecule of the non-classical DB category. The encoded molecule does not have the two amino acids motif, HN, at position +2+3 relative to the second cysteine in the β1 domain, which are typically involved in peptide binding by classical MHC IIB molecules. Below the encoded sequence together with relevant parts of the unitig sequence are shown:

7570 7580 7590 7600 7610 7620

TTGTGTGCCAGTCAACTATTTAACTTCCTCATTGCTCCCTAGGACAAATGCTCGCACAGA

7630 7640 7650 7660 7670 7680

TGCTTCCTGTTTTTGTATCATTGACACACTAGCATGTTAGCATGTCTGCTCTGAGCTCCT

M S A L S S S

7690 7700 7710 7720 7730 7740

CTTCTATCCTCCTGTTGCTATTACTATCATTATCTGGAGTGGGTGAGTAAACATCTTCTG

S I L L L L L L S L S G V

7750 7760 7770 7780 7790 7800

GTTTAACACTAATATTTGAGAGTCTGTCAATGTCTATTGTACTTACCATTAAAATGAATA

7810 7820 7830 7840 7850 7860

TGCGGACATCAACAAACAATCCTAATAGCCACTTGCTACATTACAAAGTGTTACATGAGG

7870 7880 7890 7900 7910 7920

TTATTAGACACCTGCAGCGTTCGCCTTAGTAGCTCAAACAAGCATCTGTTCACAGATTTT

7930 7940 7950 7960 7970 7980

TCTTTTTCATTCTTTTGTGAAGTTTTGTTAAAGAGCAAACAAAAATATCCACATTAACTA

7990 8000 8010 8020 8030 8040

CTGTTTGTGAACTTTATCTTTCAGATTCAGGTCTCTTCGCTCATGGTATGATGCGCTGTC

S G L F A H G M M R C Q

8050 8060 8070 8080 8090 8100

AGTTTAGTTCCAGAGATGGTCACGACGCTGTGTACATAGAGCAGTACTACTTCAACAAGA

F S S R D G H D A V Y I E Q Y Y F N K M

8110 8120 8130 8140 8150 8160

TGCTTTTAGGACAATACAACAGCACTTTAGGGAATTTCACTGCCTACACAGAGAAAGGAA

L L G Q Y N S T L G N F T A Y T E K G K

8170 8180 8190 8200 8210 8220

AAGACATTGCAGATGCCCTCAACAAAAATCCTTATTTTTTGGAACAGGAGAAAAAGAATA

D I A D A L N K N P Y F L E Q E K K N M

8230 8240 8250 8260 8270 8280

TGCAGCACAATTGCAGACCCTTCATCTCGGAGATATATACAAATATATTGGACAAGACAG

Q H N C R P F I S E I Y T N I L D K T

8290 8300 8310 8320 8330 8340

GTGATTTCAGGCAATTGCCTTGGATGCAACTCCAATCAGTAGATGCATAACTCCATTATT

8350 8360 8370 8380 8390 8400

AATAGATCACCTTTTTTTAATTTAATGTACAAAATATTACAATTACATCCCATTGCAAAT

8410 8420 8430 8440 8450 8460

ACATAACTTTTAGGATGTTGTTTAGTGGTTTGTGTCTTTTGGTATTTTTCTCCAGTTAAG

V K

8470 8480 8490 8500 8510 8520

CCCTACGTCAAACTGAGGTCCGTTGAGCCAGACAGCAGCAGGCATCCTGCTATGCTTGTT

P Y V K L R S V E P D S S R H P A M L V

8530 8540 8550 8560 8570 8580

TGCAGTGTGTACAACTTCTATCCCAAAGCAATCACAGTGACATGGCTGAGGAACGGGGAA

C S V Y N F Y P K A I T V T W L R N G E

8590 8600 8610 8620 8630 8640

GAAGTAACATCAAATGTGACTTCCACTGAGGAGCTGCCCAGTGGGAACTGGCTCTATCAG

E V T S N V T S T E E L P S G N W L Y Q

8650 8660 8670 8680 8690 8700

ATCCACTCCCACTGGGAGTATACACCCACAGCTGGAGAGAAAATCACTTGTATGGTGGAG

I H S H W E Y T P T A G E K I T C M V E

8710 8720 8730 8740 8750 8760

CACGCCAGCCTCGTGGAGCCCATGATCAAGGACTGGGGTAGGAGTGCAGCAGCATACAGG

H A S L V E P M I K D W

8770 8780 8790 8800 8810 8820

CTATGAGTTTTGACGAAGGATGTAAAATAAGATAGGATTGATAAGATCAACTTTATTGCC

8830 8840 8850 8860 8870 8880

TCTATGGGGAAATTCTTCTTGGATTCCATGCAACTGTTGTTATCAGTAAAAAGCATTAAC

8890 8900 8910 8920 8930 8940

ATCAGTACACAGCCTATAAACAGTATACATATGTACATTGCTTTGTATACATAGAAAATG

8950 8960 8970 8980 8990 9000

TATTCATTTAAATGGCCTAGATTAAGTCAAGTAAAGTAATGTATAAGGTATACCAGCTTA

9010 9020 9030 9040 9050 9060

GAGCAGAGGTTTCTGGGATGCAACAGCGTTAGGTTGAGTCGTAACAGTGAAAGAGACATC

9070 9080 9090 9100 9110 9120

AATCCTAAGCATGTTATAGGTATTATGAAATGTTTCTGATAACTGTCCTCTTTCAGACCC

D P

9130 9140 9150 9160 9170 9180

TATGCTTGAATCAGAGATGAATAAGATTGCCGTTGGGACAGCGGGGCTGCTCCTAGGGCT

M L E S E M N K I A V G T A G L L L G L

9190 9200 9210 9220 9230 9240

GGTCTTTGTAGCTGCTGGGCTGATTTACTACAAGAGGAAATCTACTGGTGAGAGACTAGA

V F V A A G L I Y Y K R K S T

9250 9260 9270 9280 9290 9300

TGTCAAATCAAGACTTCACAAAAAATAAATAAATAAATAACTATGAAATTATATCCAACT

9310 9320 9330 9340 9350 9360

CCAACGATGACAGCTGAGGTATAGCCCATGTGTGTAGAGTTGGCTCTTTTCAAAGACATA

9370 9380 9390 9400 9410 9420

TAATTAATAATTATAATATTTACATAATTCATATATTGCATATATTTATATTTATAATAG

9430 9440 9450 9460 9470 9480

CCTACATAATTAATAAATAACTATTTCACAGCTGGGCGGATATTAGTACCAACAAGTTAA

9490 9500 9510 9520 9530 9540

GGTAGGCCTTTACTTTTTTTTTTTTTTTTGTATGATTTATTTATGAGATTTTGAGCATAA

9550 9560 9570 9580 9590 9600

CTGATATACAATAAGGCCTTTACTTTTAACAATGATGTACATATGTCAACTTTGCCTGTT

9610 9620 9630 9640 9650 9660

GCTTGAGGTATGAGGGGTTTTTATCTGGTGTCCACAGGGTTTCAAGAGACGTCCGAAGTC

G F Q E T S E V

9670 9680 9690 9700 9710 9720

ACAATAGAGGATGGCAGGAGAATGTTATGGAGCGTTCTGTGTGCAGATATCTTGAAACAC

T I E D G R R M L W S V L C A D I L K H

9730 9740 9750 9760 9770 9780

ATCTCATTCTCCTGGCTTTAGCAGTTTACAATACAGAAATGTAGCCTAAAATGTATTTAT

I S F S W L *

9790 9800 9810 9820 9830 9840

TTGGCATCGGCCTTCATCAATTTTGCTATTTGATTAATGGATAAAAGATCAAATCTTAAC

***Polymixia japonica CD4*** *(CD4-1)*

>utg7180001882239 length=21684 num_frags=2516 Astat=867.00

ATAGGCTTTTGAATGTTCATGATGAAAATAAAAGTCAAATGTACTGATGTACGAACTTGTAGCTTTTGTTTTGAATGCATCTGTCAGCCAGAACGCAGAAGAAACCTGCAACATGCTATGGTTGAGTTTATGAAGCATATCAATATGATTTATGGATTATATCATTATATACAGTATACATTATATTCAGTATTGTAAAGTAATTATCTGTTTTGTTGCATCTTCAAATACATTAAATAGATTTTTCTGTATTTCCATTGTTTTTCCTCTTTCATTTCTGTAGGGTTTAAATGAAAATATTTGACGAATGTACAGAAATCGGATCTGATTTTTTTCATTGAATAAACGATAAGGGCATCAGTCATTTCTGAAGATTATTGCATATCCCCATAATGACTTAATATACCTATCCACCATAATATCAAACGTTCTATCAGTAGATTACAGAGACCCTCCAGAACTGGTGCAGTACAATGCAGGCAGGTGTTGTTAACCGTGCAGCACATCATACACTGAACGACACACACCTGTGAGCCACTCACACATGACCACCAATGACCCATATTCAGCTATACTAGAAAACAGTAAAAACTAATTTATGTAGAGTTAGAGTTAGAGTTTCTTCTCTTTTCTGATGTTGGCACACATCAATCATCAAATACAGCTTTAACACGAGTTCCATGTGGTTTACTTACTTCTGCTGTTTTGTTGCTTATCTGTGTGTGTGTGTGTGCTGTGTGTGTGTGTGCGTGTGTGTGTGTATGTGTGTGTGTCTAAAAGTGTGTGCACTTTTTGTGTTTGTGAGATTGTGCTCCTGTGTGATTTTTGTATGTTGTATGTGCCACTATGTGTGTACTGCGCCGTATACCATATAAAGGATGGGGTCATAGCGCAGGCTGGCTTCCTGTCTGTGCGTCTGTGCGTCTCACTGACTACAACACACACAGCTTGTGAAATGTCAGACCGAACTCATCGCTCACTGAGCACATCACACAGGTGAGTACTCAAAACCATGTATTCTCAATGGTGTACTTGCATGCAGATTTCCAAGGTCACCTCTTATAAAGGGAAGATATCTTAGCATTCTCGATGTCACGTACCGCAGTATTTGTCGACAATCTTCTACTGCAACAGTGTAGAAATAATGACATGTCTCAATGACACTGACGAAGATCAAGTAAAGCGACATAGAGGGGGCATTTCTGTAGTATTCCTATTTGGTCTTGGTGACAAGTCTGAGACTACATTTTGAAAGTGTTGTCTTGTCTTAGACCCTAGCTTAATATGTCTCGGTCTCAATCTCGTCTCATCTGTTATTTATTAGGTGTCATAATGCTCCCTGACACCAACACAATGGATCTCAAGCTGTTGACAGGGTGGAATCACTCCATTGTTCTGCAATATTGTGCTTGATAAATAATATACGTTTTGGAACACTATTGTTTTGTGATTGGTCTGAAAAAGTAAAAAAAAAAAAGCAAAAGTCAGAATTTCTTGGCTTGGCCCCGTCTTGACTCAGACTTGACTAGGGCCCGATCTTGGTTTTGTGTTGAGCTGGTCCAGACGAGGGCCGTGAGAGGGAATTAAAGATTTCACAAACAGTACCAAAACAGAAGTTCTTAAAATAGATCTCTCTGTTACCAGGTTTGATACAGAGCAAGTGGAAAGATCAAAACTTGTGCAATAACTGAATAACTGTGGATTTCGAAATAGTCAGGGGCACGGACGGGGTGCTAAATATTTGCTCATTTTACACAACTACAAAAGTATAGAGGAGCAAATCCCTGAAAATATGAGCATATTTTAGGTCTAGAAGTTTCCTCCTCCATGTTATTCCTGTGAAATGTTGGTGTCTATGGTTCAGCACAGCCTGGTGCACAGCAAGCTGCTCTCCCATGGTTATAACATGTTGTGGTTGAATTCTAGTCTGCTAAACTGGGACGTGATAGCAAAAACATTTGCGTATCAATATCAACATTACTGATTACTTACAAAAGTACATGAGATTTTTTTAAAGAATTAAATTACCCAGATAAGATGGTCTGTAGCAACTTATAATTGTATGGAATTGAGAGTGTAGTCGGTAATGTGTAACCTCACACTTTTGGTTCACAATTTCAAATCTTTTGCTGTTCAAAGTTTTTCCAGCAGATATACATCCATCCGTCCATCTATCTATCCATCCATCCATCCATCCATCCATCCATCTATCCATCCATCCATCCATCCATCCATTTGTCCATACCACTTGTCCAGTCAGGGTCATGGTTTTTAGGAAGCGTAACTTAGTAATAATGATTAACGCTTTGTTTTTGTTGTCATGTAATATGTGCCAAAGTTTGTATTACCACACAAATTGAATAAGACCATAACGGTATAATATTTACAGACTCATGATATTGGTCTACATCTGCTGCAGGTATCAACTATGGATATACTGTACGAAAGCTCGCAATCTGCAGACAAAGAGCGCATAGCATATAAAGTATGTTCAGCAACAATCCAAGTATTGTATTTCTGTCTAAAGTGTACAGACCCATAACCGATACATTCACATTCACGGGCTTTTTCTTTATTATAGATTCAATAATTAAATAAATGGATCAAAGTTGTACTTATTTTCTAAGCGTATAAGTGTGAAGAAAACCTACAGATTCACAATTACTTGCAAGTCACTTTTTGTAACATATCGCCAAGTAGTTTTATCTGGTCAGTCAGTCAGATGAAAGTAAACACATTAAAGATTAGGCATACAGACGCTCTAATATTAACTATTTAGTTGCTAACTATAATCTAGCAATAGACATTGATGCTAACTAACATTGATGCCCCAAATGTACTTTGAACTGAGTCTGCATTATAGTAACCTAACCAAATATTTTACACATGTAGTAGTAAACAAGCAAATCATGTTGTTGTTTTTTTTAAATCAATTTATAAACACAGCAAGTAGAAATCATTGTGTAAATCACAGCCGTTTGTGAGGCAGTAAATTACGTAAGTGAGAGAGATTATCTTTAAAATGGTCACGTTTCATGTTAGCGGTGGTCCGCCACTCTGTAGAGAAAATCATTTAACATTAGCCTTCATTACACATTGTTACGCTCTAAATAGCGAAGACATCATTTCTTGTGCCTGTACACCTAACAATGTTGATGCTATAATAGCAATATATTTAGTATTCATCATTTTGATTCAATTCTATTCTATTAACAGAGATATACAATATTTTAAACAATGTATAAAGTCATTTAATGTAACATTTTTGTAATGTAACAGTTTTCATCCATGTAGTTCAGTGGCCAGCATCAAGTCTTTACTTTGGCCTACTTTGCATCTGGGGTGTCATGTTTCCTCCTAAAGGCTAACCTAGCTATCACAGGTGTTTCCTCCCCTCTGGTGTCTTCTAAACATATTTTAAACCTGGCAAAACTGCAGTGAGAGTACATTCATATGAGACACTAGGATTCCCTTTTTAATGGTATCATTACACACACATAAACTGATACACACACACACACACACACACACACCATTCATTATTCAAAGAGAAACTGAGAGACTTGAACCTACATTTGTCCTTCCCTCTTGTGTTTCCATTGGGGCTGTATTTTACTCTGGCAAACATACGTCATCTTCTGATAAAAGCTTCACGCTTCAGCACAACTGTAACTGTATATCTTCACATGAAATGTGCCCTATCCCCCATCAGAAAGCTTTCCTATGATAAACTATAAATAAACCGTAAACTATAAATACCTATGGTTTGATTTTCAGCATAGAGAGAACAAGTGAGTCAAATGGCATTTCTCTGTTGTTCTTAGTTTGGTGGAGAAACTCTGCAAACACAAACACTTCATTACTTACTGGGGAGGCTCAGTCACATCTTATTTCCTGCAATTATGACGGTCTTTTACATATTAAAGAATATATCTTTATTGTCCACCAGGGTGGAAAATTGTCTTTGGCAATTTTCCACCCTGGTGGACAATACAGATTTATTCTTTATTGTTTATTGTATATTATAATCTTGGCTAACTGTAACAAAATACAAGAATCGGGCTAATTAGAGCACAGCATAGCAAAGCACACAAGCTCTGGCTAAACCTATTAGATTGAAGATTTATTATTTTCTGGGCATAAACTGAAGTCTCTTAAATGTTATATTGGCGTTGTAATTTCTAATGCACTGGAGGTTTGCAGACAGGCAAGCAGGTATATGAAAATCCCCAGTTCCTTTCGTAAACTATCTGGAATCATAATTACAGGCTCTAAATCCTCTGTATTTCTCACCTGGGTGAAGAATGTTCTTGTTTGTGCAAACTAGATTATGAATGATATATTCGATAGTCAGGGACTGTGGTGCAATGAGGTGTATAATATCCCTGGAACTTCCTACTTCTTTTATTTGAGGCTTCGCTCAGATATGAAAACAGTTATTTGGCACAGACGTCTTGATATGTGCTGCGTTGTCAACATCCTTTTGAATACCCCTAAAACACTGGTGTCTAGTTTTTATAGTAAAGCGATCAACGCGTCTTCCTGTGATATCACTATGGAACCAAGAGCTTCTGATGAGACAGCCAGTGTTGAAATGGATGGATATATGGAAAATATTTTATAATATCTATCCCACCAACTGATAAATGATGAATGTATGCAAAGGTATTACCTTCATTTGACAGTTTGAAGTTGTGTATGTTTTGCTCATGTGACGCTCTAGGCTCTAGGTTTTGGGGTTTGCCCTTTGATAGCAGATTTATGGGCATTTGTGAAAGGTTTGTATGACAAATCTTTTTGCCATTATGTCCCAAGTTGTTTCTTTTAGCTGATTTTAGCTGAATTTGTCTTTGACTACTATGAATAAGCGACTTTGCTTGTGTGCTTTAACAGCAGGAAAGAAAGCAATTTTGAAAAAAAATCTTCATTGTTATTGCGCAATGTTTTACTCAGATATTCTGATATTGGAACATTCGGCTGTAAAATGACATGAAAGTACGCTAAAAACATTTCAGCTTGGGCCTCAACCATCACGCCTGTCAAGTCCTTGACTGTTTCTTCACGATCTTAAGACTATGAACCAGAAGTCATTATCTCTGAATATCCATACAGTACATGTGTGTTTGTGTTTTTGTATGTGTGTGTGTGTATGTATGCATGTGCAGGCTATACGTGCACATACACATAGATGTGTGGATTCGTGCATTGGTATACGTTAGTGTACGCGTGTGTTTGTACGCATGTGTATCAGGGTTAGGTGGTGGTTCGCTTTCAATTTAGAATTTCCCCCAGTGGGAGTAATAAAGTACATCAAATCAAAGTTTACAAAAAATAAGGAATAATTGAGAATTAATGCTTTATCATTAGCGAATAAGATGCAATTTCTGTTTAGATTTTGAGTTGACTGGGTTAAGGGGTGAATTGAATCCAACATCCACATGATATTACATAATTATGTATTGTTTGTGTGTATGTGTACTGAATAGGTGCACATGTACAGCATATGTATTTATTATAAATCATTTATATATGTATTGGTTCATGTGTATACGCATACCATACATGTATGCCATACATATAATAGCCCCTTTTTTCTCTCTTTATGTTTACTGTTTAATGTTAACCCCGTTTTGAACATGTTGCGATACTCCCTCACGACTGCTGTTTGTAAATTAGTTCTACCCATATAAATAAAAAACATTATTCACAAAAAAAGTAAAGCACATTCGCACAAAACCACTGATAACAGACGATGAAGGGAGCGTAAAATGTAGGAGAGACAGAAAAGGTCAAAGGTTCTGTGTTCATCTACCATTAAACAACTGTGAGGTTTCGACGGGCCTTGAATGAAGTTGCCCTCGTGATGTCACACACAGCGTTTTCACAATCACAATAGTCAGAATGTTGTTTGTCTGTGAGTCTCACCCTCCTTTTCCACTGACTATTGTTTCTTTCTAAAAACATCAGAACAGTCATAGACAGACGGTTAGAGGCAGACAGCAACAAACGTATTGCAAGTTAGCCACTGTACTTCCCTGTCTTCACATGGCCTCAGACATCTTGTCCGATAGTGTTGGCCTACCTTCCTTGGCTAATTTACACACCCCTCTGTGTATTCAACTTGACGGTGCCATTAAATATTGAAGGCAGAGCAAAAAAATATGACAAAAAGGGGAGAAAGTGTGCGCAGAGGGAAACCCTGTGTAAGAGAGAACGAGGGAGGGCAAGCTAAGAGGAAGCAGGTAGAGCTATATTGGAGATATACCAAAAAAGTGAATCATCGCTTTGTTTTCAACCGTGGCCCACACTATATCTTTTTCTCTTTCAGTAATGACACAATGTGGCACACAACGCCCTCGCGAGGGGTCTGACTAATGAGGGAGTCCTCGCTCTCAGGTGTTTTCCGTAAGAAAAGCAGCCCAAAAGGCAGATCGTTTGTCTCCGCCGAGACGCAAATCAGTGTCAAATTTGACTTATTCGCGCTGACAGATGTATGCGCAGACATCCTCAAAGTATTCAATTGCACCAGGCCTGTTTTGTTGGATCTTGAAATAACGTTTACGTACAAAATAGAGGTAAACACTGAGACCTGCAGGTGGACATTACGCATATACTGTAGCTACAGCTGTCAAGAGCAAACGAAAGCCCCGTTTCAATGTTGTGCCCAGAAGGAACAGAGGCAGGCTTCAGGCCCTTCATCGCCGTAGAACAGAGCCAAGCAGACGCATGAAAGAAGAGAAACCTCAGAGACAGATGAGACGAGAGAAATGTTTGTTTGGTGCTTGTTGTGGTTCACGTCTCCAAACCACCTCGGCAGTCTCTGCTGTTCTCCTCCCCAACTGATCCCCGTAGCGCTCATTAACATAAAATATGGGGTTTGTTGTCCTTAAAGGGCTTTTAGTGGAATTACTTCATCATCTCATTAGGCGTGTTAAAGGAAAAGTGGGGATGTGGTATGCTGCCTAAAAAACTAAAATGGTTTTAACATTGTGAGGTACAAAATTTGTATTCAAATGGATCTCAAACTGGTGCGACCTCAAAGCATTTGTACTTTGATTATTTTTCTCTCTCTGTTGCTGACTTACACAAGTCTGCCCTGTAACTACAAAGTGTCTTGTTTTAAACGTATTCTTGGTATCAAGGATAAAACATCATGCATTGCTTTTTATACTTTCTTTTAAGGATTTGCATGAAAACAACACAAGTACAAAATAAAATAACTGTGTCTGCTGTATGCTGTTTAATTTAGCACCTCCAGTTTTTGTGAAACCTCTTTCTGGCCGTCAACAACTTACAGCAAGCGTCGGCCAGAAATGAGACTTTTCTAGTCAGTATTTGAACATAACCAAGGCTACCACATCAACGCAGGTCTATAAAGATATTTAAGATATTTATTTAGGTAACAATCCCTAGTTGTGCGCTCGTTCAGTTAGGACCGCAATAAGCCCTCAAACCAAGCTCATGAAGTAAGAATATAAAGATCTATAGATGTTATTTTGTACTGAAAGGTTTACTGTTGTTTTGACTGTACACCAGAACAGAGCAGAGTAAACAGCAGAGATAAGCACACACAGGTGTTTGGCTGGTAAACAGGTGTCCATTGACGCCAGTCTGACTAGAACAGCGAAACCCCCCTGCACACAGTGACACAGAATTTGTTCTTCACATACCGTTCAGTGACACACAATGAAGCTTTAATGTAACACTGGTTTTTACGTGGAAACGACCTCATGTCATGCAACAGAAAGACTTATTTAAAGAGGTTTTGAATTCAGCTCTCAGATACATTTAACCAAGGAGGCCCCAGCGCAGCGGCACCACTGGGTCTGCCTCCTTCCAGTTATGAAATTGTCTGTTAAATCACCAGGATGCCCTTAATTGCCATGTTGAATTATAATCGACTTTGCTACTATCAATAATTATGACACCAAACAATGACAAGATGAATGGATTGTTGAGAAATGTTAAATCTCAGATCTATCTTTACTATGTAAAATTCTTGTGTGTCTTTCCAATAATTTTTAAATACACACATTAAGTTAGATGTTGCAAAAAGAGATGACTTTAGCCAGTTAATCCTTGTTTTCACACAATTTGTGGACTATTAACTATTATGAACTTTAGTATATGTTCCTAATATCCATTCATTTTGCTATTTGGCCGTCAATACTTTGCAGTACTCATAATATTTTATGTCTGTAAGAAGTGGGTTTATAACAAATGAAAGCGTAACGTTCTTCTCCAAAACACCTTAAAGGTCTAGAGACTTCCAAGGAAGATATCCATCTTAATCCTGATGCTTGGTGGACTTTGGAAAAGGGTGTTCAGATTGTAGATGACTTGGTGGCATTGTTAAGTCTTTCGAGCAGCTCAGGGAGCAGAACAGTTTTCCGACAAACCATTTTTTTTAGTTACCTGCAGCGGAGCAGTTTTATTCCTGCCGCTGTTAGGTTACACCCCAATGGGTTCGCTCATCAGAGGCGAAGCTAAGAGACTGGCAAACTAGGCAATTTCCTTGGGCCCCCAGTCAGATGGGGCCCTGACCCCTGGCTACGATGAGACAAAATCAATTTTCAATTTAAGTGAATTATTGACGCTATCATGCATATCTCATTTCAGTTCAAAATAATATTGATCTGTTATGTGTTTTTGAGAATTCAGCAGAATCATGATCATGTCAATCTTGTCCCACCCCTTAGGCGGCTCCTTTGCCTGCAAGTATGTTTGTAAAAAGAAATATATATAGTAGGCCTATATTAGGCTAAATTTGGATGACATCATGATTGACCAATGATGGGCCAGCGAGTGATGTTTTTTGTCATTTAAAGTGATAGTCCGTAACTTGCCGTAATCATTGAGTGTTTGCACGTTTGTACAATTCCCAGAGGTTGGTTCACAGTCACCGTGTCTGTCCGCACTGATGGTTGCGCTTTTTTTTTTAAAAGACCGACACACTTGCATCTTCTTTGGAATAGTTGCAGGTTCAGTTTCTGAAGATAGCACTGAAGATAGTCTTGTCTTCGTTTTTTTCAGCCATTGTGTCTCACTTGTTTACCTACTTCCAACGTTTACATAAAATAAGTTACTTCCTATGCGCGGAACTAATTTGTTTCGTAAAATGTCGCCACCAATACTGGAATGAATTTCAGTTCCATTCCATTCCAGCTGTTGACAGTGATTGTTCAGTCACAGCTTCATTGTTCATGTATTTGATTTCTAATAATAATAATAATTTGTTCCAAAACTATAGTGTGGTTTAAATGACGTGATCTCTGCCTAGGGCCCCCAAAACTCTAGAAACACCACTGTCTCTCATTCCCCTGTAGAGAGGTTTCATTCAAGAAAAAAAAAAAACATATTATCAGGTTCTACAAAGACTTGTCAATCTCAGCTGAGGACACAGGTTATCTTAAGTCTCTCTGGGAGGCTGATCTGAGGGAAAACCCTAGATGAAGACTGTGAAAGTGTTTGGTTAAGCCTCTCTGGTTGATTATCCACCAACAAAGCTAATGACATGCAGTTTAGAATTACAAATAGACTCCATATTACTCCAGTGAAATGAAACAAGTTTAATCCAGTGTTTTCAACATATTGTAATAAACGTAAGGTTTCTGAGGGCTCATACTTCCATTGTATTTTTCATCGTCCCTTTTACGAGAGACTTTTGGGAAAAGGTTTGTAAAGAAATTGACGTCACGTTTAGAAAAAGCCTAGACTGCAAACCTATGTCGTACATTCTTGGGAGACACTCAGCACTGATCCTTAACTGCTCAATGTCCTTTTTGGTGCAAGGAGATGTGCACCAGAGAGAGACAAAGAGAGAGATTTCTGATAAACCCCCTATCCTCTCACAGCGGACTCACAGCATAATGGAACGTATCCCTATGGAAGCTATAAGCTTTTGGCTCAAGGACAAACCTTTGAAGTTTTTTTTAAATCTGCGACCATTCTTAGGCTATATTGGAGATAGAGGGGCCCAGGCTCTACGGAGCCGACTCTACGGTCTTACATGGTCTGAGATTCCTAGAAACATTTACATGCGCTAAACAAGCGGTATAATGTCATGTCTGATAATCCCTGCTTTAATTAACCATTAACATTTTTAGGTTGTTTTGTCTCTGTATACAGACAGTCCCTTTTTTTCTTTTTTTTTTTGGTCTTTCAAAGATATTGTATCTATCGTCTATATGTTTTTGTGCTAAATGGCTGACCGCCTTACTGTTAAAACCAATAATAAATATGATTTAAAAGACTTTGCAGTACAACTGATGTACGTAGCTCCCAATGTCCAGCAGTATTTGCCAAAAATGTAGAAAATATTTTAACAGTTAAAAAATTATTTTTTCTAGATCTTCAGCATCATCAACCTGACTTATTTAGATATATTTCCATCAAAGATTGTAGACCAACTCGGTATCGTGGTCTTTTTGAGCATGCAAGGGTAACAATAGGAGGGAGCACATTATATGCACAGTCGTTGGTTGTATTCTTTGGATTTAAAGGTTATTATTAAATAGGATTCCTTCACTATATGGGCATTTAACTCAGCTATGTTACCCCTTCTCTTATTTTACAGCACGTTCAACAGTTTTGACTAAAACAATTGCTCTTCACTTACAGCTCTCAGGAGTTTAACTATGTTTTTCTTTTTAAAAACATTTGCATTTTTAATTTCTATTTATTATCTGCTTTCGACATGATTGGGCTAATAGTTCATGGCGTGAATTCTGATACACTAATTAAAATACTTTAATTAGTACAATAATGTGACAAGGGGGAAATCGACTGCGGCGTTACAATAAATTAAGCGTTATCCACTCATGTGTTAGTTAGAATGTGCCAAAATTTGATAACGAGTCCAGAGAGACACAAATTATGACTTATGTTTTTGTGATAAGGTGATAAGTCAAAAAAAAAAAATTTTTTCACTCAATTAAATAATTACTTATTTATTTGTTTATTTGTTTGTTTGTCTATATATCTGGTTGATGCATTAGAAAATAAAACAAAGTATTACTTTTTACCAATCAACCAACTAAAACGTATTGCAGTTACCTTGGCATTAAAAGTGTTATATTTTCATTTAATATAATATTACAACATAGGCCATACATTTAGGCTGGCCTAACAAATAAGTTTAACAACTTAGATGGTTGTTAGCAGAGATAGAGAGGAAAGTTTGTTGAAGAAGTTCCACCCTCTATCTCTTGCGAACCAATCAAAAGGTCATTATTCTGACCTTGGTTTTGACCAATAATTGTATGCTTCTTTTTAGACCAATCAGTTGAGAAGGAGGTGCAACGCCACTCAAAACAAGCTTTTTCTGAAGAGGTTCACCATTTCCGCCAGGACCGTATCATTGTAGAGGGACACTCAACTACTGACATCACCAATTGTAAGTATACCCAAGGATTATGCCATACTTGTTACCGTAAAAGTTTCAATGTTACTTACATAACTTGCAGAAAATGTAACATATTATCATTCAGAATAACTCAGAGGTTGCTAAGTTGATAATCAAATGATTGATATGAACGTATATAGAGAGACAGAGCTATGCTGAGTTTAAAAGGAGCAGGTTGCAACATGTTGTCATATGATAATACTTTATTATACCTAATAGTCATTTTGGATTATGAATCTTTAAGACCTTAGAGCCGGAACTGTCATTTAGGGTCAAATTGATTTCTATACATCTCATTTAAAGTCTTTACGAGATCCGGTGATGAAGCATGGCACACTGCTGAACCTATGACTTAAATTGTATTAAACACCAAACTGTCAACATTTTAATAAACAGGAAAGAATGACATTAACGGTCCCCATTACACATGTTTGCAAACAAATAAGTATAAAATGTGTTATTTTATTGCCAAATATCTTTCCATGTTGAGTGCCGTTTTGATCTTCTAACATCACGCAGGGTAAAAATGCAATAGTGGGACTGCTGACAGGTAACGATGCACAGTGACGTATCATTATAGATACAATGTTCAAACTATAAAAGTAGAAATTACTTATTTATTTCTTCATAACAAAAAAAAAAAAAGCTACTTCTATCCAGGTTTGCATTATTACTTGTATTTTACAGTCGGATGCTGATAATAGTACCCGCTTATCATATTGCCAACACCGCACCATCATTAAAATAGGGCCATGTTCCACTGATTTGTTTTCTTCTATTTCTTTTTGTCGGTTTAGTTGTAGAAATACAGTATAAACAAACACTCTCAACAAGGGACGCACTGCTATTAATGCTTATTTGTGCACAACATACACCTGAGTTACACGAACAATCACAACCAGGCCGACACAAGTTAGTACTGCTTTTCCCCAATTAATTTGCTCAGTAATATAGAAAGATAGTCCTGTAACTTTTAATACCCACTGTAATTACACAGTACATGTACTAAATGTGTGAATTTTCTTGACAAATTAAAAAAAAAGAAAAAGTCAAGACATAAAACTAGGCTGGGGGCAGTCCACTAACTTTACTGATAAACAATGTTACTGGAAGTGAAACTACACTCTAACATTATGTAATCCTGACCCTATCCTATATCTTTCTTTTGTCTCAACTAAGTCTCTTCCTCGTCTCCACCTCCCCACTGTACTCCAGGGTACATTGTACAACCCTGCCCTCCCCTCACCCTGTCTCTTTGCTGTTGAACCTCCGCCCCGACCACAGAGAAGATCATCTGTTTTCTGGGTTAGCGGCGCAGGTGTCACGTCTTCGTGTGGCTGTTTCACATCACTTCAACGCTCTCTTCCGCCTCTCCTCTGTGTTTCTTTCTTTACAAACTCCGCTCTGAGTTCAGTATTAATTAATGCAGTATTGCTGTGACATTGTTTATCTGTACATACTCCCATCTGGGATTCCCATAGGTTTACATGTGTACCATATTTATGTGACCTTCATTGCTCTTGCCTGGCTGTAACTAATTTATAGTTTTAATTATACATAGAAACAACACATTTGCTTTTAAGTGTTAGTTCTTTACAATACACCGCCTCAGATTTCAATATCTCATGCAGCATGGCTTTGTGCTCATTGAAGCTCGCTCAGTCTTTCCTTACAAACTATGAATTGTCTTTGATGTGCTAGTCTGGTTGTAAATTTGTTGTAATTGGAACATAGTAAACTCACAGGGGTATGATTATATAGAAAGTATAAAGCTCCTTTGTTGTTGTTTTTGTACTGGGTTCAAATGTAGAAAACGCAACAGGAGCAAAAACGTAAATCAATAAAGTACTTAGTTTGAGTTGTACATAATGATTAATGTACTGGTACCACTGTGGAATCGTGATATTGGGCCTTAGGACACAAACTAATTTTAGCCAACTTGACAAATGTATTGCTCATTATATTTTATATAACTGGTACCCCGTAGAGATCTTGGCTGCAATTCAAATGATGGTAAGATGGTAGGAATTTGTTCCTAACTTTTGAAATTACTGTAACACATAGTCCTAGATATTTGTCAAGACTTTATAAATGACTACAAAATGAGTGTAATTATAATGATTGCATGGTTACGATGCAAATGTAATACATGACCTTACAACACAGCAGTACAACTGAATCAGTCGGTCTAAATATCAAGCAAGCACAGCCAGATGAATATTATCTTAGACTTGAGATATTTTTCAAGCAATTTAAACTTGTCTCAGACTTGGACCAAATTTTTTCTTTGTCTCTATCATATCTCGGGCATTTCTGTGTAAGGTCTTATAATTGTCACCTCAAAGAACTGTGCCTTATACTATACTAGAAATTAATTGACGATTGTCAGTTTCGTATAATTTTCGATAGATTTGACCACTCGTTTTGGTCTGAAGACCTCTCTCTCTCAACTCGGGTTCGACCCTCCTTTGGCCTCTGTCTCTGTTTTGACTCAGACTAAACAAGGTCCAGTTTTGGTTTGTCTTAAATGTGACTCAGGTGGTCTTGACTGCAAACCTGTGCGTGAATATAAAAATAATAAAAAAATAATGGCTTAGCCTGACTTTCACACTCAGAATGAGAAAAAAAAATCTGAATCTAGTTTTGAGTGCCATTATATCTCGGTAAAAATGCCAAAACTAGAGGACTAAATTAAATGTGGTTCTGATAGAAAACAACATATTGGCCCATATCGCTTCCCAAAAATCAAAATAAGTGCAACAACAAAGATTTTTGCACAACGATAACAAAAGCACCAAAAAAACAAGAGAGAGCAAGAACAGAGGGCAAGGGAGAGAGAGAGAGAAAGAGAGAGAGAGAGAGAGAGAGAGAGAGAGAGAGAGAGACATGTACCTTAAACCGCAGTCTGGGGCACTACTGGTGTTGTGGTCTCTTTTTTTCTTCTTCTTAAGCGTGGAGAAATCCGTCTTTCGTGGAACAGGGGAGGAAGAGAGGGGGTGTGAGAGATAGAGAGGGGGGGGAGAAGAGGTGAGGGATTGGGATAAGAATCTACACCTCACCAAGTCAGGGCCCACCACAACCCGGCGTACGCGTTCTCTCTCTCTCTCTCTCTCTCCCCTCCTTCACTTTGATTTCTTCATCATCCAAAGCCCACCACAGCCCTCCTACTCTCACCTTTCTGAACTCTTCCTCTTTTATCTTTTGCAAATCTCTTTAAATCTGAGACCACCACAAACCTCCTTCCTCTTTCTGTTGACATTCACTCTTTTGTGACGTAAAGTGGTGATTATAACCGCGACGTATATAACTTAATGTGTTGTTATAATGGCTTTTAATATTGTCAAAATATAATATACTTTTAATTTCAAAAACATTTTCTGCTAAAATGACATTTTAAAAGGAAAGCATGTATTTCATGTTGTTAATGACAATAATTATATAGATGGATGTGAAGACATGTAAGAAAAATCCACAGCTCTTACACTCAAAGTTATATCTTATTTATTCTTTCTCTTATATGATTTCATTTTTATTTTCATACCTATATATTCAAACGGGTATGTATTAAGAAATATGTCACTTGCAACCCACTAATTGTCTTTCAATACCTTTATTTTTCAGACGCGTGACCATGAAGGGTTTTATTCAATCTCTCGTCATCCTCCAGGTTGTCCTAAACACAACAACAGGTAGGACACAGTGTTCAGGTTGAAGCCGCTTGACTGTTATATTCCCATTGCGACAGGACAGGTGACTCTACTGTCTCGTCTCTTTTGTTTCAGCAGCTGTGGCTGTGGTGGTAGTGTACGGCCAGGTGGGTGAGCGCATCACCCTGCCTATGCCTGAAACAAACCCCAGCGAGACATATGTGCACTGGTACTTTGGAACGGCGTCAGAAGGCCTCAAGGTGCTCTCACGCAATTCCCATAGTCATACAACAATTACAAGCGGTACGTAAAGTACTTTCCATTATTCACAGATATTGACCTATTTTAGAATCAAAAGATAAAATAAATCAGTGGAATGCAAATTCTGCAAATCTTTGTAATGTTGGTAATGGCTTAAATACTACCGTGCAGATGCCAGATGGAAAGACAGGTTGTCCTTGACTGGCTACTCTCTGATCATCAACAAGATCCAACAAGACGAGTTCAAAACCTTCACCTGTGTGCTGGCGAACAGGTTGGCTATTACCTCGACCACGACGTATCAACTATACCCTGGTAAGAGGAGGTACTAAGAGAGCTGGTCTGTCACCATCTTGTAATATCAGTAAAACCGAAACACCGCAGATCAGTATGTATTTATATAGATATAGAGCTAGCTATGAGGCTAAAATATTTTACCATCTTACGTTTCTATATTTCGAGAAATACAACGTAGACATTGGAAATAAATATTGAAATTATTTTTATATAAATATTTTTATTTTCACTTTCCTTCCCATCCATTTTCTCCGTTAGTGCGTGTATCACCTGCCTCGAGTCTGTTGGCTGGGGACTCTCTATCTCTGTCCTGCGAAGTACTGAGGCCTAACAAACAACCGCAGATACACTGGCGCAATCCTCAGGACCAGGAAGTAGCCACTGGCAGTAAACTCACTGTGAAGAATGTCACGGTCCAACACAACGGAATGTGGACCTGTGTTGTGAAGTCTGACGGTCCAGAAACTCGTGCCAAAACCTCTGTTACTATAGTGGGTAAGTTACGGTACAACGGTGGCGCCTTTTGATTATGGCGTGTTTGTGTGTTTGCAGTTGTGTGTTGGGTTGGTGTTACAGCTATACATCTAATGAAGACAGCACACACACACACATAAATAGTGTGCGTTTGTTTATATTTGTGTGTGTGTGTGTGTGTGTGTGTGTGTGTGCGTACATACAGTACATGCACGAATGTACACACATAAATACACACACGTTACTTTTATATCAGTGTGGTCTTAAGCAGCAAAGAGTTGCTCACTGCTATCTCCGACATTTGTCTCTCCTGCTTCCTACCCAGACCTCTCCCAAGCCCCTTCAAGCCCTCGCTACACCTCTCTCTCCAACCGTCTCGAGATCCCCTGCTCCATCGCTCCGTACCTCTCCTGGAAAGTCCTCAAGGAAAAGGGCATCCAGAGAGGGCACTGGAGCTTCACCCCCAGCGTCCTCTCCGGGAATCCCAAGAAGCTCTTCTCCCTCTCCGTCGACGACCCGCCGACCTGGCAGGTTGAGCAAGACAGAGGACTGAGCGCGTCAGACCCGAGTAAAAACGACCTGTCTCTGCGGAGGAGGCTGGCGACGGAGGAGGACCGGGGACAGTATACCTGCGCCCTGGAATTCACGAACGGCGTCACCCTGGAGCGGTGCGTACGCGTCGAGGTGTTAAAAAGTAAGCCGGAGCGAAACGCATCGCCGTAACGCGCCCGCCTCTGTCACACGAACGCGGAGAAAATGGATCAGTTTTGATCTCTTTGCGTTTGTTTGTTCTCCGAGGCCGCCACAGTCTCCTCATCTATCTTTTTTAATCTCCTGTCTCCTCGCTCCTGTCTCCTTGCTCCTTCCTCATTGCCATTCATAACAAGTAGGACACAGTAATGTCTTTGCCACCTTTTACTTCCGTTCTATCTTGCTTTGCCAGTGTTCCCATCTCTCTGTTGCGGGGTTTATTTGGAACAGAGTCCCTGGTGTGACATGTGTGTAATTTAGTGGCTTATACACTTGTCTAGCTCCACTTGTTTTAATAGGCTATAATGAGCCTTCGCTCTGCCAGAAAAGTGTGGCAACACAATTTTAATTGGATTCTTTGTGTTCTGAGTATTTATAATAAGATATTTTTTCATTCCTTCTTTCCCTTATCCCTTATTTAACCAGGCATGTTGATCAGGAACAAATGTATATTTATAAAGACAGCTTAGGACTAATTCCATATACACACTGACACCTGGGAACAGCACAGGACAGGACACGTTCGCCTCTGAGACGCCGTACTAGAGCAGCGAGGGTCCGATAACTATCTCAAGAGCACGTTTCAGTCATATTCCAGCCAGCAGGGGGACGCAACTTGGCAACGTTTCCCTTTTCTGATAATTCCTCCGAATGTTTTGGAGCCACACAAATGTGTTTAAAAGGAACTTGAACTGAGTTTATGATTTCGTCCTTTCTTTCTTTCTCTCTCTCTTTCTCCAGTCGTCGCGTCTCCAAATGTAGTAGTGACCCGCGGCCAGCAGGTCAACCTGACCTGCAGCCTCGGCCATTCCCTGACCTCTGAACTGGAGGTGAAATGGATCTTGCCGAAGGACTCCTCCCTGCCCCCGCCGGCGACCCCTCACCCTGCCCATCTCACCGTCCTGGAAGCGGGCTACGGAGACGGCGGGACGTGGAAGTGTGAGCTATGGCGAAACCAAACGTCGCTGACATCAGCAGCAGTAACGCTGAAGATAGGTGAGCACAGGAAGTGAGAGGAAATATAAACAGGAAATATGAAATTGGTATTGGTATGAACTTTGTAGTCACAGTATTTTACTGTAGAATACAGGCTTTAATGGAATCGGCGTGCTCTACGCATGTTGCATGAGGGTTATTCGTCTCTGTTGTGCACAGAGAGTGCCCCGATGGGTGTGTGGCTGCTGGTGACCATATGTGGTGTGGTGGTCATCGTCCTCCTCCTCCTCCTCGTCACCGCCATCCTCATCCGACAACGCAGACAGGTATTGCTTTGTCAGCATTTCGCTTTCTGGCAGATTTACCTAAGAGGCATACTGGATTAATGGTAAGACTATCATGCTAAGAATGCTCTCTCTCTCTCTCTCTCTCTCTCTCTCTCTCTCTCTGGCTGACATTAGCGAACGATGAAGCCAAGGCACCCCAAGCATAGATTCTGCCGCTGTAAAAAGTAAGTACCACTTCCTGGATAATGTGTGAATGATGCCCTTGGATATGCAGTGTTGTGTATAGACACTGTGCAAGTAGCCTAAACATCTAGTTCTTTAGTCCTTATCTTTCTTCAGTTCTGTCTGTCTCTCTCTCTCTCTCTCTCTCTCTCGCTCCGTCTGTGGCTCTAACCAACACCGCTGTGTTTTGTTTCAGCCCAAAACCAAAAGGATTCTATAGAACCTAGAATCTTCTAGGGCCCAAGAGATTCTCAAGAGGACATCTCCTCAAAGTGAATATCCAATGAACTGTGTACCACTGTCATCTGTTTTAAGGGACAGACAGAGAGATTGAAACGAGGGCCACACATCAGTGATCTTTCCTATCAGATTGTATTATTTCGGAACAGTATTTGGCAAATGTACATAACGATCAGTCAAACACATTATGTTTATATTTGTTTGTTTTTGTAATTATACTAAATGCAAAAGGAACTTTTTTTTGCAGGCCTTTGTAGTTGACTACTTGGGTTTTTGGATCCGTTTTACTCAGCATTTCAACATTATTGTTTATGATAATCACAGACATTAGTAGAGCTGGATGTTGTATTCTCTTTCAGTTCCATTGTATGTGATCTGCCGTTTTCTGGTCTTTTTTTTTAGACCAAATGACCCAATTATATTTTTGAACTGTATCACGCTCTGTTATACTGCTTTGATAATATATGGTAATGGTTACATGCTTTGATTTTATAAATATATTCAAATGTTTTAACTGTAAATTAACCAATGCAGATATACTGTATCATATGACACGTGCTTGCTTTATTAAGGATGCCACACAGTCCACCTATCTACAGTGTGTGTGTGTGTGTGTGTGTGTGTGTGTGGATTGTATTTTACATGGAAAGTATAGTAGGTGTATTGTTTTTCATCATTAAAGTGTTTTCATTTGACTGGCTGAATGTATATGAACATACTGCTTTTGAAGCGTTGTATTGTCGACCCGTCTCCAATGTTCTTCTTTCTGCTGTCAGGCCAGTCCAGTAGTATGTATTAAATCCCACAATCCTTCTGGCTTCTTTTTCCAGGGCATCCTAAAAAAGTGATTATAGCCAAGCGTATAGCAGCCAGCGTATGTACAAGCAAACACACTCGCGTACAGAATAACACTCACACAGACACATTCACACACACACACACACACACACACATAATTACACGCTGTTCATTGGTATGCAAGATGTCGCTGCTGTCTCTGCTCTTCCACCAGTTAAACTCTCTCTATCGCTAATGTGAAGCCACCCTTCAGGACACTCAGTGTAATTTATTACTGTGAGCAGGGGGGGCAACAGGAAGACATGAGGAATAATCAGACAAACACAGCAAGCTGAACTAACCTAAAGTCAACCATTTACAAAGATGCACAATACAAGTGGGAGGGCAGAGCAACATGTATCGACATAGAAACATGTTAGTAGGATGTTAGCATTCTTTTGCTAGAATCTGGAAAAGGGACATACGTAGCTTGGCAGTTGGATGCCATGTTGGCAAATTGGGCACACAGGCGCCTGAAGCAATGCATCACAGAGCTCAATCTCAGCCTCGGGGAGAACAGCTCTCCCTCTGGATTAACCCCAGAACCTTCAGCCTGATTGAAGCCAATAGAAATCAACAGGCCAATCAGCACAAAGAGGATGGCGTGGTCTCTGAAACCCAGGTGCCTGATTGGACGGATAGAAGAGTGTAACTCTTACATCCACACCATTACAAGGACAGCATTAAAGCTGCTACAGTATGTGTTTCTGTTTCTACCAATAACGAAGGGGAATTACAACAACTAATTACAGTTAAACAACCTGGACTTTTACCCTCCCCTGTGGCACCGCTCCATTGCGCCTTTCCCCTGTCCATCAAAAAACCTCATACGCTACAGGTGAACAGATTCTTGGGAGGAGTTTATTGTTAGGCCCCGCTGTGGACAATATTCAAATGTTAATTAAAATTTGAAAATTAAAACGCAATACAAACAATGTAACCTCATTTTCCATTTATGCAAGAAATATTTAAGTCACAATTAAAATTAAAATGCAATTTTCTAACAATTTGAAAATGAAAATGGCATTTGACATTTCATTTTCAAAGGCTTAAACTGCTGTACAGTGGACAATATTCAAATGTTAAATCAAATTTGAAAATTAAAATATAATTTTAATATAATAAAACCATGTAGCCTAATTTTCCATTTGTGCAAGCCTTATTTAAAATGCAATTTTCTAACAATTTGAAGATGAAAATGAAAACGGCATTTGGCATTTCATTTTCAAAAAAAGGTACTATATGTAGTTTTATGCTTTTAACAGATAATAATAACATTTCGGGTTATTGCATAGTAATGGTCATGGGAGAATGAAGAAATCGCGGTTCAGATCAGTTTCCTAGAAGCCTTGTGTTCACTATGTAATTTAGTCTGCGGGAAATATGACGTCTCGCTTTACAGCACATATAAAAGTAAGTAATTTTACTTTACCACCTCTGTGAACATGTTGGCATTTCGACACCACACCATGACAGACAGCTGCAATCCAACCACACTCAGTCATGAATGTAAACAGTGGCATGTCAGTTTGCCTTTGCCTTTGCAACAATTGATAAATACCACTAGCAAACTCTAGAGGCCGCTATAAACCGCATACAGTACCTTTAACCTGCTGTACGGTGGACAATATTCAAATGCAATAAGCGGAAATGAAAATGCATCAAGGCAGGTTTTCGGTTAGGACGTTACTTCTTCGTACAGTGACTTGGTTCATTAACATGTAGTAGGGGAAGAAATCTCCAAAAAAATGCTTTAGTTTTTGAGATACCCCTACCCGAAGTAGAACTTAATTTTCAAGTTTACATTATCATTTTAATATAGGCTAGTTCCCTATAGGCTTCCATATTCCTAATTCAATAGAACTGTTAGTGTGTTGAACTGTTTGAACTGTTTGGACTGTTTGGAGGGGAAGTGAAAGTACAGATTACAGAAATGTACTGGATTTGGGCCAGACTGGAATGGGAATAAGCATCACCATCGAAGTGGAAAATTTCAGATGTTGCAATTATCTGGCAAATTAACCGTTCGTGACATGATATATTGAAATATATGAAACATATGACAGAAATACAGTTTGATGGTTTCACTGGGGTTCTGTCATGCAGCTTGGTCATATATTTATTTATTAACAGGAGCCTGTTAGAATGTTAGATTCTAATACGTTTGATTTTATTATTTTTATTGATGTTGTGTAACATTTACTTTGAAGTCCCTTTTTGTTTCTGCTTTTACTATCATCTGTTAAGCGCTTTGAGCTACAATCCTTGTATGAAAGGCGCTATATAAATAAAGTTTATTATTATTATTATTATTATTTACCATTATTTA

The utg7180001882239 sequence encodes a full-length canonical CD4-1 molecule:

MKGFIQSLVILQVVLNTTTAAVAVVVVYGQVGERITLPMPETNPSETYVHWYFGTASEGL

KVLSRNSHSHTTITSDARWKDRLSLTGYSLIINKIQQDEFKTFTCVLANRLAITSTTTYQ

LYPVRVSPASSLLAGDSLSLSCEVLRPNKQPQIHWRNPQDQEVATGSKLTVKNVTVQHNG

MWTCVVKSDGPETRAKTSVTIVDLSQAPSSPRYTSLSNRLEIPCSIAPYLSWKVLKEKGI

QRGHWSFTPSVLSGNPKKLFSLSVDDPPTWQVEQDRGLSASDPSKNDLSLRRRLATEEDR

GQYTCALEFTNGVTLERCVRVEVLKIVASPNVVVTRGQQVNLTCSLGHSLTSELEVKWIL

PKDSSLPPPATPHPAHLTVLEAGYGDGGTWKCELWRNQTSLTSAAVTLKIESAPMGVWLL

VTICGVVVIVLLLLLVTAILIRQRRQRTMKPRHPKHRFCRCKPKPKGFYRT

***Polymixia japonica CD74a***

>utg7180001886650 length=5421 num_frags=626 Astat=211.00

AAAGGATTTTTTTTTTTTTTTTTGTCAGCAGGGAAGTACAGACAGATTTCTGAAGGATTCATAACTCTAATCCGTAGCCATTTTTGTATCACTTGGCTGAAGTTTGGCTGAATGCCAGATACTGCAAAGAAGCTAATAAATGGAATATTGGCCAATTTCTTTTGGCTATTTATTGATCCTTGTTGTGTTTGACATACATTTTAACTTAATGAGGATACTATCCATGGCTAATGTTTTTTCTGTATTTTTTTCATCGCCACTTATAGGTAAAATACCTTGCATCCACTGTAGAACAAAATGAATCCTGCTCTTGTGCTGTAGGAGAATCAAATAGGAGTCTTCTTCAATATTAGATACATTTCACATTAAAAGGTAGATTTACCCACAGTGAAATCTCTTTAATGGTCACCATGCCTCACTAAAATCTCTATTATTATTATTTATATCCTTGGCTAAAATACTTTAAACTTTAGGCCTTAGGTGCTGTGGATTTAAGTTAAGTTTGTACCACAAAAAAAAAATACCACACATTATTTATTTTTTGCTGTGAGTTCAACATGGTTTCTCAATAAGAATTCTCCAGGTATTCATTTATTTTATAGCACTTCATAACTAAGATAATGAACTGAATAATTATCTATCTATCTATCTATCTATCTAGTTGTATGCAGACGTAATATCAAATAAATGTTAGAATGCTTAAATCTGGTTAGGTCGCTGAACATAATAACGTTTCACTATTGTCTGCCTCTACCAACAAGTGATGATGGCTCCAGTTAACCAATGGCAGCACAGGTCAATTGAACCAGCCCACAGCACCTTCAAGACTGGGACTTTCCACTGCTTCATCTTTCAGTTCATCATTTGGTGGAACCTTATTCTCAACGCGCACGCCTCACGTATTCACTGGGCTATTTGAACGCTTCCTCGGACATTGTCCACTTACTTTTGTTGAAGGCACTGAAGAGAGATGGAACGGCCTCAGGAAGATGCGCCACTGGCCGCAGGGAGCCTGGCTGGGAGTGAGGAGGCACTGGTTGCCCCCGCAAGAGCGAGAGGGTGAGAGACATGTATAATAATAGGGCATACATTCACTTTTCAATATCTGCCAATCAATAGCCTATTACAAAATTGTAATCCCTTAATTTCTTGGCATTTTCATCGCTAAAATGTTTTAATATTCATTCCCTTATATGATTACCTTGTTTAGCCTTTTACATGTAGTTTACATGCGCCACAACTGGGTAATCCCTTTAGAAACAATATGTGCAACAATATGTGCTGCATATAGACAACATAGAGTAGGTTGTGTAAGACCTAAAAGTGGAAATAATAATCATAAGTAAAAGGCATTTTATCTTAAAATATCTAGATAATAATAAAAATAATATTTATCTTTTAAAGTATGTATTTTGTAGTGAGAGTGAGAGTTTATACTGAGAAATTAAACTTCCCTTTACAGTTCTTGAAGTCGTCTGCCTAGCAACTAGTGACGCCTCTCATATTATTGGCTGATAATGAGCAGCAGAGAAAAATACGGGAATGTATTTACATACTGCGTGAATTATTTTTGATAAGGAACTTGCTTATATGTATAAATGTGTTACGTTTTACTTTTGCTTTGAAATCGACATTATGGATAACTAATTTAATTAATTAATCAGAATTTAACTAAGGGGTCTTTCTTTGTGTTCTGGACTGTCCTGAGGTAAATCAAAATATCCACACGTTTTGGAAATGACTCAACTAATATGCTAGAATTAAAGGGGACCTCCCCCCAAAATCAATTTTAATAATTTAAAAACTACTTCTCACTTTTGCATTCTTCCTATTTGTATCTGCCTTATGTTATCCCTGTTCTCTACACTTGGCTATTGTCCCCTCTCTCCCACTTTTTTTAGCCCCTTGTTTAGGTTTTTTTTATTATCTTTTATCAAGGGACATCCGTGTTTTAATCGTTCTCTCTGTATGATCAGGGGCTCCAACAGCCGTGCATTTAAGGTGGCAGGGCTGACAACACTGGCATGTCTGCTGCTAGCCAGCCAGGTCTTCACAGCCTACATGGTTTTCAACCAGAAGGGGCAGATCCACGCCCTGCAGAAGAACTCTGAGACCATGCGTAAAGATCTGACCCGCAGTGCCCAGGGTAAGGCGTTAATCATCAGCCAACATTAATTGTCATCAGACAGAAGGGAGTTGAGGCTAGGACTTAGGAAATAGGGAACGAAGTGAGACTCTTCGAAGAAGGAAATGAAAGGGAAAAGACTGGAATGCGGAACACGGGAACAAAAACATTTGTGGTAAAAGGAGAAACAACAGCAGTGTTCTGTGTTAGTACTGTACTTATCGCCACAAGCATTTATCAGTTTAATCTATATTTATTTTACCATGTTTTCTGAAATTCTAATCATCTTGGCCATTACACTTCTTCTAATAGAAGGTCAGCCAATAAATCTAATAATAAGGTACAATAAATTGTTCCTAGCAGCGGCCCCAGTGCGGATGCACATGCCTATGAACAGCCTGCCCCTTCTCATGGACTTGACAGACGATGGCGTGGAAACAACCAAGACACCCATGACAGTAGGTCCACATCTAGGTGGCAGCAGCAGCCTCGTTTTCAAAACAGACTGAAAGTCTGTTCTGGATTATGTAAAAAAAAAAAAAAAAATCAGATGTTGAATTTTTAAAAACATAAATGTGAACATTTATTAAGAAAAAAAATGTAATACATACAATTTATTATGCCTTTTTTCCCCCCTATCTTTCTACTATACCACCAGAAACTGGAGGACACTGCAATTGTTAGTGTTGAGAAGCAGGTGAAGGATCTCCTGGCGGTATGTGGCGTTCAGCATCACGAATAGCCTGACATTGGTTACTATCCTATTCTACTCCTATCCTCTATTAACTCTGGATGTGAGTGGTTGTGATTAAATTATAGTTAATGTTTTTGATCAGAACTCCCAGCTGCCCCAGTTCAACGAGACCTTCCTCACCAACCTGCAGGGCCTGCAGAAGGAGATGGAGGAGAGTGACTGGAAGGTGATGTAACGCACATACACATGTGTACACAAGACACGCACGCTCAAAGCAAAAACATCTCGCGTGACTTTATGACTTGTCGATATCTTTACCTGAACCCTTTGACTCCTCAGTCTTTTGAGTCCTGGATGTATCATTGGCTGCTCTTCCAGATGGCCCAGGAGAAGCCGCCTGCACCCCCCACAGCTCAGTCAGGTGAGTGTAACGAATCTACCCTTCTCTCCCCGACATGTCATTTATGTTACATTTCAAGCATTTATCTGACATTCTTATCCAGAGAGACCCACAGTAAATCCCACAGTAAAATAAACTTAGATTCCTAGATCACCAAAATAACAATAAGCCGATAGTAATGACCGCAAAGGGGTTGGGGCCCAAGTTCCCTGACAGTATTTTTGTTTTGACGTTACAATAATCATGTAATAGAGAAGTGCTCGGAGAAGAGGACACATAAGAGTTAGTGAGAAAGATAGAAGGGATCCTTCGGGAGAGTTTCTGATGTCTGACAAAGGAAATAACGCACCGTGTAGCGCAACTGCCGCCGTCACATCTGACTCACCGTAGCTTCTCCACAGCACAGTTTGTCATCTCAAAAGCAGATAATTCTTCAATTCTTTCTTCACTGGCGATCTATTGAAAATATTAGTCCTGCACTATGCCTTCAGGTTCTCCATCTCTGTGAAACAGGCCACTTCATATCCTAATGTCTACTCTCTCCATCCCTCAGCCACTCACCTCCAGACCAAGTGCCAGAGAGAGGCGGCCCCTGGAGCAGGGAAGATAGGTTCCTACAAACCCCAGTGTGATGAGCAGGGCCACTACAAGCCCATGCAGTGCTGGCACAGTACTGGCTTCTGCTGGTGTGTGGATGCGACAGGCACCCCCATCGAGGGCACTTCCATTCGTGGCAGACCTGACTGTCAGAGAGGTAAATCCGTATCTTAACCCCCCCCCCATGAACAAGAACATTAAATTAAAGCCCACCTCCAGCCAATTTTACCTCCTGCTTTCCTGTCCAATTGATTATAGTATTATAATTATATTATATTGATTATAATTGCAATTTAACTTCACACACTACCAACACCATGGCAGCTTGGATACTGATACATACATTTTGCCAGTTTTTGCTAAAAAAGTGCTGTTTTTGGAGGAGCTTGAAATTTTGTATATTTGAAGCAAAAATTGCTCATTTGTCAAAAACGTATGGTTGTGACTTTGGAATCGATTTATAGCAGCAGAGGTTGGCATTGGATAATAATAAACAGCTCAAAAATGCAAAAACAAATACTGGAGGAGGATTTTAAGTGGACCAAGTTTTTACTCATTCTCTGTTGGTTATTCTGCCCAGTCCCTCGTCGTATGATGGCTTTCCCAAGGATGATGCAGTTGAAGACCATCAACGTTGATGGTGAGCGGCTCAGATTAACTTTTAGAGTACAATAGAGCTTGTATTTCAATGGTTGATACACAATTTAATTTATCTGTGATTTTTTTCCCAGAGGGAACAAACTGAGAGGATTGCGCCAGAGTACCTGATGCCTCTCTTAACGTAATCTTTCCAGAGAGATCAGAAGAAGTATTTTAAGAGCAGGATTTCTTCATACAGCTTCCACTTTATATTGCTGTGACGGTATTTGTGATTCACCAATAATTATATGATATGATACGATTATATGATGATACAAATCTTACATTTTTATTTGTTCATTTGCATATAACCTGATTAAATAAAATGAATACATAGACATCTGTTACATTTTGCTCATTGCTTTTAATGATTTTCTTTCCATAATAGCTTTTGCTTGGAGGTGATCTTGTTGACACATTAAGATTCAACCTATATATTCTGACATATGAAACAAGTTTAACTGTGTTTTGGTAGCATTTAAGAATGTGACTGTATTAGAAAGGGATTCCTCCATAGAGGTAAGAATAGAAGAATGAAAATAGTTTTTGGTTCTATAAACGATGACGTTGCAATACATATATGAATTGTAATAGAGACATAATCAAATAAACTTAAACCTGATTAGTTATTGTGTCATCTGTGATATTGCTGGCTTATTGTAGAAGAACTCTTTCCTTTTTAAGTTTAAGGTTTTGCTTCTCATATTTCTACCCATTTTAAGTTGGTTGATGACAAAATTGTAATAGATGCATTTACAGAATAAAAATGCATTAATTTGGTCGCATGATCTACAATAAGCGCAATAGAACTTTTTAGTATCTGTCATATAACAGATATAACAACATAACATTTCTTCTGTATAATTTTAGAGGGATTTTATGCGAGATATATGATTTCATACAAATCATTAATAATTTTAATTTGTCCCAGGTTCTCAAAACTGCCTTTGCACTACAATTGCCTTA

The utg7180001886650 sequence encodes a full-length canonical CD74a molecule:

MERPQEDAPLAAGSLAGSEEALVAPARARGGSNSRAFKVAGLTTLACLLLASQVFTAYMV

FNQKGQIHALQKNSETMRKDLTRSAQAAPVRMHMPMNSLPLLMDLTDDGVETTKTPMTKL

EDTAIVSVEKQVKDLLANSQLPQFNETFLTNLQGLQKEMEESDWKSFESWMYHWLLFQMA

QEKPPAPPTAQSATHLQTKCQREAAPGAGKIGSYKPQCDEQGHYKPMQCWHSTGFCWCVD

ATGTPIEGTSIRGRPDCQRVPRRMMAFPRMMQLKTINVDEGTN

***Polymixia japonica CD74b***

>utg7180000447839 length=2499 num_frags=258 Astat=111.00

(the complementary sequence is shown)

TAAAACAAGTGAACTTTGTGTTGGTGTGGGGAGACTTTTTGTTCAAAATGGATCAGAATTTCATCAGCATCTCTGAATCAATTTTCTTCAGTGTATTACGTACATTGTTTTGTTTACAGGAAAGGGGTAAGTACTATTTAAATAAAAAAAGATCCATAGTGCAACTTTCCCGATACTGAAGTTTGTAACCCTAGATTTAGCACCACTTCCTTCATATATTTCCATGTCATTCAGTCAATATCCGTACACACCTAAGATGGGAGGACTTTAACTGCTAATCGATGGGAGTTTCCCAAGGTCACCTTGATGTGATCATACAGGTCAAAAATGAGGAAATGTTCTTGCTCTGGCGTCACGCGGAAAACTATGAAAACCCTTCGATTTAAAGAAGGCGTCTGGCCAGCAACTTTTTCAACTATTTCATCATTCACTTTTGTCGTGACGTAGACACCGTGACCACGGTTCCTCAGCGCCACACTGTTCCGAACAGCGAAGTACCACCCCTGCTTACCTTCGCTCCTCCTTTAACGCCCTCTCTTCTTCTTCATGTGTCCTCTCCTCTCTCCTAGGTATTCATTCTCCTTTGCCTCTAAGTGTCTGAGGACATATGTATTTATCTGACGTTCGTAGGTAGTTCAGCATGCCCTTCACCTGGGGACTTCAATTAAGAACAGATTCAGAGAATGACCCCAGTCAACAGTGTGATTCCATGTTGATATCTAAACATCTCTCTCCTTAATGTCTTCTCTTTCAATTCAGTTTATTTCAATTCAATTCAATTTGCTTTATTGGCTTTACCCAAAGCAGTTTACAAAACATACAATTTGAATTCCAAAACAAACATTTTTGACTTGGTGGTTATTGGTTAATATTTACAACTGGTCAGTATGAGTATAAGATAAGGCAGTAAGATTGCATCATGCTGCTTACAATCACACCAGTTGTTTCAATAAAGTCGACTACAGGTTTTATTTAATTGTTTTTTACTCTGTGTGTGTGTGTGTGTGTGTGTGGTTCTGTTGGTCACTCACTGTCCTTCAGGTTGTGGCATTTATGAACGCTTTGGGCAATAGATATGCAGTGTTTCCCTTACCAAGAATTACTTAGACTAATTTAGAATTTTCATCAAGCTCTGTGAATTGTCGTACGACTAAATCCAACTTTTTCAGATATTGCTCCCTGGCGTTATGGAGTCCTCTCTCTCTCTCTCCGTCTGATTTTCCTTCTCTCTGGTTCCCAGTGGCTCTTCGTCTCGGGCCTTTAAGGTGGCAGGCCTCACCGTGTTGGCATGCCTGCTGATAGCCGGTCAGGCCCTGACGGCATACCTTGTTCTCAGCCAGAGAAGTGACATCAGATCTCTGCAAGAGCAGAACAATGGCCTGAAGAGTGACATGCAGAGAGGAAGAGCTGGTGAGTAGGGGAAGTTTACATTTAGATTTGGCAGGCTCTCTTATCCAGAGTTATTTGTATTAAGTGACAACAGCAACATTCGGTGATAGATGGTGACTTAACAGAAATGTATTTAAATTAAAATCTTCAGAGATCTTCAAGATTGCCACTTTAATCTCTTCCAACTTAATTTCCCCAATGGGACAAACAAAGTTGTCTCAACTCTCCATCACACTCTCTCTCTTTCTCTCTAGCCGCTGTGCCTATGAAGATCCACATGCCCATGAACATGTTGCCCATGCTGACTAGCGACTCCACTGATGAGGAGGTAAGGTTACACAGACCGCCATGATCTTTTGGTATCAGTTTGGACGAGGTAGCGTTGCTGTTTCACAATCTATGCCTCGCCACCCAGAACGGTTAATAGGGCCTATTTCTGGAGGATGAGTACCATTGTTGTAAGTAGGCGTTTGGGCAAAACTGAACGTGCCCAAATTGTGTCCCAAGCTGGATCACAAAACATACAGGGCCCAGTCCAAAACACATGAGCGTCTGAATCGGAGTCACCTATTTATTGTCACAACAAACCAACAATACGTGTCACAGCTAAATGTGAGATATAGATACGTTGATACTATAATGGTCCTATCCTTAGATAATCTGTGTATATACCAATGACTGACATACTCAAAAAATCCCAGTCTTGATAAGCTGATGTTGACTCATGTATAAAGTTCTTCATCAGTGAATTTTACTTTGGTTGTTGACGCCCATCTGGTTGTGGGTTTCACCCTATTCTCACTTCTGTTTTGTTTTTTCTTTTTCTGTCAGGGGGCATCCACCGAGGGCCCAAAAGCAGAAGGTATGATCTATCCATCTTTCTGTCTGTCTGTCTCTCCGTACTTCCACTCAACGCTCTCCTGCTGTAATTCGGCGAGAAGGATCAAATATCATGCGTTGTTTTCTTCCACGTTTTCTCCAAAGTTCAATCGATTACGTGTCCCCGCTCTCCTCTCTAAAGTCACCAGGTTCTGACTCAGTCAACTGTTCGACCTAACTCTCCTTTCCTTACTCCTCACCTCTCCCCCTCCGCTCACGTTCCCTTTCCTC

The utg7180000447839 sequence only encodes the middle part of a CD74b molecule. Below the encoded CD74b sequences together with the relevant part of the unitig are shown:

1150 1160 1170 1180 1190 1200

TGTCGTACGACTAAATCCAACTTTTTCAGATATTGCTCCCTGGCGTTATGGAGTCCTCTC

1210 1220 1230 1240 1250 1260

TCTCTCTCTCCGTCTGATTTTCCTTCTCTCTGGTTCCCAGTGGCTCTTCGTCTCGGGCCT

G S S S R A F

1270 1280 1290 1300 1310 1320

TTAAGGTGGCAGGCCTCACCGTGTTGGCATGCCTGCTGATAGCCGGTCAGGCCCTGACGG

K V A G L T V L A C L L I A G Q A L T A

1330 1340 1350 1360 1370 1380

CATACCTTGTTCTCAGCCAGAGAAGTGACATCAGATCTCTGCAAGAGCAGAACAATGGCC

Y L V L S Q R S D I R S L Q E Q N N G L

1390 1400 1410 1420 1430 1440

TGAAGAGTGACATGCAGAGAGGAAGAGCTGGTGAGTAGGGGAAGTTTACATTTAGATTTG

K S D M Q R G R A

1450 1460 1470 1480 1490 1500

GCAGGCTCTCTTATCCAGAGTTATTTGTATTAAGTGACAACAGCAACATTCGGTGATAGA

1510 1520 1530 1540 1550 1560

TGGTGACTTAACAGAAATGTATTTAAATTAAAATCTTCAGAGATCTTCAAGATTGCCACT

1570 1580 1590 1600 1610 1620

TTAATCTCTTCCAACTTAATTTCCCCAATGGGACAAACAAAGTTGTCTCAACTCTCCATC

1630 1640 1650 1660 1670 1680

ACACTCTCTCTCTTTCTCTCTAGCCGCTGTGCCTATGAAGATCCACATGCCCATGAACAT

A A V P M K I H M P M N M

1690 1700 1710 1720 1730 1740

GTTGCCCATGCTGACTAGCGACTCCACTGATGAGGAGGTAAGGTTACACAGACCGCCATG

L P M L T S D S T D E E

1750 1760 1770 1780 1790 1800

ATCTTTTGGTATCAGTTTGGACGAGGTAGCGTTGCTGTTTCACAATCTATGCCTCGCCAC

1810 1820 1830 1840 1850 1860

CCAGAACGGTTAATAGGGCCTATTTCTGGAGGATGAGTACCATTGTTGTAAGTAGGCGTT

1870 1880 1890 1900 1910 1920

TGGGCAAAACTGAACGTGCCCAAATTGTGTCCCAAGCTGGATCACAAAACATACAGGGCC

1930 1940 1950 1960 1970 1980

CAGTCCAAAACACATGAGCGTCTGAATCGGAGTCACCTATTTATTGTCACAACAAACCAA

1990 2000 2010 2020 2030 2040

CAATACGTGTCACAGCTAAATGTGAGATATAGATACGTTGATACTATAATGGTCCTATCC

2050 2060 2070 2080 2090 2100

TTAGATAATCTGTGTATATACCAATGACTGACATACTCAAAAAATCCCAGTCTTGATAAG

2110 2120 2130 2140 2150 2160

CTGATGTTGACTCATGTATAAAGTTCTTCATCAGTGAATTTTACTTTGGTTGTTGACGCC

2170 2180 2190 2200 2210 2220

CATCTGGTTGTGGGTTTCACCCTATTCTCACTTCTGTTTTGTTTTTTCTTTTTCTGTCAG

2230 2240 2250 2260 2270 2280

GGGGCATCCACCGAGGGCCCAAAAGCAGAAGGTATGATCTATCCATCTTTCTGTCTGTCT

G A S T E G P K A E

2290 2300 2310 2320 2330 2340

GTCTCTCCGTACTTCCACTCAACGCTCTCCTGCTGTAATTCGGCGAGAAGGATCAAATAT

2350 2360 2370 2380 2390 2400

CATGCGTTGTTTTCTTCCACGTTTTCTCCAAAGTTCAATCGATTACGTGTCCCCGCTCTC

2410 2420 2430 2440 2450 2460

CTCTCTAAAGTCACCAGGTTCTGACTCAGTCAACTGTTCGACCTAACTCTCCTTTCCTTA

2470 2480 2490

CTCCTCACCTCTCCCCCTCCGCTCACGTTCCCTTTCCTC
