# Supplementary material for: Major histocompatibility complex (MHC) fragment numbers alone – in Atlantic cod and in general - do not represent functional variability
Source: F1000Res. 2018 Sep 6;7:963. Originally published 2018 Jun 28. [Version 2] doi: 10.12688/f1000research.15386.2 (PMC6081975; doi:10.12688/f1000research.15386.2)
Supplement: Supplementary file 5 [file f1000research-7-17714-s0003.tgz › 21fbb7e8-d3d6-4249-8d31-daebce814a25.docx]

**Supplementary File 3:** Detailed explanation of Table 1

Main text Table 1 shows the lowest percentages of amino acid sequence identities between membrane-distal domains (α1+α2 for MHC I, α1 for MHC IIA, β1 for MHC IIB) of same category MHC molecules that we were able to find for reported sequences within the same species. Whether sequences are allelic or from different loci was not considered. In some species no genes for particular categories were found (black boxes), while in other instances only one seemingly intact gene sequence was found (1 sequence) or only pseudogenes were found (pseudogene). Only the membrane-distal domains were considered because they are important for antigen-fragment binding and for interaction with T cell receptors. For a division of teleost MHC class I sequences into lineages U, Z, L, P and S, see reference^1^. The category "U classical" only considers U lineage molecules in which at least six of the eight characteristic "key" amino acids for binding of the peptide ligand termini have been conserved^2^. Bony fishes do not have a conserved non-classical MHC class II lineage, and the emerging picture is that most teleost fish have classical MHC class II genes belonging to the DA lineage, plus often a number of non-classical genes which were derived from duplicates of the classical genes at some point in evolution and which are not consistently inherited^3^. The DA lineage separated from DB category sequences probably early in the teleost line, with the relationship between the various extant DB genes and DA genes not fully resolved^3^. The MHC class II lineage DE already separated from the DA/DB lineage before the separation between the ancestors of teleost fish and gar, and this very ancient lineage appears to have been lost in most teleost fish^3^. For finding the lowest percentages of amino acid sequence identities in the respective species, we started with the alignments of teleost MHC class I and II sequences that we showed previously^1,3^ in combination with sequence analysis software that determined the sequence identity percentages (GENETYX Version 12.0.3, Tokyo, function "Create %Identity Matrix" with setting "Gaps are NOT taken into account"). Then, by various blastp searches, we tried to find even more divergent sequences in the nonredundant sequence section of the NCBI database, and incorporated them into the same analysis for calculation of the amino acid identity percentages. Finally, for all seven fish species we retrieved all GenBank protein sequence database accessions which were found with the search word "MHC" (>300 for Atlantic cod, also hundreds for most of the other fishes), and separated them into the respective MHC categories based on their designations and our phylogenetic analysis (Muscle alignment followed by neighbor joining tree analysis including reference sequences). We then aligned those sequences with seemingly intact membrane-distal domains together with our other set of sequences (using GENETYX Version 12.0.3, Tokyo, function "Muscle Alignment"), and then determined the identity percentages as described above. For finding cod Z lineage sequences we analysed the sequence scaffolds published by Malmstrøm *et al.*^4^ as we did elsewhere^1^, and then determined the lowest percentage of amino acids between their α1+α2 sequences as done for the other sequences. Below follow the names plus GenBank accessions (between brackets) and/or relevant article references for the sequences whose comparisons gave the sequence identity percentages shown in the table. Although not necessarily canonical full-length MHC molecules, all the compared sequences might form intact molecules.

In reference^1^, estimations of the gene+pseudogene numbers in the haploid genomes of the several MHC categories in the seven here listed fish species were given. For the U lineage sequence numbers in zebrafish, salmon, medaka, fugu, stickleback and tilapia these estimates were 4, 7, 13, 8, 29, and 45, respectively (reference^1^). Although these estimations rely on the quality of the available information, there is little doubt that these fish species have considerably fewer U lineage sequences than the estimated 80-100 in cod (although that was analysed by a different method^4^). Despite the limited number of U lineage genes in zebrafish and salmon, in these species impressive allelic/haplotype variation can be found among the expressed classical U lineage sequences^1^, reflected in the here presented 40% and 47% amino acid sequence identities between their most divergent alleles.

Note: Our analyses were performed as part of an intended correspondence article submitted in its last form to Nature Genetics in February 2017. It then took 15 months before we received the first response, which was final and negative. We did not re-perform the analyses, although new fish MHC sequences keep being published. The reason for not updating our analyses is that it is a real lot of work, while we believe that essentially the data did not change.

**The sequence pairs whose distal domain identity percentages are shown in Table 1:**

**Zebrafish**

**MHC class I** U classical UBA (NM_131471) and UJA (NM_200406); see reference^5^

U all UIA (KC626502) and UMA (KR086347); see references^5,6^

Z mhc1ze (NM_194425) and ZJA (KC607869); see reference^7^

L LFA (NM_001327913) and (XM_001340377); see references^1,8^

**MHC class II** DA IIA D8.37_A1 (XM_005167233) and D8.37_A4 (NM_001005975); (two loci . on Chr. 8) see reference^3^

DA IIB D8.35_B2 (NM_001080633) and MHCII (NM_001005943) ; see references^3,9^

DB IIA D8.46_A (XM_694857) and D8.45_A2 (XM_009304226); (two loci on Chr.8) see reference^3^

DB IIB D18_B (XM_005159194) and D8.45_B2 (XM_009304199); (a locus on Chr.18 and a locus on Chr.8) see reference^3^

**Salmon**

**MHC class I** U classical UBA*1001 (AF504024) and UBA*4001 (JN897012); see reference^1^ and . site https://www.ebi.ac.uk/ipd/mhc/fish/index.html

U all UDA*0101 (FJ969490) and UHA1*0101 (FJ969489); see reference^1^

Z ZBA*0101 (GQ505860) and ZE*0101 (DQ099914); see reference^1^

L SAA*0101 (FJ969488) and (XM_014188252); see reference^1^

**MHC class II** DA IIA DAA*0801 (AY780910) and DAA*1201 (AY780914); see references^3,10^ and site <https://www.ebi.ac.uk/ipd/mhc/fish/index.html>

DA IIB DAB*0101 (X70166) and DAB*0601 (X70165); see references^3,11^ and site <https://www.ebi.ac.uk/ipd/mhc/fish/index.html>

DB IIA DCA (XM_014150051) and DDA (XM_014130418); see reference^3^

DB IIB DCB*0103 (KC316031) and DDB (XM_014130415); see reference^3^

DE IIA DEA*0101 and DEA*0102 (KC316033); see reference^3^

DE IIB DEB*0101 and DEB*0102 (KC316036); see reference^3^

**Medaka**

**MHC class I** U classical UBA*0206 (AB451005) and UGA*2001 (AB604103); see references^12,13^

*Note: Classical nature of medaka UGA has been questioned, but there is also only 53% identity between the recognized classical medaka sequences UBA*0206 (BAJ07301) and UBA*0201 (BAB83850) (references^11,12^)*

U all UEA*0201 (BA000027) and UHA*2201 (AB604115); see references^1,13^

Z OL17 (XM_011493562) and (XM_011480916); see reference^1^

**MHC class II** DA IIA DCA*21 (JQ743250 ) and DFA*21 (JQ743256); see references^3,14^

DA IIB DAB*206 (JQ743290) and DCB (AB033214); see references^3,14^

DB IIA DDA*21 (JQ743252) and DEA*11 (JQ743255); see references^3,14^

DB IIB DDB1*21 (JQ743261) and DEB*21 (JQ743264); see references^3,14^

**Fugu**

**MHC class I** U classical TR3 (XM_011609132) and I103 (CAC13116); see references^1,15^

U all TR7 and TR13; see reference^1^ (sequences can be retrieved from Text S2 in that article)

P TR26 and TR29; see reference^1^ (sequences can be retrieved from Text S2 in that article)

**MHC class II** DA IIA (XM_011619962) and (XM_011621535); see reference^3^

DA IIB (XM_011620545) and (XM_003976765); see reference^3^

**Stickleback**

**MHC class I** U classical GA1 and Gaac-UAA*01 (ABN14358); see reference^1^ (sequence . GA1 can be retrieved from Text S2 in that article) and reference^1^

U all GA12 and GA16; (two loci on linkage group X) see reference^1^ (sequences can be retrieved from Text S2 in that article)

**MHC class II** DA IIA GVII_A and DAA*01 (AY713945); see reference^3^ (sequence GVII_A can be . retrieved from Text S1 in that article) and reference^17^

DA IIB GVII_B and DBB (AY713945); see reference^3^ (sequence GVII_B can be retrieved from Text S1 in that article) and reference^17^

**Tilapia**

**MHC class I** U classical ON1 (XM_005457584) and ON20 (XM_013265954); see reference^1^

U all ON32 and ON45 (XM_013267852); see reference^1^ (sequences can be retrieved from Text S2 in that article)

Z ON22 (XM_005463504) and ON24 (XM_005464317); see reference^1^

**MHC class II** DA IIA O22_A1 (XM_005460587) and O745_A (XM_005465903); see . reference^3^

DA IIB O31_B1 (XM_013274958) and MHC II beta (JN983489); see reference^3^

DB IIA O29_A3 (XM_003451009) and O57_A; see reference^3^ (sequences can be retrieved from Text S1 in that article)

DB IIB O9_B (XM_005471522) and O57_B; see reference^3^ (sequences can be retrieved from Text S1 in that article)

**Cod**

**MHC class I** U classical clone 0122 (KF033069) and clone 0101 (JX567634); see references^4,18^

U all clone 0122 (KF033069) and clone 0101 (JX567634); see references^4,18^

Z Scaffold_CAEA020024072_Z and Scaffold_CAEA020024073_Z; see reference^4^

**References used in this supplementary file**

1. Grimholt, U., Tsukamoto, K., Azuma, T., Leong, J., Koop, B.F. & Dijkstra, J.M. A comprehensive analysis of teleost MHC class I sequences. *BMC Evol. Biol.* **15**, 32 (2015).

2. Hashimoto, K., Okamura, K., Yamaguchi, H., Ototake, M., Nakanishi, T. & Kurosawa, Y. Conservation and diversification of MHC class I and its related molecules in vertebrates. *Immunol. Rev.* **167**, 81-100 (1999).

3. Dijkstra, J.M., Grimholt, U., Leong, J., Koop, B.F., Hashimoto, K. Comprehensive analysis of MHC class II genes in teleost fish genomes reveals dispensability of the peptide-loading DM system in a large part of vertebrates. *BMC Evol. Biol.* **13**, 260 (2013).

4. Malmstrøm, M., Matschiner, M., Tørresen, O.K., Star, B., Snipen, L.G., Hansen, T.F., Baalsrud, H.T., Nederbragt, A.J., Hanel, R., Salzburger, W., Stenseth, N.C., Jakobsen, K.S. & Jentoft, S. Evolution of the immune system influences speciation rates in teleost fishes. *Nat. Genet.* **48**, 1204-1210 (2016).

5. McConnell, S.C., Restaino, A.C. & de Jong, J.L. Multiple divergent haplotypes express completely distinct sets of class I MHC genes in zebrafish. *Immunogenetics* **66**, 199-213 (2014).

6. Dirscherl, H. & Yoder, J.A. A nonclassical MHC class I U lineage locus in zebrafish with a null haplotypic variant. *Immunogenetics* **67**, 501-513 (2015).

7. Dirscherl, H. & Yoder, J.A. Characterization of the Z lineage Major histocompatability complex class I genes in zebrafish. *Immunogenetics* **66**, 185-98 (2014).

8. Dirscherl, H., McConnell, S.C., Yoder, J.A. & de Jong, J.L. The MHC class I genes of zebrafish. *Dev. Comp. Immunol.* **46**, 11-23 (2014).

9. Ono, H., Klein, D., Vincek, V., Figueroa, F., O'hUigin, C., Tichy, H. & Klein, J. Major histocompatibility complex class II genes of zebrafish. *Proc. Natl. Acad. Sci. U.S.A.* **89**, 11886-11890 (1992).

10. Consuegra, S., Megens, H.J., Leon, K., Stet, R.J. & Jordan, W.C. Patterns of variability at the major histocompatibility class II alpha locus in Atlantic salmon contrast with those at the class I locus. *Immunogenetics* **57**, 16-24 (2005).

11. Stet, R.J., de Vries, B., Mudde, K., Hermsen, T., van Heerwaarden, J., Shum, B.P. & Grimholt, U. Unique haplotypes of co-segregating major histocompatibility class II A and class II B alleles in Atlantic salmon (Salmo salar) give rise to diverse class II genotypes. *Immunogenetics* **54**, 320-331 (2002).

12. Nonaka, M.I. & Nonaka, M. Evolutionary analysis of two classical MHC class I loci of the medaka fish, Oryzias latipes: haplotype-specific genomic diversity, locus-specific polymorphisms, and interlocus homogenization. *Immunogenetics* **62**, 319-32 (2010).

13. Nonaka, M.I., Aizawa, K., Mitani, H., Bannai, H.P. & Nonaka, M. Retained orthologous relationships of the MHC Class I genes during euteleost evolution. *Mol. Biol. Evol.* **28**, 3099-3112 (2011).

14. Bannai, H.P. & Nonaka, M. Comprehensive analysis of medaka major histocompatibility complex (MHC) class II genes: implications for evolution in teleosts. *Immunogenetics* **65**, 883-895 (2013).

15. Clark, M.S., Pontarotti, P., Gilles, A., Kelly, A. & Elgar, G. Identification and characterization of a beta proteasome subunit cluster in the Japanese pufferfish (Fugu rubripes). *J. Immunol.* **165**, 4446-4452 (2000).

16. Schaschl, H. & Wegner, K.M. Contrasting mode of evolution between the MHC class I genomic region and class II region in the three-spined stickleback (Gasterosteus aculeatus L.; Gasterosteidae: Teleostei). *Immunogenetics* **59**, 295-304 (2007).

17. Reusch, T.B., Schaschl, H. & Wegner, K.M. Recent duplication and inter-locus gene conversion in major histocompatibility class II genes in a teleost, the three-spined stickleback. *Immunogenetics* **56**, 427-37 (2004).

18. Star, B. *et al.* The genome sequence of Atlantic cod reveals a unique immune system. *Nature* **477**, 207-210 (2011).
