# Supplementary material for: Major histocompatibility complex (MHC) fragment numbers alone – in Atlantic cod and in general - do not represent functional variability
Source: F1000Res. 2018 Sep 6;7:963. Originally published 2018 Jun 28. [Version 2] doi: 10.12688/f1000research.15386.2 (PMC6081975; doi:10.12688/f1000research.15386.2)
Supplement: Peer review history

# Peer review: Referee response for version 2

**DOI:** 10.5256/f1000research.17714.r38018

**Published:** 2018-09-17

**Authors:** Anthony B. Wilson

**Affiliations:** 1 Department of Biology, Brooklyn College CUNY, New York, NY, USA; 2 The Graduate Center, City University of New York, New York, NY, USA

No further amendments are required.  As highlighted by Dr. Kulski in his review, I'm hopeful that this debate will prompt the authors and others to tackle this fascinating evolutionary question!

I have read this submission. I believe that I have an appropriate level of expertise to confirm that it is of an acceptable scientific standard.

# Peer review: Referee response for version 2

**DOI:** 10.5256/f1000research.17714.r38019

**Published:** 2018-09-13

**Authors:** Brian Dixon

**Affiliations:** 1 Department of Biology, University of Waterloo, Waterloo, ON, Canada

The additional analysis and explanation of the basis for their comments strengthens this article.

I have read this submission. I believe that I have an appropriate level of expertise to confirm that it is of an acceptable scientific standard.

# Peer review: Referee response for version 2

**DOI:** 10.5256/f1000research.17714.r38020

**Published:** 2018-09-07

**Authors:** Jerzy K. Kulski

**Affiliations:** 1 Department of Molecular Life Science, Division of Basic Medical Science and Molecular Medicine, Tokai University School of Medicine, Isehara, Japan; 2 School of Psychiatry and Clinical Neurosciences, The University of Western Australia , 35 Stirling Highway Crawley, WA 6009, Australia

No further comments were required.

I have read this submission. I believe that I have an appropriate level of expertise to confirm that it is of an acceptable scientific standard.

# Peer review: Referee response for version 1

**DOI:** 10.5256/f1000research.16766.r36540

**Published:** 2018-08-06

**Authors:** Jerzy K. Kulski

**Affiliations:** 1 Department of Molecular Life Science, Division of Basic Medical Science and Molecular Medicine, Tokai University School of Medicine, Isehara, Japan; 2 School of Psychiatry and Clinical Neurosciences, The University of Western Australia , 35 Stirling Highway Crawley, WA 6009, Australia

The correspondence by Dijkstra & Grimholt ^1^^ ^provides critical concerns about a publication by Malmstrøm et al in Nature Genetics in October 2016 ^2^, concluding that their new model of MHC class I evolution, reflected in their title “Evolution of the immune system influences speciation rates in teleost fish”, is unsubstantiated. I concur with their three main criticisms “(1) that the authors did not provide sufficient evidence for presence or absence of intact functional MHC class I or MHC class II system genes, (2) that they did not discuss that an MHC subpopulation gene number alone is a very incomplete measure of MHC variance, and (3) that the MHC system is more likely to reduce speciation rates than to enhance them.”

 All three critical points are well founded and stand-alone without too much need for further support. However, I have added the following 14-point commentary for Dijkstra & Grimholt ^1^, Malmstrøm et al (2016) ^2^ and others to consider and elaborate on if they would like to because they are important concerns in the field of MHC genomics, biological function and evolution.

 1. According to Dijkstra & Grimholt ^1^^ ^“the MHC counting strategy by Malmstrøm et al. ^2^^ ^should be deemed incomplete and far too simplistic. For their number determination Malmstrøm et al. ^2^^ ^solely relied on estimation of U plus Z lineage genomic **α**3 exon fragment numbers, despite that the typical “birth and death” mode of MHC evolution can produce many pseudogenes.”  I agree that this is a major problem with the Malmstrøm et al (2016) ^2^^ ^paper, one that also omits the other categories of the MHC I such as the L, S and P lineages that might contribute to a much large number of MHC I-like genes. In this regard, it seems that Malmstrøm et al. ^2^^ ^have taken only the genomic exon fragment numbers of the MHC I U and Z lineages to represent the entire immune system of their title.

 2. Dijkstra & Grimholt ^1^are also right to point out that low coverage sequencing by next generation sequencing (NGS) can result in the artifactual loss of genes that in turn can lead to misleading or incorrect conclusions when counting for gene copy numbers or looking for gene losses. Malmstrøm et al (2016) ^2^^ ^sequencing coverage was 9 to 39x and they recovered only about 75% of the conserved eukaryotic genes. Therefore, in this situation, the *de novo *low coverage sequencing data should not have been used as evidence for the absence of genes from the genome without providing a properly organized high coverage map of genomic assemblies to show where the sequences are missing in the genomes. The reviewers and editors of the Malmstrøm et al (2016) ^2^paper should have been aware of this basic problem of using low coverage NGS, particularly with respect to looking for a few needles in a haystack. For a better understanding of the advantages and disadvantages of MHC genotyping and haplotyping by NGS see the review by Shiina et al (2016) ^3^.

 3. Malmstrøm et al (2016) ^2^^ ^reported that there was no overall correlation between the combined MHC copy numbers and the HOX gene copy numbers that were used as a control. As already pointed out by Dijkstra & Grimholt ^1^, the U and Z genes should have been analysed separately. Nevertheless, it would have been interesting to see how these duplicated HOX development genes, which also have been implicated in driving speciation, compared with the properly classified duplicated MHC class I adaptive immune genes (separate Z, X, L, S and P lineages) at the classical and non-classical level in Fig 3 during speciation rate simulations ^2^. ^^In addition, it appears from Fig S3 that Malmstrøm et al (2016) ^2^  might have missed an inverse relationship between MHC & HOX for the MHC copy numbers up to 25 and direct correlation between MHC & HOX for the MHC copy number from 25 to 50.

 4. It seems absurd to count up only the short sequences of a3 fragments from a low coverage sequence library and extrapolate the numbers counted in Fig 2b to reconstruct an artificial model for duplicated MHC gene copies influencing speciation or evolution without first knowing their categories (classical versus non-classical presentations), functions, overall structure and coding ability, transcriptional activity and genomic locations.  Malmstrøm et al (2016) ^2^^ ^provided no properly organized genomic assemblies or genomic gene maps and no information about genomic distribution of the MHC I or II sequenced fragments or the duplication mechanisms involved. If they had done so, they might have added important information to better assess and place the threshold MHC I copy numbers and gene distributions into some sort of genomic perspective ^4^. More reliable models for the evolution of MHC class I genomic duplications might be achieved by providing duplication gene maps and the phylogenic relationships of the duplicated gene sequences showing likely duplication mechanisms, where and how these genes are located relative to each other, and how the genomic structures have changed in a comparative analysis between species. See Kulski et al. (2004) ^5^^ ^for an example of one such duplication model. Mapping with phylogeny is a more informative approach than just constructing phylogenetic trees using one or more single exonic sequences from a limited number of each species and then claiming that the changes in copy numbers influence the speciation rates of almost the entire number of extant fish species. Perhaps, the Malmstrøm et al (2016) ^2^ low coverage sequence libraries could still be used effectively to reconstruct full-length gene structures and targeted genomic regions that harbour multiple copies of the MH(C) genes in a comparative analysis.

 5. The multiple-optima Ornstein Uhlenbeck (OU) model ^6^. 

 (a) According to Malmstrøm et al (2016) ^2^^ ^the multiple-optima OU model vastly outperformed alternatives such as Brownian motion, white noise, single-peaked OU and early-burst models. This find­ing corroborated their hypothesis that MHC I copy number evolution is characterized by selection toward intermediate optima, resulting from a tradeoff between detection and elimination of pathogens. Presumably, the authors preferred the OU multiple-optima model to the other models because it supported rather than falsified their hypothesis. Of course, this highly artificial computing model did not detect a tradeoff between detection and elimination of pathogens, this would be the authors’ own biological hypothesis and bias.  The OU model intrinsically sets optima (biases) according to its built-in algorithm ^6^, and this is one of the main objections to the use of this prediction model. The OU model artificially generates bias because its purpose is to find the trait optimum that stabilizes selection ^6^. The misguided conclusion by Malmstrøm et al (2016) ^2^^ ^in using the OU model is that the trait optimum influences speciation. 

 (b) Interestingly, Malmstrøm et al (2016) ^2^^ ^did not directly test the opposing hypothesis that the MHC I copy number evolution is not characterized by selection toward intermediate optima, and does not result from a tradeoff between detection and elimination of pathogens. Possibly, their best control in this regard was the simple Brownian model that did not work as well for them as the multiple-optima OU model that has extra parameters such as the addition of an overall optimum trait value to which all lineages are attracted. Other evolutionists often prefer the Brownian model for the reason that it is a simple, neutral model without the added bias of creating optimum trait value.

 ( c ) The OU multiple-optima model is not a fool-proof algorithm, and a number of evolutionists believe that it can be an unreliable or misleading model. According to Cooper et al (2016) ^7^, although widely used, the properties of the OU model, and other direct extensions of the Brownian model, are poorly understood leading to the potential for inappropriate use and misinterpretation of results. In particular, Cooper et al (2016) ^7^ used computer simulation studies to demonstrate that the single peaked OU model error rates are unacceptably high when tree size is small (< 200 species tips), when likelihood ratio tests or Akaike information criterion (AICc) are used to select the best model, and when measurement errors are introduced into the data. They also showed that when the alpha parameter of trait evolution was extremely small (<1) in the OU model it was indistinguishable from Brownian motion, and as the alpha value became larger it favoured OU prediction models, until the larger values of alpha were indistinguishable from white noise and it was therefore independent of phylogeny. The alpha values for the Malmstrøm et al (2016) ^2^^ ^model selection analysis were markedly less than one (Supplementary Table 13), suggesting that they could have accepted the Brownian model over the OU model as the better model fit. 

 6. The BiSSE threshold model. 

 Malmstrøm et al (2016) ^2^^ ^carried out binary state speciation and extinction (BiSSE) analysis to estimate differences in diversification rate between lineages with high and low MHC I copy numbers. They found that diver­sification rates based on correlation estimates differed most when the threshold was placed between 20 and 25 copies (Fig. 3c). With a threshold in this range, the model with two separate speciation rates for lineages with high and low copy numbers was statistically better supported than a model with a single speciation rate parameter.  On this basis, they concluded that, ‘These results suggest that the influence of MHC I genes on speciation rates is stronger in species that have already evolved at least 20 copies.’ In comparison, the number of MHC I gene copy numbers in humans (excluding haplotype differences) is approximately 18 genes; 6 classical and non-classical MHC I genes, 5 CD1 genes, and 5 PHFZ genes (MICA, MICB, MR1, HFE, Zn-A2-GP, *etc*). Thus, in comparison to some fish species, humans are diversifying along very nicely as a ‘diversified’ species approaching the ‘magical’ threshold of between 20 and 25 MHC I copies. 

 It is noteworthy that Maddison et al (2007) ^8^^ ^highlighted the following assumptions that need to be taken into account when using their BiSSE package. For the BiSSE model analysis none of the characters associated with speciation rates can be said to be causing or influencing evolution, even if Maddison et al (2007) ^8^^ ^write, ‘the correct conclusion given a significant result using our method is that the character examined or a codistributed character *appear to be controlling diversification rates.*’ At best, the binary character state is an association, at worse a misleading one. Maddison et al (2007) ^8^^ ^provided the following cautions and assumptions about the likelihood of the BiSSE (binary-state speciation and extinction) model:

 (a) the transitions happen instantaneously over the time scales considered (i.e., ignore periods of time during which a species is polymorphic). 

 (b) these events are independent of one another; in particular, the character state change does not, in and of itself, cause speciation (or vice versa).

 (c) an accurate rooted phylogenetic tree with branch lengths is known (the "inferred tree") and the character state is known for each of the terminal taxa. 

 (d) the tree is assumed complete: all extant species in the group have been found and included.  

 (e) all terminal taxa are contemporaneous, and the tree is ultrametric (i.e., the total root-to-tip distance is the same for all tips). 

 I’m not convinced that Malmstrøm et al (2016) ^2^^ ^considered or accepted these constraining assumptions when using BiSSE modeling.

 7. Speciation and diversification rates. 

 (a) In the study by Santini et al (2009) ^9^, the speciation rates within the Percomorpha clade were calculated to be at least ten times greater than in the Gadiformes order. Yet, according to Malmstrøm et al (2016) ^2^, there were fewer than 20 copies of the U genes for each of the 5 species in the Perciformes clade, compared to more than 20 copies and up to 100 copies of the U genes in 16 of 30 species in the Gadiformes order (Fig 2b, Malmstrøm et al 2016 ^2^). The use of only 5 species in the Perciformes order is a gross underestimate of the ten thousand or more species found in that order ^9^. Moreover, in the Gadiformes order, there were closely related species (n=16) with > 20 U genes and different groups of closely related species (n=14) with < 20 U genes. Again, the number of species that were sequenced in the Gadiformes order is grossly under-represented. The Gadiformes comprises 10 families and more than 600 species ^9^, whereas Malmstrøm et al (2016) ^2^^ ^sequenced only 27, i.e.,  27 x 100/600 = 2,700/600 = 4.7% analysis of extant sequence, a percentage that is simply not good enough to support their extravagant conclusions.  Is < 5% of the 600 species really representative of the Gadiformes. Malmstrøm et al (2016) ^2^^ ^have to be more temperate with their conclusions using such a small representative sample.There are clear inconsistencies with MHC I a3 fragment copy numbers in the Gadiformes order. The MHC I a3 frag copy numbers are low (<20) for Moridae and M. occidentalis in Macrourinae, and for Phycinae, Lotinae, and three species in Gadinae. Five species of Gadinae have between 20 and 40 copies. On the other hand, Bregmacerotidae, Merlucciidae, Melanonidae, Muraenolepidodae, and Trachyrincinae have MHC I a3 fragment copy numbers between 50 and 100. The threshold levels (20 to 25) are all over the place. Moreover, the lineage, genomic block duplication and hitchhiking (linkage) effects on MHC gene duplications (8 to >100 copies) in the Gadiformes have not been taken into account in the analysis of speciation rates (Fig 3, Malmstrøm et al  2016 ^2^), and, therefore, make the entire analysis unreliable.

 (b) “Diversification rate analyses were calculated on the basis of the time-calibrated phylogeny and counts of species richness in each of the 37 mutually exclusive clades of teleost fishes" ^2^; mostly from the Gadiformes order. The MHC I speciation model of Malmstrøm et al (2016) ^2^^ ^appears contradictory for the Perciformes (10,033 species) that have speciation rates 18 times greater than Gadiformes (555 species) ^9^^ ^and yet the MHC I a3 fragment copy numbers are at least two times lower in Perciformes than Gadiformes (Fig 2b, Malmstrøm et al  2016 ^2^). Also, the Anabantiformes have 252 species – a species rate 40 times lower than Gadiformes ^9^ and yet their MHC I a3 fragment copy numbers are at least two times higher than in Perciformes.

 (c) Considering that there are more than 29,000 species of teleost fishes ^9^, a highly limited analysis by Malmstrøm et al (2016) ^2^^ ^of using a sample group of less than 0.2% of the available extant species cannot be considered to be statistically, taxonomically or biologically significant or sufficiently reliable to conclude that, “Evolution of the immune system influences speciation rates in teleost fish” ^2^. ^^What does a species half-life of 25 million years mean in the context of 29,000 species of teleost fishes? If the multiple-regime OU model is wrong, highly biased or misinterpreted then does it validate or support the overall hypothesis of Malmstrøm et al (2016) ^2^? Also, what does an optimal trait actually mean in the context of 29,000 species? If, a suboptimal number of MHC I copies are detrimental to a species, then how have divergent species managed to survive for so long with a half-life of 25 million years of adaptation?  Also, if, as Malmstrøm et al (2016) ^2^^ ^say, ‘Such gene family expansions may promote biological diversification by introducing new raw genetic material, potentially resulting in sub- or neofunctionalization and thus novel immunological pathways.’, then which of the non-optimal (greater than or less than the threshold of 20 -25 copies) MHC I genes are detrimental to the species? In this regard, there must be a gradation of functionally good and bad MHC I genes as their copy numbers approach the threshold (optimally good) and then deteriorate beyond it. Is this assumption of a MHC I copy number functional trait value as a quantitative marker of speciation at all testable?

 8. In their discussion, Malmstrøm et al (2016) ^2^^  ^referenced the hypothesis of T cell depletion and hybrid fitness by Eizaguirre et al (2009) ^10^^ ^and concluded that,"Our analyses identify this threshold at 20–25 MHC I copies, suggesting that the effect of T cell depletion on hybrid fitness becomes more pronounced in this range and that this might affect mate choice in species with copy numbers above this threshold, promoting inbreeding and reinforcement."  Eizaguirre et al (2009) ^10^^ ^suggested that, “Super-optimal individual MHC diversity should be a common disadvantage for species hybrids in vertebrates, resulting in elevated parasite loads.”  In this regard, if high copy numbers of the MHC class I genes leads to hybridization and loss of the immune system as inferred by Eizaguirre et al (2009) ^10^, then this more than likely would lead to extinction of populations and species. Extinction would be the most extreme and bizarre form of immune system influence on speciation rates. Furthermore, it is extremely speculative for Malmstrøm et al (2016) ^2^^ ^to say that high copy numbers of the MHC class I genes with copy numbers above the threshold of 20 to 25 copies, promotes inbreeding and reinforcement, because, in fact, there is no such evidence for it. A more reasonable hypothesis is that high copy numbers of linked MHC class I genes, such as in rhesus macaque, or the mouse ^11^, or the cod ^12^, might benefit the species to better adapt to microbial inhabitants in a greater variety of geographical environments, although the evidence for this is tenuous as well. Despite ongoing debates, the selective advantage of MHC diversity in host-pathogen coevolution might not be easily resolved (at the macroevolution level) because of the constant number of insults by large numbers of pathogens in the life-time of an individual organism, population or a species and the arms race or Red Queen effect. Studies on extant species will always discover an example of a pathogen associated with a polymorphic MHC gene that might favour selective advantage for host-pathogen coevolution, whereas the pathogen that caused the extinction of a species is rarely or never found. To conclude that the immune system (that is, different copy numbers of the class I MHC genes ^2^) influences speciation rates, it would have to be shown that the immunity gene products can commonly create reproductive barriers or genetic incompatibilities among populations that permit the maintenance of the genetic and phenotypic distinctive­ness of these populations in geographical proximity ^13^; and this was not done ^2^.

 9. Malmstrøm et al (2016) ^2^^ ^did not provide any reliable evidence to support their speculation that evolution of the immune system influences speciation rates in teleost fish or that increas­ing MHC I diversity facilitates speciation ^7^.  Instead, Malmstrøm et al (2016) ^2^^ ^used their limited data and analyses using speculative models to jump to highly unsupported conclusions and quickly position the cart before the horse. Dijkstra & Grimholt ^1^ pointed out that the Malmstrøm et al (2016) ^2^^ ^title “Evolution of the immune system influences speciation rates in teleost fish”, is unsubstantiated, and that their hypothesis seems to be “the wrong way around”. It should have been, “Speciation (rate) influences the evolution of the immune system in teleost fish.” Or, “Speciation rates are associated with diversity of MHC class I genes in teleost fishes”, which perhaps is too obvious and underwhelming. This is not simply the chicken or the egg causality dilemma; in fact, the change in title is better supported by the literature and the established theories of MHC genomic evolution in vertebrates ^4^. However, because it is less “sexy” and controversial than the original title, it might not have been so readily published. 

 10. A large number and variety of genome-wide gene duplications have been associated with speciation ^13^, that is, genomic gene duplications are not limited to only class I MHC genes. If MHC I gene duplications effect or affect speciation, how do the other hundreds of gene duplications contribute to speciation rates? Also, do sequence variants or mutations in non-duplicated genes have any influence on speciation rates? It seems absurd to pick on only one group of gene duplications (e.g., MHC class I genes ^2^) as those that are responsible for speciation and ignore all the others as an inconvenience. For example, a relatively recent comparative genomic study revealed how genomes change with speciation in an examination of genomes from five cichlid fish species, an ancestral lineage from the Nile, and four species from the East Africa lakes, Tanganyika, Malawi, and Victoria ^14^. Compared to the ancestral Nile lineage, the East African cichlid genomes had many alterations in regulatory elements, accelerated evolution of protein-coding elements in genes for pigmentation, an excess of gene duplications, and other distinct features that affect gene expression associated with transposable element insertions and novel microRNA. Each species also contains a reservoir of mutations different from the other species ^14^. Much of the diversity between the cichlid fish species evolved in a nonparallel manner often rapidly due to sexual selection and genetic conflicts between males and females or between different regions of the genome at a regulatory level ^14^^ ^rather than by the slower and weaker forces of classical natural selection ^13^. If sexual selection and genetic conflict at the genomic regulatory level are the prime movers of speciation rate, it is difficult to conclude that the variable diversity of a few MHC gene copies are responsible for speciation as well as for the many other associated genomic changes associated with speciation.

 11. Malmstrøm et al (2016) ^2^^ ^informed us in the introduction section of their publication that "Our results highlight the plasticity of the vertebrate adaptive immune system and support the role of MHC genes as ‘speciation genes’, promoting rapid diversification in teleost fishes."  MHC class I gene copy number variability occurs across many different species, families, orders and domains. Because there is such enormous variability in MHC class I gene copy number for hundreds ^4^^ ^or possibly even thousands of different chordate species, it is not possible to conclude meaningfully that the expansion of MHC class I genes provides an undefined advantage of one species over another. For example, the great apes (humans, chimpanzees, gorillas and orangutans) have about six functional MHC class I genes, whereas the old and new world monkeys often have up to 15 or more ^4^^,^^11^. Is this evidence that the MHC class I genes influence the rate of speciation in primates? And if so, what does that really mean in the whole scheme of things? How do the species with low copy numbers of MHC class I genes survive so well over millions of years without the presence of another 90 to 100 copies of MHC class I genes? This question is often neglected, and yet it is important for a better understanding of the function and evolution of MHC genes between and within the vertebrate species. 

 12. Taxonomic and lineage markers.

 Mutations, indels and duplications drive diversity and evolution. However, most mutated genes within species and their families do not create or influence speciation rates in the sense that Malmstrøm et al (2016) ^2^^ ^use the term, ‘speciation genes’. In comparative genomics and their sequence relationships between different species, most genomic sequences range between newly derived genes and the ultraconserved or the essential core coding and noncoding genes with varying amounts of sequence differences. Some genic and nongenic sequences such as the MHC genes and retrotransposons are highly polymorphic and therefore are useful taxonomic markers at the individual, population, species and broader lineage levels. The MHC gene sequences clearly are one of these useful taxonomic or lineage markers along with olfactory receptors, immunoglobulins, globins, HOX, TOLLs, KIRs, mitochondrial DNA, ribosomal RNA sequences and thousands of others that can be used comparatively in the phylogeny to undertake an examination of the accuracy and reliability of current taxonomical rankings and sister lineages. However, because many thousands of coding and noncoding genes (or sequences) are variants (polymorphic) or vary in copy numbers, we do not immediately or easily imply that all or some of them are responsible for speciation without providing further concrete evidence. This kind of extrapolation without the burden of proof is absurd and wrong. Similarly, to say that the polymorphisms demonstrate natural selection as if natural selection was a biological or molecular mechanism is meaningless without showing experimentally how these polymorphisms benefit or disadvantage the organism over all the other different polymorphisms. 

 13. On the basis of either *a priori *or *a posteriori *reasoning, the immune system obviously affects the wellbeing of individuals and populations, but whether it can be extrapolated to speciation events and speciation rates remains highly dubious and most probably unlikely. It seems too farfetched to blame MHC class I genes with high copy numbers over threshold levels of promoting inbreeding and reinforcement ^2^^ ^because this in turn could create hybrid inviability or sterility resulting in postzygotic isolation. Although the population conditions in many models of rapid speciation do favour inbreeding and/or hybridization ^13^, none of the teleost species tested by Malmstrøm et al (2016) ^2^^ ^were shown to be either inbreeding or in postzygotic isolation. The factors responsible for either prezygotic or postzygotic isolation are likely to be independent of the adaptive immune system, although zealots might argue otherwise. Hybridization between diverging lineages in post-zygotic reproductive isolation can trigger genome instability. For most animals without an adaptive immune system and for plants without a MHC, speciation depends on the shrinkage, expansion and equilibrium (e.g., aneuploidization and dysploidy) of the genome and the containment and functionality of all the essential genomic information to develop an optimal balance between stability and plasticity within the organism in order to first survive and then propagate and expand itself as a new species ^13^. In those rare and ‘traumatic’ transitional situations, there is no need for particular ‘speciation’ genes such as variable copies of the class I MHC genes to influence speciation. The rarely observed transition from population ‘trauma’ to a new speciation event depends on an array of totally different factors for creating postzygotic isolation events including interbreeding between semi-isolated populations and an elaboration on the existence of stress-induced changes in chromosomal and ploidy integrity both in hostile and non-hostile environments.

 14. Finally, Malmstrom et al (2016) ^2^^ ^admirably sequenced 66 teleost species by a next generation sequencing method and identified an array of MHC I and MHC II exonic fragments for phylogenetic and speciation analysis using the multiple-regime OU model to predict the optimal MHC I copy number as an evolutionary trait optimum affecting speciation. However, the conclusions of the paper by Malmstrom et al (2016) ^2^^ ^especially for the MHC I gene copy numbers are unreliable because they are based on far too many assumptions, speculations, contradictions, incomplete or missing data and unproven predictive models with little or no empirical evidence in support. Nevertheless, their simple, but controversial hypothesis is published, and now it is up to them and others to test its validity and "consider plausible alternative hypotheses in a firm hypothesis-testing framework in which alternative hypotheses make clear [and sensible] predictions of emerging patterns that can be unambiguously associated with particular models." ^7^

I have read this submission. I believe that I have an appropriate level of expertise to confirm that it is of an acceptable scientific standard.

## References

[1] DijkstraJGrimholtU: Major histocompatibility complex (MHC) fragment numbers alone – in Atlantic cod and in general - do not represent functional variability. *F1000Research*.2018;7: 10.12688/f1000research.15386.1 10.12688/f1000research.15386.1 PMC608197530135730

[2] MalmstrømMMatschinerMTørresenOKStarBSnipenLGHansenTFBaalsrudHTNederbragtAJHanelRSalzburgerWStensethNCJakobsenKSJentoftS: Evolution of the immune system influences speciation rates in teleost fishes. *Nat Genet*.48(10) : 10.1038/ng.3645 1204-10 10.1038/ng.3645 27548311

[3] ShiinaTSuzukiSKulskiJ: MHC Genotyping in Human and Nonhuman Species by PCRbased Next-Generation Sequencing.2016; 10.5772/61842 10.5772/61842

[4] KulskiJKShiinaTAnzaiTKoharaSInokoH: Comparative genomic analysis of the MHC: the evolution of class I duplication blocks, diversity and complexity from shark to man. *Immunol Rev*.2002;190:95-122 1249300910.1034/j.1600-065x.2002.19008.x

[5] KulskiJKAnzaiTShiinaTInokoH: Rhesus macaque class I duplicon structures, organization, and evolution within the alpha block of the major histocompatibility complex. *Mol Biol Evol*.2004;21(11) : 10.1093/molbev/msh216 2079-91 10.1093/molbev/msh216 15269276

[6] HansenTF: STABILIZING SELECTION AND THE COMPARATIVE ANALYSIS OF ADAPTATION. *Evolution*.1997;51(5) : 10.1111/j.1558-5646.1997.tb01457.x 1341-1351 10.1111/j.1558-5646.1997.tb01457.x 28568616

[7] CooperNThomasGHVendittiCMeadeAFreckletonRP: A cautionary note on the use of Ornstein Uhlenbeck models in macroevolutionary studies. *Biol J Linn Soc Lond*.2016;118(1) : 10.1111/bij.12701 64-77 10.1111/bij.12701 27478249PMC4949538

[8] MaddisonWPMidfordPEOttoSP: Estimating a binary character's effect on speciation and extinction. *Syst Biol*.2007;56(5) : 10.1080/10635150701607033 701-10 10.1080/10635150701607033 17849325

[9] SantiniFHarmonLJCarnevaleGAlfaroME: Did genome duplication drive the origin of teleosts? A comparative study of diversification in ray-finned fishes. *BMC Evol Biol*.2009;9: 10.1186/1471-2148-9-194 194 10.1186/1471-2148-9-194 19664233PMC2743667

[10] EizaguirreCLenzTLTraulsenAMilinskiM: Speciation accelerated and stabilized by pleiotropic major histocompatibility complex immunogenes. *Ecol Lett*.2009;12(1) : 10.1111/j.1461-0248.2008.01247.x 5-12 10.1111/j.1461-0248.2008.01247.x 19087108

[11] ShiinaTBlancherAInokoHKulskiJK: Comparative genomics of the human, macaque and mouse major histocompatibility complex. *Immunology*.2017;150(2) : 10.1111/imm.12624 127-138 10.1111/imm.12624 27395034PMC5214800

[12] ParhamP: How the codfish changed its immune system. *Nature Genetics*.2016;48(10) : 10.1038/ng.3684 1103-1104 10.1038/ng.3684 27681288

[13] SeehausenOButlinRKKellerIWagnerCEBoughmanJWHohenlohePAPeichelCLSaetreGPBankCBrännströmABrelsfordAClarksonCSEroukhmanoffFFederJLFischerMCFooteADFranchiniPJigginsCDJonesFCLindholmAKLucekKMaanMEMarquesDAMartinSHMatthewsBMeierJIMöstMNachmanMWNonakaERennisonDJSchwarzerJWatsonETWestramAMWidmerA: Genomics and the origin of species. *Nat Rev Genet*.2014;15(3) : 10.1038/nrg3644 176-92 10.1038/nrg3644 24535286

[14] BrawandDWagnerCELiYIMalinskyMKellerIFanSSimakovONgAYLimZWBezaultETurner-MaierJJohnsonJAlcazarRNohHJRussellPAkenBAlföldiJAmemiyaCAzzouziNBaroillerJFBarloy-HublerFBerlinABloomquistRCarletonKLConteMAD'CottaHEshelOGaffneyLGalibertFGanteHFGnerreSGreuterLGuyonRHaddadNSHaertyWHarrisRMHofmannHAHourlierTHulataGJaffeDBLaraMLeeAPMacCallumIMwaikoSNikaidoMNishiharaHOzouf-CostazCPenmanDJPrzybylskiDRakotomangaMRennSCPRibeiroFJRonMSalzburgerWSanchez-PulidoLSantosMESearleSSharpeTSwoffordRTanFJWilliamsLYoungSYinSOkadaNKocherTDMiskaEALanderESVenkateshBFernaldRDMeyerAPontingCPStreelmanJTLindblad-TohKSeehausenODi PalmaF: The genomic substrate for adaptive radiation in African cichlid fish. *Nature*.2014;513(7518) : 10.1038/nature13726 375-381 10.1038/nature13726 25186727PMC4353498

# Response

**Published:** 2018-09-03

**Authors:** Johannes M. Dijkstra

**Affiliations:** Fujita Health University, Japan

Dear Dr. Jerzy Kulski,

 Thank you for your review and support of our article. We appreciate that an expert such as you is willing to join the public debate so that erroneous/unsubstantiated messages like the ones presented by Malmstrøm *et al.* can not take hold in our field.

 Your comments are very extensive and valuable, and we now refer the readers to them.

 Our special fields of scientific expertise are MHC genes and molecules, and in our first manuscript version we only concentrated on those. If there is no sufficient unity among the units used for mathematical modeling, that modeling, or the functional explanation of the resulting model, can never make sense. However, we now realized that for a large audience these MHC-specific issues might not be so clear, and that it is better to also address the questionable modeling methods used by Malmstrøm *et al. *Therefore, we now have added two paragraphs dedicated to this questionable modeling, titled “ *Detailed discussion of the use of the Ornstein-Uhlenbeck model by by Malmstrøm et al. ^1^*” and “ *Additional criticisms in regard to the modelling by Malmstrøm et al.* ^1^”. We realize that we use different language in regard to these topics than a theoretical biologist would use, but we hope that nevertheless we address the issues clearly and correctly.

 Sincerely,

 also on behalf of Dr. Unni Grimholt

 Hans (J.M.) Dijkstra

# Peer review: Referee response for version 1

**DOI:** 10.5256/f1000research.16766.r36541

**Published:** 2018-07-27

**Authors:** Brian Dixon

**Affiliations:** 1 Department of Biology, University of Waterloo, Waterloo, ON, Canada

The critique of Malstrøm et al. presented here makes some very valid points that are well supported by the literature.

 It has long been true in fish MHC research that the fact that a gene has not been reported to be present in a particular species does not mean that is not present. Modern genomics techniques has presented better proof for this assertion, with the lack of an MHCII/CD4 pathway in gadids being the most prominent example, but even modern genomics techniques are not iron clad 100% proof and should be checked very carefully before definitive statements are made. Thus the comments about verifying the presence or absence of specific genes in the numerous  species by other means is valid.

 Additionally, the treatment of all U and Z genes as identical units while ignoring the allelic diversity of each gene within those classes is indeed a serious flaw in the reasoning of Malstrøm et al. There is significant variability in diversity U gene families which will have differing effects on T cell selection that simply counting gene numbers will not address.

 Dijkstra and Grimholt's critique should be carefully read and addressed.

I have read this submission. I believe that I have an appropriate level of expertise to confirm that it is of an acceptable scientific standard.

# Response

**Published:** 2018-09-03

**Authors:** Johannes M. Dijkstra

**Affiliations:** Fujita Health University, Japan

Dear Dr. Brian Dixon,

 Thank you for your review and support of our article. We appreciate that an expert such as you is willing to join the public debate so that erroneous/unsubstantiated messages like the ones presented by Malmstrøm *et al.* can not take hold in our field.

 Sincerely,

 also on behalf of Dr. Unni Grimholt

 Hans (J.M.) Dijkstra

# Peer review: Referee response for version 1

**DOI:** 10.5256/f1000research.16766.r35833

**Published:** 2018-07-19

**Authors:** Anthony B. Wilson

**Affiliations:** 1 Department of Biology, Brooklyn College CUNY, New York, NY, USA; 2 The Graduate Center, City University of New York, New York, NY, USA

Dijkstra & Grimholt present a critical analysis of Malmstrom *et al.*'s 2016 *Nature Genetics* article ^1^, which investigated the evolution of MHC I and II loci in gadiform fishes using a low coverage genomic screen of 66 species, inferring a link between adaptive immune evolution and speciation rates in this group.  Dijkstra & Grimholt’s criticisms are wide ranging – I deal with each of their major areas of concern below:

**I. MHC Class II loss in gadiform fishes.**  The authors highlight two serious flaws in the Malmstrom analysis, demonstrating that the original dataset contains sequence reads of MHC II and associated loci in several species that were overlooked in the original analysis.  Equally importantly, datasets for several of the outgroup taxa lack these genes, raising questions concerning the reliability of the underlying data.  Malmstrom *et al.*'s genomic screen is understandably low coverage given the taxonomic breadth of their survey, but I agree with Dijkstra & Grimholt that based on the existing evidence, one cannot confidently infer the timing of MHC II gene loss in this group.

**II. MHC I allele counting strategy.**  Djikstra & Grimholt challenge the allele counting strategy used by Malmstrom et al, particularly their focus on U & Z loci (teleost fish have at least 5 different MHC I lineages ^2^), based on their assumption that these loci are chiefly involved in binding peptide ligands.  While I agree that grouping U & Z loci together simplifies their known functional complexity (I was rather confused by this approach myself when reading the original paper), here I feel that Djikstra & Grimholt could be more constructive in their criticism.  At present, its not entirely clear what type of analysis they would feel would be most suitable.  I would also suggest providing slightly more context on the study system to assist readers who may be unfamiliar with the original work. 

 While Dijkstra & Grimholt have elsewhere provided compelling evidence that Z loci may have a very different function, its not clear whether they’re suggesting that Malmstrom *et al.* should have focused solely on U loci, or whether it would have been more appropriate to include all MHC lineages in their analyses.  Either way, I would have liked to see whether analyzing the data in the manner preferred by the authors would impact the conclusions of the original article.

 I agree that experimental evidence would be necessary to conclusively demonstrate a link between allelic diversity and function, but given the taxonomic breadth of Malmstrom *et al.*'s study, surely they wouldn’t expect experimental evidence for all species included in the original study - How much experimental evidence would they deem sufficient?  At present, its not clear whether they’re simply suggesting that Malmstrom *et al.* should have been more circumspect in their conclusions, or whether they feel that the results of the analysis are entirely unreliable.  Clarification of this point is essential.

**III. Testing the relationship between MHC allelic diversity and speciation rates in gadiform fishes.**The authors raise concerns on the modelling approach used by Malmstrom *et al*., including their combined analysis of U and Z loci (see above), and their lack of a biologically realistic model of gene evolution, incorporating MHC gene gain and loss ^3^ – I agree with these criticisms.  I do, however, take some issue with their contention that Malmstrom *et al.*'s hypothesis is wholly invalid.  While there is indeed strong evidence of trans-species MHC polymorphism in some well-studied vertebrate lineages, this does not invalidate an experimental test of an alternative hypothesis.  If Dijkstra & Grimholt feel that Malmstrom *et al.* have their hypothesis “the wrong way around”, are there any data/analyses that could convince them otherwise?

I have read this submission. I believe that I have an appropriate level of expertise to confirm that it is of an acceptable scientific standard, however I have significant reservations, as outlined above.

## References

[1] MalmstrømMMatschinerMTørresenOKStarBSnipenLGHansenTFBaalsrudHTNederbragtAJHanelRSalzburgerWStensethNCJakobsenKSJentoftS: Evolution of the immune system influences speciation rates in teleost fishes. *Nat Genet*.48(10) : 10.1038/ng.3645 1204-10 10.1038/ng.3645 27548311

[2] GrimholtUTsukamotoKAzumaTLeongJKoopBFDijkstraJM: A comprehensive analysis of teleost MHC class I sequences. *BMC Evol Biol*.2015;15: 10.1186/s12862-015-0309-1 32 10.1186/s12862-015-0309-1 25888517PMC4364491

[3] NeiMRooneyAP: Concerted and birth-and-death evolution of multigene families. *Annu Rev Genet*.2005;39: 10.1146/annurev.genet.39.073003.112240 121-52 10.1146/annurev.genet.39.073003.112240 16285855PMC1464479

# Response

**Published:** 2018-09-03

**Authors:** Johannes M. Dijkstra

**Affiliations:** Fujita Health University, Japan

Dear Dr. Anthony B. Wilson,

 Thank you for your review and support of our article. We appreciate that an expert such as you is willing to join the public debate so that erroneous/unsubstantiated messages like the ones presented by Malmstrøm *et al.* can not take hold in our field.

 You suggest us to provide lay readers with more background information. Following your suggestion, we have tried to do so, by writing a tentative introduction section, but decided not to use it as we found that the necessary size and discussions would distort the article too much. This article is a correspondence, a discussion about another paper, and we feel that this discussion character should be clear throughout the article. Furthermore,  F1000Research proscribes that “Correspondence articles are short, peer reviewed comments”, and, as it is, the article is already quite lengthy.

 You ask us to be more constructive in our criticism towards Malmstrøm *et al.*, and to explain to them what they should have been doing instead. First of all, we would have liked them to make their claims on MHC class II system genes solid, because that seems within close experimental reach, and we believe that they should have concentrated their article on that topic. In regard to MHC class I, we would have liked them to either study those genes intensively, or to have refrained from any modeling, and especially to have refrained from highlighting a resulting model in the title. In the supplementary files 1 and 2 we already made some detailed comments about which experiments would be necessary to make the MHC class II system claims more solid and within acceptable standards. Now we also added a conclusion section to the main text which explains, in general terms, what we would like Malmstrøm *et al.* to do or not to do as follows:

**“Conclusion**

 “Malmstrøm *et al.* ^1^ used low-coverage genome sequencing for comparison of 66 mostly neoteleost fish, and so helped with elucidating their phylogeny. They found that intact MHC class II system genes may be completely absent in Gadiformes, and believed that related non-gadiform fishes have intact MHC class II system genes. However, their genomic databases were incomplete and in the case of many Gadiformes spiked with reads from MHC class II system genes that may or may not be contaminations, so that final conclusions require some additional analysis of at least a few species at the gadiform/non-gadiform clades border. We suggest that they need to perform a number of PCR and sequencing experiments to clarify this matter. When comparing class I and class II situations in their investigated neoteleosts, Malmstrøm *et al.* ^1^ also found that their earlier theory, which was that the absence of an MHC class II system might explain the high number of MHC class I genes in Atlantic cod ^13^, could not be corroborated. Instead, solely based on estimations of U+Z a3 fragment numbers, they ^1^ proposed a new theory on MHC class I evolution which they referred to in their manuscript title. We hope to have shown sufficiently that their conclusions on MHC class I evolution were unsubstantiated, that estimation of U+Z a3 fragment numbers is not a proper way to analyze MHC functions or MHC evolution, and that, apart from not investigating logical units that are better suited for their methods of modeling, also the number estimations and modelling systems used by Malmstrøm *et al.* ^1^ were flawed and/or non-trustworthy. Before any meaningful discussion can be started about the evolution of MHC class I genes in neoteleosts, a much higher level of information about sequences and genomic positions is necessary.”

 As to your question how we would have addressed the issue of MHC numbers/variation and thymic T cell depletion. The answer is that we probably wouldn’t try. Meaningful modeling would require a better understanding of how positive and negative T cell selections in the thymus, involving multiple different MHC molecules, contribute both quantitatively and qualitatively to the T cell pool. Regardless of the thymic selection model, we might be interested in MHC class I gene numbers, but then we would first want to separate the question into functional MHC subclasses. For example, it may be interesting to see whether there is some evolutionary pattern in the number of classical type polymorphic MHC class I genes (a question which Malmstrøm *et al.* erroneously seem to think that they were addressing), in the number of genes of nonclassical MHC class I families, or the number of MHC class I pseudogenes. Possibly, selection for increased diversification speeds may select for unstable haplotypes with many tandemly organized genes and pseudogenes that can function as a recombination reservoir. It would be interesting to see whether there are differences in numbers of MHC class I pseudogenes between those fish species that more stably maintained ancient variation and those that more rapidly acquired new variation. Basically, we would try to get good information on all investigated genes (and not just count a3 fragments), and then would try to answer questions one at the time and directly connected to the data (and not try to make an unsubstantiated overarching model). Without good data, we simply wouldn’t start modeling.

 I hope our above answer, and our extended criticism in the article main text of the models used by Malmstrøm *et al.*, is also sufficient as a response to the issues that you raised in paragraph III of your review.

 Sincerely,

 also on behalf of Dr. Unni Grimholt

 Hans (J.M.) Dijkstra
